# Novel 3‑Methoxypropanamide Derivatives as Potential Antiseizure and Antinociceptive Agents: Experimental Evidence from In Vitro and In Vivo Studies

**DOI:** 10.1021/acs.jmedchem.5c02093

**Published:** 2026-05-01

**Authors:** Marcin Jakubiec, Mirosław Zagaja, Katarzyna Socała, Michał Abram, Szczepan Mogilski, Gniewomir Latacz, Małgorzata Szafarz, Joanna Szala-Rycaj, Joanna Karnafał-Ziembla, Maja Kudrycka, Nikola Gapińska, Alan González Ibarra, Justyna Turek, Łukasz Gąsior, Bernadeta Szewczyk, Elżbieta Wyska, Marta Andres-Mach, Piotr Wlaź, Krzysztof Kamiński

**Affiliations:** † Department of Medicinal Chemistry, Faculty of Pharmacy, 49573 Jagiellonian University Medical College, Medyczna 9, 30-688 Cracow, Poland; ‡ Department of Experimental Pharmacology, 183620 Institute of Rural Health, Jaczewskiego 2, 20-090 Lublin, Poland; § Biomedical Research Laboratory, Institute of Biological Sciences, 49686 Maria Curie-Skłodowska University, Akademicka 19, 20-033 Lublin, Poland; ∥ Department Pharmacodynamics, Faculty of Pharmacy, Jagiellonian University Medical College, Medyczna 9, 30-688 Cracow, Poland; ⊥ Department of Chemical Technology and Biotechnology of Drugs, Faculty of Pharmacy, Jagiellonian University Medical College, Medyczna 9, 30-688 Cracow, Poland; # Pharmacokinetics and Preliminary Toxicological Analysis Laboratory, Center for the Development of Therapies for Civilization and Age-Related Diseases, Jagiellonian University Medical College, Skawińska 8, 30-688 Krakow, Poland; ¶ Department of Pharmacokinetics and Physical Pharmacy, Faculty of Pharmacy, Jagiellonian University Medical College, Medyczna 9, 30-688 Cracow, Poland; ∇ Doctoral School of Quantitative and Natural Sciences, Maria Curie-Skłodowska University, Weteranów 18, 20-038 Lublin, Poland; ○ Department of Molecular Biology, Institute of Biological Sciences, Maria Curie-Skłodowska University, Akademicka 19, 20-033 Lublin, Poland; ⧫ Department of Neurobiology, 69714 Maj Institute of Pharmacology, Polish Academy of Sciences, Smętna 12, 31-343 Kraków, Poland

## Abstract

In this study, novel derivatives based on the 3-methoxypropanamide core were designed, synthesized, and evaluated *in vitro* and *in vivo*. These compounds demonstrated broad-spectrum antiseizure activity, with **(*R*)-** **46** emerging as the lead candidate. Following intraperitoneal administration, **(*R*)-** **46** showed ED_50_ values of 35.6 mg/kg (MES), 8.4 mg/kg (6 Hz, 32 mA), and 19.1 mg/kg (6 Hz, 44 mA), significantly elevating seizure thresholds in multiple models without affecting grip strength. Chronic treatment suppressed seizure progression in the PTZ kindling model, with minimal impact on hippocampal inflammatory markers or amino acids profile. Importantly, **(*R*)-** **46** exhibited antinociceptive effects in formalin-, capsaicin-, oxaliplatin- and streptozotocin-induced pain models. Pharmacokinetic and *in vitro* ADME-Tox studies indicated favorable drug-like properties. Mechanistic studies suggest dual mode of action, including 5-HT_2C_ receptor agonism and inhibition of voltage-gated sodium channels. These results support further preclinical development of **(*R*)-** **46** as a promising candidate for treating epilepsy and pain-related disorders.

## Graphical abstract

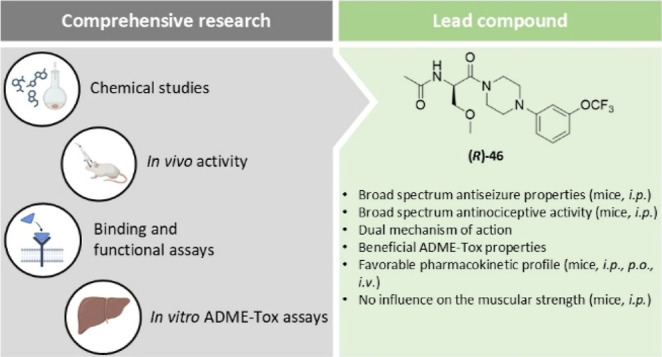

## Introduction

Epilepsy is one of the commonest neurological disorders with frequency of occurrence about 1% in the population and is characterized by a predisposition to recurrent unprovoked seizures resulting from abnormal brain bioelectrical activity. Importantly, this harmful neurological condition significantly reduces patients’ quality of life at all levels—personal, professional, and social.^1,2^ Despite the availability of more than 40 antiseizure medications (ASMs), approximately one-third of patients suffer from drug-resistant epilepsy (DRE), defined as failure to achieve sustained seizure freedom despite adequate trials of at least two appropriately chosen and tolerated ASMs.^3^ DRE is associated with substantially increased risks of premature mortality, cognitive impairment, injuries, psychiatric comorbidities, and reduced quality of life.

When monotherapy fails, polytherapy is often employed, combining ASMs with complementary mechanisms of action and favorable safety profiles. However, this approach frequently introduces complications such as pharmacokinetics, a higher risk of drug–drug interactions (DDIs), and reduced patient compliance. In response to these limitations, the development of multifunctional (multitarget) drugs has emerged as a promising strategy.^4,5^ These compounds are designed to simultaneously engage multiple molecular targets involved in seizure initiation and propagation, potentially offering improved therapeutic outcomes with fewer side effects. This strategy offers several advantages, including more predictive pharmacokinetics, improved patient compliance, and a reduced risk of DDIs.^6–8^ Multitarget ligands are increasingly explored for multifactorial diseases such as Alzheimer’s and Parkinson’s disease, depression, schizophrenia, cancer, inflammation, and neurological disorders, including epilepsy and neuropathic pain.^9–16^ Moreover, multifunctional compounds may be effective in the treatment of high drug resistance diseases, including neurological disorders such as epilepsy, as well as infectious diseases caused by pathogens such as bacteria or fungi.^17–19^ Taking the above facts, epilepsy is undoubtedly one of the most complex neurological disorders, characterized by a multifactorial etiology, a complex pathomechanism, and a high risk of drug resistance. Therefore, current research efforts are increasingly focused on the development of novel ASMs with broad-spectrum efficacy and multitarget profiles, particularly those capable of addressing the unmet needs in DRE management.

Following the multitarget-oriented design concept, we have recently created several series of hybrid molecules built on the pyrrolidine-2,5-dione core, with the most promising representatives being exemplified by the following compounds (**KA-11** and **(*R*)-KA-138**),^20,21^ as well as non-imide analogues such as **KJ-79** (and its enantiomers described in the patent WO2025136133) and **(*R*)-KJ-28**.^22,23^ These compounds, designed as modified amino acid derivatives based on α-alanine (**KA-11** and **KJ-79**) or phenylglycine (**(*R*)-KA-138** and **(*R*)-KJ-28**), were synthesized to explore the effect of this structural modification on antiseizure efficacy (Figure 1). Importantly, our previous work confirmed the potential of hybrid molecules as promising drug candidates with broad-spectrum activity in animal seizure models, particularly in the maximal electroshock seizure (MES) test and the 6 Hz psychomotor seizure models (32 and 44 mA). The *in vivo* profiles observed for these compounds suggest their potential utility in treating diverse forms of epilepsy, including tonic–clonic seizures with or without secondary generalization, focal onset seizures and notably DRE.

**Figure 1:**
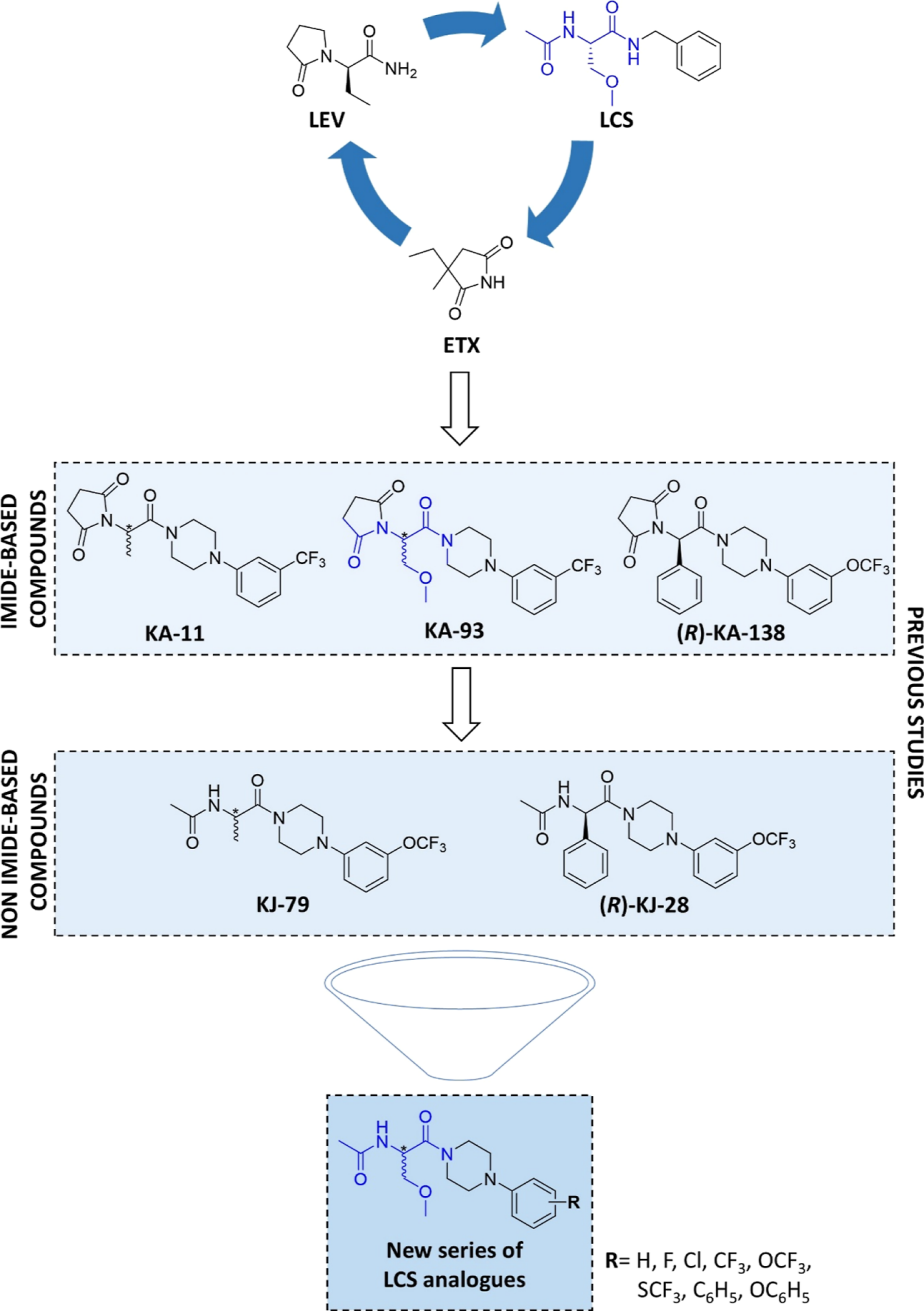
Scientific rationale and development process yielding new hybrid molecules reported herein.

Originally, compounds bearing the pyrrolidine-2,5-dione scaffold were rationally designed to incorporate structural fragments from three pharmacologically distinct ASMs: ethosuximide (ETX), effective exclusively in the *sc*PTZ model; levetiracetam (LEV), active in the 6 Hz (32 mA) model; and lacosamide (LCS), which shows efficacy in both the MES and 6 Hz (32/44 mA) tests. As a result, one of the most effective compounds developed previously, **KA-93**—a closer analogue of LCS, demonstrated potent protective effects in the MES and 6 Hz (32 mA) models.^24^ However, its efficacy in the more pharmacoresistant 6 Hz (44 mA) model was limited (ED_50_ = 101.5 mg/kg; see Figure 1), despite incorporating the 3-methoxypropanamide fragment characteristic of LCS.

To address this weakness, our current study focused on enhancing activity in the 6 Hz (44 mA) model by introducing further structural modifications aimed at more closely mimicking the structure of LCS, a model ASM effective in this test. Specifically, the succinimide ring present in **KA-93** was replaced with an acetyl group, an essential structural feature of LCS, while maintaining key fragments of the original **KJ-79** and **(*R*)-KJ-28** compounds. This included the addition of a methoxy group to the **KJ-79** core and the replacement of the phenylglycine moiety in **(*R*)-KJ-28** with a 3-methoxypropanamide fragment. Through this rational design, we aimed to improve protective activity across all key seizure models, with particular emphasis on the challenging 6 Hz (44 mA) test.

Based on our previous studies, guided by structure–activity relationship (SAR) analysis and in an effort to minimize animal use, we restricted our chemical space to derivatives bearing electron-withdrawing substituents (namely F, Cl, CF_3_, C_6_H_5_, OCF_3_, OC_6_H_5_, SCF_3_) at the 3- or 4-positions or 3,4- and 3,5-disubstituted phenylpiperazine derivatives (as preferential for antiseizure activity).^20,22–25^

To facilitate more advanced preclinical evaluation, we additionally synthesized the *R*- and *S*-enantiomers of the most active racemic candidates identified in this study. This allowed us to establish which spatial configuration is favored for optimal biological activity. Notably, developing the most active stereoisomers is essential for the further progression of these compounds toward clinical evaluation.

The studies presented herein describe a comprehensive approach to the discovery of novel ASM candidates. This approach encompasses the design, chemical synthesis, and extensive *in vivo* evaluation of antiseizure activity within a focused library of compounds. For the most potent enantiomers, preliminary ADME-Tox profiling was conducted using a series of standard assays, including assessments of membrane permeability, metabolic stability, hepatotoxicity, neurotoxicity, potential interactions with cytochrome P450 isoforms CYP3A4 and CYP2D6, as well as the formation of reactive metabolites and the induction of phospholipidosis. Moreover, for the lead compound, additional studies were conducted to determine protein binding in mouse plasma as well as tissue binding in mouse brain. Furthermore, the lead compound was evaluated in the pentylenetetrazole (PTZ) kindling model and a mouse seizure threshold tests to confirm its anticonvulsant efficacy. Pharmacokinetic and antinociceptive studies were also performed for the most promising candidate. To further elucidate the mechanism of action, the most effective compound underwent detailed *in vitro* binding and functional assays.

## Results and Discussion

### Chemistry

In the chemical studies, a library of 17 racemates and 3 pairs of enantiomers were obtained (Scheme 2). The enantiomers were synthesized for the 3 most active racemic compounds. All compounds were designed following drug-like physicochemical properties based on Lipinski (RO5) and Veber rules using the SwissAdme software,^26,27^ as well as central nervous system multiparameter optimization (CNS MPO) algorithm.^28^ The physicochemical properties of all obtained racemates are shown in Table S1. The final compounds (racemates and its enantiomers) were obtained by applying the multistep synthetic procedure that also involved the preparation of selected noncommercial amines (**A10**–**A18**), which were synthesized in a two-step reaction according to Scheme 1. First, intermediates **A1**–**A9** were obtained by a reaction of aryl bromides with 1-Boc-piperazine in the Buchwald–Hartwig amination reaction in the nitrogen atmosphere.^29^ The removal of the Boc group in acid conditions (TFA) followed by neutralization with 25% ammonium hydroxide yielded the desired 1-phenylpiperazine derivatives **A10**–**A18** which were used for further reactions without purification.

**Scheme 1:**
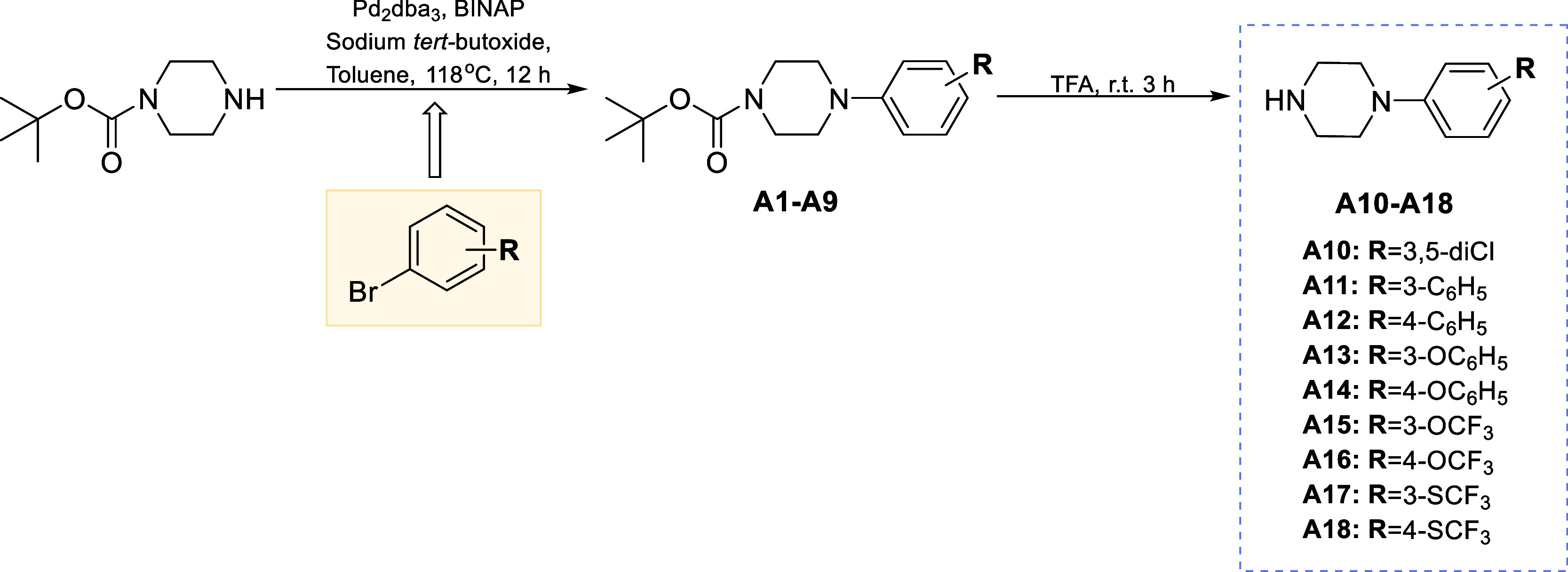
Synthesis of Boc-Protected Intermediates (**A1–A9**) and Non-commercial 4-Arylpiperazine Derivatives **A10–A18**

The final compounds were synthesized according to Scheme 2. First, the condensation reaction of appropriate 1-phenylpiperazine derivatives (commercial or noncommercial, **A10**–**A18**), with Boc-*O*-methyl-dl-serine (as racemate) or Boc-*O*-methyl-d-serine (as *R*-enantiomer) or Boc-*O*-methyl-l-serine (as *S*-enantiomer) in the presence of DCC yielded intermediates **(*R*,*S*)-1**–**(*R*,*S*)-17**, **(*R*)-10**, **(*S*)-10**, **(*R*)-12**, **(*S*)-12**, **(*R*)-16** and **(*S*)-16**. In the next step, as a result of removal of the Boc-protecting group by the addition of TFA, the amine derivatives **(*R*,*S*)-18**–**(*R*,*S*)-34**, **(*R*)-27**, **(*S*)**-**27**, **(*R*)-29**, **(*S*)-29**, **(*R*)-33** and **(*S*)-33** were obtained. The target compounds **(** ***R*,*S*)-35**–**(*R*,*S*)-51**, **(*R*)-44**, **(*S*)-44**, **(*R*)-46**, **(*S*)-46**, **(*R*)-50** and **(*S*)-50** were obtained in an acylation reaction of amine intermediates by acetyl chloride. The crude reaction mixtures were purified by column chromatography. After evaporation of the organic solvents under reduced pressure and a final washing with diethyl ether, the desired compounds were obtained as white solids.

**Scheme 2:**
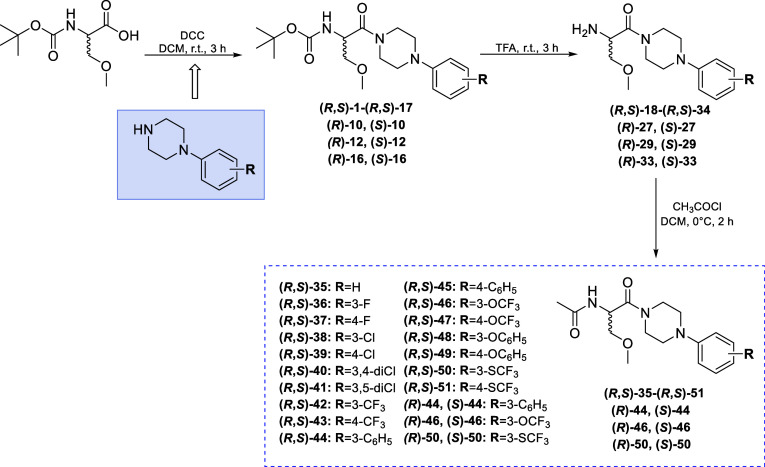
General Synthetic Route for Target Compounds **(*R*,*S*)-35**–**(*R*,*S*)-51**, **(*R***)**-44**, **(*S***)**-44,** **(*R*)-46**, **(*S*)-46**, **(*R*)-50**, and **(*S*)-50**

All final compounds were obtained in good yields (>85%). Their structures were confirmed by ^1^H NMR and ^13^C NMR spectra analysis. Moreover, LC–MS spectra were obtained for all intermediates and final compounds, with the purity determined by UPLC method exceeding 98% (for final compounds). High-resolution mass spectrometry (HRMS) analysis was also conducted for the most potent compounds. The enantiomeric purity of **(*R*)-44**, **(*S*)-44**, **(*R*)-46**, **(*S*)-46**, **(*R*)-50** and **(*S*)-50** exceeded 98% as determined by chiral supercritical fluid chromatography (SFC) analysis. To confirm the enantiomeric purity, the chiral SFC resolution was performed also for the respective racemate, namely **(*R,S*)-44**, **(*R,S*)-46** and **(*R,S*)-50**. The physicochemical and spectral data for intermediates and the final compounds are summarized in the Materials and Methods section. The procedure for synthesis of starting amines **A1–A18** and analytical data are described in Supporting Information.

### Antiseizure Activity

The therapeutic effectiveness of novel ASM candidates continues to be primarily evaluated through phenotypic, target-independent methodologies.^30,31^ In support of this strategy, the Epilepsy Therapy Screening Program (ETSP) conducted by the National Institute of Neurological Disorders and Stroke (NIH, Bethesda, MD, USA), has played a key role in the identification and development of several clinically approved ASMs by emphasizing *in vivo* evaluations.^32^ Among the variety of seizure models employed in preclinical research, a select group is widely regarded as the gold standard. These include the MES model, which replicates tonic-clonic epilepsy and partial convulsions with or without secondary generalization, the 6 Hz (32 mA) seizure model, which corresponds to partial-onset seizures, and the 6 Hz (44 mA), which is recognized as a model of human DRE.^33,34^ In this study, all final racemates (*R*,*S* **)-35**–(*R*,*S* **)-51** were subjected to preliminary evaluation in all three tests, namely the MES and 6 Hz (32/44 mA) models following intraperitoneal (*i.p.*) administration at a dose of 100 mg/kg in mice, 30 min postinjection, with each treatment group consisting of four animals. The outcomes of these experiments are presented in Table S2.

According to the MES screening data, the maximal 100% antiseizure protection (four out of four mice were protected) was demonstrated for **(*R*,*S*)-42**, **(*R*,*S*)-44**, **(*R*,*S*)-46**, **(*R*,*S*)-48**-**(*R*,*S*)-50**. Still satisfactory 75% protection (three out of four mice were protected), was demonstrated for compound **(*R*,*S*)-47**. Another molecule, **(*R*,*S*)-41** demonstrated 50% seizure protection, whereas **(*R*,*S*)-51** showed 25% protection. The remaining compounds showed no activity in this seizure model.

In the 6 Hz (32 mA) model, the tested compounds exhibited significantly stronger protection. The highest protection (100%) was observed for **(*R*,*S*)-** **38**, **(*R*,*S*)-** **41**, **(*R*,*S*)-** **42**, **(*R*,*S*)-** **44**, **(*R*,*S*)-** **46**, **(*R*,*S*)-** **47**, **(*R*,*S*)-** **49** and **(*R*,*S*)-** **50**, whereas compounds **(*R*,*S*)-** **43**, **(*R*,*S*)-** **48** and **(*R*,*S*)-** **51** were also active and protected 75% of mice. Compounds **(*R*,*S*)-** **36**, **(*R*,*S*)-** **40** and **(*R*,*S*)-** **45** showed 50% efficacy in this seizure model, whereas compound **(*R*,*S*)-** **37** showed limited protection (25%). Notably, only 2 compounds (**(** ***R***, ***S*** **)-35** and **(*R*,*S* **)-39)****) were devoid of any protection in the 6 Hz (32 mA) test.

The subsequent phase of the *in vivo* screening involved evaluating all compounds using the 6 Hz (44 mA) model to confirm their broad-spectrum antiseizure efficacy. The maximal 100% antiseizure protection was demonstrated for **(*R*,*S*)-42**, **(*R*,*S*)-44**, **(*R*,*S*)-46**-**(*R*,*S*)-48** and **(*R*,*S*)-50**. Weaker, nevertheless satisfying activity was provided by **(*R*,*S*)-41**, **(*R*,*S*)-43**, **(*R*,*S*)-49** and **(*R*,*S*)-51**. Only 50% efficacy in this seizure model was noted for **(*R*,*S*)-37**, **(*R*,*S*)-38** and **(*R*,*S*)-45**, whereas **(*R*,*S*)-36** and **(*R*,*S*)-40** showed 25% protection. Other molecules showed no activity in this model.

In conclusion, the screening results indicate that the strongest antiseizure effects were shown by compounds bearing electron-withdrawing substituents at the 3-position of the phenylpiperazine moiety, including such examples as –CF_3_, –C_6_H_5_ -OCF_3_, –OC_6_H_5_, –SCF_3_ and 4-position of the phenylpiperazine moiety, specifically –OCF_3_, –OC_6_H_5_.

In the next step of the pharmacological profiling, the median effective doses (ED_50_) were determined for compounds showing minimum 75% protection at the dose of 100 mg/kg in each model, namely MES and 6 Hz (32/44 mA) tests. To evaluate the safety profile at 0.5 h post administration, the median toxic doses (TD_50_) were assessed using the chimney test, a standard method for detecting motor coordination impairments. Protective indexes (PIs), serving as indicators of the therapeutic window and benefit-risk ratio of the tested compounds, were then calculated using the ED_50_ and TD_50_ values (PI = TD_50_/ED_50_). These data are presented in Table 1, alongside with previously reported data for clinically established ASMs used as references: LEV, which is effective in the 6 Hz (32 mA) model; LCS, active in MES and 6 Hz (32/44 mA) models; and valproic acid (VPA), a well-known broad-spectrum ASM effective in the MES and 6 Hz (32/44 mA) tests.

**Table 1:** ED_50_, TD_50_, and PI Values for Racemates and Model ASMs (Mice, *i.p.*)­^a^

cmpd	PT (h)^b^	ED_50_ MES (mg/kg)	ED_50_ 6 Hz (32 mA)(mg/kg)	ED_50_ 6 Hz (44 mA)(mg/kg)	TD_50_ (mg/kg)	PI (TD_50_/ED_50_)
**(*R,S*)-42**	0.5	68.4 (62.9–74.3)	35.3 (29.0–43.1)	35.2 (23.5–52.7)	164.5* (152.4–177.5)	2.4 (MES)
						4.7 (6 Hz, 32 mA)
						4.7 (6 Hz, 44 mA)
**(*R,S*)-44**	**0.5**	**32.0 (28.5–55.0)**	**15.8 (10.8–23.2)**	**23.4 (18.5–29.6)**	65.3* (53.1–80.5)	**2.0 (MES)**
						**4.1** **(6 Hz, 32 mA)**
						**2.8** **(6 Hz, 44 mA)**
**(*R,S*)-46**	**0.5**	**38.3 (34.9–42.1)**	**10.0 (5.6–17.8)**	**22.9 (12.3–42.6)**	86.7* (79.7–94.2)	**2.3 (MES)**
						**8.7** **(6 Hz, 32 mA)**
						**3.8** **(6 Hz, 44 mA)**
**(*R,S*)-47**	0.5	76.3 (65.6–88.6)	24.5 (16.6–36.3)	24.5 (16.7–36.3)	152.7* (133.1–175.2)	2.0 (MES)
						6.2 (6 Hz, 32 mA)
						6.2 (6 Hz, 44 mA)
**(*R,S*)-48**	0.5	53.0 (51.2–55.0)	26.0 (20.0–33.6)	37.1 (27.8–49.4)	122.2* (107.6–138.8)	2.3 (MES)
						4.7 (6 Hz, 32 mA)
						3.3 (6 Hz, 44 mA)
**(*R,S*)-49**	0.5	66.5 (61.8–71.6)	20.2 (15.0–27.0)	23.9 (17.2–33.1)	98.4* (82.8–116.8)	1.5 (MES)
						4.9 (6 Hz, 32 mA)
						4.1 (6 Hz, 44 mA)
**(*R,S*)-50**	**0.5**	**37.5 (32.5–43.2)**	**14.3 (10.2–20.1)**	**18.3 (13.7–24.3)**	111.3* (100.5–123.2)	**3.0 (MES)**
						**7.8** **(6 Hz, 32 mA)**
						**6.1** **(6 Hz, 44 mA)**
**VPA** ^c^	0.5	252.7 (220.1–290.2)	130.6 (117.6–145.2)	183.1 (143.5–233.7)	430.7** (407.9–454.9)	1.7 (MES)
						3.3 (6 Hz, 32 mA)
						2.3 (6 Hz, 44 mA)
**LEV** ^c^	1.0	>500	15.7 (11.2–18.4)	204.0 (154.5–269.5)	>500**	>31.5 (6 Hz, 32 mA)
						>2.5 (6 Hz, 44 mA)
**LCS** ^c^	0.5	9.2 (8.5–10.0)	5.3 (3.5–7.8)	6.9 (5.4–8.6)	46.2** (44.5–48.0)	5.0 (MES)
						8.8 (6 Hz, 32 mA)
						6.7 (6 Hz, 44 mA)

a **Data for the most potent compounds have been bolded for better visualization**. Results are represented as median ±95% confidence interval. ED_50_, median effective dose. TD_50_, median toxic dose determined in the *chimney test or the **rotarod test. PI, Protective index, TD_50_/ED_50_.

b Pretreatment time.

c Reference ASMs: valproic acid (VPA), levetiracetam (LEV) and lacosamide (LCS) data taken from own experiments. No mortality was observed in the MES model among the tested compounds.

The data obtained demonstrated that all tested racemates effectively protected mice against seizures in all acute animal models of seizures, namely MES and 6 Hz (32/44 mA) tests. Based on the ED_50_, TD_50_, and PI values, data confirmed that substitution at the 3-position of the phenylpiperazine fragment is preferred (**(*R*,*S*)-46** vs **(*R*,*S*)-47** and **(*R*,*S*)-48** vs **(*R*,*S*)-49**). The most potent protection in the aforementioned seizure tests was shown for **(*R*,*S*)-44**, **(*R*,*S*)-46** and **(*R*,*S*)-50**. These compounds are about 2-fold more potent in comparison to other compounds reported herein and most of them have more favorable PIs. It is noteworthy that **(*R*,*S*)-44**, **(*R*,*S*)-46** and **(*R*,*S*)-50** showed almost 10-fold better potency vs LEV in the 6 Hz (44 mA) model of DRE and similar or even better in the 6 Hz (32 mA) seizure model. These compounds were also more effective in all seizure models and possessed more beneficial PI than VPA. Nevertheless, **(*R*,*S*)-44**, **(*R*,*S*)-46** and **(*R*,*S*)-50** exhibited lower potency in all three tests and safety margin compared to LCS.

Based on the potent protection in the MES and 6 Hz (32/44 mA) seizure models of **(*R*,*S*)-44**, **(*R*,*S*)-46** and **(*R*,*S*)-50**, in the next step we obtained and tested *in vivo* its *R*- and *S*-enantiomers in order to determine the preferred spatial configuration for biological activity (Table 2). It is important to emphasize that individual enantiomers (or diastereoisomers) of drug candidates may exhibit markedly different pharmacodynamic, pharmacokinetic, and safety profiles.^35^ Employing a chiral-switch strategy for existing racemic drugs, or developing new molecules directly in their defined enantiomeric or diastereoisomeric form, is a valuable medicinal chemistry approach that can enhance efficacy and/or reduce toxicity. This tendency is well illustrated by several ASMs, such as LEV, LCS, and eslicarbazepine, which were introduced into clinical use specifically as single enantiomers.^36^

**Table 2:** ED_50_, TD_50_, and PI Values for Enantiomers and Model ASMs (Mice, *i.p.*)­^a^

cmpd	PT (h)^b^	ED_50_ MES (mg/kg)	ED_50_ 6 Hz (32 mA) (mg/kg)	ED_50_ 6 Hz (44 mA) (mg/kg)	TD_50_ (mg/kg)	PI (TD_50_/ED_50_)
						
**(*R*)-44**	0.5	28.2 (24.9–32.0)	9.1 (5.7–14.5)	10.0 (5.6–17.8)	69.4* (61.4–78.3)	2.5 (MES)
						7.6 (6 Hz, 32 mA)
						6.9 (6 Hz, 44 mA)
**(*S*)-44**	0.5	31.6 (26.0–38.5)	18.0 (11.2–29.0)	26.0 (20.0–33.6)	65.7* (60.3–71.6)	2.1 (MES)
						3.7 (6 Hz, 32 mA)
						2.5 (6 Hz, 44 mA)
**(*R*)-46**	**0.5**	**35.6 (31.9–39.7)**	**8.4 (3.8–18.5)**	**19.1 (12.9–28.2)**	87.2* (76.7–99.0)	**2.4 (MES)**
						**10.4** **(6 Hz, 32 mA)**
						**4.6** (6 Hz, 44 mA)
**(*S*)-46**	0.5	42.7 (39.4–46.4)	14.7 (10.2–21.3)	22.7 (16.7–30.9)	98.4* (82.8–116.8)	2.3 (MES)
						6.7 (6 Hz, 32 mA)
						4.4 (6 Hz, 44 mA)
**(*R*)-50**	0.5	33.5 (27.9–40.2)	12.1 (7.2–20.6)	18.2 (13.3–24.9)	98.4* (82.8–116.8)	2.9 (MES)
						8.1 (6 Hz, 32 mA)
						5.2 (6 Hz, 44 mA)
**(*S*)-50**	0.5	36.5 (30.9–43.2)	14.4 (10.8–19.3)	20.0 (15.2–26.4)	94.8 (90.7–99.2)	2.6 (MES)
						6.6 (6 Hz, 32 mA)
						4.7 (6 Hz, 44 mA)
**VPA** ^c^	0.5	252.7 (220.1–290.2)	130.6 (117.6–145.2)	183.1 (143.5–233.7)	430.7** (407.9–454.9)	1.7 (MES)
						3.3 (6 Hz, 32 mA)
						2.3 (6 Hz, 44 mA)
**LEV** ^c^	1.0	>500	15.7 (11.2–18.4)	204.0 (154.5–269.5)	>500**	>31.5 (6 Hz, 32 mA)
						>2.5 (6 Hz, 44 mA)
**LCS** ^c^	0.5	9.2 (8.5–10.0)	5.3 (3.5–7.8)	6.9 (5.4–8.6)	46.2** (44.5–48.0)	5.0 (MES)
						8.8 (6 Hz, 32 mA)
						6.7 (6 Hz, 44 mA)

a **Data for the most potent compound has been bolded for better visualization**. Results are represented as median ±95% confidence interval. ED_50_, median effective dose. TD_50_, median toxic dose determined in the *chimney test or the **rotarod test. PI, Protective index, TD_50_/ED_50_.

b Pretreatment time.

c Reference ASMs: valproic acid (VPA), levetiracetam (LEV) and lacosamide (LCS) data taken from own experiments. No mortality was observed in the MES model among the tested compounds.

The data obtained revealed very potent protection for all tested enantiomers. Importantly, these data proved more beneficial antiseizure protection for *R*-enantiomers (eutomers) compared to *S*-enantiomers (distomers) in MES and 6 Hz (32/44 mA) seizure models. Furthermore, the *R*-enantiomers were also characterized by more favorable PIs compared to the *S*-enantiomers. The most potent protection across MES and 6 Hz (44 mA) was noted for, **(*R*)-44**, however in 6 Hz (32 mA) test **(*R*)-46** proved to be the most effective. In comparison to **(*R*)-44**, **(*R*)-46** showed significantly lower impact on motor coordination (TD_50_ value). For **(*R*)-44**, despite showing better efficacy and PI in the 6 Hz (44 mA) model, the low TD_50_ values observed suggest a higher potential for neurotoxicity and related adverse effects, which could limit its therapeutic window and overall development potential. On the other hand **(*R*)-50**, also demonstrated good activity, comparable to **(*R*)-46**. However, previous studies on glycinamide derivatives^22^ have indicated an increased risk of hepatotoxicity of the analogue possessing the 3-SCF_3_ substituent, which raised concerns regarding its further development. Another important feature supporting the selection of **(*R*)-46** over **(*R*)-50** is that the SCF_3_ moiety present in **(*R*)-50** is known to be susceptible to oxidative biotransformation. Oxidation of sulfur has been shown to occur *in vivo*, potentially altering both the pharmacokinetic properties and biological activity of a compound, thereby raising further uncertainty about the stability and predictability of **(*R*)-50**’s pharmacological profile.^37^ Taking these factors into consideration, **(*R*)-46** was selected as the lead compound within this focused library, as it demonstrated the most balanced efficacy–safety profile, making it the most suitable candidate for extensive characterization. Furthermore, it should be stressed that **(*R*)-46** was more potent and showed higher PI values than VPA. On the other hand, LCS one of the chemical precursors for **(*R*)-46**, showed the best effectiveness of all obtained substances, however due to significant impact on motor coordination, PI value were a little less favorable in 6 Hz (32 mA) test, a little more favorable 6 Hz (44 mA) in comparison to **(*R*)-46**. It is noteworthy that **(*R*)-46** showed 10-fold better potency vs LEV in the 6 Hz (44 mA) model of DRE and almost 2-fold in the 6 Hz (32 mA) seizure model. The very promising antiseizure activity of **(*R*)-46** in the MES and 6 Hz models led to further evaluation in additional *in vitro* and *in vivo* experiments assessing its pharmacokinetic properties, safety, and efficacy.

To evaluate the impact of structural modifications described herein on antiseizure activity, the pharmacological profiles of several previously synthesized hit compounds were compared with the lead compound **(*R*)-46**, as outlined below.

As shown in Table 3, **(*R*)-46** exhibited strong activity in both MES and 6 Hz seizure models, with particularly beneficial ED_50_ values combined with a favorable safety profile. **(*R*)-46**, which has a methoxymethylene fragment in place of the methyl group present in its close analogue **(*R*)-KJ-79**, showed comparable activity in the MES test and increased potency in the 6 Hz models. Although **(*R*)-KJ-79** showed comparable influence on motor coordination, its protective indices were modestly lower than those of **(*R*)-46**, particularly in the 6 Hz 44 mA test. Compound **(*R*)-KJ-28** (also non-imide analogue), which has an additional aromatic ring in the structure, displayed diminished activity across all models, most notably in the MES test. Although it showed relatively low influence on motor coordination its PIs in all three aforementioned seizure models were clearly lower than those observed for **(*R*)-46**, indicating a narrower therapeutic window. These results suggest that the structural modification in **(*R*)-46** positively enhanced both potency and therapeutic potential compared to **(*R*)-KJ-28**. On the other hand, **KA-93** (closest imide analogue), showed the highest ED_50_ values in all models, particularly in the 6 Hz (32 mA) test, indicating the lowest potency among all compounds. Although, it showed relatively low influence on motor coordination it results in a relatively low PIs. **(*R*)-46**, showed almost 2.5-fold better potency vs **KA-93** in MES test and almost 5-fold better potency in the 6 Hz (32 mA) and 6 Hz (44 mA) seizure models. This data confirmed that exchange the succinimide ring in **KA-93** to the acetyl group (see Figure 1), which appears in the structure of LCS, significantly enhanced the protection in the 6 Hz (44 mA) model of DRE and additionally increased activity in other electrically induced seizures, namely MES and 6 Hz (32 mA).

**Table 3:**
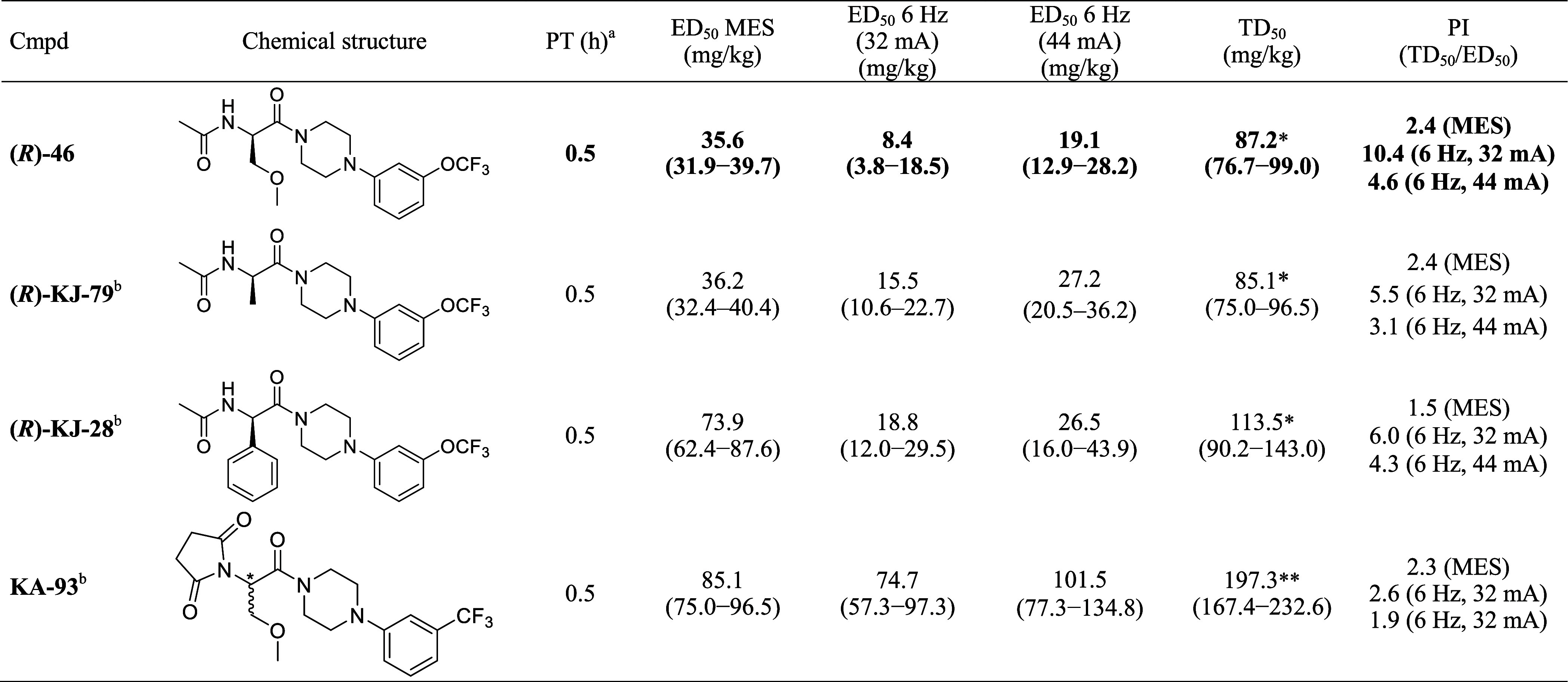
ED_50_, TD_50_, and PI Values for Previously Synthesized Hit Compounds **(*R*)-KJ-79**, **(*R*)-KJ-28, KA-93** and Lead Compound **(*R*)-46** (Mice, *i.p.*)

Data for the **(*R*)-46** has been bolded for better visualization. Results are represented as median ±95% confidence interval. ED_50_, median effective dose. TD_50_, median toxic dose determined in the *chimney test or the **rotarod test. PI, Protective index, TD_50_/ED_50_.

a Pretreatment time.

b Data for compounds: **(*R*)-KJ-79** described in the patent WO2025136133; **(*R*)-KJ-28** described as compound (*R*)-32 from ref;^22^ **KA-93** described as compound 30 from ref.^24^

In summary, while structural modifications introduced in **(*R*)-KJ-79**, **(*R*)-KJ-28**, and **KA-93** influenced potency and motor coordination to varying degrees, none surpassed the lead compound **(*R*)-46** in terms of overall activity and PIs. These results confirm **(*R*)-46** as the most promising compound in all our chemical library and a strong candidate for further development.

### *In Vitro* ADME-Tox Assays

The differences in biological activity as well as pharmacokinetic or toxicological properties of enantiomers can be also observed in *in vitro* studies.^38^ Although enantiomers share the same chemical composition and molecular weight, they frequently exhibit distinct ADME-Tox profiles due to their differential interactions with biological targets such as enzymes, transporters, and receptors. Therefore, to ensure the selection of the most suitable compound for further development, a comparative assessment of selected ADME-Tox properties was performed at this stage. This approach enabled the identification of the enantiomer with the most favorable safety profile, providing a rational basis for its advancement into subsequent preclinical studies. Thus, the series of *in vitro* ADME-Tox assays were used here in order to determine and compare the drug-like properties of obtained within this study enantiomers **(*R*)-46** and **(*S*)-46**. The ADME-Tox parameters were shown in Table 4.

**Table 4:** ADME-Tox Parameters Determined *In Vitro* for **(*R*)-46** and **(*S*)-46**

ADME-tox assay *in vitro*	**(*R*)-46**	**(*S*)-46**	reference drug
PAMPA – Pe (10^–6^ cm/s) ± SD	6.0 ± 2.2	6.8 ± 0.8	6.3 ± 1.1 (caffeine)
PAMPA membrane retention *R* (%)	8.5	7.4	2.4 (caffeine)
plasma protein binding – fraction bound f_b_ (% ± SD)	72.1 ± 11.3	70.6 ± 12.0	98.5 ± 2.1 (warfarin)
MLM t_0.5_ [min]	128.4	130.8	22.0 (verapamil)
MLM Cl_int_ [mL/min/kg]	42.3	41.5	239.5 (verapamil)
CYP3A4 activity (% of control ±SD at 10 μM)	95.8 ± 1.5	97.4 ± 8.1	5.1 ± 0.2 [1 μM] (ketoconazole)
CYP2D6 activity (% of control ±SD at 10 μM)	107.9 ± 0.6	109.3 ± 1.0	4.4 ± 0.7 [1 μM] (quinidine)

PAMPA assay did not show marked differences in passive transport through biological membrane between the tested enantiomers. What is more, the obtained permeability coefficients Pe’s for **(*R*)-46** and **(*S*)-46** were excellent and similar to the well permeable reference caffeine.

Plasma protein binding test showed that the protein-bound fraction (f_b_) is quite low compared to the reference drug warfarin (98.5%), with a similar value of about 70% for both enantiomers. However, according to the information provided by the manufacturer of used TRANSIL PPB Binding Kit, the f_b_ for these compounds may be even more favorable due to the limitations related to the low precision of the results for compounds weakly bound (below 70% to plasma proteins, see Supporting Information).

Metabolic stability was tested with use of mouse liver microsomes (MLMs). Both compounds were quite stable in MLMs with t_0.5_ = 128.4 and 130.8 min and calculated intrinsic clearances Cl_int_ = 42.3 mL/min/kg and 41.5 mL/min/kg for enantiomer (*R*) and (*S*), respectively. The t_0.5_ and Cl_int_ parameters for the unstable reference verapamil incubated under the same conditions were 22 min and 239.5 mL/min/kg. However, despite of the similar t_0.5_ and Cl_int_ values the MS spectra supported by *in silico* metabolism prediction (MetaSite 8.0.1 software) showed significant differences in metabolic pathways between **(*R*)-46** and **(*S*)-46** (Figures S1–S4).

The drug–drug interaction studies were done by measurement of CYP3A4 and 2D6 isoforms activity in the presence of respective enantiomer. The obtained results did not show remarkable effect on the tested CYPs isoforms at the dose of 10 μM of **(*R*)-46** and **(*S*)-46**. Moreover, no differences between enantiomers were reported (Table 4, Figures S5 and S6).

Hepatotoxicity and neurotoxicity *in vitro* assays with use of hepatoma HepG2 and neuroblastoma SH-SY5Y cell lines, respectively, showed a slightly better safety profile of **(*R*)-46**. This enantiomer was less hepatotoxic in comparison to **(*S*)-46**, as statistically significant decrease of cells viability was observed only at the highest dose of **(*R*)-46** (100 μM), whereas for **(*S*)-46** also at 50 μM (Figure 2). Similarly, **(*S*)-46** showed statistically significant effect on neuroblastoma cells at 100 μM but no toxic effect on neurons was determined for (*R*) enantiomer (Figure 3).

**Figure 2:**
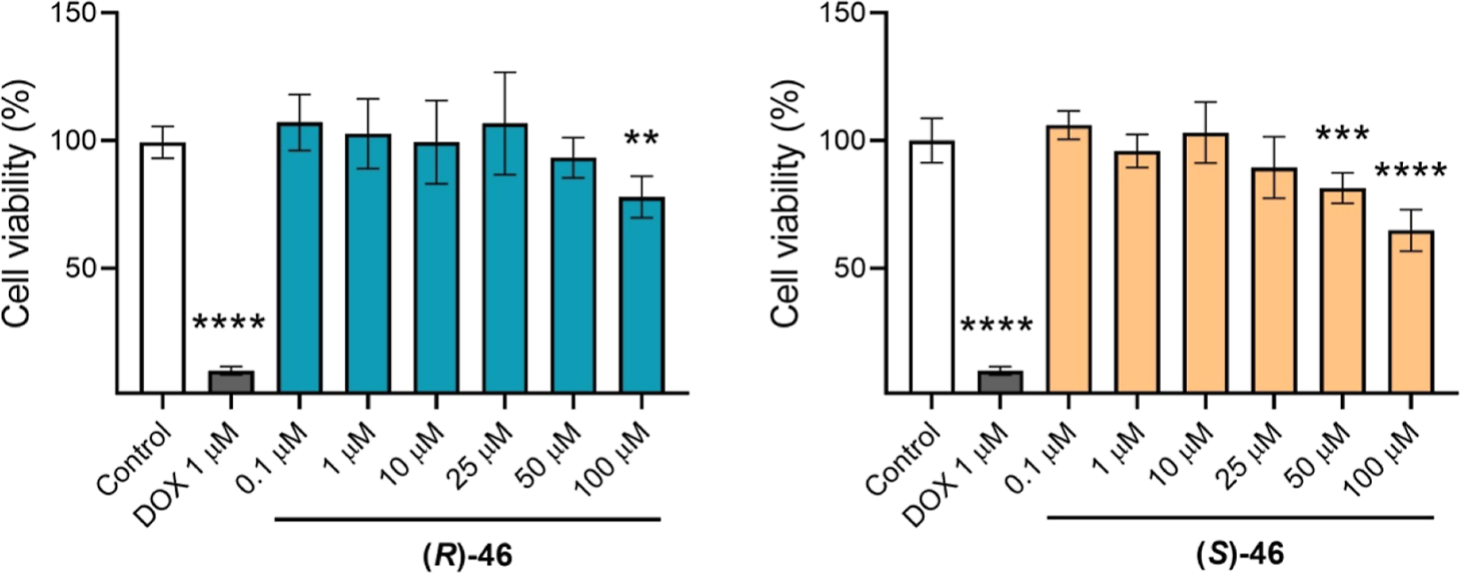
Effect of cytostatic drug doxorubicin (DOX, 1 μM) and **(*R*)-46**, **(*S*)-46** enantiomers on hepatoma HepG2 cell line viability after 72 h of incubation at 37 °C, 5% CO_2_. Data are presented as means ± SD. The statistical significance was evaluated by a one-way ANOVA, followed by Bonferroni’s comparison test; **p < 0.01, ***p < 0.001, ****p < 0.0001 vs control DMSO 1% in growth media (GraphPad Prism 8). Data from two independent experiments.

**Figure 3:**
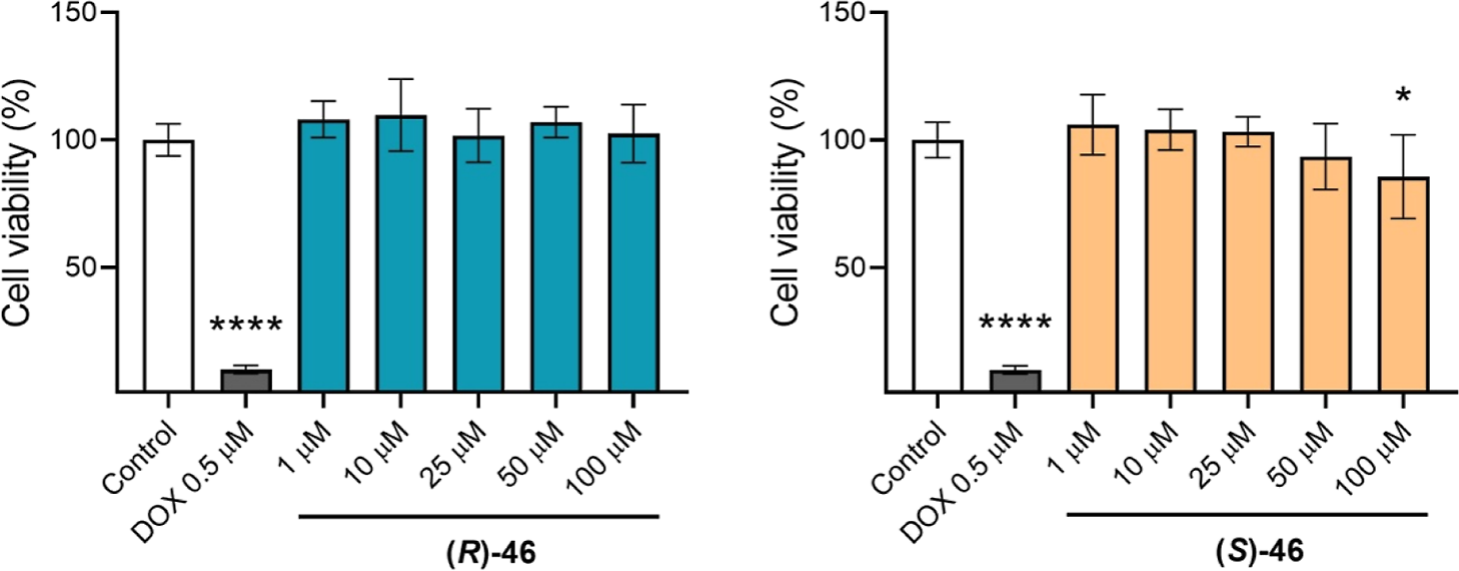
Effect of cytostatic drug doxorubicin (DOX, 0.5 μM) and **(*R*)-46**, **(*S*)-46** enantiomers on SH-SY5Y cell line viability after 48 h of incubation at 37 °C, 5% CO_2_. Data are presented as means ± SD. The statistical significance was evaluated by a one-way ANOVA, followed by Bonferroni’s *post hoc* test; *p < 0.05, ****p < 0.0001 vs control DMSO 1% in growth media (GraphPad Prism 8). Data from two independent experiments.

The more comprehensive hepatotoxicity studies included the potential induction of phospholipidosis and the determination of presence of reactive metabolites (RMs). Phospholipidosis is a disorder in which excessive accumulation of phospholipids occurs inside the hepatocyte in the presence of cationic amphiphilic drugs (CAD). Such accumulation can lead to an inflammatory reaction and histopathological changes in the liver. In the image of the cell, the formation of concentric structures called lamellar bodies by phospholipids is observed. During this assay, HepG2 cells were exposed for 24 h to 10 μM and 50 μM of verapamil (a phospholipidosis inducer), as well as to 50 μM and 100 μM concentrations of **(*R*)-46 and (** ***S*)-46**. The cells were stained next by LYSO-ID Red cytotoxicity kit containing Dual Color Detection Reagent enables for fluorescence visualization of nuclei and lysosome/lysosome-like organelle perturbations in live cells. The collected images did not show the induction of phospholipidosis by **(*R*)-46** and **(*S*)-46** in comparison to the positive control verapamil (see Figures S7 and S8). Moreover, the red fluorescent lysosomal signal was also measured by Leica DMi8 microscopy (Figure 4A) and confirmed the negative results from microscopy imaging.

**Figure 4:**
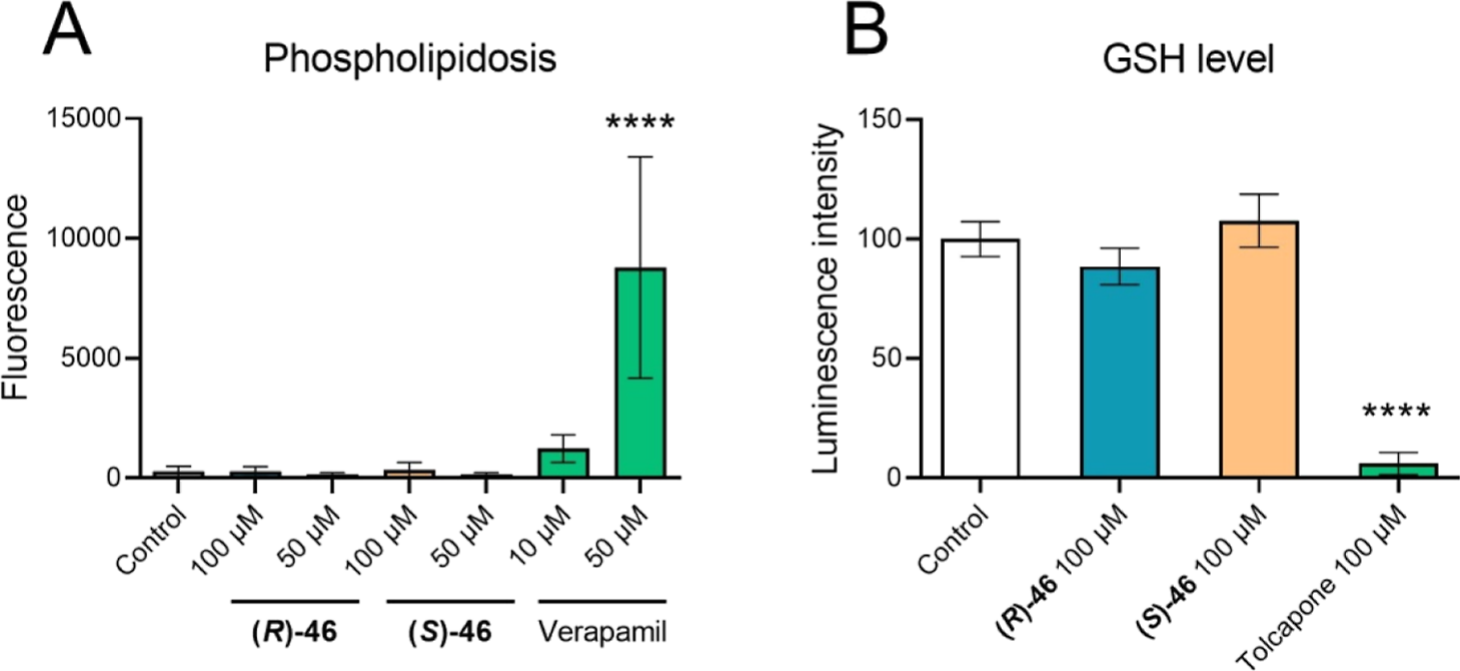
Red fluorescent lysosomal signal intensity measured by Leica DMi8 microscopy. The HepG2 cells were treated by 10 μM and 50 μM of verapamil and by 50 μM and 100 μM **(*R*)-46** and **(*S*)-46** (A). The cells were stained next by LYSO-ID Red cytotoxicity kit containing Dual Color Detection Reagent. The GSH-level in HepG2 cells after 24 h by 100 μM (***R*)-46**, **(*S*)-46** and positive reference tolcapone (B). Data are presented as means ± SD. The statistical significance was evaluated by a one-way ANOVA, followed by Bonferroni’s *post hoc* test; ****p < 0.0001 vs control DMSO 1% in growth media (GraphPad Prism 8).

Drug-induced liver damage (DILI) can be induced in many ways. One of them is formation of RMs. It was reported that RMs of several ASMs i.e., phenytoin or carbamazepine could be involved in the development of idiosyncratic DILI (iDILI).^39^ Thus, within these studies the ASMs candidates **(*R*)-46** and **(*S*)-46** were examined whether they produce RMs in hepatocyte cells or not. The performed test based on the established previously assay with the measurement of glutathione (GSH) level in hepatoma cells.^39^ The GSH-conjugating system plays a crucial role in detoxification of human body including the neutralization of electrophilic RMs pathway in the liver. Therefore, in the presence of RMs a decrease in intracellular GSH can be observed. The GSH-level in HepG2 cells after 24 h of HepG2 cells treatment by 100 μM of **(*R*)-46** and **(*S*)-46** was shown in Figure 4B. Tolcapone, the drug used in the treatment of Parkinson’s disease (PD), was used here as a positive control with confirmed metabolism into RMs responsible for hepatotoxicity in PD patients.^40^ The obtained data did not show the RMs formation by **(*R*)-46** and **(*S*)-46**.

In summary, *in vitro* ADME-Tox evaluation of the enantiomers **(*R*)-46** and **(*S*)-46** revealed generally similar properties, including excellent passive membrane permeability, moderate plasma protein binding, and high metabolic stability in MLMs. Importantly, despite comparable t_0.5_ and Cl_int_ values, *in silico* and MS-based metabolic profiling indicated notable differences in metabolic pathways between the enantiomers. Additionally, neither **(*R*)-46** nor **(*S*)-46** exhibited significant inhibition of CYP3A4 or CYP2D6, suggesting a low risk of drug–drug interactions. However, *in vitro* toxicity studies demonstrated a more favorable safety profile for **(*R*)-46**, which showed reduced hepatotoxicity and neurotoxicity compared to *S*-enantiomer. Moreover, both enantiomers did not show the induction of phospholipidosis and did not cause the RMs formation. These findings, together with previously obtained *in vivo* results, indicate that **(*R*)-46** possesses more advantageous ADME-Tox characteristics, supporting its further development as a promising lead compound.

In order to evaluate and compare the metabolic stability of the newly developed compound, namely **(*R*)-46** with earlier non-imide analogues synthesized by our group, we performed *in vitro* assays using human liver microsomes (HLMs).

All tested compounds exhibited very good metabolic stability (Table 5), especially when compared to the reference drug verapamil, which showed a remaining fraction of only 30.8% under the same conditions. Moreover, the stability of **(*R*)-46** is comparable or better than previously developed analogues, supporting its suitability for further preclinical development.

**Table 5:** Metabolic Stability of **(*R*)-46** and Previously Obtained **(*R*)-KJ-28** and **KJ-79** in the Presence of HLMs

cmpd	% remaining
**(*R*)-46**	85.8
**(*R*)-KJ-28** ^a^	78.5
**KJ-79** ^a^	90.6
**verapamil**	30.8

a Data for compounds: **KJ-79** described as compound 28 from ref;^23^ **(*R*)-KJ-28** described as compound (*R*)-32 from ref.^22^

To further compare the compounds **(*R*)-46, KJ-79**, and **(*R*)-KJ-28**, in Figure 5 we present the results of hepatotoxicity studies performed using HepG2 hepatoma cell lines.

**Figure 5:**
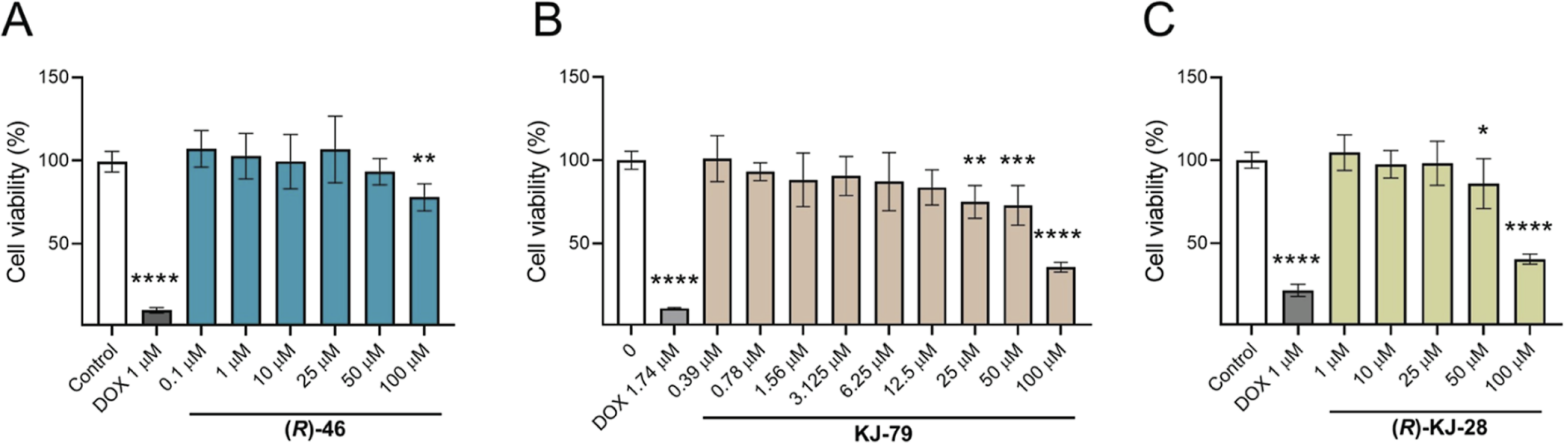
Effect of cytostatic drug doxorubicin (DOX) and **(*R*)-46** (A), **KJ-79** described as compound **28** from ref^23^ (B) and **(*R*)-KJ-28** described as compound (*R*)-**32** from ref^22^ (C) on hepatoma HepG2 cell line viability after 72 h of incubation at 37 °C, 5% CO_2_. Data are presented as means ± SD. The statistical significance was evaluated by a one-way ANOVA, followed by Bonferroni’s comparison test; *p < 0.05, **p < 0.01, ***p < 0.001, ****p < 0.0001 vs control DMSO 1% in growth media (GraphPad Prism 8).

The results clearly show that the lead compound **(*R*)-46** exhibits the lowest risk of hepatotoxicity. Specifically, **(*R*)-46** reduced cell viability only at a concentration of 100 μM, whereas **KJ-79** caused a significant decrease already at 25 μM, and **(** ***R*)-KJ-28** at 50 μM. This finding further differentiates **(*R*)-46** from the other analogues and clearly highlights its overall advantage within this derivative.

In the next part of the *in vitro* characterization of the lead compound **(*R*)-46**, additional experiments were performed to assess its binding to mouse plasma proteins and brain tissue (Table 6). It should be noted that only the unbound fraction of a compound is pharmacologically active, able to cross biological membranes, interact with the molecular target, and exert the therapeutic effect.

**Table 6:** Plasma Protein and Brain Tissue Binding of **(*R*)-46** and Reference Compounds (Mice)^a^

cmpd	% protein bound (plasma)	% tissue bound (brain)
**(*R*)-46**	41.2	50.4
**acebutolol**	0.9	22.6
**warfarin**	85.5	38.3
**sertraline**	96.9	99.5

a Binding studies were performed commercially in Eurofins Laboratories (St Charles, MO, USA).

The results showed that **(*R*)-46** exhibits weak to moderate binding to plasma proteins and brain tissue, leaving a relevant free fraction available for pharmacological activity. In contrast, warfarin and sertraline exhibit very high plasma protein binding levels, which significantly limits their free (unbound) fractions. Additionally, sertraline shows high brain tissue binding, whereas warfarin shows low brain tissue binding. By comparison, acebutolol exhibits very low binding, resulting in a much higher free fraction. Against this background, the binding profile of **(*R*)-46** can be considered balanced and favorable, ensuring adequate levels of free drug without the drawbacks of either very low or very high binding.

### Effect on Seizure Threshold and Neuromuscular Strength in Mice

To better characterize the influence of **(*R*)-** **46** on seizure susceptibility, we tested its effect at three doses on the thresholds for different types of seizures in mice (Figure 6). We found that **(*R*)-** **46** at doses of 10 and 30 mg/kg significantly increased the threshold for tonic hindlimb extension in the maximal electroshock seizure threshold (MEST) test (*p* < 0.01 and *p* < 0.0001, respectively). At the dose of 30 mg/kg, it caused over a ∼2-fold increase in the CC_50_ value and this effect was comparable to the effect of VPA (positive control) tested at 150 mg/kg. **(*R*)-** **46** also dose-dependently increased the threshold for the 6 Hz-induced psychomotor (limbic) seizures (*p* < 0.001 at 20 mg/kg and *p* < 0.0001 at 30 mg/kg). Next, **(*R*)-** **46** at doses of 10, 30, and 50 mg/kg slightly increased the thresholds for the first myoclonic twitch in the *iv*PTZ test (*p* < 0.05 for all doses). It also raised the threshold for generalized clonic seizure with the loss of righting reflex but only at the highest dose tested (*p* < 0.0001). In the *iv*PTZ seizure test in mice, clonic seizure is followed by bilateral forelimb tonic extension that is usually quickly followed by hindlimb tonus and death caused by respiratory arrest. **(*R*)-** **46** at a dose of 10 mg/kg had no effect on tonic fore- and hindlimb extension. When tested at a dose of 30 mg/kg, it did not raise the threshold for forelimb tonus, though it diminished hindlimb tonus (and death) in 6 out of 10 mice. Interestingly, **(*R*)-** **46** at the highest dose tested (50 mg/kg) completely inhibited both fore- and hindlimb tonus in 7 out of 9 mice and hindlimb tonus in 2 mice. This finding suggests that **(*R*)-** **46** may have the potential to inhibit the propagation of seizure activity in the brain.

**Figure 6:**
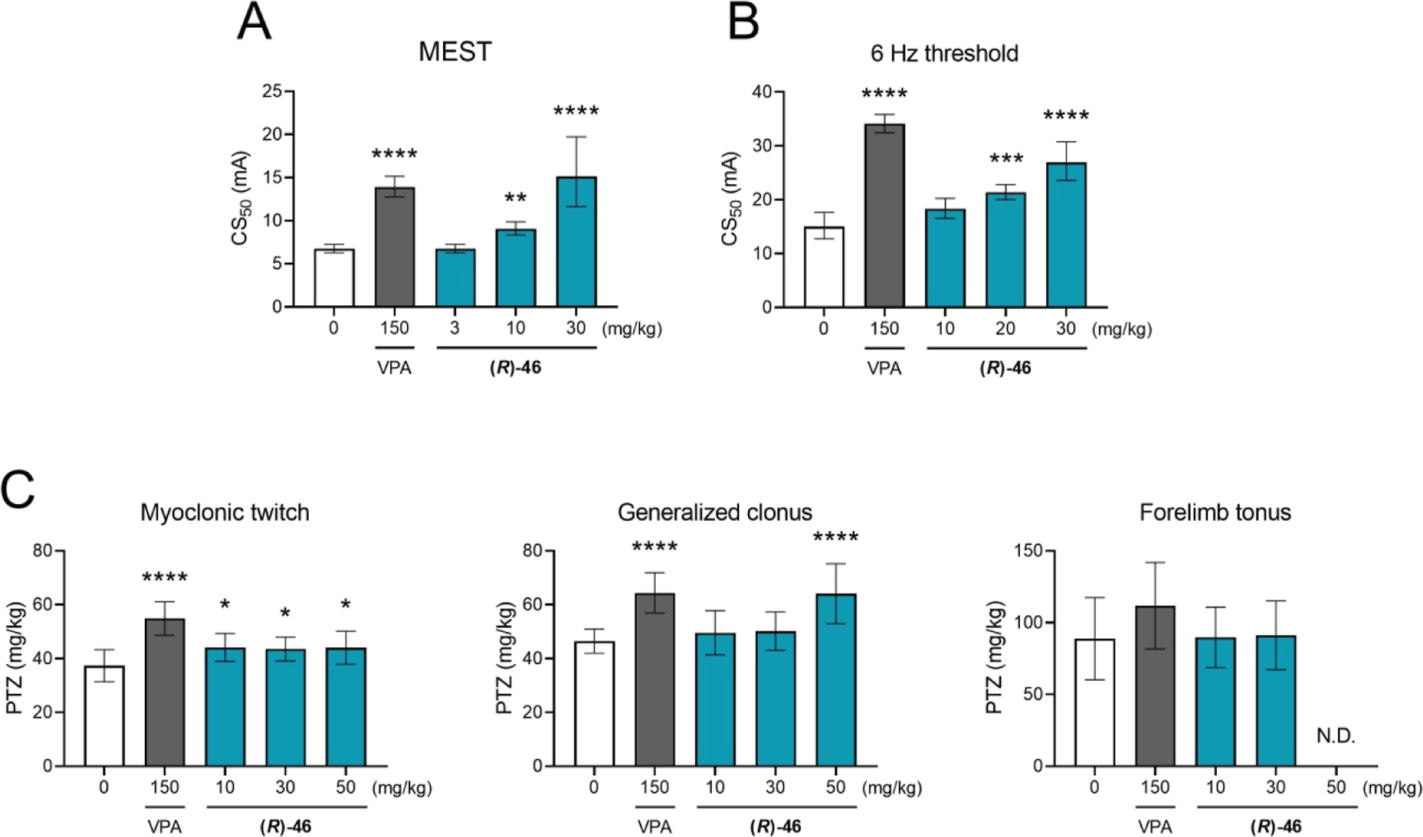
Effect of **(*R*)-46** on seizure thresholds in (A) the MEST test, (B) the 6 Hz-induced seizure test, and (C) the *iv*PTZ test in mice. **(*R*)-46** and VPA were administered *i.p*., 30 and 15 min before the tests, respectively. In panel A and B, data are presented as CC_50_ values (in mA) with upper and lower 95% confidence limits (*n* = 20 animals/group). In panel C, results are presented as means ± SD in mg/kg of PTZ necessary to evoke each of the three studied end points (*n* = 10–13 animals/group). The statistical significance was evaluated by a one-way ANOVA followed by Dunnett’s *post hoc* test; *p < 0.05, **p < 0.01, ***p < 0.01, ****p < 0.0001 vs control group (GraphPad Prism 8). N.D.—not determined due to the lack of forelimb tonus at highest dose tested.

For comparison, compound **(*R*)-46** exhibited a similar effect on the threshold for myoclonic and clonic seizures as compounds **(*R*)-KJ-28** (see compound (*R*)-32)^22^ and **KJ-79** (see compound 28)^23^ (all tested at 50 mg/kg, *i.p.*). Additionally, **(*R*)-46** had a comparable effect to compound **KJ-79** on forelimb tonus—both compounds completely abolished the occurrence of tonic extension of the forelimbs in the majority of animals. In contrast, **(*R*)-KJ-28** had no effect on this type of seizure.

The grip strength test and chimney test were performed just before all seizure tests to evaluate potential adverse effects of **(*R*)-46**. At the range of doses tested (3–50 mg/kg), **(*R*)-46** did not significantly affect neuromuscular strength or motor coordination in mice (Table S3).

### PTZ-Induced Kindling Model in Mice

To further evaluate the antiseizure potential of **(*R*)-46**, we examined its effect after chronic treatment on the development of the PTZ-induced kindling in mice. The PTZ-induced kindling model is a well-established method for inducing a chronic increase in seizure susceptibility. Repeated administration of PTZ at a subconvulsive dose (40 mg/kg, three times per week) increased the average seizure severity score in the control group from 1.0 ± 0.0 (after the first injection) to 3.9 ± 1.7 (after the final injection). **(*R*)-46** administered repeatedly at a dose of 30 mg/kg did not significantly impact kindling development. When injected at a dose of 50 mg/kg, it slightly suppressed kindling progression leading to a significant reduction in the mean seizure severity score compared to the PTZ control group (*p* < 0.05 at the last PTZ injection). Specifically, the mean seizure severity score in the group of animals treated with **(*R*)-46** at 50 mg/kg after the last PTZ injection was 2.0 ± 1.4. For comparison, the mean seizure severity in VPA-treated group (150 mg/kg) was 1.7 ± 1.4 (Figure 7).

**Figure 7:**
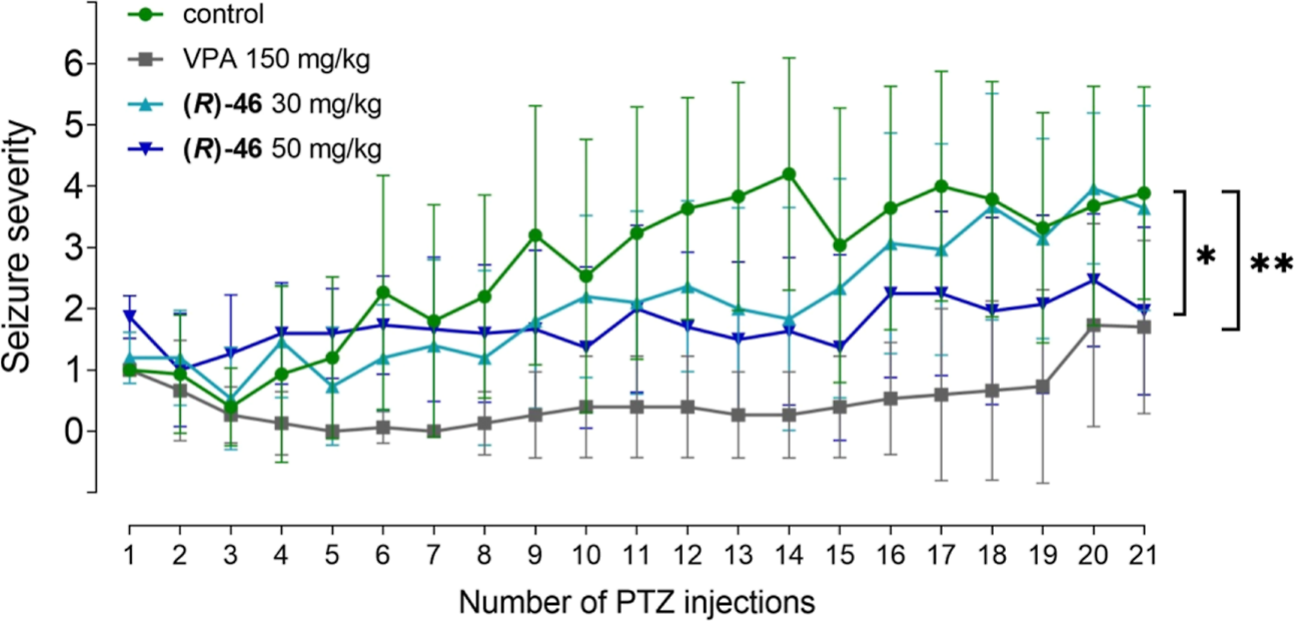
Effect of repeated treatment with **(*R*)-46** on the progression of PTZ kindling in mice. **(*R*)-46**, VPA or vehicle were administered *i.p.* every 24 h. Kindling was induced by administering PTZ at a subconvulsive dose of 40 mg/kg (*i.p.*) three times a week, 30 min after the administration of **(*R*)-46**, VPA, or vehicle. Data are presented as average seizure scores (±SD) assessed according to the Racine scale (*n* = 13–15 animals). The statistical significance was evaluated by a mixed effects model for repeated measures followed by Tukey’s *post hoc* test: *p < 0.05, **p < 0.01 vs control group (GraphPad Prism 8).

When compared with compounds **(*R*)-KJ-28** (see compound (*R*)-**32**)^22^ and **KJ-79** (see compound **28**),^23^ **(*R*)-46** demonstrated the most pronounced activity in the PTZ kindling model, significantly attenuating kindling progression at 50 mg/kg. **(*R*)-KJ-28** also exhibited activity in this model, but only at a higher dose of 80 mg/kg, suggesting a weaker potency compared to **(*R*)-46**. In contrast, compound **KJ-79** had no effect on kindling progression at any of the tested doses, indicating a lack of efficacy in this model; however, this might be due to relatively low doses used (i.e., 10–40 mg/kg).

### Effect on Cytokines and Amino Acids Level in the Hippocampus of PTZ-Kindled Mice

After kindling completion, we determined the levels of inflammatory markers (TNF-α, IFN-γ, IL-1β, IL-6, IL-10, and IL-17A) and amino acids, including GABA and glutamate, in the hippocampus to provide additional insights into the pharmacological effects of **(*R*)-46**. Kindled seizures caused ∼30% increase in the concentrations of IL-1β, IL-10, and IL-17A as compared to the nonkindled control mice. However, neither **(*R*)-46** nor VPA reversed these changes (Figure S9).

Quantitative analysis of amino acid concentrations in hippocampal extracts revealed no statistically significant differences among the experimental groups (Table S4). Glutamate was the most abundant amino acid detected. Glutamine and GABA also showed high abundance across all groups. A small, though not statistically significant, decrease in the concentrations of glutamate in the hippocampus of VPA- and **(*R*)-46**-treated kindled mice was observed. Minor amino acids such as methionine, isoleucine, and phenylalanine were consistently present at low concentrations (typically <0.1 nmol/mg), with minimal intergroup variability. Similarly, branched-chain amino acids (leucine, isoleucine, valine) exhibited stable profiles.

### Antinociceptive Activity

LCS, a compound originally developed as an antiepileptic drug, exhibits promising analgesic properties in preclinical models. Its primary mechanism of action involves the selective enhancement of slow inactivation of voltage-gated sodium channels, particularly the Nav1.3, Nav1.7, and Nav1.8 subtypes, without affecting fast inactivation. LCS may also modulate the activity of CRMP2 (Collapsin Response Mediator Protein 2), which regulates the expression of Nav1.7 channels, and influence the expression of pro-inflammatory cytokines (TNF-α, IL-1β). These effects reduce neuronal hyperexcitability and suggest its potential use in the treatment of chronic inflammatory and neuropathic pain.^41^

In animal models, LCS has demonstrated efficacy in reducing neuropathic pain induced by nerve injury, chemotherapy, as well as in models of inflammatory pain. Notably, it reduced both mechanical and thermal hypersensitivity, often showing effects comparable to or better than pregabalin, with a lower sedative profile.^42,43^

Although LCS is not yet approved for pain treatment in clinical practice, the available data indicate its high efficacy and good tolerability. Therefore, LCS represents a promising therapeutic alternative in neuropathic pain (Phase III trials), particularly in patients unresponsive to standard treatments. These data encouraged us to assess the potential of the LCS-based hybrid compound **(*R*)-46** not only as an ASM, but also as a novel analgesic, offering broader therapeutic utility.

As illustrated in Figure 8A, *i.p*. administration of **(*R*)-46** led to a marked reduction in pain-related behaviors during both phases of the formalin test, an established model used to differentiate between acute nociceptive pain (phase I) and prolonged inflammatory pain (phase II).^44^ The compound displayed dose-dependent antinociceptive effects, with ED_50_ values of 46.2 mg/kg for phase I and 21.7 mg/kg for phase II. Furthermore, **(*R*)-46** effectively suppressed the capsaicin-evoked nociceptive response (Figure 8B), yielding an ED_50_ of 34.1 mg/kg. This result suggests that **(*R*)-46** may interfere with neurogenic pain mechanisms, likely by modulating the release of pro-nociceptive neuropeptides such as substance P and calcitonin gene-related peptide (CGRP), which contribute to peripheral sensitization and neurogenic inflammation.^45^

**Figure 8:**
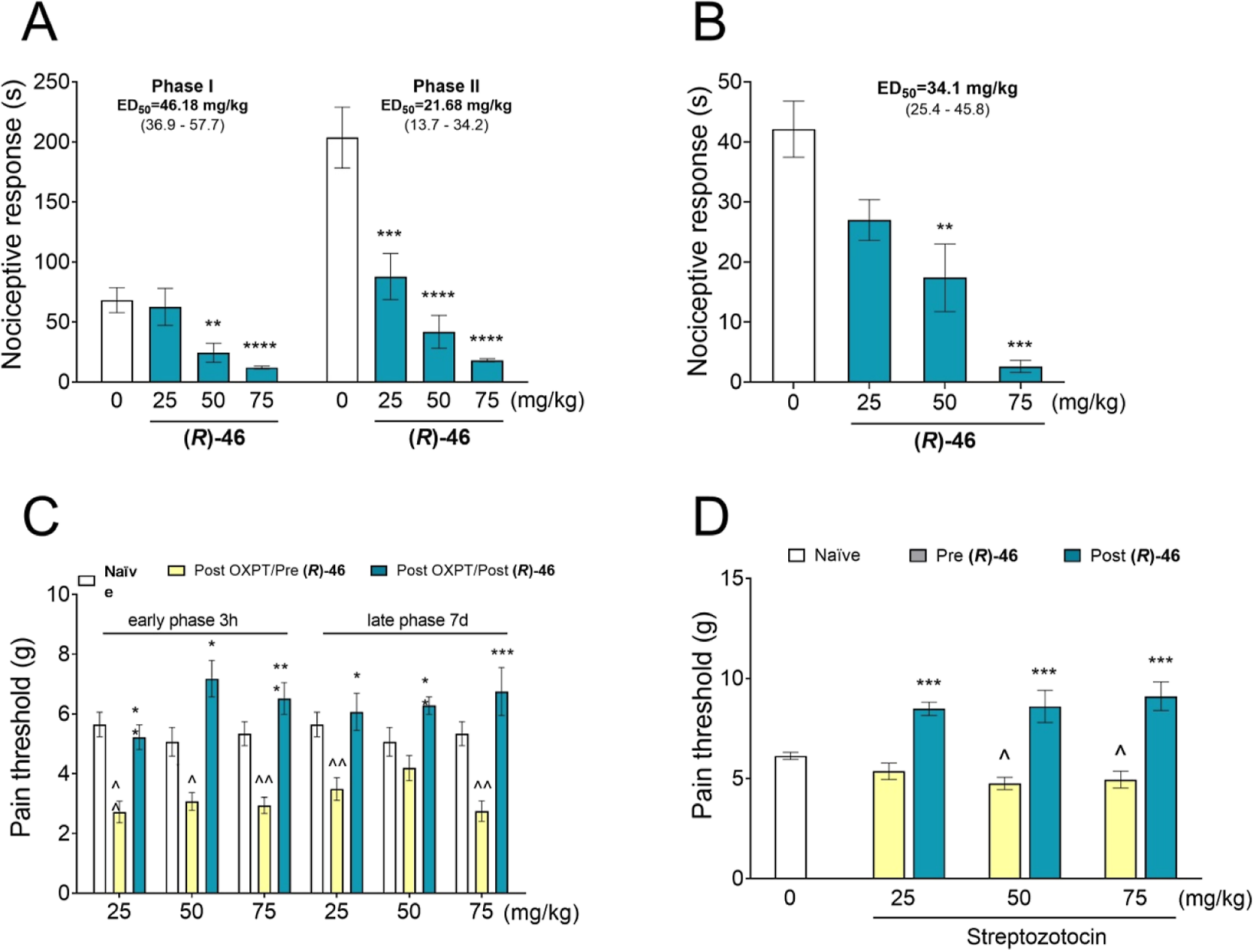
Antinociceptive activity of (***R*)-46** in the animal models of pain (A–D). The compound was tested in the formalin test (A), capsaicin test (B), OXPT-induced (C) and STZ-induced (D) neuropathic pain models, where mechanical allodynia was measured by the electronic von Frey test (C,D). The results are presented as bar plots showing the mean ± SEM. The statistical significance was evaluated by a one-way ANOVA followed by Dunnett’s *post hoc* test (A,B); **p < 0.01, ***p < 0.001, ****p < 0.0001 when compared to vehicle-treated animals; two-way ANOVA followed by Tukey’s *post hoc* test (C,D), *p < 0.05, **p < 0.01, ***p < 0.001, when compared to OXPT- or STZ-treated animals; ^*p*< 0.05, ^*p* < 0.01, when compared to naïve animals, *n* = 8–10 mice per group (GraphPad Prism 8).

In addition, **(*R*)-46** showed robust efficacy in two distinct models of neuropathic pain: one induced by oxaliplatin (OXPT) (Figure 8C), mimicking chemotherapy-induced peripheral neuropathy (CIPN),^46^ and the other by streptozotocin (STZ) (Figure 8D), representing diabetic neuropathy.^47^ Both OXPT and STZ produced mechanical hypersensitivity in naïve animals, indicative of hyperalgesia and allodynia. Treatment with **(*R*)-46** not only reversed these pain symptoms but also elevated the mechanical threshold above baseline levels observed prior to neuropathy induction. These findings underscore the compound’s strong therapeutic potential for managing neuropathic pain across different etiologies.

As shown in Figure 9, compound **(*R*)-46** produced a statistically significant reduction in spontaneous locomotor activity, but this effect was observed only at the highest tested dose of 75 mg/kg. Notably, **(*R*)-46** demonstrated robust analgesic efficacy at lower doses (25 and 50 mg/kg), which did not impair locomotor performance. These findings suggest that the observed antinociceptive effects are not attributable to sedation or motor impairment, thereby supporting the specificity of **(*R*)-46**’s analgesic action and minimizing the likelihood of false-positive results due to nonspecific behavioral suppression.

**Figure 9:**
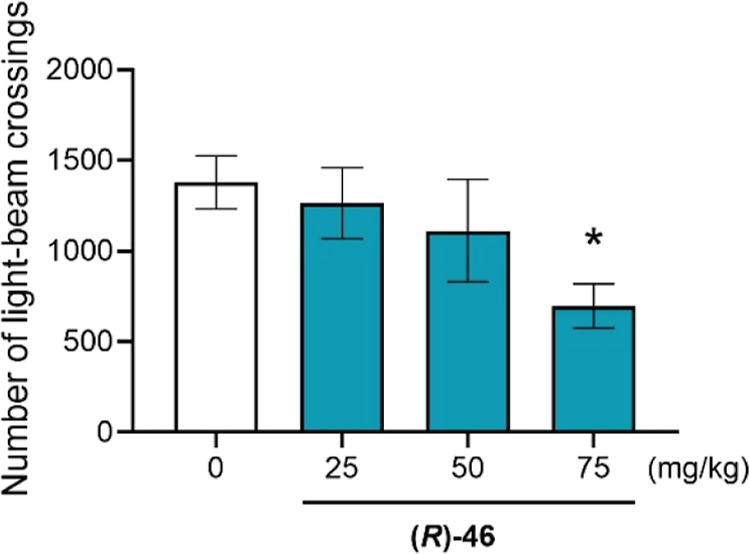
Influence of **(*R*)-46** on mice spontaneous locomotor activity. The results are presented as bar plots showing the mean ± SEM. The statistical significance was evaluated by a one-way ANOVA followed by Dunnett’s *post hoc* test; *p < 0.05 (GraphPad Prism 8).

In summary, **(*R*)-46** exhibited a broad analgesic spectrum, effectively attenuating pain across multiple etiologies and demonstrating a profile comparable to that of previously characterized compounds such as **(*R*)-KJ-28** (see compound (*R*)-32)^22^ or **KJ-79** (see compound 28).^23^ Importantly, **(*R*)-46** showed superior potency and efficacy across all applied models and methods when compared to **(*R*)-KJ-28**. Furthermore, in both neurogenic and neuropathic pain paradigms, **(*R*)-46** produced a significantly higher efficacy (maximal possible effect) than compound **KJ-79**, underscoring its enhanced potential in managing complex pain conditions.

### Pharmacokinetic Studies

The data presented in Figures S10, S11 and Table 7 indicate that **(*R*)-46** was very rapidly absorbed after extravascular administration to mice. The time to reach maximum concentration (*t*_max_) both after *i.p*. and *p.o.* dosing was 5 min that was the first sampling time point. The elimination half-lives were similar in serum and brain after both *i.p.* doses and they were relatively long ranging from 55.86 to 75.02 min. A more than proportional increase in AUC was observed with increasing *i.p.* doses both in serum and brain tissue. The 2-fold greater *i.p.* dose of **(*R*)-46** resulted in a 2.57- and 3.32-fold increases in AUC estimated in serum and brain, respectively; indicating the saturation of some processes involved in compound pharmacokinetics in these dose levels. Consequently, CL/F (calculated as Dose/AUC) was about 20% lower after the higher dose. Similarly, the volume of distribution (*V*_*z*_/F) was 25% lower after administration of the higher *i.p.* dose. Administration of the tested compound orally led to lower values of *C*_max_ and AUC both in serum and brain in comparison to those estimated after *i.p.* administration of the same dose. The maximal concentrations in these matrices were 2–3 times lower than those observed after *i.p.* dosing. This difference was not that evident in the case of AUC. Following *i.v.* administration, the values of t_0.5λz_ were identical in serum and brain and they were about 26 min. The volume of distribution was 1.59 L/kg that was much lower than that estimated after extravascular dosing but still larger than mouse body water. Compound **(*R*)-46** quite well penetrated to brain that is the site of action for this compound. The brain-to-serum AUC ratio was close to 1 for both *i.p.* doses and about 0.7 for *p.o.* and *i.v* doses. Absolute bioavailability values of **(*R*)-46** calculated after 25 mg/kg *p.o.*, 25 mg/kg *i.p.,* and 50 mg/kg *i.p.* were 0.78, 0.92, and 1.15, respectively. Pharmacokinetic parameters estimated using a two-compartment pharmacokinetic model after *i.v.* administration were as follows: elimination rate constant (*k*_10_) was 0.055 1/min, elimination half-life was 12.63 min, clearance was 0.052 L/min/kg, volume of central compartment was 0.95 L/kg, and steady-state volume of distribution was 2.65 L/kg.

**Table 7:** Pharmacokinetic Parameters of **(*R*)-46** Estimated in Serum and Brain Following *i.v., p.o.,* and *i.p.* Administration of This Compound to Mice

estimate								
pharmacokinetic parameter (unit)^a^	serum	brain	serum	brain	serum	brain	serum	brain
	10 mg/kg *i.v.*		25 mg/kg *p.o.*		25 mg/kg *i.p.*		50 mg/kg *i.p.*	
*t*_max_ (min)	0	5	5	5	5	5	5	5
*C*_max_/*C*_0_ (ng/mL(g))	7235.16	6716.67	7026.67	5625.83	19783.33	17441.67	30466.67	33433.33
λ_*z*_ [1/min]	0.026	0.026	0.0068	0.0074	0.011	0.009	0.012	0.012
*t*_0.5λz_ [min]	26.62	26.41	102.28	93.31	60.45	75.02	57.83	55.86
AUC_0‑*t*_ [ng·min/mL(g)]	242273.3	169977.7	460745.3	344922.3	554665.8	546240.8	1424004.1	1823166.7
AUC_0‑∞_ [ng·min/mL(g)]	242341.3	169977.7	474355.0	353003.8	554821.0	546628.0	1424674.5	1823614.1
*V*_z_/F [L/kg]	1.59	–	7.78	–	3.93	–	2.93	–
CL/F [L/min/kg]	0.041	–	0.05	–	0.045	–	0.035	–
MRT [min]	21.34	20.74	107.50	87.39	26.78	27.58	42.36	40.18

a Pharmacokinetic parameters: *t*_max_ time to reach *C*_max_; *C*_max_ maximum serum/brain concentration; λ_*z*_ terminal slope; *t*_0.5 λz_ terminal half-life; AUC_0‑*t*_ area under the curve from time zero to the last measurable time point; AUC_0‑∞_ area under the curve from time zero to infinity; *V*_*z*_/F volume of distribution; CL/F clearance; MRT mean residence time.

### *In Vitro* Binding and Functional Assays

Due to the structural similarity of compound **(*R*)-** **46** to previously reported chemical prototypes as well as LCS, its mechanism of action was explored through a series of *in vitro* binding and functional assays.^22,23,25,48^ These included evaluation of its interaction with voltage-gated sodium channels (site 2) and Cav1.2 calcium channels, as well as functional studies on TRPM8, TRPA1, TRPV1, and 5-HT_2C_ receptors. Notably, altered sodium or calcium conductance is frequently linked to the pathophysiology of epilepsy and neuropathic pain^49–53^ and these ion channels represent well-recognized molecular targets for various structurally distinct ASMs, including LCS, ethosuximide, carbamazepine, and lamotrigine.^54^ With regard to the compound’s cardiac safety, the influence of **(*R*)-** **46** on the hERG potassium channel, one of the most critical off-targets associated with drug-induced proarrhythmic risk, was also evaluated (Table 8).

**Table 8:** *In Vitro* Binding and Functional Assays for **(*R*)-46**

binding studies	source	% inhibition of control specific binding (concentration [μM])^a^
Na^+^ channel (site 2)	rat cerebral cortex	73.6 (10)
potassium channel (hERG)	human recombinant HEK-293 cell	7.7 (10)
Cav1.2 (L-type) human calcium ion channel binding (dihydropyridine site)	human recombinant (CHO cells)	–6.8 (100)

functional studies	source	% inhibition of control agonist response (concentration [μM])^a^
TRPM8 (antagonist effect)	human recombinant HEK-293 cell	–16.5 (10)
TRPA1 transient potential ion channel cell based antagonist calcium flux assay	human recombinant HEK-293 cell	–13.3 (10)
TRPV1 (antagonist effect)	human recombinant CHO cells	–5.1 (10)
5-HT_2C_ (agonist effect)	human recombinant HEK-293 cell	77.6 (10)

a Results showing activity higher than 50% are considered to represent significant effects of the test compounds; results showing an inhibition between 25% and 50% are indicative of moderate effect; results showing an inhibition lower than 25% are not considered significant and mostly attributable to variability of the sign al around the control level. Binding and functional studies were performed commercially in Eurofins Laboratories (Poitiers, France).

As shown in Table 8, **(*R*)-46** demonstrated strong effect of sodium channels and no effect on Cav_1.2_ calcium channels. These *in vitro* findings indicate that modulation of sodium currents may play a key role in the broad-spectrum antiseizure and antinociceptive effects observed for **(*R*)-46** *in vivo*. Moreover, compound **(*R*)-46** showed no affinity for the hERG channel (Table 8). This lack of interaction with these key cardiac ion channels is favorable from a cardiac safety perspective and supports its assessment within the comprehensive *in vitro* proarrhythmia assay (CiPA) panel.^55^

Functional assays further revealed that **(*R*)-46** did not modulate TRPM8, TRPA1, and TRPV1 channel responses, distinguishing it from previously reported phenylglycine derivatives.^22^ Interestingly, an agonist effect was observed at the 5-HT_2C_ receptor (EC_50_ = 2.9 μM**)**, a target recently implicated in the antiseizure activity of serotonergic drugs such as lorcaserin receptor (EC_50_ = 9 nM**)**.^56^ This finding suggests a serotonergic component may be also responsible for the antiseizure profile of **(** ***R*)-46**.

Voltage-gated sodium channels (Nav) represent critical molecular targets in epilepsy and pain, with distinct subtypes contributing to pathophysiology. Nav1.1 is predominantly expressed in inhibitory GABAergic interneurons, and its dysfunction has been strongly linked to genetic epilepsies such as Dravet syndrome. Nav1.2 is widely distributed in excitatory neurons of the central nervous system and plays an important role in seizure initiation and propagation. Nav1.3, while normally expressed at low levels in the adult brain, is markedly upregulated following neuronal injury or in chronic epilepsy, where it may contribute to aberrant excitability. Nav1.6 is essential for high-frequency firing and sustained action potential conduction; excessive activity of this subtype has been associated with epileptogenesis as well as pain-related diseases. Finally, Nav1.7 is primarily expressed in peripheral sensory neurons and is critically involved in pain signaling.^54,57,58^ The binding assays revealed an interaction of **(*R*)-46** with site 2 of the sodium channel (Table 8). Considering the structural similarity of the synthesized compounds to LCS and its known mechanism of action, enhancement of slow inactivation of voltage-gated sodium channels without affecting fast inactivation, we conducted a more detailed functional characterization. Specifically, whole-cell patch-clamp studies were performed using N1E-115 neuroblastoma cells, which endogenously express multiple sodium channel subtypes relevant to epilepsy and pain (including Nav1.1, Nav1.2, Nav1.3, Nav1.6, and Nav1.7). These experiments assessed the effects of **(*R*)-46** on sodium channels in resting, fast-inactivated, and slow-inactivated states. The results are summarized in Tables 9 and 10, Figures S12 and S13.

**Table 9:** Effect of the **(** ***R*)-46** (250 μM) and Carbamazepine (250 μM) on the Remaining Current Amplitude in Voltage-Gated Sodium Channels Expressed in N1E-115 Neuroblastoma Cells in Use Dependence Protocol

application	remaining current amplitude (mean ± SD)	
	first pulse (%)	last pulse (%)
**control**	100.92 ± 0.75	105.44 ± 0.78
**(*R*)-46**	95.60 ± 1.51	91.72 ± 0.54
**carbamazepine**	91.07 ± 0.77	91.21 ± 5.39

**Table 10:** Effect of the **(*R*)-46** (250 μM) and LCS (250 μM) on the Remaining Current Amplitude in Voltage-Gated Sodium Channels Expressed in N1E-115 Neuroblastoma Cells in State Dependence Protocol

application	parameter	remaining current amplitude (mean ± SD) (%)
**control**	resting state	97.47 ± 2.75
	fast inactivated state	92.69 ± 11.52
	slow inactivated state	91.58 ± 6.05
**(*R*)-46**	resting state	84.96 ± 2.22
	fast inactivated state	50.25 ± 4.43
	slow inactivated state	30.45 ± 9.85
**LCS**	resting state	93.89 ± 2.78
	fast inactivated state	73.97 ± 1.57
	slow inactivated state	53.07 ± 1.69

Compound **(** ***R*)-46** at a concentration of 250 μM caused a slight reduction in sodium current amplitude from the first pulse and the last pulse. In comparison, carbamazepine at the same concentration also exhibited minimal changes across the first and last pulse indicating no effect on the sodium current in this protocol carbamazepine was used as a reference drug because it is a well-established sodium channel blocker with known use-dependent inhibition; however, at the tested concentration of 250 μM, it did not produce a significant effect either.

At a concentration of 250 μM, **(*R*)-46** reduced sodium current amplitude in both fast and slow inactivated states. In contrast, LCS at the same concentration produced a significant reduction in peak sodium current only during the slow inactivation state. This differential effect suggests that **(*R*)-46** exhibits a broader inhibitory profile compared to LCS.

Given that **(** ***R*)-46** showed sodium channel binding affinity in binding assays and modulatory effects in electrophysiological assays, an important consideration was its selectivity profile, particularly with respect to Nav1.5, the predominant cardiac sodium channel. Modulation of Nav1.5 is associated with severe cardiotoxic effects and represents a critical off-target liability for CNS-active compounds.^55^ ,^59^ To address this, we performed whole-cell patch-clamp experiments to assess the effect of **(*R*)-46** on Nav1.5 currents (Table 11 and Figure S14).

**Table 11:** Effect of the **(*R*)-46** (250 μM), LCS (250 μM) and Propafenone (10 μM), as Positive Control on the Remaining Current Amplitude in Nav1.5 Sodium Channels Stably Expressed in CHO Cells

application	parameter	remaining current amplitude (mean ± SD) (%)
**control**	resting state	102.03 ± 3.62
	fast inactivated state	85.35 ± 8.99
	slow inactivated state	100.70 ± 3.76
**(*R*)-46**	resting state	89.58 ± 0.05
	fast inactivated state	69.04 ± 7.39
	slow inactivated state	69.15 ± 3.14
**LCS**	resting state	93.74 ± 2.33
	fast inactivated state	77.69 ± 1.15
	slow inactivated state	73.97 ± 0.07
**propafenone**	resting state	52.16 ± 2.37
	fast inactivated state	18.17 ± 0.09
	slow inactivated state	6.35 ± 2.20

At a concentration of 250 μM, **(*R*)-46** produced only a minimal reduction in sodium current amplitude, indicating negligible activity at this subtype. Notably, the observed effect was comparable to that of LCS (250 μM), a clinically used ASM with a favorable cardiac safety profile. In contrast, propafenone (antiarrhythmic drug, positive control) markedly reduced Nav1.5 currents at substantially lower concentrations (10 μM), particularly in the fast and slow inactivated states. These findings suggest that **(*R*)-46** exhibits a favorable selectivity profile toward neuronal sodium channels while avoiding significant interaction with cardiac isoforms, thereby reducing the risk of adverse cardiovascular effects.

In summary, *in vitro* binding and functional studies suggest that the interaction of **(*R*)-46** with sodium channels and 5-HT_2C_ receptor may contribute to its broad-spectrum antiseizure and antinociceptive activities. This mechanism of action on clearly distinguishes the compound from those obtained previously, such as **KJ-79** and **(*R*)-KJ-28**. **KJ-79** was found to inhibit fast sodium currents, similar to **(*R*)-KJ-28**; however, the latter also acts as a TRPV1 channel antagonist, clearly demonstrating that even closely related molecules can differ mechanistically. In contrast, our lead compound **(*R*)-46** not only inhibits fast sodium currents but also acts as an agonist of the 5-HT_2C_ receptor, a property that may contribute to its enhanced antiseizure efficacy. This dual mechanism appears particularly promising for the treatment of severe and drug-resistant forms of epilepsy, including Lennox–Gastaut syndrome, Dravet syndrome, and other focal epilepsies.^60–63^

## Conclusions

In the present study, using multitarget-oriented design concept, we synthesized 17 racemic compounds and 6 enantiomers, the latter derived from the 3 most active racemates. These novel compounds demonstrated robust antiseizure properties. They were effective in standard *in vivo* seizure models, including the maximal electroshock seizure (MES) test, the psychomotor 6 Hz (32 mA) model, and notably the 6 Hz (44 mA) model of drug-resistant epilepsy (DRE). The most active compound, **(*R*)-46**, also increased seizure thresholds in the MES, 6 Hz, and *iv*PTZ models without affecting grip strength. Chronic administration of **(*R*)-46** at 50 mg/kg moderately suppressed PTZ-induced kindling, resulting in a significant reduction in seizure severity compared to controls. Following kindling, **(*R*)-46** did not significantly alter hippocampal levels of inflammatory markers or amino acids, although a minor, nonsignificant decrease in glutamate concentration was observed. In pain models, **(*R*)-46** exhibited strong efficacy in formalin-induced tonic pain, capsaicin-induced pain, OXPT-induced peripheral neuropathy, and STZ-induced diabetic neuropathy. To strengthen the rationale for further development of our lead compound, we conducted explicit head-to-head comparisons between **(*R*)-46** and previously characterized analogues, including **KA-93**, **(*R*)-KJ-28**, **KJ-79**, or its *R*-enantiomer, namely **(*R*)-KJ-79**. These comparisons, summarized in Table 3 and discussed in detail within the sections on antiseizure and antinociceptive activity, consistently demonstrate the superior efficacy and potency of **(*R*)-46** particularly in pharmacoresistant seizure models such as 6 Hz (44 mA), PTZ kindling and pain models.

Pharmacokinetic and *in vitro* ADME-Tox profiling confirmed favorable drug-like properties, supporting its potential for further preclinical development. **(** ***R*)-46** exhibits a unique dual mechanism of action, combining 5-HT_2_C receptor agonism with voltage-gated sodium channel blockade, as demonstrated by patch-clamp studies. Importantly, **(*R*)-46** showed no significant interaction with hERG, Cav1.2, or Nav1.5 channels, indicating a favorable cardiac safety profile However, further electrophysiological investigations targeting specific Nav subtypes are warranted.

Overall, the data obtained strongly suggest that **(*R*)-46** stands out as the most promising compound in the entire series. It exhibits the highest anticonvulsant and antinociceptive potency, operates through a distinct mechanism of action, and shows a reduced likelihood of hepatotoxic effects. These combined properties make **(*R*)-46** a compelling candidate for further preclinical development aimed at epilepsy and neuropathic pain therapy.

## Materials and Methods

### Chemistry

#### General Information

All chemicals and solvents were purchased from commercial suppliers and were used without further purification. Melting points (mp.) were determined in open capillaries on a Büchi 353 melting point apparatus (Büchi Labortechnik, Flawil, Switzerland). TLC and the gradient UPLC chromatography were used to assess the purity and homogeneity of the compounds. TLC was carried out on silica gel 60 F_254_ precoated aluminum sheets (Macherey-Nagel, Düren, Germany), using the following developing systems: S_1_–DCM/MeOH (9:0.3; *v/v*), S_2_–DCM/MeOH (9:0.5; *v/v*). Spots detection: UV light (λ = 254 nm). The UPLC and mass spectra (LC–MS) were obtained on Waters ACQUITY TQD system (Waters, Milford, CT, USA) with the MS-TQ detector and UV–vis-DAD eλ detector. The ACQUITY UPLC BEH C18, 1.7 μm (2.1 × 100 mm) column was used with the VanGuard Acquity UPLC BEH C18, 1.7 μm (2.1 × 5 mm) (Waters, Milford, CT, USA). Standard solutions (1 mg/mL) of each compound were prepared in analytical grade MeCN/water mixture (1:1; *v/v*). Conditions applied were as follows: eluent A (water/0.1% HCOOH), eluent B (MeCN/0.1% HCOOH), a flow rate of 0.3 mL/min, a gradient of 5–100% B over 10 min, and an injection volume of 10 μL. The UPLC analyses and high-resolution mass spectra (LC-HRMS) were obtained on Waters ACQUITY I-Class PLUS SYNAPT XS High Resolution Mass Spectrometer (Waters, Milford, CT, USA) with the MS-Q-TOF detector and UV–vis-DAD eλ detector. The ACQUITY UPLC CSH C18, 1.7 μm (2.1 × 100 mm) column was used with the VanGuard Acquity UPLC CSH C18, 1.7 μm (2.1 × 5 mm) (Waters, Milford, CT, USA). Standard solution (1 mg/mL) of compound was prepared in analytical grade MeCN/water mixture (1:1; *v/v*). Conditions applied were as follows: eluent A (water/0.1% HCOOH), eluent B (MeCN/0.1% HCOOH), a flow rate of 0.3 mL/min, a gradient of 5–100% B over 13 min, and an injection volume of 1 μL. The UPLC retention times (*t*_R_) are given in minutes. The purity of target compounds determined by the use of the chromatographic UPLC method was ≥98%. Preparative column chromatography was performed using silica gel 60 (particle size 0.063–0.200; 70–230 Mesh ATM) purchased from Merck (Darmstadt, Germany). ^1^H NMR and ^13^C NMR spectra were obtained in a JEOL-500 spectrometer (JEOL USA, Inc. MA, USA) in CDCl_3_ operating at 500 MHz (^1^H NMR) and 126 MHz (^13^C NMR). Chemical shifts are reported in δ values (ppm) relative to TMS δ = 0 (^1^H), as an internal standard. The *J* values are expressed in Hertz (Hz). Signal multiplicities are represented by the following abbreviations: s (sdtinglet), br s (broad singlet), d (doublet), br d (broad doublet), dd (double doublet), ddd (double double doublet), t (triplet), td (triple doublet), q (quartet), m (multiplet). Chiral SFC assays were conducted on Agilent 1260 Infinity II Analytical SFC. The Trefoil CEL2, column (2.5 μm, 150 × 2.1 mm) was used. Standard solutions of each compound were prepared in methanol. The conditions applied were as follows: CO_2_/MeOH = 80/20 (*v*/*v*), flow rate: 0.7 mL/min, (compounds **(*R*)-** **44** and **(** ***S*)-** **44**); CO_2_/MeOH = 88/12 (*v*/*v*), flow rate: 0.7 mL/min, (compounds **(*R*)-** **46**, **(*S*)-** **46**, **(*R*)-** **50** and **(*S*)-** **50**, detection at λ = 210 nm. Oven temperature: 40 °C.

#### General Method for the Preparation of Compounds (*R,S*)-**1**–(*R,S*)-**17**, (*R*)-**10**, (*S*)-**10**, (*R*)-**12**, (*S*)-**12**, (*R*)-**16** and (*S*)-**16**

DCC (1.13 g, 5.5 mmol, 1.2 equiv) was dissolved in 20 mL of DCM. Afterward, this solution was added to Boc-*O*-methyl-dl-serine (as racemates **(*R*,*S*)-1**–**(*R*,*S*)-17**) or Boc-*O*-methyl-d-serine (as *R*-enantiomers—**(*R*)-10, (*R*)-12, (*R*)-16**) or Boc-*O*-methyl-l-serine (as *S*-enantiomers - **(*S*)-10, (*S*)-12, (*S*)-16**) (1 g, 4.6 mmol, 1 equiv) dissolved in 20 mL of DCM (while stirring). After 0.5 h the respective commercial or noncommercial (**A10**-**A18**) piperazine derivatives (4.6 mmol, 1 equiv) dissolved in 5 mL of DCM was added in drops. The mixture was stirred for approximately 3 h at room temperature and evaporated to dryness. The column chromatography was applied for purification of crude products, using developing system S_1_. The desired compounds were obtained as light oils followed by concentration of organic solvents under reduced pressure.

#### (*R,S*)-Tert-butyl (3-methoxy-1-oxo-1-(4-phenylpiperazin-1-yl)­propan-2-yl)­carbamate (*R,S*)**-1**

Yellow oil, yield 88% (1.46 g); TLC: *R*_f_ = 0.78 (S_1_); UPLC (purity >99%): *t*_R_ = 6.23 min. LC–MS (ESI): *m*/*z* calcd for C_19_H_29_N_3_O_4_ (M + H)^+^, 364.22; found, 364.3.

#### (*R,S*)-Tert-butyl (1-(4-(3-fluorophenyl)­piperazin-1-yl)-3-methoxy-1-oxopropan-2-yl)­carbamate (*R,S*)**-2**

Yellow oil, yield 90% (1.57 g); TLC: *R*_f_ = 0.79 (S_1_); UPLC (purity = 96.55%): *t*_R_ = 6.75 min. LC–MS (ESI): *m*/*z* calcd for C_19_H_28_ FN_3_O_4_ (M + H)^+^, 382.21; found, 382.2.

#### (*R,S*)-Tert-butyl (1-(4-(4-fluorophenyl)­piperazin-1-yl)-3-methoxy-1-oxopropan-2-yl)­carbamate (*R,S*)**-3**

Yellow oil, yield 91% (1.58 g); TLC: *R*_f_ = 0.78 (S_1_); UPLC (purity >99%): *t*_R_ = 6.34 min. LC–MS (ESI): *m*/*z* calcd for C_19_H_28_FN_3_O_4_ (M + H)^+^, 382.21; found, 382.2.

#### (*R*,*S*)-*Tert*-butyl (1-(4-(3-chlorophenyl)­piperazin-1-yl)-3-methoxy-1-oxopropan-2-yl)­carbamate (*R*,*S*)**-4**

Yellow oil, yield 87% (1.58 g); TLC: *R*_f_ = 0.81 (S_1_); UPLC (purity >99%): *t*_R_ = 7.23 min. LC–MS (ESI): *m*/*z* calcd for C_19_H_28_ClN_3_O_4_ (M + H)^+^, 398.18; found, 398.4.

#### (*R,S*)-Tert-butyl (1-(4-(5-chlorophenyl)­piperazin-1-yl)-3-methoxy-1-oxopropan-2-yl)­carbamate (*R,S*)-5

Yellow oil, yield 91% (1.67 g); TLC: *R*_f_ = 0.81 (S_1_); UPLC (purity = 98.23%): *t*_R_ = 7.49 min. LC–MS (ESI): *m*/*z* calcd for C_19_H_28_ClN_3_O_4_ (M + H)^+^, 398.18; found, 398.4.

#### (*R,S*)-Tert-butyl (1-(4-(3,4-dichlorophenyl)­piperazin-1-yl)-3-methoxy-1-oxopropan-2-yl)­carbamate (*R,S*)**-6**

Yellow oil, yield 89% (1.75 g); TLC: *R*_f_ = 0.83 (S_1_); UPLC (purity >99%): *t*_R_ = 8.00 min. LC–MS (ESI): *m*/*z* calcd for C_19_H_27_Cl_2_N_3_O_4_ (M + H)^+^, 433.13; found, 433.1.

#### (*R,S*)-Tert-butyl (1-(4-(3,4-dichlorophenyl)­piperazin-1-yl)-3-methoxy-1-oxopropan-2-yl)­carbamate (*R,S*)**-7**

Yellow oil, yield 90% (1.77 g); TLC: *R*_f_ = 0.83 (S_1_); UPLC (purity = 98.58%): *t*_R_ = 8.03 min. LC–MS (ESI): *m*/*z* calcd for C_19_H_27_Cl_2_N_3_O_4_ (M + H)^+^, 433.13; found, 433.1.

#### (*R,S*)-Tert-butyl (3-methoxy-1-oxo-1-(4-(3-(trifluoromethyl)­phenyl)­piperazin-1-yl)­propan-2-yl)­carbamate (*R,S*)**-8**

Yellow oil, yield 87% (1.71 g); TLC: *R*_f_ = 0.82 (S_1_); UPLC (purity >99%): *t*_R_ = 7.52 min. LC–MS (ESI): *m*/*z* calcd for C_20_H_28_F_3_N_3_O_4_ (M + H)^+^, 432.21; found, 432.3.

#### (*R,S*)-Tert-butyl (3-methoxy-1-oxo-1-(4-(4-(trifluoromethyl)­phenyl)­piperazin-1-yl)­propan-2-yl)­carbamate (*R,S*)**-9**

Yellow oil, yield 85% (1.67 g); TLC: *R*_f_ = 0.82 (S_1_); UPLC (purity >99%): *t*_R_ = 7.33 min. LC–MS (ESI): *m*/*z* calcd for C_20_H_28_F_3_N_3_O_4_ (M + H)^+^, 432.21; found, 432.4.

#### (*R,S*)-Tert-butyl (1-(4-([1,1′-biphenyl]-3-yl)­piperazin-1-yl)-3-methoxy-1-oxopropan-2-yl)­carbamate (*R,S*)**-10**

Yellow oil, yield 87% (1.74 g); TLC: *R*_f_ = 0.77 (S_1_); UPLC (purity >99%): *t*_R_ = 7.77 min. LC–MS (ESI): *m*/*z* calcd for C_25_H_33_N_3_O_4_ (M + H)^+^, 440.25; found, 440.2.

#### (*R*)-Tert-butyl (1-(4-([1,1′-biphenyl]-3-yl)­piperazin-1-yl)-3-methoxy-1-oxopropan-2-yl)­carbamate (*R*)**-10**

Yellow oil, yield 89% (1.78 g); TLC: *R*_f_ = 0.76 (S_1_); UPLC (purity = 98.63%): *t*_R_ = 7.75 min. LC–MS (ESI): *m*/*z* calcd for C_25_H_33_N_3_O_4_ (M + H)^+^, 440.25; found, 440.2.

#### (*S*)-Tert-butyl (1-(4-([1,1′-biphenyl]-3-yl)­piperazin-1-yl)-3-methoxy-1-oxopropan-2-yl)­carbamate (*S*)**-10**

Yellow oil, yield 90% (1.80 g); TLC: *R*_f_ = 0.76 (S_1_); UPLC (purity >99%): *t*_R_ = 7.74 min. LC–MS (ESI): *m*/*z* calcd for C_25_H_33_N_3_O_4_ (M + H)^+^, 440.25; found, 440.2.

#### (*R,S*)-Tert-butyl (1-(4-([1,1′-biphenyl]-4-yl)­piperazin-1-yl)-3-methoxy-1-oxopropan-2-yl)­carbamate (*R,S*)**-11**

Yellow oil, yield 86% (1.72 g); TLC: *R*_f_ = 0.77 (S_1_); UPLC (purity = 96.90%): *t*_R_ = 7.83 min. LC–MS (ESI): *m*/*z* calcd for C_25_H_33_N_3_O_4_ (M + H)^+^, 440.25; found, 440.3.

#### (*R,S*)-Tert-butyl (3-methoxy-1-oxo-1-(4-(3-(trifluoromethoxy)­phenyl)­piperazin-1-yl)­propan-2-yl)­carbamate (*R,S*)**-12**

Yellow oil, yield 92% (1.88 g); TLC: *R*_f_ = 0.82 (S_1_); UPLC (purity >99%): *t*_R_ = 7.52 min. LC–MS (ESI): *m*/*z* calcd for C_20_H_28_F_3_N_3_O_5_ (M + H)^+^, 448.20; found, 448.4.

#### (*R*)-Tert-butyl (3-methoxy-1-oxo-1-(4-(3-(trifluoromethoxy)­phenyl)­piperazin-1-yl)­propan-2-yl)­carbamate (*R*)**-12**

Yellow oil, yield 88% (1.80 g); TLC: *R*_f_ = 0.81 (S_1_); UPLC (purity >99%): *t*_R_ = 7.54 min. LC–MS (ESI): *m*/*z* calcd for C_20_H_28_F_3_N_3_O_5_ (M + H)^+^, 448.20; found, 448.4.

#### (*S*)-Tert-butyl (3-methoxy-1-oxo-1-(4-(3-(trifluoromethoxy)­phenyl)­piperazin-1-yl)­propan-2-yl)­carbamate (*S*)**-12**

Yellow oil, yield 89% (1.82 g); TLC: *R*_f_ = 0.82 (S_1_); UPLC (purity >99%): *t*_R_ = 7.53 min. LC–MS (ESI): *m*/*z* calcd for C_20_H_28_F_3_N_3_O_5_ (M + H)^+^, 448.20; found, 448.3.

#### (*R,S*)-Tert-butyl (3-methoxy-1-oxo-1-(4-(4-(trifluoromethoxy)­phenyl)­piperazin-1-yl)­propan-2-yl)­carbamate (*R,S*)**-13**

Yellow oil, yield 89% (1.82 g); TLC: *R*_f_ = 0.81 (S_1_); UPLC (purity >97.44%): *t*_R_ = 7.54 min. LC–MS (ESI): *m*/*z* calcd for C_20_H_28_F_3_N_3_O_5_ (M + H)^+^, 448.20; found, 448.3.

#### (*R,S*)-Tert-butyl (3-methoxy-1-oxo-1-(4-(3-phenoxyphenyl)­piperazin-1-yl)­propan-2-yl)­carbamate (*R,S*)**-14**

Yellow oil, yield 87% (1.81 g); TLC: *R*_f_ = 0.84 (S_1_); UPLC (purity >98.78%): *t*_R_ = 7.86 min. LC–MS (ESI): *m*/*z* calcd for C_25_H_33_N_3_O_5_ (M + H)^+^, 456.25; found, 456.3.

#### (*R,S*)-Tert-butyl (3-methoxy-1-oxo-1-(4-(4-phenoxyphenyl)­piperazin-1-yl)­propan-2-yl)­carbamate (*R,S*)**-15**

Yellow oil, yield 88% (1.83 g); TLC: *R*_f_ = 0.84 (S_1_); UPLC (purity >99%): *t*_R_ = 7.89 min. LC–MS (ESI): *m*/*z* calcd for C_25_H_33_N_3_O_5_ (M + H)^+^, 456.25; found, 456.3.

#### (*R,S*)-Tert-butyl (3-methoxy-1-oxo-1-(4-(3-((trifluoromethyl)­thio)­phenyl)­piperazin-1-yl)­propan-2-yl)­carbamate (*R,S*)**-16**

Yellow oil, yield 89% (1.88 g); TLC: *R*_f_ = 0.85 (S_1_); UPLC (purity >99%): *t*_R_ = 8.86 min. LC–MS (ESI): *m*/*z* calcd for C_20_H_28_F_3_N_3_O_4_S (M + H)^+^, 464.18; found, 464.3.

#### (*R*)-Tert-butyl (3-methoxy-1-oxo-1-(4-(3-((trifluoromethyl)­thio)­phenyl)­piperazin-1-yl)­propan-2-yl)­carbamate (*R*)-**16**

Yellow oil, yield 91% (1.92 g); TLC: *R*_f_ = 0.85 (S_1_); UPLC (purity = 97.56%): *t*_R_ = 8.73 min. LC–MS (ESI): *m*/*z* calcd for C_20_H_28_F_3_N_3_O_4_S (M + H)^+^, 464.18; found, 464.2.

#### (*S*)-Tert-butyl (3-methoxy-1-oxo-1-(4-(3-((trifluoromethyl)­thio)­phenyl)­piperazin-1-yl)­propan-2-yl)­carbamate (*S*)**-16**

Yellow oil, yield 86% (1.82 g); TLC: *R*_f_ = 0.85 (S_1_); UPLC (purity >99%): *t*_R_ = 8.78 min. LC–MS (ESI): *m*/*z* calcd for C_20_H_28_F_3_N_3_O_4_S (M + H)^+^, 464.18; found, 464.2.

#### (*R,S*)-Tert-butyl (3-methoxy-1-oxo-1-(4-(4-((trifluoromethyl)­thio)­phenyl)­piperazin-1-yl)­propan-2-yl)­carbamate (*R,S*)**-17**

Yellow oil, yield 91% (1.92 g); TLC: *R*_f_ = 0.85 (S_1_); UPLC (purity = 97.45%): *t*_R_ = 8.39 min. LC–MS (ESI): *m*/*z* calcd for C_20_H_28_F_3_N_3_O_4_S (M + H)^+^, 464.18; found, 464.2.

#### General Method for the Preparation of Compounds (*R*,*S*)**-18**–**(** *R* **,** *S* **)-34, (** *R* **)-27**, **(** *S* **)-27, (** *R* **)-29**, **(** *S* **)-29**, **(** *R* **)-33** and **(** *S* **)-33**

The solution of **(*R*,*S*)-1**–**(*R*,*S*)-17, (*R*)-10**, **(*S*)-10, (*R*)-12**, **(*S*)-12**, **(*R*)-16** and **(*S*)-16** (3.8 mmol, 1 equiv) in DCM (30 mL) was treated with TFA (3 equiv) and stirred at room temperature for 3 h. Afterward, the organic solvents were evaporated to dryness. The resulting oil residue was dissolved in water (20 mL), and then 25% ammonium hydroxide was carefully added to pH = 8. The aqueous layer was extracted with DCM (3 × 20 mL), dried over Na_2_SO_4_, and concentrated to give the **(*R*,*S*)-18**–**(*R*,*S*)-34, (*R*)-27**, **(*S*)-27, (*R*)-29**, **(*S*)-29**, **(*R*)-33** and **(*S*)-33** as yellow oils. Intermediates **(** ***R*,*S*)-18**–**(*R*,*S*)-34, (*R*)-27**, **(** ***S*)-27, (*R*)-29**, **(*S*)-29**, **(*R*)-33** and **(*S*)-33** were advanced as substrates without purification for the last reaction.

#### (*R,S*)-2-Amino-3-methoxy-1-(4-phenylpiperazin-1-yl)­propan-1-one (*R,S*)**-18**

Yellow oil, yield 98% (0.98 g); TLC: *R*_f_ = 0.18 (S_2_); UPLC (purity >99%): *t*_R_ = 3.09 min. LC–MS (ESI): *m*/*z* calcd for C_14_H_21_N_3_O_2_ (M + H)^+^, 264.17; found, 264.3.

#### (*R,S*)-2-Amino-1-(4-(3-fluorophenyl)­piperazin-1-yl)-3-methoxypropan-1-one (*R,S*)-**19**

Yellow oil, yield 97% (1.04 g); TLC: *R*_f_ = 0.19 (S_2_); UPLC (purity >99%): *t*_R_ = 3.65 min. LC–MS (ESI): *m*/*z* calcd for C_14_H_20_FN_3_O_2_ (M + H)^+^, 282.16; found, 282.2.

#### (*R,S*)-2-Amino-1-(4-(4-fluorophenyl)­piperazin-1-yl)-3-methoxypropan-1-one (*R,S*)**-20**

Yellow oil, yield 98% (1.05 g); TLC: *R*_f_ = 0.19 (S_2_); UPLC (purity = 96.79%): *t*_R_ = 3.28 min. LC–MS (ESI): *m*/*z* calcd for C_14_H_20_FN_3_O_2_ (M + H)^+^, 282.16; found, 282.3.

#### (*R,S*)-2-Amino-1-(4-(3-Chlorophenyl)­piperazin-1-yl)-3-methoxypropan-1-one (*R,S*)**-21**

Yellow oil, yield 97% (1.10 g); TLC: *R*_f_ = 0.20 (S_2_); UPLC (purity >99%): *t*_R_ = 3.93 min. LC–MS (ESI): *m*/*z* calcd for C_14_H_20_ClN_3_O_2_ (M + H)^+^, 298.13; found, 298.5.

#### (*R,S*)-2-Amino-1-(4-(4-Chlorophenyl)­piperazin-1-yl)-3-methoxypropan-1-one (*R,S*)**-22**

Yellow oil, yield 96% (1.08 g); TLC: *R*_f_ = 0.20 (S_2_); UPLC (purity >99%): *t*_R_ = 3.99 min. LC–MS (ESI): *m*/*z* calcd for C_14_H_20_ClN_3_O_2_ (M + H)^+^, 298.13; found, 298.2.

#### (*R,S*)-2-Amino-1-(4-(3,4-dichlorophenyl)­piperazin-1-yl)-3-methoxypropan-1-one (*R,S*)**-23**

Yellow oil, yield 96% (1.08 g); TLC: *R*_f_ = 0.20 (S_2_); UPLC (purity = 98.32%): *t*_R_ = 4.60 min. LC–MS (ESI): *m*/*z* calcd for C_14_H_19_Cl_2_N_3_O_2_ (M + H)^+^, 332.09; found, 332.3.

#### (*R,S*)-2-Amino-1-(4-(3,5-dichlorophenyl)­piperazin-1-yl)-3-methoxypropan-1-one (*R,S*)**-24**

Yellow oil, yield 98% (1.23 g); TLC: *R*_f_ = 0.20 (S_2_); UPLC (purity >99%): *t*_R_ = 4.82 min. LC–MS (ESI): *m*/*z* calcd for C_14_H_19_Cl_2_N_3_O_2_ (M + H)^+^, 332.09; found, 332.3.

#### (*R,S*)-2-Amino-3-methoxy-1-(4-(3-(trifluoromethyl)­phenyl)­piperazin-1-yl)­propan-1-one (*R,S*)**-25**

Yellow oil, yield 96% (1.21 g); TLC: *R*_f_ = 0.20 (S_2_); UPLC (purity >99%): *t*_R_ = 4.56 min. LC–MS (ESI): *m*/*z* calcd for C_15_H_20_F_3_N_3_O_2_ (M + H)^+^, 332.15; found, 332.3.

#### (*R,S*)-2-Amino-3-methoxy-1-(4-(4-(trifluoromethyl)­phenyl)­piperazin-1-yl)­propan-1-one (*R,S*)-**26**

Yellow oil, yield 98% (1.23 g); TLC: *R*_f_ = 0.20 (S_2_); UPLC (purity >99%): *t*_R_ = 4.51 min. LC–MS (ESI): *m*/*z* calcd for C_15_H_20_F_3_N_3_O_2_ (M + H)^+^, 332.15; found, 332.3.

#### (*R,S*)-1-(4-([1,1′-biphenyl]-3-yl)­piperazin-1-yl)-2-Amino-3-methoxypropan-1-one (*R,S*)**-27**

Yellow oil, yield 97% (1.25 g); TLC: *R*_f_ = 0.20 (S_2_); UPLC (purity = 97.49%): *t*_R_ = 4.88 min. LC–MS (ESI): *m*/*z* calcd for C_20_H_25_N_3_O_2_ (M + H)^+^, 340.20; found, 340.3.

#### (*R*)-1-(4-([1,1′-biphenyl]-3-yl)­piperazin-1-yl)-2-amino-3-methoxypropan-1-one (*R*)**-27**

Yellow oil, yield 96% (1.24 g); TLC: *R*_f_ = 0.20 (S_2_); UPLC (purity >99%): *t*_R_ = 4.83 min. LC–MS (ESI): *m*/*z* calcd for C_20_H_25_N_3_O_2_ (M + H)^+^, 340.20; found, 340.3.

#### (*S*)-1-(4-([1,1′-biphenyl]-3-yl)­piperazin-1-yl)-2-amino-3-methoxypropan-1-one (*S*)**-27**

Yellow oil, yield 98% (1.26 g); TLC: *R*_f_ = 0.20 (S_2_); UPLC (purity = 98.66%): *t*_R_ = 4.82 min. LC–MS (ESI): *m*/*z* calcd for C_20_H_25_N_3_O_2_ (M + H)^+^, 340.20; found, 340.3.

#### (*R,S*)-1-(4-([1,1′-biphenyl]-4-yl)­piperazin-1-yl)-2-Amino-3-methoxypropan-1-one (*R,S*)**-28**

Yellow oil, yield 98% (1.26 g); TLC: *R*_f_ = 0.20 (S_2_); UPLC (purity >99%): *t*_R_ = 4.95 min. LC–MS (ESI): *m*/*z* calcd for C_20_H_25_N_3_O_2_ (M + H)^+^, 340.20; found, 340.3.

#### (*R,S*)-2-Amino-3-methoxy-1-(4-(3-(trifluoromethoxy)­phenyl)­piperazin-1-yl)­propan-1-one (*R,S*)**-29**

Yellow oil, yield 96% (1.27 g); TLC: *R*_f_ = 0.20 (S_2_); UPLC (purity >99%): *t*_R_ = 4.63 min. LC–MS (ESI): *m*/*z* calcd for C_15_H_20_F_3_N_3_O_3_ (M + H)^+^, 348.15; found, 348.4.

#### (*R*)-2-Amino-3-methoxy-1-(4-(3-(trifluoromethoxy)­phenyl)­piperazin-1-yl)­propan-1-one (*R*)**-29**

Yellow oil, yield 97% (1.28 g); TLC: *R*_f_ = 0.20 (S_2_); UPLC (purity >99%): *t*_R_ = 4.49 min. LC–MS (ESI): *m*/*z* calcd for C_15_H_20_F_3_N_3_O_3_ (M + H)^+^, 348.15; found, 348.1.

#### (*S*)-2-Amino-3-methoxy-1-(4-(3-(trifluoromethoxy)­phenyl)­piperazin-1-yl)­propan-1-one (*S*)**-29**

Yellow oil, yield 96% (1.27 g); TLC: *R*_f_ = 0.20 (S_2_); UPLC (purity >99%): *t*_R_ = 4.53 min. LC–MS (ESI): *m*/*z* calcd for C_15_H_20_F_3_N_3_O_3_ (M + H)^+^, 348.15; found, 348.2.

#### (*R,S*)-2-Amino-3-methoxy-1-(4-(4-(trifluoromethoxy)­phenyl)­piperazin-1-yl)­propan-1-one (*R,S*)**-30**

Yellow oil, yield 98% (1.30 g); TLC: *R*_f_ = 0.20 (S_2_); UPLC (purity = 97.44%): *t*_R_ = 4.66 min. LC–MS (ESI): *m*/*z* calcd for C_15_H_20_F_3_N_3_O_3_ (M + H)^+^, 348.15; found, 348.3.

#### (*R*,*S*)-2-Amino-3-methoxy-1-(4-(3-phenoxyphenyl)­piperazin-1-yl)­propan-1-one (*R*,*S*)-**31**

Yellow oil, yield 98% (1.32 g); TLC: *R*_f_ = 0.20 (S_2_); UPLC (purity >99%): *t*_R_ = 4.98 min. LC–MS (ESI): *m*/*z* calcd for C_20_H_25_N_3_O_3_ (M + H)^+^, 356.19; found, 356.3.

#### (*R*,*S*)-2-Amino-3-methoxy-1-(4-(4-phenoxyphenyl)­piperazin-1-yl)­propan-1-one (*R*,*S*)-**32**

Yellow oil, yield 96% (1.30 g); TLC: *R*_f_ = 0.20 (S_2_); UPLC (purity >99%): *t*_R_ = 4.88 min. LC–MS (ESI): *m*/*z* calcd for C_20_H_25_N_3_O_3_ (M + H)^+^, 356.19; found, 356.2.

#### (*R,S*)-2-Amino-3-methoxy-1-(4-(3-((trifluoromethyl)­thio)­phenyl)­piperazin-1-yl)­propan-1-one (*R,S*)**-33**

Yellow oil, yield 96% (1.32 g); TLC: *R*_f_ = 0.20 (S_2_); UPLC (purity = 98.45%): *t*_R_ = 5.38 min. LC–MS (ESI): *m*/*z* calcd for C_15_H_20_F_3_N_3_O_2_S (M + H)^+^, 364.13; found, 364.3.

#### (*R*)-2-Amino-3-methoxy-1-(4-(3-((trifluoromethyl)­thio)­phenyl)­piperazin-1-yl)­propan-1-one (*R*)**-33**

Yellow oil, yield 97% (1.34 g); TLC: *R*_f_ = 0.20 (S_2_); UPLC (purity >99%): *t*_R_ = 5.31 min. LC–MS (ESI): *m*/*z* calcd for C_15_H_20_F_3_N_3_O_2_S (M + H)^+^, 364.13; found, 364.2.

#### (*S*)-2-Amino-3-methoxy-1-(4-(3-((trifluoromethyl)­thio)­phenyl)­piperazin-1-yl)­propan-1-one (*S*)**-33**

Yellow oil, yield 98% (1.35 g); TLC: *R*_f_ = 0.20 (S_2_); UPLC (purity >99%): *t*_R_ = 5.42 min. LC–MS (ESI): *m*/*z* calcd for C_15_H_20_F_3_N_3_O_2_S (M + H)^+^, 364.13; found, 364.2.

#### (*R,S*)-2-Amino-3-methoxy-1-(4-(4-((trifluoromethyl)­thio)­phenyl)­piperazin-1-yl)­propan-1-one (*R,S*)**-34**

Yellow oil, yield 97% (1.34 g); TLC: *R*_f_ = 0.20 (S_2_); UPLC (purity = 96.78%): *t*_R_ = 5.38 min. LC–MS (ESI): *m*/*z* calcd for C_15_H_20_F_3_N_3_O_2_S (M + H)^+^, 364.13; found, 364.2.

#### General Method for the Preparation of final Compounds (*R,S*)-**35**–(*R,S*)-**51**, (*R*)-**44**, (*S*)-**44**, (*R*)-**46**, (*S*)-**46**, (*R*)-**50** and (*S*)-**50**

Intermediates **(*R*,*S*)-18**–**(*R*,*S*)-34**, **(*R*)-27**, **(** ***S*)-27**, **(*R*)-29**, **(*S*)-29**, **(*R*)-33** and **(*S*)-33** (3.5 mmol, 1 equiv) was dissolved in 20 mL of DCM. Afterward, triethylamine (TEA) (10.5 mmol, 3 equiv) was added while stirring at 0 °C to the solution. Final compounds **(*R*,*S*)-35**–**(*R*,*S*)-51**, (***R*)-44**, **(*S*)-44**, **(*R*)-46**, **(*S*)-46**, **(*R*)-50** and **(*S*)-50** were prepared by dropwise adding of acetyl chloride (5.3 mmol, 1.5 equiv) at 0 °C on the ice bath. The reaction mixture was allowed to warm up to room temperature and was stirred for an additional 2 h and evaporated to dryness. Next, the crude product was purified applying column chromatography using developing system S_2_. The desired compounds were obtained as white solids followed by concentration of organic solvents under reduced pressure and wash-up by diethyl ether.

#### (*R,S*)-*N*-(3-methoxy-1-oxo-1-(4-phenylpiperazin-1-yl)­propan-2-yl)­acetamide (*R,S*)**-35**

White solid. Yield: 90% (0.96 g); mp 176.6–177.8 °C; TLC: R_f_ **=** 0.43 (S_2_); UPLC (purity = 98.87%): *t*_R_ = 4.15 min. LC–MS (ESI): *m*/*z* calcd for C_16_H_23_N_3_O_3_ (M + H)^+^, 306.18; found, 306.2. ^1^H NMR (500 MHz, CDCl_3_) δ: 2.00 (s, 3 H), 3.10–3.16 (m, 2 H), 3.18–3.27 (m, 2 H), 3.31 (s, 3 H), 3.46 (dd, *J* = 9.0, 7.5 Hz, 1 H), 3.58 (dd, *J* = 9.1, 4.9 Hz, 1 H), 3.65–3.82 (m, 3 H), 3.84–3.92 (m, 1 H), 5.15 (td, *J* = 7.6, 5.0 Hz, 1 H), 6.64 (br d, *J* = 7.6 Hz, 1 H), 6.87–6.96 (m, 3 H), 7.26 (dd, *J* = 8.7, 7.5 Hz, 2 H). ^13^C NMR (126 MHz, CDCl_3_) δ: 23.3, 42.3, 45.9, 48.6, 49.3, 49.9, 59.4, 73.2, 116.7, 120.7, 129.4, 150.9, 168.8, 169.7.

#### (*R,S*)-*N*-(1-(4-(3-fluorophenyl)­piperazin-1-yl)-3-methoxy-1-oxopropan-2-yl)­acetamide (*R,S*)**-36**

White solid. Yield: 89% (1.01 g); mp 162.1–163.2 °C; TLC: R_f_ **=** 0.43 (S_2_); UPLC (purity >99%): *t*_R_ = 4.79 min. LC–MS (ESI): *m*/*z* calcd for C_16_H_22_FN_3_O_3_ (M + H)^+^, 324.17; found, 324.2. ^1^H NMR (500 MHz, CDCl_3_) δ: 2.00 (s, 3 H), 3.10–3.17 (m, 2 H), 3.19–3.26 (m, 2 H), 3.31 (s, 3 H), 3.45 (dd, *J* = 9.2, 7.5 Hz, 1 H), 3.59 (dd, *J* = 9.2, 4.9 Hz, 1 H), 3.65–3.73 (m, 2 H), 3.75–3.82 (m, 1 H), 3.89 (ddd, *J* = 12.9, 6.0, 3.2 Hz, 1 H), 5.14 (td, *J* = 7.7, 4.9 Hz, 1 H), 6.53–6.60 (m, 3 H), 6.65 (ddd, *J* = 8.4, 2.1, 1.0 Hz, 1 H), 7.19 (td, *J* = 8.2, 7.2 Hz, 1 H). ^13^C NMR (126 MHz, CDCl_3_) δ: 23.3, 42.1, 45.7, 48.6, 48.8, 49.3, 59.4, 73.2, 103.4 (d, *J* = 25.4 Hz) 106.9 (d, *J* = 21.1 Hz), 111.7 (d, *J* = 2.4 Hz), 130.4 (d, *J* = 9.7 Hz), 152.5 (d, *J* = 9.7 Hz), 163.9, (d, *J* = 243.9 Hz), 168.9, 169.7.

#### (*R,S*)-*N*-(1-(4-(4-fluorophenyl)­piperazin-1-yl)-3-methoxy-1-oxopropan-2-yl)­acetamide (*R,S*)**-37**

White solid. Yield: 91% (1.03 g); mp 164.5–165.9 °C; TLC: R_f_ **=** 0.43 (S_2_); UPLC (purity >99%): *t*_R_ = 4.83 min. LC–MS (ESI): *m*/*z* calcd for C_16_H_22_FN_3_O_3_ (M + H)^+^, 324.17; found, 324.2. ^1^H NMR (500 MHz, CDCl_3_) δ: 2.00 (s, 3 H), 2.98–3.16 (m, 4 H), 3.31 (s, 3 H), 3.46 (dd, *J* = 9.2, 7.5 Hz, 1 H), 3.58 (dd, *J* = 9.0, 5.0 Hz, 1 H), 3.65–3.81 (m, 3 H), 3.84–3.94 (m, 1 H), 5.14 (td, *J* = 7.7, 5.0 Hz, 1 H), 6.59 (br d, *J* = 7.7 Hz, 1 H), 6.81–6.90 (m, 2 H), 6.92–7.01 (m, 2 H). ^13^C NMR (126 MHz, CDCl_3_) δ: 23.3, 42.4, 46.0, 48.6, 50.4, 50.9, 59.4, 73.2, 115.8 (d, *J* = 22.3 Hz), 118.7 (d, *J* = 7.9 Hz), 147.5 (d, *J* = 2.4 Hz), 157.7 (d, *J* = 239.6 Hz), 168.8, 169.7.

#### (*R,S*)-*N*-(1-(4-(3-Chlorophenyl)­piperazin-1-yl)-3-methoxy-1-oxopropan-2-yl)­acetamide (*R,S*)**-38**

White solid. Yield: 87% (1.04 g); mp 156.4–157.8 °C; TLC: R_f_ **=** 0.43 (S_2_); UPLC (purity >99%): *t*_R_ = 5.19 min. LC–MS (ESI): *m*/*z* calcd for C_16_H_22_ClN_3_O_3_ (M + H)^+^, 340.14; found, 340.5. ^1^H NMR (500 MHz, CDCl_3_) δ: 1.99 (s, 3 H), 3.11 (br d, *J* = 7.7 Hz, 2 H), 3.17–3.26 (m, 2 H), 3.30 (s, 3 H), 3.45 (dd, *J* = 9.0, 7.6 Hz, 1 H), 3.58 (dd, *J* = 9.0, 4.9 Hz, 1 H), 3.63–3.71 (m, 2 H), 3.74–3.81 (m, 1 H), 3.87 (td, *J* = 6.6, 2.9 Hz, 1 H), 5.14 (td, *J* = 7.7, 4.9 Hz, 1 H), 6.62 (br d, *J* = 7.7 Hz, 1 H), 6.73–6.79 (m, 1 H), 6.81–6.88 (m, 2 H), 7.16 (t, *J* = 8.0 Hz, 1 H). ^13^C NMR (126 MHz, CDCl_3_) δ: 23.3, 42.1, 45.7, 48.6, 48.8, 49.4, 59.3, 73.1, 114.5, 116.4, 120.3, 130.3, 135.1, 151.9, 168.9, 169.7.

#### (*R,S*)-*N*-(1-(4-(4-Chlorophenyl)­piperazin-1-yl)-3-methoxy-1-oxopropan-2-yl)­acetamide (*R,S*)**-39**

White solid. Yield: 91% (1.08 g); mp 153.2–154.7 °C; TLC: R_f_ **=** 0.43 (S_2_); UPLC (purity >99%): *t*_R_ = 5.23 min. LC–MS (ESI): *m*/*z* calcd for C_16_H_22_ClN_3_O_3_ (M + H)^+^, 340.14; found, 340.5. ^1^H NMR (500 MHz, CDCl_3_) δ: 2.00 (s, 3 H), 3.04–3.12 (m, 2 H), 3.12–3.21 (m, 2 H), 3.31 (s, 3 H), 3.45 (dd, *J* = 9.2, 7.5 Hz, 1 H), 3.58 (dd, *J* = 9.2, 4.9 Hz, 1 H), 3.65–3.82 (m, 3 H), 3.88 (br dd, *J* = 6.7, 3.0 Hz, 1 H), 5.14 (td, *J* = 7.7, 4.9 Hz, 1 H), 6.57 (br d, *J* = 7.7 Hz, 1 H), 6.78–6.90 (m, 2 H), 7.18–7.24 (m, 2 H). ^13^C NMR (126 MHz, CDCl_3_) δ: 23.3, 42.2, 45.8, 48.6, 49.3, 49.9, 59.4, 73.2, 117.9, 125.6, 129.2, 149.5, 168.9, 169.7.

#### (*R,S*)-*N*-(1-(4-(3,4-dichlorophenyl)­piperazin-1-yl)-3-methoxy-1-oxopropan-2-yl)­acetamide (*R,S*)**-40**

White solid. Yield: 85% (1.11 g); mp 144.1–145.6 °C; TLC: R_f_ **=** 0.48 (S_2_); UPLC (purity >99%): *t*_R_ = 6.34 min. LC–MS (ESI): *m*/*z* calcd for C_16_H_21_Cl_2_N_3_O_3_ (M + H)^+^, 375.10; found, 375.1. ^1^H NMR (500 MHz, CDCl_3_) δ: 1.99 (s, 3 H), 3.03–3.13 (m, 2 H), 3.14–3.24 (m, 2 H), 3.30 (s, 3 H), 3.44 (dd, *J* = 8.9, 7.7 Hz, 1 H), 3.58 (dd, *J* = 9.0, 5.0 Hz, 1 H), 3.63–3.71 (m, 2 H), 3.77 (br dd, *J* = 12.7, 3.3 Hz, 1 H), 3.88 (td, *J* = 6.6, 2.6 Hz, 1 H), 5.13 (td, *J* = 7.8, 5.0 Hz, 1 H), 6.58 (br d, *J* = 7.7 Hz, 1 H), 6.72 (dd, *J* = 8.9, 2.9 Hz, 1 H), 6.93 (d, *J* = 2.9 Hz, 1 H), 7.27 (d, *J* = 8.9 Hz, 1 H). ^13^C NMR (126 MHz, CDCl_3_) δ: 23.3, 42.0, 45.6, 48.6, 48.8, 49.3, 59.4, 73.1, 115.9, 117.9, 123.3, 130.7, 133.0, 150.2, 169.0, 169.7.

#### (*R,S*)-*N*-(1-(4-(3,5-dichlorophenyl)­piperazin-1-yl)-3-methoxy-1-oxopropan-2-yl)­acetamide (*R,S*)**-41**

White solid. Yield: 92% (1.21 g); mp 141.4–142.6 °C; TLC: R_f_ **=** 0.48 (S_2_); UPLC (purity = 98.75%): *t*_R_ = 6.46 min. LC–MS (ESI): *m*/*z* calcd for C_16_H_21_Cl_2_N_3_O_3_ (M + H)^+^, 375.10; found, 375.1. ^1^H NMR (500 MHz, CDCl_3_) δ: 2.00 (s, 3 H), 3.13 (br dd, *J* = 7.5, 3.4 Hz, 2 H), 3.18–3.28 (m, 2 H), 3.31 (s, 3 H), 3.40–3.49 (m, 1 H), 3.59 (dd, *J* = 8.9, 4.9 Hz, 1 H), 3.63–3.72 (m, 2 H), 3.74–3.83 (m, 1 H), 3.89 (dt, *J* = 6.7, 3.1 Hz, 1 H), 5.13 (td, *J* = 7.7, 4.9 Hz, 1 H), 6.52 (br d, *J* = 7.7 Hz, 1 H), 6.73 (d, *J* = 1.7 Hz, 2 H), 6.8 (t, *J* = 1.7 Hz, 1 H). ^13^C NMR (126 MHz, CDCl_3_) δ: 23.3, 42.0, 45.5, 48.3, 48.6, 48.9, 59.4, 73.1, 114.4, 119.8, 135.7, 152.2, 169.0, 169.7.

#### (*R,S*)-*N*-(3-methoxy-1-oxo-1-(4-(3-(trifluoromethyl)­phenyl)­piperazin-1-yl)­propan-2-yl)­acetamide (*R,S*)**-42**

White solid. Yield: 91% (1.19 g); mp 125.6–126.7 °C; TLC: R_f_ **=** 0.51 (S_2_); UPLC (purity >99%): *t*_R_ = 6.20 min. LC–MS (ESI): *m*/*z* calcd for C_17_H_22_F_3_N_3_O_3_ (M + H)^+^, 374.16; found, 374.3. ^1^H NMR (500 MHz, CDCl_3_) δ: 2.00 (s, 3 H), 3.11–3.20 (m, 2 H), 3.21–3.29 (m, 2 H), 3.31 (s, 3 H), 3.46 (dd, *J* = 9.0, 7.6 Hz, 1 H), 3.59 (dd, *J* = 9.2, 4.9 Hz, 1 H), 3.66–3.75 (m, 2 H), 3.77–3.86 (m, 1 H), 3.87–3.95 (m, 1 H), 5.15 (td, *J* = 7.7, 4.9 Hz, 1 H), 6.60 (br d, *J* = 7.2 Hz, 1 H), 7.04 (dd, *J* = 8.3, 2.3 Hz, 1 H), 7.08–7.15 (m, 2 H), 7.35 (t, *J* = 8.0 Hz, 1 H). ^13^C NMR (126 MHz, CDCl_3_) δ: 23.3, 42.1, 45.7, 48.6, 48.8, 49.4, 59.3, 73.1, 112.8 (q, *J* = 3.6 Hz), 116.84 (q, *J* = 3.6 Hz), 119.4, 124.2 (q, *J* = 272.8 Hz), 129.8, 131.7 (q, *J* = 32.0 Hz), 151.0, 169.0, 169.7.

#### (*R,S*)-*N*-(3-methoxy-1-oxo-1-(4-(4-(trifluoromethyl)­phenyl)­piperazin-1-yl)­propan-2-yl)­acetamide (*R,S*)**-43**

White solid. Yield: 89% (1.17 g); mp 122.3–123.8 °C; TLC: R_f_ **=** 0.51 (S_2_); UPLC (purity = 98.97%): *t*_R_ = 6.18 min. LC–MS (ESI): *m*/*z* calcd for C_17_H_22_F_3_N_3_O_3_ (M + H)^+^, 374.16; found, 374.2. ^1^H NMR (500 MHz, CDCl_3_) δ: 2.00 (s, 3 H), 3.23 (br dd, *J* = 7.6, 3.9 Hz, 2 H), 3.28–3.37 (m, 5 H), 3.45 (dd, *J* = 8.9, 7.7 Hz, 1 H), 3.60 (dd, *J* = 9.0, 5.0 Hz, 1 H), 3.66–3.74 (m, 2 H), 3.78–3.84 (m, 1 H), 3.91 (td, *J* = 6.5, 2.7 Hz, 1 H), 5.15 (td, *J* = 7.7, 4.9 Hz, 1 H), 6.52 (br d, *J* = 7.7 Hz, 1 H), 6.91 (d, *J* = 8.6 Hz, 2 H), 7.49 (d, *J* = 8.6 Hz, 2 H). ^13^C NMR (126 MHz, CDCl_3_) δ: 23.3, 42.0, 45.5, 48.1, 48.6, 59.4, 73.2, 115.2, 121.51 (q, *J* = 32.0 Hz), 124.63 (q, *J* = 271.0 Hz, 1 C), 126.6 (q, *J* = 3.6 Hz), 152.9, 169.0, 169.7.

#### (*R,S*)-*N*-(1-(4-([1,1′-biphenyl]-3-yl)­piperazin-1-yl)-3-methoxy-1-oxopropan-2-yl)­acetamide (*R,S*)**-44**

White solid. Yield: 89% (1.19 g); mp 148.3–149.6 °C; TLC: R_f_ **=** 0.51 (S_2_); UPLC (purity >99%): *t*_R_ = 6.09 min. LC–MS (ESI): *m*/*z* calcd for C_22_H_27_N_3_O_3_ (M + H)^+^, 382.21; found, 382.2. ^1^H NMR (500 MHz, CDCl_3_) δ: 2.02 (s, 3 H), 3.34 (s, 3 H), 3.43–3.65 (m, 5 H), 3.72–3.82 (m, 1 H), 4.16–4.59 (m, 3 H), 5.06 (br s, 1 H), 6.63 (br s, 1 H), 7.32–7.49 (m, 3 H), 7.57 (br d, *J* = 7.5 Hz, 3 H), 7.68 (br d, *J* = 7.7 Hz, 1 H), 7.76 (br s, 1 H), 8.08 (br s, 1 H). ^13^C NMR (126 MHz, CDCl_3_) δ: 23.1, 39.7, 43.3, 48.7, 52.7, 55.2, 59.4, 72.5, 119.7, 120.1, 127.2, 128.6, 128.9, 129.2, 131.0, 138.8, 142.5, 144.1, 169.6, 170.4.

#### (*R*)-*N*-(1-(4-([1,1′-biphenyl]-3-yl)­piperazin-1-yl)-3-methoxy-1-oxopropan-2-yl)­acetamide (*R*)**-44**

White solid. Yield: 90% (1.21 g); mp 153.6–154.8 °C; TLC: R_f_ **=** 0.51 (S_2_); UPLC (purity = 98.12%): *t*_R_ = 6.08 min. LC–MS (ESI): *m*/*z* calcd for C_22_H_27_N_3_O_3_ (M + H)^+^, 382.21; found, 382.2. UPLC/HRMS (purity = 98.01%): *t*_R_ = 6.27 min. HRMS (ESI-QTOF): *m*/*z* calcd for C_22_H_27_N_3_O_3_ (M + H)^+^, 382.2086; found, 382.2153. ^1^H NMR (500 MHz, CDCl_3_) δ: 2.00 (s, 3 H), 3.32 (s, 3 H), 3.44–3.55 (m, 4 H), 3.59 (dd, *J* = 8.9, 5.44 Hz, 1 H), 3.71 (br s, 1 H), 4.07–4.54 (m, 4 H), 4.98–5.12 (m, 1 H), 6.63 (br d, *J* = 5.0 Hz, 1 H), 7.33–7.39 (m, 1 H), 7.40–7.46 (m, 2 H), 7.49–7.59 (m, 3 H), 7.62 (br d, *J* = 7.59 Hz, 1 H), 7.67 (br s, 1 H), 7.98 (br s, 1 H). ^13^C NMR (126 MHz, CDCl_3_) δ: 23.1, 39.9, 43.5, 48.6, 54.7 (br d, *J* = 0.9 Hz) 59.4, 72.5, (br d, *J* = 1.05 Hz), 119.3 (br d, *J* = 0.9 Hz), 119.7 (br s), 127.2, 128.5, 129.2, 130.9, 139.0, 143.9, 169.5, 170.3 (br d, *J* = 1.20 Hz). Chiral SFC >98% ee (t_R_ = 4.156 min).

#### (*S*)-*N*-(1-(4-([1,1′-biphenyl]-3-yl)­piperazin-1-yl)-3-methoxy-1-oxopropan-2-yl)­acetamide (*S*)**-44**

White solid. Yield: 87% (1.17 g); mp 155.2–156.4 °C; TLC: R_f_ **=** 0.51 (S_2_); UPLC (purity >99%): *t*_R_ = 6.06 min. LC–MS (ESI): *m*/*z* calcd for C_22_H_27_N_3_O_3_ (M + H)^+^, 382.21; found, 382.2. UPLC/HRMS (purity >99%): *t*_R_ = 6.27 min. HRMS (ESI-QTOF): *m*/*z* calcd for C_22_H_27_N_3_O_3_ (M + H)^+^, 382.2086; found, 382.2153. ^1^H NMR (500 MHz, CDCl_3_) δ: 1.94 (s, 3 H), 3.27 (s, 3 H), 3.40–3.60 (m, 4 H), 3.73–3.89 (m, 1 H), 4.03–4.32 (m, 5 H), 4.92 (br s, 1 H), 7.27–7.45 (m, 3 H), 7.57 (br s, 3 H), 7.60–7.75 (m, 2 H), 8.02 (br s, 1 H). ^13^C NMR (126 MHz, CDCl_3_) δ: 23.1, 39.9 (br d, *J* = 0.8) 43.5 (br d, *J* = 0.6 Hz), 48.6, 54.7 (d, *J* = 5.9 Hz) 59.4, 72.6, 119.4 (br d, *J* = 0.8 Hz) 119.4, 119,7, 127.2, 128.6, 129.2, 130.9, 139.0, 144.0, 169.5, 170.2 (br d, *J* = 0.9 Hz). Chiral SFC >98% ee (t_R_ = 4.942 min).

#### (*R,S*)-*N*-(1-(4-([1,1′-biphenyl]-4-yl)­piperazin-1-yl)-3-methoxy-1-oxopropan-2-yl)­acetamide (*R,S*)**-45**

White solid. Yield: 92% (1.23 g); mp 132.6–133.9 °C; TLC: R_f_ **=** 0.51 (S_2_); UPLC (purity = 98.67%): *t*_R_ = 5.97 min. LC–MS (ESI): *m*/*z* calcd for C_22_H_27_N_3_O_3_ (M + H)^+^, 382.21; found, 382.3. ^1^H NMR (500 MHz, CDCl_3_) δ: 2.01 (s, 3 H), 3.14–3.22 (m, 2 H), 3.24–3.31 (m, 2 H), 3.32 (s, 3 H), 3.47 (dd, *J* = 9.2, 7.5 Hz, 1 H), 3.61 (dd, *J* = 9.2, 4.9 Hz, 1 H), 3.67–3.77 (m, 2 H), 3.78–3.84 (m, 1 H), 3.88–3.97 (m, 1 H), 5.16 (td, *J* = 7.6, 4.9 Hz, 1 H), 6.56 (br d, *J* = 7.7 Hz, 1 H), 6.95–7.04 (m, 2 H), 7.26–7.32 (m, 1 H), 7.36–7.46 (m, 2 H), 7.49–7.59 (m, 4 H). ^13^C NMR (126 MHz, CDCl_3_) δ: 23.4, 42.3, 45.9, 48.7, 49.2, 49.7, 59.4, 73.2, 116.8, 126.7, 126.7, 128.0, 128.8, 133.4, 140.7, 150.1, 168.9, 169.7.

#### (*R,S*)-*N*-(3-methoxy-1-oxo-1-(4-(3-(trifluoromethoxy)­phenyl)­piperazin-1-yl)­propan-2-yl)­acetamide (*R,S*)**-46**

White solid. Yield: 86% (1.18 g); mp 121.3–122.7 °C; TLC: R_f_ **=** 0.51 (S_2_); UPLC (purity >99%): *t*_R_ = 5.83 min. LC–MS (ESI): *m*/*z* calcd for C_17_H_22_F_3_N_3_O_4_ (M + H)^+^, 390.16; found, 390.5. ^1^H NMR (500 MHz, CDCl_3_) δ: 2.02 (s, 3 H), 3.34 (s, 3 H), 3.43–3.53 (m, 4 H), 3.61 (dd, *J* = 8.9, 5.4 Hz, 1 H), 3.67–3.76 (m, 1 H), 4.18–4.46 (m, 4 H), 5.00–5.10 (m, 1 H), 6.50 (br d, *J* = 7.2 Hz, 1 H), 7.32 (dt, *J* = 8.5, 0.9 Hz, 1 H), 7.56 (t, *J* = 8.3 Hz, 1 H), 7.64 (s, 1 H), 7.84 (dd, *J* = 8.2, 1.6 Hz, 1 H). ^13^C NMR (126 MHz, CDCl_3_) δ: 23.3, 42.1, 45.6, 48.6, 48.7, 49.3, 59.4, 73.2, 109.0, 112.2, 114.4, 120.5 (q, *J* = 257.0 Hz), 130.3, 150.4 (d, *J* = 1.8 Hz), 152.1, 169.0, 169.7.

#### (*R*)-*N*-(3-methoxy-1-oxo-1-(4-(3-(trifluoromethoxy)­phenyl)­piperazin-1-yl)­propan-2-yl)­acetamide (*R*)**-46**

White solid. Yield: 91% (1.25 g); mp 128.1–129.6 °C; TLC: R_f_ **=** 0.51 (S_2_); UPLC (purity = 98.25%): *t*_R_ = 5.78 min. LC–MS (ESI): *m*/*z* calcd for C_17_H_22_F_3_N_3_O_4_ (M + H)^+^, 390.16; found, 390.2. UPLC/HRMS (purity = 98.20%): *t*_R_ = 6.08 min. HRMS (ESI-QTOF): *m*/*z* calcd for C_17_H_22_F_3_N_3_O_4_ (M + H)^+^, 390.1596, found, 390.1650. ^1^H NMR (500 MHz, CDCl_3_) δ: 2.00 (s, 3 H), 3.32 (s, 3 H), 3.40–3.53 (m, 4 H), 3.59 (dd, *J* = 8.9, 5.6 Hz, 1 H), 3.64–3.71 (m, 1 H), 4.17–4.37 (m, 4 H), 4.99–5.09 (m, 1 H), 6.61 (br d, *J* = 7.0 Hz, 1 H), 7.23–7.29 (m, 1 H), 7.53 (t, *J* = 8.2 Hz, 1 H), 7.58 (s, 1 H), 7.75 (dd, *J* = 8.2, 1.4 Hz, 1 H). ^13^C NMR (126 MHz, CDCl_3_) δ: 23.1, 39.9, 43.5, 48.6, 54.4 (br d, *J* = 8.7 Hz), 54.3, 54.4, 59.4, 72.5, 113.9, 119.5, 120.6 (q, *J* = 257.0 Hz), 121.3, 131.9, 144.2, 150.2, (d, *J* = 1.9 Hz), 169.5, 170.2. Chiral SFC >98% ee (t_R_ = 2.766 min).

#### (*S*)-*N*-(3-methoxy-1-oxo-1-(4-(3-(trifluoromethoxy)­phenyl)­piperazin-1-yl)­propan-2-yl)­acetamide (*S*)**-46**

White solid. Yield: 88% (1.21 g); mp 127.7–129.1 °C; TLC: R_f_ **=** 0.51 (S_2_); UPLC (purity >99%): *t*_R_ = 5.80 min. LC–MS (ESI): *m*/*z* calcd for C_17_H_22_F_3_N_3_O_4_ (M + H)^+^, 390.16; found, 390.2. UPLC/HRMS (purity >99%): *t*_R_ = 6.08 min. HRMS (ESI-QTOF): *m*/*z* calcd for C_17_H_22_F_3_N_3_O_4_ (M + H)^+^, 390.1596; found, 390.1650. ^1^H NMR (500 MHz, CDCl_3_) δ: 2.00 (s, 3 H), 3.10–3.19 (m, 2 H), 3.21–3.29 (m, 2 H) 3.31 (s, 3 H), 3.46 (dd, *J* = 9.0, 7.7 Hz, 1 H), 3.59 (dd, *J* = 9.0, 4.9 Hz, 1 H), 3.65–3.75 (m, 2 H) 3.76–3.84 (m, 1 H), 3.87–3.97 (m, 1 H), 5.14 (td, *J* = 7.7, 4.9 Hz, 1 H), 6.53 (br d, *J* = 7.7 Hz, 1 H), 6.67–6.76 (m, 2 H), 6.80 (dd, *J* = 8.4, 2.2 Hz, 1 H), 7.21–7.29 (m, 1 H). ^13^C NMR (126 MHz, CDCl_3_) δ: 23.3, 42.1, 45.6, 48.6, 48.7, 49.3, 59.4, 73.2, 109.0 (d, *J* = 0.6 Hz), 112.2, 114.4, 120.6 (q, *J* = 257.0 Hz), 130.3, 150.4 (d, *J* = 1.8 Hz), 152.1, 169.0, 169.7. Chiral SFC >98% ee (t_R_ = 3.238 min).

#### (*R,S*)-*N*-(3-methoxy-1-oxo-1-(4-(4-(trifluoromethoxy)­phenyl)­piperazin-1-yl)­propan-2-yl)­acetamide (*R,S*)**-47**

White solid. Yield: 89% (1.22 g); mp 135.6–136.7 °C; TLC: R_f_ **=** 0.51 (S_2_); UPLC (purity >99%): *t*_R_ = 5.72 min. LC–MS (ESI): *m*/*z* calcd for C_17_H_22_F_3_N_3_O_4_ (M + H)^+^, 390.16; found, 390.2. ^1^H NMR (500 MHz, CDCl_3_) δ: 2.01 (s, 3 H), 3.34 (s, 3 H), 3.39–3.53 (m, 4 H), 3.61 (dd, *J* = 8.9, 5.7 Hz, 1 H), 3.69 (br s, 1 H), 4.18–4.49 (m, 4 H), 4.98–5.07 (m, 1 H), 6.48 (br d, *J* = 6.9 Hz, 1 H), 7.34 (d, *J* = 8.6 Hz, 2 H), 7.87 (br d, *J* = 8.9 Hz, 2 H)·^13^C NMR (126 MHz, CDCl_3_) δ: 23.1, 39.8, 43.5, 48.7, 54.9, 59.4, 72.4, 120.3 (q, *J* = 259.1 Hz), 122.8, 123.1, 140.7, 149.5 (br d, *J* = 0.6 Hz), 169.6, 170.4.

#### (*R,S*)-*N*-(3-methoxy-1-oxo-1-(4-(3-phenoxyphenyl)­piperazin-1-yl)­propan-2-yl)­acetamide (*R,S*)**-48**

White solid. Yield: 87% (1.21 g); mp 144.1–145.4 °C; TLC: R_f_ **=** 0.51 (S_2_); UPLC (purity = 98.34%): *t*_R_ = 6.05 min. LC–MS (ESI): *m*/*z* calcd for C_22_H_27_N_3_O_4_ (M + H)^+^, 398.20; found, 398.2. ^1^H NMR (500 MHz, CDCl_3_) δ: 2.00 (s, 1 H), 3.06–3.15 (m, 1 H), 3.16–3.26 (m, 1 H), 3.31 (s, 1 H), 3.45 (dd, *J* = 8.9, 7.5 Hz, 1 H), 3.58 (dd, *J* = 8.9, 4.9 Hz, 1 H), 3.63–3.73 (m, 1 H), 3.74–3.82 (m, 1 H), 3.87 (td, *J* = 6.5, 3.0 Hz, 1 H), 5.13 (td, *J* = 7.6, 4.9 Hz, 1 H), 6.44–6.55 (m, 1 H), 6.58 (t, *J* = 2.3 Hz, 1 H), 6.62–6.68 (m, 1 H), 6.94–7.03 (m, 1 H), 7.06–7.12 (m, 1 H), 7.20 (t, *J* = 8.2 Hz, 1 H), 7.29–7.36 (m, 1 H). ^13^C NMR (126 MHz, CDCl_3_) δ: 23.3, 42.2, 45.8, 48.6, 49.0, 49.5, 59.4, 73.2, 107.2, 110.6, 111.3, 119.0, 123.3, 129.8, 130.3, 152.4, 157.2, 158.3, 168.8, 169.7.

#### (*R,S*)-*N*-(3-methoxy-1-oxo-1-(4-(4-phenoxyphenyl)­piperazin-1-yl)­propan-2-yl)­acetamide (*R,S*)**-49**

White solid. Yield: 92% (1.28 g); mp 148.6–149.9 °C; TLC: R_f_ **=** 0.51 (S_2_); UPLC (purity >99%): *t*_R_ = 5.84 min. LC–MS (ESI): *m*/*z* calcd for C_22_H_27_N_3_O_4_ (M + H)^+^, 398.20; found, 398.2. ^1^H NMR (500 MHz, CDCl_3_) δ: 2.01 (s, 3 H), 3.08 (br dd, *J* = 7.5, 3.7 Hz, 2 H), 3.12–3.22 (m, 2 H), 3.32 (s, 3 H), 3.43–3.53 (m, 1 H), 3.6 (dd, *J* = 9.2, 4.9 Hz, 1 H), 3.66–3.83 (m, 3 H), 3.86–3.95 (m, 1 H), 5.15 (td, *J* = 7.6, 4.9 Hz, 1 H), 6.54 (br d, *J* = 7.7 Hz, 1 H), 6.86–6.99 (m, 6 H), 7.04 (t, *J* = 7.5 Hz, 1 H), 7.26–7.34 (m, 2 H). ^13^C NMR (126 MHz, CDCl_3_) δ: 23.4, 42.4, 46.0, 48.7, 50.2, 50.8, 59.4, 73.2, 117.9, 118.5, 120.5, 122.7, 129.7, 147.3, 150.8, 158.3, 168.8, 169.7.

#### (*R,S*)-*N*-(3-methoxy-1-oxo-1-(4-(3-((trifluoromethyl)­thio)­phenyl)­piperazin-1-yl)­propan-2-yl)­acetamide (*R,S*)**-50**

White solid. Yield: 91% (1.29 g); mp 119.6–121.0 °C; TLC: R_f_ **=** 0.51 (S_2_); UPLC (purity >99%): *t*_R_ = 6.78 min. LC–MS (ESI): *m*/*z* calcd for C_17_H_22_F_3_N_3_O_3_S (M + H)^+^, 406.14; found, 406.2. ^1^H NMR (500 MHz, CDCl_3_) δ 2.01 (s, 3 H), 3.34 (s, 3 H), 3.36–3.44 (m, 2 H), 3.44–3.50 (m, 1 H), 3.56 (br s, 1 H), 3.61 (dd, *J* = 9.0, 5.3 Hz, 1 H), 4.08–4.26 (m, 4 H), 5.08 (td, *J* = 7.6, 5.4 Hz, 1 H), 6.45 (br d, *J* = 7.2 Hz, 1 H), 7.45–7.53 (m, 1 H), 7.53–7.59 (m, 1 H), 7.70 (s, 1 H), 7.77 (br d, *J* = 8.0 Hz, 1 H). ^13^C NMR (126 MHz, CDCl_3_) δ: 23.3, 42.1, 45.7, 48.6, 48.8, 49.3, 59.4, 73.2) 118.6, 123.8, 125.3, 129.7 (q, *J* = 308.0 Hz), 127.8, 130.2, 151.5, 169.0, 169.7.

#### (*R*)-*N*-(3-methoxy-1-oxo-1-(4-(3-((trifluoromethyl)­thio)­phenyl)­piperazin-1-yl)­propan-2-yl)­acetamide (*R*)**-50**

White solid. Yield: 90% (1.28 g); mp 124.7–126.1 °C; TLC: R_f_ **=** 0.51 (S_2_); UPLC (purity >99%): *t*_R_ = 6.83 min. LC–MS (ESI): *m*/*z* calcd for C_17_H_22_F_3_N_3_O_3_S (M + H)^+^, 406.14; found, 406.2. UPLC/HRMS (purity = 98.9%): *t*_R_ = 6.45 min. HRMS (ESI-QTOF): *m*/*z* calcd for C_17_H_22_F_3_N_3_O_3_S (M + H)^+^, 406.1368; found, 406.1445. ^1^H NMR (500 MHz, CDCl_3_) δ: 2.01 (s, 3 H), 3.34 (s, 3 H), 3.36–3.44 (m, 3 H), 3.48 (d, *J* = 8.0 Hz, 1 H), 3.56 (br s, 1 H), 3.61 (dd, *J* = 9.0, 5.3 Hz, 1 H), 4.07–4.27 (m, 4 H), 5.08 (td, *J* = 7.6, 5.4 Hz, 1 H), 6.45 (br d, *J* = 7.2 Hz, 1 H), 7.47–7.53 (m, 1 H), 7.54–7.60 (m, 1 H), 7.70 (s, 1 H), 7.77 (br d, *J* = 8.0 Hz, 1 H). ^13^C NMR (126 MHz, CDCl_3_) δ: 23.2, 40.4, 44.1, 48.6, 52.9, 53.2, 59.4, 72.8, 122.6, 126.8, 126.9, 129.3 (q, *J* = 308.4 Hz), 131.3, 134.4, 145.76,169.4, 170.0. Chiral SFC >98% ee (t_R_ = 4.195 min).

#### (*S*)-*N*-(3-methoxy-1-oxo-1-(4-(3-((trifluoromethyl)­thio)­phenyl)­piperazin-1-yl)­propan-2-yl)­acetamide (*S*)**-50**

White solid. Yield: 88% (1.25 g); mp 127.3–128.9 °C; TLC: R_f_ **=** 0.51 (S_2_); UPLC (purity >99%): *t*_R_ = 6.89 min. LC–MS (ESI): *m*/*z* calcd for C_17_H_22_F_3_N_3_O_3_S (M + H)^+^, 406.14; found, 406.1. UPLC/HRMS (purity >99%): *t*_R_ = 6.45 min. HRMS (ESI-QTOF): *m*/*z* calcd for C_17_H_22_F_3_N_3_O_3_S (M + H)^+^, 406.1368; found, 406.1445. ^1^H NMR (500 MHz, CDCl_3_) δ: 2.00 (s, 3 H), 3.11–3.19 (m, 2 H), 3.21–3.29 (m, 2 H), 3.31 (s, 3 H), 3.46 (dd, *J* = 9.0, 7.7 Hz, 1 H), 3.59 (dd, *J* = 9.0, 4.9 Hz, 1 H), 3.65–3.75 (m, 2 H), 3.76–3.84 (m, 1 H), 3.87–3.96 (m, 1 H), 5.15 (td, *J* = 7.7, 4.9 Hz, 1 H), 6.55 (br d, *J* = 7.7 Hz, 1 H), 6.98–7.04 (m, 1 H), 7.12–7.18 (m, 2 H), 7.29 (t, *J* = 8.1 Hz, 1 H). ^13^C NMR (126 MHz, CDCl_3_) δ 23.3, 42.1, 45.7, 48.6, 48.8, 49.3, 59.4, 73.2, 118.6, 123.8, 125.3, 129.7 (q, *J* = 308.0 Hz) 127.8, 130.2, 151.5, 169.0, 169.7. Chiral SFC >98% ee (t_R_ = 5.025 min).

#### (*R,S*)-*N*-(3-methoxy-1-oxo-1-(4-(4-((trifluoromethyl)­thio)­phenyl)­piperazin-1-yl)­propan-2-yl)­acetamide (*R,S*)**-51**

White solid. Yield: 91% (1.29 g); mp 132.6–133.7 °C; TLC: R_f_ **=** 0.51 (S_2_); UPLC (purity = 98.78%): *t*_R_ = 6.30 min. LC–MS (ESI): *m*/*z* calcd for C_17_H_22_F_3_N_3_O_3_S (M + H)^+^, 406.14; found, 406.2. ^1^H NMR (500 MHz, CDCl_3_) δ: 2.00 (s, 3 H), 3.22 (br d, *J* = 7.6 Hz, 2 H), 3.29–3.38 (m, 5 H), 3.45 (dd, *J* = 8.9, 8.0 Hz, 1 H), 3.59 (dd, *J* = 8.9, 4.9 Hz, 1 H), 3.65–3.73 (m, 2 H), 3.76–3.85 (m, 1 H), 3.90 (td, *J* = 6.6, 2.6 Hz, 1 H), 5.14 (td, *J* = 7.3, 5.2 Hz, 1 H), 6.54 (br d, *J* = 7.7 Hz, 1 H), 6.81–6.94 (m, 2 H), 7.49–7.54 (m, 2 H). ^13^C NMR (126 MHz, CDCl_3_) δ: 23.3, 42.0, 45.5, 47.8, 48.4, 48.6, 59.4, 73.1, 113.1, 116.0, 129.7 (q, *J* = 308.0 Hz), 138.1, 152.4, 169.0, 169.7.

### Antiseizure Activity—MES, 6 Hz (32/44 mA) and Chimney Tests

#### Animals

The experiments were carried out on male Swiss Albino mice weighing between 22 and 26 g purchased from the Laboratory Animal Breeding Facility (Warsaw, Poland) at the age of 4–5 weeks. They were kept in colony cages under standardized laboratory conditions: natural light–dark cycle 12/12 h, temperature 20–24 °C, air humidity 45–65% and free access to tap water and food (chow pellets). The animals were left to adapt under laboratory conditions for 7 days. All procedures involving animals and their care were performed in accordance with the current European Community and Polish legislation on animal experimentation. The studies were carried out under experimental protocols approved by the Local Ethics Committee in Lublin (67/2022, 68/2023 and 80/2024) in accordance with the European Communities Council Directive of 22 September 2010 (2010/63/EU). All efforts were made to minimize animal suffering and to use only the number of animals necessary to produce reliable scientific data according to the 3Rs rule.

#### Antiseizure Activity and Acute Neurotoxicity

In the preliminary screening phase, four mice were randomly allocated to each treatment group, with every animal used only once. For the determination of ED_50_ and TD_50_ values, four groups of eight mice each received different doses of the tested compounds. Protective indixes (PIs) for the investigated compounds and the reference ASMs were calculated as the ratio of the TD_50_ obtained in the chimney test to the corresponding ED_50_ derived from the MES or 6 Hz (32 mA or 44 mA) assays (PI = TD_50_/ED_50_). The PI provides an estimate of the therapeutic window for each compound.

All compounds were suspended in a 1% aqueous Tween 80 solution and administered intraperitoneally in a single dose of 10 mL/kg. Fresh suspensions were prepared immediately prior to each experimental session. The detailed *in vivo* procedures are described elsewhere: the maximal electroshock seizure test (MES),^64^ the 6 Hz psychomotor seizure model (32 mA and 44 mA),^34,65^ the chimney test.^66^ The reference ASMs were purchased from commercial suppliers: VPA (Sigma-Aldrich, St. Louis, MO, USA), LCS and LEV (UCB Pharma, Braine l’Alleud, Belgium).

### *In Vitro* ADME-Tox Studies

All assays and protocols used for evaluation of compounds **(*R*)-** **46** and **(*S*)-** **46** ADME-Tox parameters were described previously, excluding reactive metabolites formation testing.^25,48,67–70^

#### Permeability

Precoated PAMPA Plate System Gentest was provided by Corning, (Tewksbury, MA, USA) and the assay was performed according to the procedures provided by Corning (Tewksbury, MA).

#### Protein binding Analyses

PPB studies were performed with use of the commercial TRANSIL^XL^ PPB Assay (Sovicell, Leipzig, Germany) containing different concentrations of human serum albumin (HSA) and α1-acid glycoprotein (AGP) mixed in physiological ratio of 24:1. The PPB parameters of tested compounds and highly bound reference warfarin were determined and calculated using the protocol and equations provided by the manufacturer.

#### Pharmacokinetics

The PK *in vitro* parameters of enantiomers were tested in mouse liver microsomes MLMs (Sigma-Aldrich, St. Louis, MO, USA). The *t*_1/2_ values and intrinsic clearances (CL_int_) were calculated by using the protocols and formulas proposed by Obach.^71^ The microsomal protein/g of liver weight was considered as 45 mg and the liver weight/kg of body 87 g in mouse. The UPLC and mass spectra (LC–MS) were obtained on Waters ACQUITY TQD system (Waters, Milford, CT, USA) with the MS-TQ detector and UV–vis-DAD eλ detector. The metabolic biotransformation pathways were predicted *in silico* by MetaSite 8.0.1 provided by Molecular Discovery Ltd. (Hertfordshire, UK).

#### CYP Assays

The luminescent CYP3A4 P450-Glo and CYP2D6 P450-Glo (Promega, Madison, WI, USA) were used for determination of drug–drug interactions. All tests were performed following in details procedures provided by manufacturer.

#### Neurotoxicity and Hepatotoxicity

The classic MTS tests determining cells viability (CellTiter 96 AQueous Non-Radioactive Cell Proliferation Assay, Promega, Madison, WI, USA) were done in hepatoma HepG2 and SH-SY5Y cell lines from ATCC (American Type Culture Collection, Manassas, VA, USA).

#### Phospholipidosis

The HepG2 cells were treated by 10 μM and 50 μM of phospholipidosis inductor Verapamil and by 50 μM and 100 μM of **(*R*)-46** and **(*S*)-46**. The cells were stained next by LYSO-ID Red cytotoxicity kit containing Dual Color Detection Reagent (Enzo Biochem, Inc. NY, USA). The red fluorescent lysosomal signal and the blue nuclear signal were registered by microscope Leica DMi8 (Leica, Wetzlar, Germany).

#### Reactive Metabolites Formation

The assay was performed according to the method described previously in the literature.^39^ HepG2 cells were pretreated fist by d,l-buthionine-S,R-sulfoximine (BSO) (Sigma-Aldrich, St. Louis, MO, USA), which is an inhibitor of γ-glutamylcysteine synthetase causing the intracellular GSH depletion. This step was aimed at reducing the physiological GSH level and increase the sensitivity of a cell-based assay for detecting GSH level changes in the presence of RMs. Next, the cells were exposed on 100 μM of Tolcapone (the positive control with confirmed RMs) and 100 μM of **(*R*)-46** and **(*S*)-46**. Finally, the GSH level was evaluated with use of luminescence assay GSH-Glo (Promega, Madison, WI, USA).

#### HLMs metabolic Stability

The metabolic stability assay was performed on human liver microsomes purchased from Sigma-Aldrich (St. Louis, MO). All tests were performed following in details procedures provided by manufacturer.

#### Protein Binding (Plasma CD-1 Mouse) and Tissue Binding (Brain, CD-1 Mouse)

Was carried out commercially in Eurofins Laboratories (St Charles, MO, USA) according to literature.^72^

#### Statistics

The statistical significances in all performed ADME-Tox experiments were calculated by GraphPad Prism 8.0.1 (one-way ANOVA, followed by Bonferroni’s multiple comparison test).

### Seizure Threshold Tests and PTZ Kindling in Mice

#### Animals

The experiments were carried out on male CD-1 mice purchased from the Faculty of Pharmacy, Jagiellonian University Medical College (Krakow, Poland) at the age of 4–5 weeks. The animals were kept in groups of 7–8 under controlled laboratory conditions (temperature 21–24 °C, relative humidity 45–65%, and artificial 12-h light/dark cycle) with free access to food and tap water. Housing and experiment procedures were conducted in compliance with Polish regulations and the European Union Directive of 22 September 2010 (2010/63/EU) regarding the protection of animals used for scientific purposes. Experimental protocol was approved by the Local Ethics Committees for Experiments on Animals in Lublin, Poland (approval no. 21/2024).

#### Seizure Threshold Tests

For acute seizure tests, **(*R*)-46** were suspended in a 1% solution of Tween 80 and administered *i.p.,* 30 min before the tests. VPA (as sodium salt, Sigma-Aldrich) was dissolved in normal saline and administered 15 min prior testing. The MEST, the 6 Hz seizure threshold test, and the *iv* PTZ seizure threshold test were performed according to the method described in detail elsewhere.^73^ In addition, the grip strength test and the chimney test were carried just before the seizure thresholds tests, as described previously.^74^

#### PTZ-Induced Kindling

The kindling procedure was performed as described in detail in our previous studies.^75,76^ Briefly, seizures were induced three times a week by administering PTZ at 40 mg/kg (*i.p.*), 30 min after administration of **(*R*)-46**, VPA, or vehicle. VPA and PTZ were dissolved in saline, whereas **(*R*)-46** was suspended in 1% Tween 80. **(*R*)-46**, VPA, and vehicle were given *i.p.* every 24 h. Seizure severity was scored using the modified Racine’s scale.^75,76^ The experimental groups were as follows: (a) 1% Tween + saline (nonkindled control group), (b) 1% Tween + PTZ (PTZ-kindled control group); (d) VPA at 150 mg/kg + PTZ (positive control group); (e)–(f) **(*R*)-46** at 30 and 50 mg/kg + PTZ. Twenty-four h after the last PTZ injection, animals were euthanized and their brains were collected for anlaysis of cytokines and amino acids in the hippocampus. Following euthanasion, hippocampi were immedietly dissected from the brains, frozen, and stored at −80 °C until assays.

### Analysis of Cytokines and Amino Acids in the Hippocampus

#### Protein Isolation

Frozen hippocampal tissues (typically 10–20 mg) were weighed and then homogenized in ice-cold lysis buffer (500 μL of lysis buffer per 10 mg of tissue) using a Teflon-glass homogenizer in lysis buffer provided in the Bio-Plex Cell Lysis Kit (#171304011, Bio-Rad) until a homogeneous suspension was obtained (typically 10–15 strokes). All steps were performed on ice to prevent protein degradation. Following homogenization, the lysates were centrifuged at 4500*g* for 20 min at 4 °C to pellet cellular debris. The supernatant, containing the soluble protein fraction, was carefully transferred to a new microcentrifuge tube. Protein concentration in the obtained lysates was determined using the BCA Protein Assay Kit (Thermo Fisher Scientific, Cat# 23225) according to the manufacturer’s instructions.

#### Measurement of Cytokines in the Hippocampus Using Luminex

To measure pro-inflammatory cytokines in the hippocampus, a Bio-Plex Pro Mouse Cytokine Th17 Panel A 6-Plex (Cat# M6000007NY, Bio-Rad) was used, following the manufacturer’s instructions. For brain tissue homogenization, the Bio-Plex Cell Lysis Kit (Cat# 171304011) was utilized. Lysates were diluted to a final protein concentration of 1000 mg/mL in the Bio-Plex Assay Diluent for subsequent Luminex analysis. Briefly, diluted magnetic beads were added to the assay plate and washed with wash buffer. Brain tissue homogenates and standards were then added to the wells and incubated at room temperature with shaking at 850 rpm for 30 min. Following incubation, the plates were washed three times with wash buffer. The detection antibody was added, and plates were incubated again for 30 min at room temperature with shaking at 850 rpm. After another three washes, streptavidin-phycoerythrin (SA-PE) was added and incubated for 10 min under the same conditions. Plates were then washed three more times, and the beads were resuspended in assay buffer for acquisition. Cytokine levels were measured using the Luminex FLEXMAP 3D system (Luminex Corp.).

#### Measurement of Amino Acids in the Hippocampus

A 400 μL volume of 1% acidified methanol solution (methanol: acetic acid) was added to hippocampal samples for amino acid extraction. The samples were sonicated for two cycles of 30 s at 30% amplitude and subsequently centrifuged at 10,000 rpm for 10 min. The supernatant was collected and dried under reduced pressure.

The analytical protocol was adapted from the method described by Shimbo et al.^77^ dried sample extracts were reconstituted in 200 μL of 200 mM borate buffer (pH 8.5), followed by liquid–liquid extraction with 300 μL of dichloromethane. After thorough mixing, samples were centrifuged at 13,000*g* for 10 min to remove nonpolar interferences. A 30 μL aliquot of the aqueous phase was collected for derivatization. Fifteen μL of 3-aminopyridyl-*N*-hydroxysuccinimidyl carbamate (APDS) solution and a mixture of isotopically labeled internal standards were added. The reaction volume was completed to 600 μL with borate buffer, and the mixture was incubated at 60 °C for 20 min to ensure complete derivatization. The resulting solutions were filtered, and a 2 μL volume was injected into a Kinetex C18 column (1.7 μm, 100 Å, 150 × 2.1 mm) for separation.

Chromatographic separations were achieved using a binary gradient system composed of 200 mM ammonium formate (mobile phase A) and acetonitrile (mobile phase B), at a flow rate of 0.3 mL/min and a column temperature of 40 °C. The gradient profile was as follows: 2% to 6% B in the first 0.10 min, held at 6% B until 2.0 min, increased to 13% B by 5.0 min, maintained until 6.0 min, then ramped to 30% B by 8.0 min and to 60% B by 8.10 min. This composition was held until 9.10 min, followed by a re-equilibration to 2% B over 0.10 min and a 5 min postrun at 2% B.

Mass spectrometric detection was carried out using a Shimadzu Nexera XR LC system coupled to a Shimadzu LCMS-8030 triple quadrupole mass spectrometer equipped with an electrospray ionization (ESI) source operating in positive ion mode. The ionization parameters were as follows: spray voltage, 4500 V; nebulizing gas (N_2_), 1.5 L/min; drying gas (N_2_), 15 L/min; desolvation line temperature, 250 °C. Data acquisition was performed in multiple reaction monitoring (MRM) mode, selecting precursor ions of the APDS-labeled amino acids and their common fragment at *m*/*z* 121.1. Transitions were optimized to maximize signal intensity for quantification.

Identification of amino acids was based on retention times and *m*/*z* values in comparison to a reference standard mixture (type B (High range) and type AN (High range), FUJIFILM/Wako Pure Chemical Corporation). Quantification was conducted using calibration curves constructed from external standards, determining the linear dynamic range, limit of detection (LOD), and limit of quantification (LOQ). The internal standards employed included l-Asp-^13^C_4_,^15^N, l-val-^13^C_5_, l-pro-^13^C_5_,^15^N, and norvaline.

### Antinociceptive Activity

#### General Information and Statistical Analysis

Adult male CD-1 mice (18–25 g) were sourced from the accredited animal facility at the Jagiellonian University Medical College (Kraków, Poland). Animals were randomly assigned to experimental groups comprising 8–10 individuals, with each mouse used in a single behavioral test to avoid cross-experiment interference. All behavioral observations were conducted by trained experimenters under standardized conditions. Immediately after testing, animals were euthanized by cervical dislocation to minimize suffering.

All procedures were carried out in accordance with Polish legal regulations and the European Union Directive 2010/63/EU on the protection of animals used for scientific purposes. Experimental protocols were approved by the Local Ethics Committee in Kraków (approval numbers: 104/2015, 124/2015, 559/2021 and 614/2022) and complied with guidelines issued by the International Council for Laboratory Animal Science (ICLAS).

The compound **(*R*)-46** was suspended in a 1% aqueous solution of Tween 80 and administered *i.p*. Thirty min prior to testing. Control animals received an equivalent volume of the vehicle (1% Tween 80 in water, *i.p.*) to serve as a negative control.

The means ± standard error of the mean (SEM) was used to present data. The data was analyzed using GraphPad Prism Software (v.5). One-way analysis of variance (ANOVA) and the post hoc Dunnett’s multiple comparison test or two-way ANOVA (when appropriate) were used to calculate statistically significant differences between groups. The significance threshold was established at *p* < 0.05. The ED_50_ values were statistically determined with 95% confidence limits using the log-probit method.

#### Formalin Test

Mice received an *i.p.* injection of the test compound or vehicle. Thirty min later, 20 μL of a 2.5% formalin solution was administered into the plantar surface of the right hind paw. Immediately following the injection, each animal was placed individually in a transparent glass observation chamber. Nociceptive behavior—specifically, the time spent licking or biting the injected paw—was recorded during two distinct observation periods: the early phase (0–5 min) reflecting acute pain, and the late phase (15–30 min) corresponding to inflammatory pain.^78^

#### Capsaicin Test

Mice were injected *i.p.* with either the test compound or vehicle. After a 30 min interval, 1.6 μg of capsaicin dissolved in 20 μL of 0.9% saline was administered into the plantar surface of the right hind paw. Immediately following the injection, each mouse was placed in an individual glass container and observed for 5 min. The duration of nociceptive behavior—defined as time spent licking or biting the injected paw—was recorded.^78^

#### OXPT-Induced Pain

Peripheral neuropathy was induced by a single *i.p.* injection of OXPT at a dose of 10 mg/kg, prepared in a 5% glucose solution (Polfa Kutno, Poland). Mechanical sensitivity was evaluated using an electronic von Frey apparatus (Bioseb, France), equipped with a single flexible filament that delivered an increasing force (0–10 g) to the plantar surface of the hind paw. A nocifensive response—defined as paw withdrawal—was automatically detected and the corresponding force recorded as the mechanical pain threshold. Measurements were taken at three time points: prior to OXPT administration (baseline), and at 3 h and 7 days postinjection. A significant reduction in mechanical threshold was considered indicative of tactile allodynia. Only animals meeting this criterion were included in the pharmacological testing phase. On the test day, mice were placed individually in wire mesh-bottomed chambers and allowed to habituate for 60 min. Following habituation, the mechanical threshold was assessed three times with at least 30 s between trials. The average of these three measurements was used as the baseline. Mice exhibiting allodynia were then pretreated *i.p.* with the test compound or compound combinations. Thirty min post-treatment, the mechanical threshold was reassessed using the same procedure.^78^

#### STZ-Induced Pain

Diabetic neuropathy was induced in mice via a single *i.p.* injection of STZ at a dose of 200 mg/kg. STZ was dissolved in a 5% glucose solution to a final concentration of 10 mg/mL. This treatment results in the development of a diabetic neuropathy model characterized by neuropathic pain, manifested as a decreased mechanical pain threshold in response to tactile stimulation. To evaluate the effect of the test compound on mechanical allodynia, the von Frey test was performed using the same procedure described for the OXPT-induced neuropathy model. The experimental timeline included two main stages. On Day 1, a baseline nociceptive response was recorded to establish the initial mechanical pain threshold, followed by a single *i.p.* injection of STZ. On Day 21, blood glucose levels were measured to identify hyperglycemic animals suitable for further evaluation. Subsequently, nociceptive responses were reassessed to confirm the development of mechanical allodynia. After this, the test compound was administered *i.p*., and the nociceptive response was measured again 30 min later to determine the compound’s potential to alleviate allodynia.^79^

#### Influence on mice Spontaneous Locomotor Activity

Spontaneous locomotor activity was evaluated using activity cages (Activity Cage 7441; Ugo Basile, Italy). These devices consist of specially designed enclosures measuring 40 × 40 × 30 cm, equipped with infrared light sources and photodetectors positioned on opposite sides. Movement across the infrared beam was detected as a signal interruption, which was automatically recorded by a connected computer as a unit of locomotor activity. The test compound was administered *i.p*., and 30 min later, each mouse was individually placed in the activity cage. Locomotor activity was then recorded continuously over a 30 min period. The total number of infrared beam interruptions served as the quantitative measure of spontaneous activity. Following each session, animals were removed from the apparatus, and the base of each cage was thoroughly cleaned with 70% ethanol to eliminate scent cues and prevent behavioral interference in subsequent trials.^80^

### Pharmacokinetic *In Vivo* Study

#### Animals

Male CD-1 mice sourced from the accredited animal facility at the Jagiellonian University Medical College (Kraków, Poland), weighting 27–33 g housed in conditions of the constant temperature with the 12:12 h light–dark cycle with free access to food and water were used in this study. The investigated compound was suspended in 1% Tween in water for injection (Polpharma, Poland) and administered *i.p.* at a dose of 25 and 50 mg/kg. In addition, the compound was administered orally (*p.o.*) at a dose of 25 mg/kg and intravenously (*i.v.*) at a dose of 10 mg/kg after dissolving in the mixture of DMSO/PEG400/water (1:2:7 *v/v/v*) The mice were sacrificed by decapitation under isoflurane anesthesia. Blood samples were collected at 5, 15, 30, 60, 120, 240, and 480 min after dosing and brains were harvested at the same time points. Blood was allowed to clot at room temperature for 20 min and serum was separated by centrifugation at 8000*g* (Eppendorf MiniSpin centrifuge, Germany) for 10 min. The samples were stored at −80 °C until analysis. All animal procedures were approved by the First Ethical Committee on Animal Experimentation in Kraków (license no. 270/2019).

#### Analytical Method

The levels of compound **(*R*)-46** in mouse serum and brain tissue were quantified using a liquid chromatography-tandem mass spectrometry (LC–MS/MS) technique. Mouse brain samples were homogenized in distilled water at a 1:4 weight-to-volume ratio using a ULTRA-TURRAX T10 basic homogenizer (IKA, Germany). For sample preparation, 50 μL of either brain homogenate or serum was mixed with 150 μL of 0.1% formic acid in acetonitrile containing an internal standard (IS). The mixture was agitated for 10 min using a shaker (IKA Vibrax VXR, Germany), followed by centrifugation at 8000*g* for 5 min (Eppendorf miniSpin centrifuge, Germany). The resulting supernatants were transferred to autosampler vials and 1 μL was injected into the LC–MS/MS system. The autosampler temperature was maintained at 15 °C. Chromatographic separation was carried out on an Exion LC AC HPLC system (Sciex, USA) equipped with a Hypersil Gold C18 column (3 × 50 mm, 5 μm; Thermo Scientific, USA). The oven temperature was set at 30 °C. A gradient elution was employed using mobile phase A (0.1% formic acid in acetonitrile) and mobile phase B (0.1% formic acid in water). The gradient started with 95% B and 5% A for the first 2 min, transitioned linearly to 5% B over the next 2 min, held isocratically for 2 min, then rapidly returned to 95% B in 0.1 min, and remained at that composition for the remainder of the 10 min run. The flow rate was maintained at 0.4 mL/min. Detection was performed using a QTRAP 4500 mass spectrometer (Sciex, USA) with an electrospray ionization (ESI) source. Instrument parameters were optimized for sensitivity at unit resolution. The ion source temperature was set at 450 °C and the spray voltage was 5500 V. Curtain gas (CUR) was maintained at 40 psi and the collision gas (CAD) was set to medium. Multiple reaction monitoring (MRM) was conducted in positive ion mode, tracking transitions from *m*/*z* 390 to 247 (CE = 23 eV) and *m*/*z* 390 to 144 (CE = 21 eV) for **(*R*)-46**, and from *m*/*z* 436 to 207 (CE = 42 eV) for valsartan, which served as IS.

A stock solution of **(*R*)-46** was prepared in DMSO at 1 mg/mL and working solutions were diluted in methanol. Calibration samples were prepared by spiking 45 μL of matrix (plasma or brain homogenate) with 5 μL of standard solution at concentrations ranging from 0.01 to 200 μg/mL, followed by vortexing for 10 s. For serum, two separate calibration curves were generated to cover low and high concentration ranges. Samples in the upper range were diluted 10-fold with the precipitation solvent. Calibration curves were constructed by plotting the analyte-to-IS peak area ratio against analyte concentration, using weighted (1/x^2^) linear regression. Quantification ranges were 0.001–5 μg/mL and 0.1–20 μg/mL for serum, and 0.005–25 μg/g for brain tissue. Samples exceeding the upper quantification limit were diluted 10-fold with blank matrix. The method met FDA criteria for accuracy and precision in bioanalytical validation. No significant matrix effects or stability issues were observed during routine analysis. Data were processed using Analyst software version 1.7.

#### Pharmacokinetic Data Analysis

To assess pharmacokinetic parameters after intravenous and extravascular administration the noncompartmental approach was used. The maximum concentration (*C*_max_) and the time to reach maximum concentration (*t*_max_) in serum and brain after *i.p*. and oral dosing were obtained directly from the concentration vs time data. The initial serum concentration (*C*_0_) after *i.v.* injection was estimated by back-extrapolating the serum concentration–time curve to time zero on a semi-logarithmic plot. The terminal elimination rate constant (λ_*z*_) was estimated by linear regression and terminal half-life (t_0.5λz_) was calculated as ln2/λ_*z*_. Based on serum concentration vs time data, clearance (CL/F) and volume of distribution based on the terminal phase (V_*z*_/F) were also calculated. The first parameter was estimated as Dose/AUC_0‑∞_, where AUC_0‑∞_ is the area under concentration vs time curve from the time of dosing to infinity obtained by the linear trapezoidal rule. The extrapolated terminal area was defined as C_n_/λ_*z*_, where C_n_ is the last data point. *V*_*z*_/F was calculated according to the equation: D/(λ_*z*_·AUC_0‑∞_), where F is fraction absorbed, and mean residence time (MRT) was calculated as AUMC_0‑∞_/AUC_0‑∞_, where AUMC_0‑∞_ is the area under the first moment curve from the time of dosing to infinity. Absolute bioavailability (F_a_) was calculated as follows: F_a_ = AUC_i.p.(or p.o.)_·D_i.v._/(AUC_i.v._·D_i.p.(or p.o.)_). Concentration vs time data after *i.v.* administration were also analyzed using the compartmental approach in ADAPT5 (BMSR, CA, USA).

### Binding/Functional Studies

Binding/functional studies were carried out commercially in Eurofins Laboratories (Poitiers, France) and Eurofins Panlabs Discovery Services Taiwan, Ltd. (New Taipei City, Taiwan) using testing procedures reported previously (for details see Table S5).

Analysis of effects on sodium channels endogenously expressed in N1E-115 neuroblastoma cells and CHO cells stably expressing human Nav1.5 channel was carried out commercially in B’SYS GmbH (Witterswil, Switzerland). The whole-cell patch-clamp technique was used to investigate the effect of one concentration of **(*R*)-46** and LCS on sodium channels expressed in N1E-115 neuroblastoma cells and CHO cells stably expressing human Nav1.5 channel in the resting, fast and slow inactivated state using standard procedures reported in Web site https://bsys.ch/ion-channel-screening/patch-clamping and reported previously.^81^

## Supplementary Material





## Notes

Synthetic procedure, physicochemical and spectral data for the starting amines, *in silico* studies, antiseizure screening data, effect of on neuromuscular strength and motor coordination in mice effect on the concentrations of amino acids in the hippocampus of kindled mice, binding/functional assay information, *in silico* metabolic biotransformations pathways, metabolism data (ion fragment analysis and structures of probable metabolites), influence on CYP3A4 and CYP2D6 activity, PK profiles, rate/state dependence effects on sodium channels, ^1^H NMR, ^13^C NMR spectra for target compounds and HRMS spectra for selected molecules, chiral SFC traces (PDF)
Molecular formula strings of the final compounds together with biological data (CSV)

## Abbreviations Used

ADME-Tox: absorption, distribution, metabolism, excretion, toxicity
ASM: antiseizure medication
CAD: cationic amphiphilic drugs
CGRP: calcitonin gene-related peptide
CiPA: comprehensive in vitro proarrhythmia assay
CNS: central nervous system
DCC: dicyclohexylcarbodiimide
DCM: dichloromethane
DDIs: drug–drug interactions
DILI: drug-induced liver damage
DOX: doxorubicin
DRE: drug-resistant epilepsy
ETX: ethosuximide
GABA: gamma-aminobutyric acid
GSH: glutathione
HRMS: high resolution mass spectroscopy
Six Hz: six-Hertz seizure test
IFN-γ: interferon gamma
IL: interleukin
LC-MS: liquid chromatography–mass spectrometry
LCS: lacosamide
LEV: levetiracetam
MeCN: acetonitrile
MES: maximal electroshock seizure test
MEST: maximal electroshock seizure threshold
MeOH: methanol
MLMs: mouse liver microsomes
OXPT: oxaliplatin
PAMPA: parallel artificial membrane permeability assay
PD: Parkinson’s disease
PI: protective index (TD_50_/ED_50_)
PT: pretreatment time
PTZ: pentylenetetrazole
RM: reactive metabolites
SAR: structure–activity relationship
*sc*PTZ: subcutaneous pentylenetetrazole seizure test
SFC: supercritical fluid chromatography
STZ: streptozotocin
TFA: trifluoroacetic acid
TNF-α: tumor necrosis factor alpha
UPLC: ultraperformance liquid chromatography
VPA: valproic acid.
