# Supplementary material for: Novel 3‑Methoxypropanamide Derivatives as Potential Antiseizure and Antinociceptive Agents: Experimental Evidence from In Vitro and In Vivo Studies
Source: J Med Chem. 2026 May 1;69(9):10019–45. doi: 10.1021/acs.jmedchem.5c02093 (PMC13308885; doi:10.1021/acs.jmedchem.5c02093)

Supplementary Information

Novel 3-Methoxypropanamide Derivatives as Potential Antiseizure and Antinociceptive Agents: Experimental Evidence from In Vitro and In Vivo Studies

Marcin Jakubiec [†*], Mirosław Zagaja [‡], Katarzyna Socała [⁑], Michał Abram [†], Szczepan Mogilski [⊥], Gniewomir Latacz [▲,^], Małgorzata Szafarz [◊], Joanna Szala-Rycaj [‡], Joanna Karnafał- Ziembla [▲], Maja Kudrycka [^], Nikola Gapińska [⁑,♦], Alan González Ibarra [#], Justyna Turek [■], Łukasz Gąsior [■], Bernadeta Szewczyk [■], Elżbieta Wyska [◊], Marta Andres-Mach [‡], Piotr Wlaź [⁑], and Krzysztof Kamiński [†,]

[†] Department of Medicinal Chemistry, Faculty of Pharmacy, Jagiellonian University Medical College, Medyczna 9, 30-688 Cracow, Poland

[‡] Department of Experimental Pharmacology, Institute of Rural Health, Jaczewskiego 2, 20-090 Lublin, Poland

[⁑] Biomedical Research Laboratory, Institute of Biological Sciences, Maria Curie-Skłodowska University, Akademicka 19, 20–033 Lublin, Poland

[⊥] Department Pharmacodynamics, Faculty of Pharmacy, Jagiellonian University Medical College, Medyczna 9, 30-688 Cracow, Poland

[▲] Department of Chemical Technology and Biotechnology of Drugs, Faculty of Pharmacy, Jagiellonian University Medical College, Medyczna 9, 30-688 Cracow, Poland

[^] Pharmacokinetics and Preliminary Toxicological Analysis Laboratory, Center for the Development of Therapies for Civilization and Age-Related Diseases, Jagiellonian University Medical College, Skawińska 8, 30-688 Krakow, Poland

[◊] Department of Pharmacokinetics and Physical Pharmacy, Faculty of Pharmacy, Jagiellonian University Medical College, Medyczna 9, 30-688 Cracow, Poland

[♦] Doctoral School of Quantitative and Natural Sciences, Maria Curie-Skłodowska University, Weteranów 18, 20-038 Lublin, Poland

[#] Department of Molecular Biology, Institute of Biological Sciences, Maria Curie-Skłodowska University, Akademicka 19, 20-033 Lublin, Poland

[■] Department of Neurobiology, Maj Institute of Pharmacology, Polish Academy of Sciences, Smętna 12, 31-343 Kraków, Poland

*__Corresponding author__:

Marcin Jakubiec, Jagiellonian University Medical College, Faculty of Pharmacy, Department of Medicinal Chemistry, Medyczna 9, 30-688 Kraków, Poland.

E-mail: marcin.jakubiec@uj.edu.pl

Table of contents

The procedure for synthesis, physicochemical and spectra data for A1-A9. 3

The procedure for synthesis, physicochemical and spectra data for A10-A18. 4

Table S1. Parameters calculated according to Lipinski rule, Veber rule, and CNS MPO.. 6

Table S2. Antiseizure activity screening data for compounds **(*R*,*S*)-35**–**(*R*,*S*)-51** in mice *i.p.* (100 mg/kg). 8

Table S3. Effect of **(*R*)-46** on neuromuscular strength and motor coordination in mice. 9

Table S4. Effects of **(*R*)-46** at a dose of 50 mg/kg on the concentrations of amino acids in the hippocampus of kindled mice. 10

Table S5. Radioligand binding and functional assays. 11

Explanation of low TQI according to manufacturer's manual (troubleshooting) 12

Fig. S1. The MetaSite 6.0.1. software prediction of the most probable sites of **(*R*)-46** and **(*S*)-46**.. 12

Fig. S2. The MetaSite 6.0.1. software prediction of the most probable hydroxylations/oxydations of **(*R*)-46**. 13

Fig. S3. MS of **(*R*)-46** and its metabolites after 120 min incubation with MLMs. 14

Fig. S4. MS of **(*S*)-46** and its metabolites after 120 min incubation with MLMs. 15

Fig. S5. The influence on CYP3A4 activity. 16

Fig. S6. The influence on CYP2D6 activity. 16

Fig. S7. Microscopy images of not treated HepG2 cells and exposed for 24 h on 10 µM and 50 µM of verapamil. 17

Fig. S8. Microscopy images of HepG2 cells exposed for 24 h on 50 µM, 100 µM of **(*R*)-46** and 50 µM, 100 µM of **(*S*)-46**. 18

Fig. S9. Effects of **(*R*)-46** at a dose of 50 mg/kg on the concentrations of inflammatory markers in the hippocampus of kindled mice. 19

Fig. S10. Mean (±SD) serum and brain concentrations of **(*R*)-46** after *i.p.* administration of this compound at two doses 25 mg/kg and 50 mg/kg to mice 20

Fig. S11. Mean (±SD) serum and brain concentrations of **(*R*)-46** after *p.o.* and *i.v.* administration of this compound to mice. 21

Fig. S12. Rate dependence effect of control, Carbamazepine and **(*R*)-46** on voltage gated sodium channels expressed in N1E-115 cells. 22

Fig. S13. State dependence effect of control, LCS and **(*R*)-46** on voltage gated sodium channels expressed in N1E-115 cells. 23

Fig. S14. Effect of control, LCS and **(*R*)-46** and Propafenone on voltage gated sodium channel subunit Nav1.5 expressed in CHO cells. 23

References 25

Chiral SFC traces 26

HRMS traces 29

^{1}H NMR and ^{13}C NMR spectra for the final compounds 41

The procedure for synthesis, physicochemical and spectra data for A1-A9.

The starting (non-commercial) Boc-derivatives of 4-aryl-piperazine were obtained in *N*-arylation reaction according to **Scheme 1**. The appropriate aryl bromide (10 mmol, 1 eq), Pd_2dba_3 (0.37 g, 0.4 mmol, 0.04 eq), BINAP (0.37 g, 0.59 mmol, 0.06 eq), sodium tert-butoxide (1.35 g, 14 mmol, 1.4 eq), and Boc-piperazine (3.74 g, 20 mmol, 2 eq) were suspended in an inert gas (nitrogen) atmosphere in 50 mL of dry toluene. Next, the reaction mixture was refluxed for 12 h, subsequently cooled, and filtered through Celite 545 Merck (Darmstadt, Germany), and then concentrated under reduced pressure. The Boc protected amines **A1**−**A9** were purified by column chromatography using the following developing systems: S_1.

***Tert*-butyl 4-(3,5-dichlorophenyl)piperazine-1-carboxylate (A1)**. Yellow oil, yield 65% (2.15 g); TLC: R_f = 0.83 (S_1); UPLC (purity > 99%): t_R = 9.32 min. LC-MS (ESI): *m/z* calcd for $C_{15}H_{20}Cl_2N_2O_2$ $(M+H)^+$ 331.09, found 331.1.

***Tert*-butyl 4-([1,1'-biphenyl]-3-yl)piperazine-1-carboxylate (A2)**. Yellow oil, yield 64% (2.17 g); TLC: R_f = 0.70 (S_2); UPLC (purity = 90.8%): t_R = 8.95 min. LC-MS (ESI): *m/z* calcd for $C_{21}H_{26}N_2O_2$ $(M+H)^+$ 339.23, found 339.2.

***Tert*-butyl 4-([1,1'-biphenyl]-4-yl)piperazine-1-carboxylate (A3)**. Yellow oil, yield 62% (2.13 g); TLC: R_f = 0.70 (S_2); UPLC (purity > 99%): t_R = 8.88 min. LC-MS (ESI): *m/z* calcd for $C_{21}H_{26}N_2O_2$ $(M+H)^+$ 339.23, found 339.2.

***Tert*-butyl 4-(3-phenoxyphenyl)piperazine-1-carboxylate (A4)**. Yellow oil, yield 67% (2.37 g); TLC: R_f = 0.77 (S_2); UPLC (purity = 92.3%): t_R = 9.07 min. LC-MS (ESI): *m/z* calcd for $C_{21}H_{26}N_2O_3$ $(M+H)^+$ 355.20, found 355.3.

***Tert*-butyl 4-(4-phenoxyphenyl)piperazine-1-carboxylate (A5)**. Yellow oil, yield 63% (2.29 g); TLC: R_f = 0.77 (S_2); UPLC (purity = 93.7%): t_R = 9.12 min. LC-MS (ESI): *m/z* calcd for $C_{21}H_{26}N_2O_3$ $(M+H)^+$ 355.20, found 355.3.

***Tert*-butyl 4-(3-(trifluoromethoxy)phenyl)piperazine-1-carboxylate (A6)**. Yellow oil, yield 68% (2.36 g); TLC: R_f = 0.72 (S_1); UPLC (purity > 99%): t_R = 8.73 min. LC-MS (ESI): *m/z* calcd for $C_{16}H_{21}F_3N_2O_3$ $(M+H)^+$ 347.16, found 347.2.

***Tert*-butyl 4-(4-(trifluoromethoxy)phenyl)piperazine-1-carboxylate (A7)**. Yellow oil, yield 65% (2.31 g); TLC: R_f = 0.72 (S_1); UPLC (purity = 94.7%): t_R = 8.62 min. LC-MS (ESI): *m/z* calcd for $C_{16}H_{21}F_3N_2O_3$ $(M+H)^+$ 347.16, found 347.2.

***Tert*-butyl 4-(3-((trifluoromethyl)thio)phenyl)piperazine-1-carboxylate (A8)**. Yellow oil, yield

71% (2.57 g); TLC: R_f = 0.74 (S_1); UPLC (purity = 91.2%): t_R = 9.14 min. LC-MS (ESI): *m/z* calcd for $C_{16}H_{21}F_3N_2O_2S$ $(M+H)^+$ 363.13, found 363.2.

***Tert*-butyl 4-(4-((trifluoromethyl)thio)phenyl)piperazine-1-carboxylate (A9)**. Yellow oil, yield 70% (2.55 g); TLC: R_f = 0.74 (S_1); UPLC (purity = 93.8%): t_R = 9.02 min. LC-MS (ESI): *m/z* calcd for $C_{16}H_{21}F_3N_2O_2S$ $(M+H)^+$ 363.13, found 363.2.

The procedure for synthesis, physicochemical and spectra data for A10-A18.

The solution of **A1**–**A9** (5 mmol, 1 eq) in DCM (15 mL) was treated with TFA (1.71 g, 15 mmol, 3 eq) and stirred at room temperature for 3 h. Afterwards, the organic solvents were evaporated to dryness. The resulting oil residue was dissolved in water (20 mL), and then 25% ammonium hydroxide was carefully added to pH = 8. The aqueous layer was extracted with DCM (3 × 20 mL), dried over Na_2SO_4, and concentrated to give **A10**–**A18** as yellow or bronze oils. Non-commecial amines **A10**–**A18** were used as substrates for the next reactions without purification. The synthetic pathway is shown in **Scheme 1**.

1-(3,5-Dichlorophenyl)piperazine (A10). Yellow oil, yield 97% (1.21 g); TLC: R_f = 0.48 (S_3); UPLC (purity > 99%): t_R = 3.99 min. LC-MS (ESI): *m/z* calcd for $C_{10}H_{12}Cl_2N_2$ $(M+H)^+$ 231.04, found 231.0. ^1H NMR (500 MHz, $CDCl_3$) δ 2.02–2.18 (m, 1 H, piperazine) 2.97–3.00 (m, 4 H, piperazine), 3.11–3.14 (m, 4 H, piperazine), 6.72 (s, 2 H, ArH), 6.77 (s, 1 H, ArH).

1-([1,1'-Biphenyl]-3-yl)piperazine (A11). Yellow oil, yield 95% (1.13 g); TLC: R_f = 0.52 (S_3); UPLC (purity > 99%): t_R = 4.39 min. LC-MS (ESI): *m/z* calcd for $C_{16}H_{18}N_2$ $(M+H)^+$ 239.15, found 239.2. ^1H NMR (300 MHz, $CDCl_3$) δ 1.15–1.28 (m, 1 H, piperazine), 3.14–3.76 (m, 8 H, piperazine), 6.65–7.73 (m, 9 H, ArH).

1-([1,1'-Biphenyl]-4-yl)piperazine (A12). Yellow oil, yield 95% (1.13 g); TLC: R_f = 0.52 (S_3); UPLC (purity > 99%): t_R = 4.40 min. LC-MS (ESI): *m/z* calcd for $C_{16}H_{18}N_2$ $(M+H)^+$ 239.15, found 239.2. ^1H NMR (500 MHz, $CDCl_3$) δ 1.69 (m, 1 H, piperazine), 2.97–3.09 (m, 4 H, piperazine), 3.14–3.23 (m, 4 H, piperazine), 6.91–7.05 (m, 2 H, ArH), 7.20–7.31 (m, 1 H, ArH), 7.35–7.44 (m, 2 H, ArH), 7.49–7.65 (m, 4 H, ArH).

1-(3-Phenoxyphenyl)piperazine (A13). Yellow oil, yield 97% (1.23 g); TLC: R_f = 0.45 (S_3); UPLC (purity = 93.6%): t_R = 4.61 min. LC-MS (ESI): *m/z* calcd for $C_{16}H_{18}N_2O$ $(M+H)^+$ 255.15, found 255.3. ^1H NMR (500 MHz, $CDCl_3$) δ 2.59–2.69 (m, 1 H, piperazine) 3.00 (s, 1 H, piperazine) 3.14–3.22 (m, 4 H, piperazine) 3.27–3.34 (m, 3 H, piperazine) 6.41–6.72 (m, 3 H, ArH) 6.96–7.39 (m, 6 H, ArH).

1-(4-Phenoxyphenyl)piperazine (A14). Yellow oil, yield 97% (1.23 g); TLC: R_f = 0.45 (S_3); UPLC (purity = 91.6%): t_R = 4.63 min. LC-MS (ESI): *m/z* calcd for $C_{16}H_{18}N_2O$ $(M+H)^+$ 255.15, found 255.3.

^{1}H NMR (500 MHz, $CDCl_3$) δ 2.60–2.77 (m, 1 H, piperazine), 2.99–3.22 (m, 8 H, piperazine), 6.80 –7.08 (m, 7 H, ArH), 7.18–7.37 (m, 2 H, ArH).

1-(3-(Trifluoromethoxy)phenyl)piperazine (A15). Yellow oil, yield 97% (1.20 g); TLC: R_f = 0.41 (S_3); UPLC (purity = 95.7%): t_R = 3.99 min. LC-MS (ESI): *m/z* calcd for $C_{11}H_{13}F_3N_2O$ $(M+H)^+$ 247.10, found 247.1. ^{1}H NMR (300 MHz, $CDCl_3$) δ 1.23 (s, 1 H, piperazine) 2.91–3.16 (m, 8 H, piperazine) 6.59–6.85 (m, 3 H, ArH), 7.19 (t, *J*=8.3 Hz, 1 H, ArH).

1-(4-(Trifluoromethoxy)phenyl)piperazine (A16). Yellow oil, yield 97% (1.20 g); TLC: R_f = 0.41 (S_3); UPLC (purity = 97.4%): t_R = 3.98 min. LC-MS (ESI): *m/z* calcd for $C_{11}H_{13}F_3N_2O$ $(M+H)^+$ 247.10, found 247.1. ^{1}H NMR (500 MHz, $CDCl_3$) δ 2.56–2.77 (m, 1 H, piperazine), 2.87–3.56 (m, 8 H, piperazine), 6.78–7.00 (m, 2 H, ArH), 7.09 (br d, *J*=8.45 Hz, 2 H, ArH).

1-(3-((Trifluoromethyl)thio)phenyl)piperazine (A17). Yellow oil, yield 96% (1.25 g); TLC: R_f = 0.42 (S_3); UPLC (purity = 93.9%): t_R = 3.69 min. LC-MS (ESI): *m/z* calcd for $C_{11}H_{13}F_3N_2S$ $(M+H)^+$ 263.08, found 263.1. ^{1}H NMR (500 MHz, $CDCl_3$) δ 2.65–2.71 (m, 1 H, piperazine), 3.10–3.16 (m, 3 H, piperazine), 3.18–3.31 (m, 5 H, piperazine), 6.99–7.01 (m, 1 H, ArH), 7.09–7.16 (m, 2 H, ArH), 7.25–7.30 (m, 1 H, ArH).

1-(4-((Trifluoromethyl)thio)phenyl)piperazine (A18). Yellow oil, yield 96% (1.25 g); TLC: R_f = 0.42 (S_3); UPLC (purity = 91.7%): t_R = 3.64 min. LC-MS (ESI): *m/z* calcd for $C_{11}H_{13}F_3N_2S$ $(M+H)^+$ 263.08, found 263.1. ^{1}H NMR (500 MHz, CHLOROFORM-*d*) δ ppm 1.80–2.06 (m, 1 H, piperazine), 2.97–3.06 (m, 4 H, piperazine), 3.17–3.29 (m, 4 H, piperazine), 6.81–6.94 (m, 2 H, ArH), 7.44–7.58 (m, 2 H, ArH).

In silico studies

Table S1. Parameters calculated according to Lipinski rule, Veber rule, and CNS MPO.

Cmpd	R_1	Lipinski rule				Veber rule		CNS MPO[e]
		MW ≤500	cLog P ≤5	HBD[a] ≤5	HBA[b] ≤10	NBR[c] ≤10	TPSA[d] ≤140 Å^2	≤4
**(*R*,*S*)-35**	H	305.37	0.76	1	3	7	61.88	5.75
**(*R*,*S*)-36**	3-F	323.36	1.16	1	4	7	61.88	5.75
**(*R*,*S*)-37**	4-F	323.36	1.16	1	4	7	61.88	5.75
**(*R*,*S*)-38**	3-Cl	339.82	1.36	1	3	7	61.88	5.75
**(*R*,*S*)-39**	4-Cl	339.82	1.36	1	3	7	61.88	5.75
**(*R*,*S*)-40**	3,4-diCl	374.26	1.88	1	3	7	61.88	5.65
**(*R*,*S*)-41**	3,5-diCl	374.26	1.88	1	3	7	61.88	5.65
**(*R*,*S*)-42**	3-CF_3	373.37	1.87	1	6	8	61.88	5.65
**(*R*,*S*)-43**	4-CF_3	373.37	1.87	1	6	8	61.88	5.65
**(*R*,*S*)-44**	3-C_6H_5	381.47	2.09	1	3	8	61.88	5.60
**(*R*,*S*)-45**	4-C_6H_5	381.47	2.09	1	3	8	61.88	5.60
**(*R*,*S*)-46**	3-OCF_3	389.37	1.64	1	7	9	71.11	5.54
**(*R*,*S*)-47**	4-OCF_3	389.37	1.64	1	7	9	71.11	5.54
**(*R*,*S*)-48**	3-OC_6H_5	397.47	1.95	1	4	9	71.11	5.48
**(*R*,*S*)-49**	4-OC_6H_5	397.47	1.95	1	4	9	71.11	5.48
**(*R*,*S*)-50**	3-SCF_3	405.44	2.27	1	6	9	87.18	5.33
**(*R*,*S*)-51**	4-SCF_3	405.44	2.27	1	6	9	87.18	5.33

[a] HBD – number of hydrogen bond donors, [b] HBA – number of hydrogen bond acceptors, [c] NBR – number of rotatable bonds, [d] TPSA – topological polar surface area, [e] CNS MPO – Central Nervous System Multi-Parameter Optimization scores were calculated using the Instant JChem 21.4.0 software (ChemAxon).

In the field of drug discovery, especially for orally administered therapeutics, the evaluation of drug-like physicochemical properties is crucial. Two widely accepted guidelines for this purpose are Lipinski's Rule of Five (RO5) and Veber's criteria. According to RO5, compounds are considered to have favorable oral bioavailability when they meet the following conditions: molecular weight (MW) ≤ 500 Da, lipophilicity (log P) ≤ 5, number of hydrogen bond donors (HBD) ≤ 5, and number of hydrogen bond acceptors (HBA) ≤ 10. Complementary to this, Veber's rule emphasizes a maximum of 10 rotatable bonds and a topological polar

surface area (TPSA) not exceeding 140 Å² as key determinants for oral drug-likeness. All compounds presented in this study conform to both RO5 and Veber's criteria, suggesting a favorable pharmacokinetic profile for oral administration.

Furthermore, to evaluate the potential of the compounds to cross the blood-brain barrier (BBB), the Central Nervous System Multiparameter Optimization (CNS MPO) score was calculated using Instant JChem software (ChemAxon, version 21.4.0). The CNS MPO approach integrates six critical physicochemical parameters: calculated lipophilicity (ClogP), distribution coefficient at physiological pH 7.4 (ClogD), molecular weight (MW), TPSA, hydrogen bond donors (HBD), and the pKa of the most basic functional group. Each parameter contributes a score between 0 and 1, resulting in a cumulative CNS MPO score ranging from 0 to 6, with higher values indicating a more favorable profile for CNS drug development. A threshold score of ≥ 4.0 is generally accepted as a benchmark for CNS drug candidates during the hit-to-lead phase. Notably, all designed compounds achieved CNS MPO scores equal to or above 4.0, and all racemic compounds exceeded a score of 5.0, reflecting optimal characteristics for effective BBB penetration.

Table S2. Antiseizure activity screening data for compounds **(*R*,*S*)-35**–**(*R*,*S*)-51** in mice *i.p.* (100 mg/kg).

Cmpd	R_1	MES[a]	6 Hz (32 mA)[b]	6 Hz (44 mA)[c]
**(*R*,*S*)-35**	H	0/4	0/4	0/4
**(*R*,*S*)-36**	3-F	0/4	2/4	1/4
**(*R*,*S*)-37**	4-F	0/4	1/4	2/4
**(*R*,*S*)-38**	3-Cl	0/4	4/4	2/4
**(*R*,*S*)-39**	4-Cl	0/4	0/4	0/4
**(*R*,*S*)-40**	3,4-diCl	0/4	2/4	1/4
**(*R*,*S*)-41**	3,5-diCl	2/4	4/4	3/4
**(*R*,*S*)-42**	3-CF_3	4/4	4/4	4/4
**(*R*,*S*)-43**	4-CF_3	0/4	3/4	3/4
**(*R*,*S*)-44**	3-C_6H_5	4/4	4/4	4/4
**(*R*,*S*)-45**	4-C_6H_5	0/4	2/4	2/4
**(*R*,*S*)-46**	3-OCF_3	4/4	4/4	4/4
**(*R*,*S*)-47**	4-OCF_3	3/4	4/4	4/4
**(*R*,*S*)-48**	3-OC_6H_5	4/4	3/4	4/4
**(*R*,*S*)-49**	4-OC_6H_5	4/4	4/4	3/4
**(*R*,*S*)-50**	3-SCF_3	4/4	4/4	4/4
**(*R*,*S*)-51**	4-SCF_3	1/4	3/4	3/4

Data indicate number of mice protected/number of mice tested. Dose of 100 mg/kg was administered *i.p.* The animals were examined at 0.5 h. A dash indicates not tested. [a] MES – maximal electroshock seizure test; [b] 6 Hz – psychomotor seizure test, 32 mA; [c] 6 Hz –seizure test, 44 mA.

Table S3. Effect of **(*R*)-46** on neuromuscular strength and motor coordination in mice.

Group	Neuromuscular strength (mN/kg)	Impairment of motor coordination (%)
	before MEST test	
control	29.53 ± 4.8	0
VPA (150 mg/kg)	28.73 ± 3.86	0
**(*R*)-46** (3 mg/kg)	27.53 ± 4.83	0
**(*R*)-46** (10 mg/kg)	30.51 ± 5.46	0
**(*R*)-46** (30 mg/kg)	29.44 ± 2.46	0
	before 6 Hz seizure threshold test	
control	26.28 ± 4.28	0
VPA (150 mg/kg)	28.05 ± 2.94	0
**(*R*)-46** (10 mg/kg)	29.35 ± 3.27	0
**(*R*)-46** (20 mg/kg)	28.3 ± 3.49	0
**(*R*)-46** (30 mg/kg)	28.39 ± 3.32	0
	before ivPTZ test	
control	29.25 ± 4.26	0
VPA (150 mg/kg)	26.83 ± 4.58	0
**(*R*)-46** (10 mg/kg)	27.20 ± 4.16	0
**(*R*)-46** (30 mg/kg)	28.26 ± 4.78	0
**(*R*)-46** (50 mg/kg)	27.27 ± 3.02	0

Data are presented as means (SD) of grip strengths in millinewtons per gram of mouse body weight (mN/g) and as a percentage of animals showing motor coordination impairment (n=12 animals/group). **(*R*)-46** was suspended in 1% Tween 80 and administered *i.p.* 30 min before testing, while valproate (VPA; positive control) was dissolved in saline and injected *i.p.* 15 min before testing. The grip strength test and the chimney test were performed shortly before seizure threshold tests. Changes in neuromuscular strength were analyzed using one way ANOVA (GraphPad Prism 8).

Table S4. Effects of **(*R*)-46** at a dose of 50 mg/kg on the concentrations of amino acids in the hippocampus of kindled mice.

Amino Acid	Group, nmol/mg (×10)			
	control	control	VPA	**(*R*)-46**
	non-kindled	kindled		
Alanine	3.6 (0.7)	3.9 (0.6)	3.6 (0.4)	3.4 (0.9)
Asparagine	0.5 (0.1)	0.6 (0.1)	0.6 (0.1)	0.5 (0.1)
Aspartic acid	13.5 (1.7)	13.6 (1.3)	12.8 (1.7)	11.6 (1.6)
GABA	28.1 (3.9)	30.4 (4.5)	29.4 (7.3)	29.0 (7.5)
Glutamic acid	68.4 (9.0)	67.8 (6.8)	60.8 (11.2)	57.2 (10.2)
Glutamine	42.5 (5.3)	37.7 (5.6)	36.6 (4.0)	34.7 (6.4)
Glycine	8.4 (1.1)	8.8 (1.1)	9.2 (1.5)	8.5 (1.1)
Isoleucine	0.2 (0.1)	0.3 (0.1)	0.3 (0.1)	0.3 (0.1)
Leucine	0.6 (0.1)	0.8 (0.3)	0.8 (0.3)	0.8 (0.2)
Lysine	1.6 (0.2)	1.7 (0.2)	1.8 (0.2)	1.5 (0.3)
Methionine	0.2 (0.0)	0.3 (0.1)	0.3 (0.1)	0.3 (0.1)
Phenylalanine	0.4 (0.1)	0.5 (0.1)	0.5 (0.1)	0.5 (0.1)
Proline	0.5 (0.1)	0.7 (0.2)	0.7 (0.3)	0.7 (0.2)
Serine	8.0 (1.4)	8.6 (1.0)	7.7 (1.0)	7.4 (1.5)
Threonine	2.6 (0.6)	2.4 (0.4)	2.6 (0.3)	2.3 (0.5)
Tyrosine	0.4 (0.1)	0.6 (0.1)	0.5 (0.1)	0.5 (0.2)
Valine	0.7 (0.1)	0.8 (0.2)	0.8 (0.2)	0.7 (0.2)

(*R*)-46, valproate (VPA, 150 mg/kg) or vehicle were administered *i.p.* every 24 h. Kindling was induced by administering PTZ at a subconvulsive dose of 40 mg/kg (*i.p.*) three times a week, 30 min after the administration of compound **(*R*)-46**, VPA, or vehicle (n=9–10). Data are presented as means (±SD). The statistical significance was evaluated by a one-way ANOVA (GraphPad Prism 8).

Table S5. Radioligand binding and functional assays.

**Binding studies**	
Na^+ channel (site 2)	[1]
Cav1.2 (L-type) Human Calcium Ion Channel Binding (Dihydropyridine Site)	[2]
Potassium channel (hERG)	[3]
**Functional studies**	
TRPA1 (*h*) transient potential ion channel cell based antagonist calcium flux assay	[4]
TRPM8 (*h*) (antagonist effect)	[5]
TRPV1 (VR1) *(h)* (antagonist effect)	[6]
5-HT_{2C} (agonist effect)	[7]

Assays were performed commercially in Eurofins Laboratories (Poitiers, France) or Eurofins Panlabs Discovery Services Taiwan, Ltd. (New Taipei City, Taiwan).

Explanation of low TQI according to manufacturer’s manual (troubleshooting):

Compounds with very low affinity to plasma proteins and hence high free fractions (fu > 30%) are not accurately predicted. Low affinity compounds yield supernatant concentrations in the assay that deviate only marginally from the reference signals

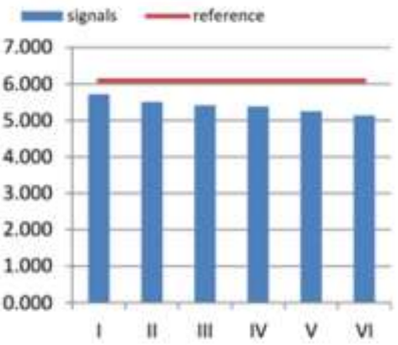

Illustration of a bar plot of a compound exhibiting weak protein binding (c.f. individual data analysis tabs of the spreadsheet). The blue bars show the detected signals in the supernatants of TRANSIL wells I to VI. As the compound binds only weekly to the proteins, supernatant concentrations differ only marginally from the reference signals (red line).

Metabolic stability *in silico*

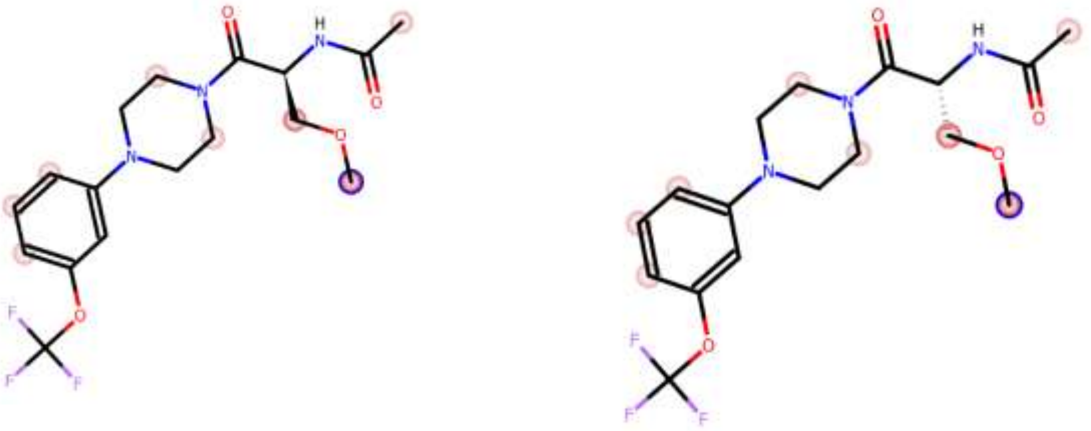

Fig. S1. The MetaSite 6.0.1. software prediction of the most probable sites of **(*R*)-46** and **(*S*)-46**. The darker red color – the higher probability to be involved in the metabolism pathway. The blue circle marked the site of compound with the highest probability of metabolic bioconversion.

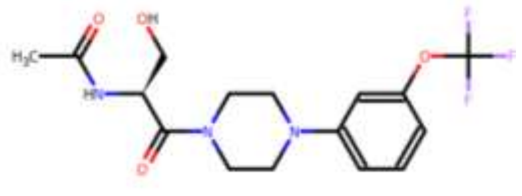

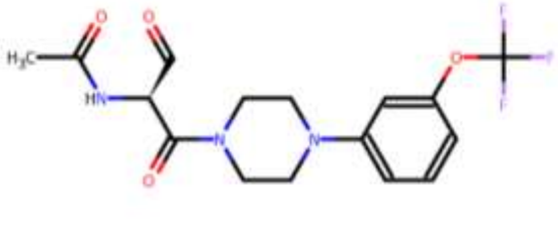

100% **50%**

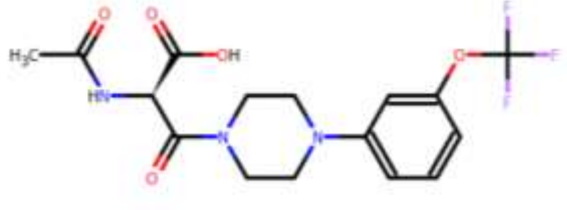

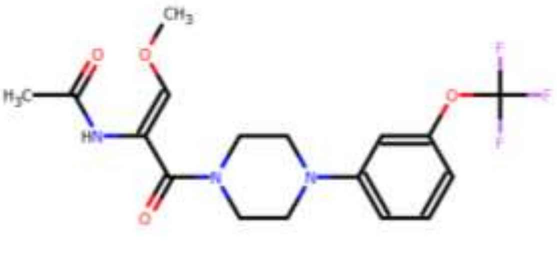

50% **50%**

Fig. S2. The MetaSite 6.0.1. software prediction of the most probable hydroxylations/oxydations of **(*R*)-46**. The results obtained for **(*S*)-46** were similar to **(*R*)-46**.

Metabolic stability *in vitro*. Determination of metabolic pathways (mouse liver microsomes)

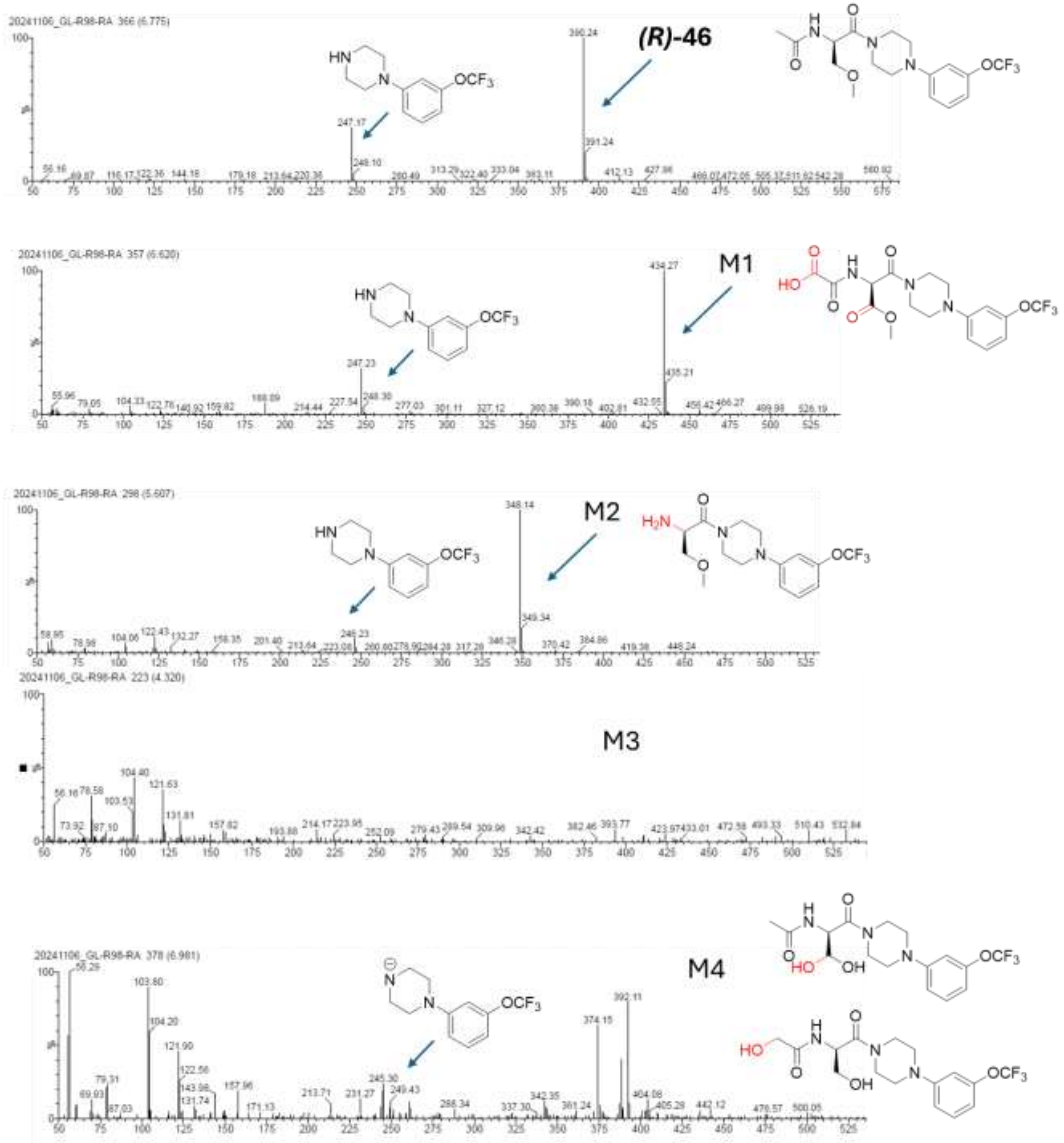

Fig. S3. MS of **(*R*)-46** and its metabolites after 120 min incubation with MLMs. The most probable structures of metabolites were also proposed.

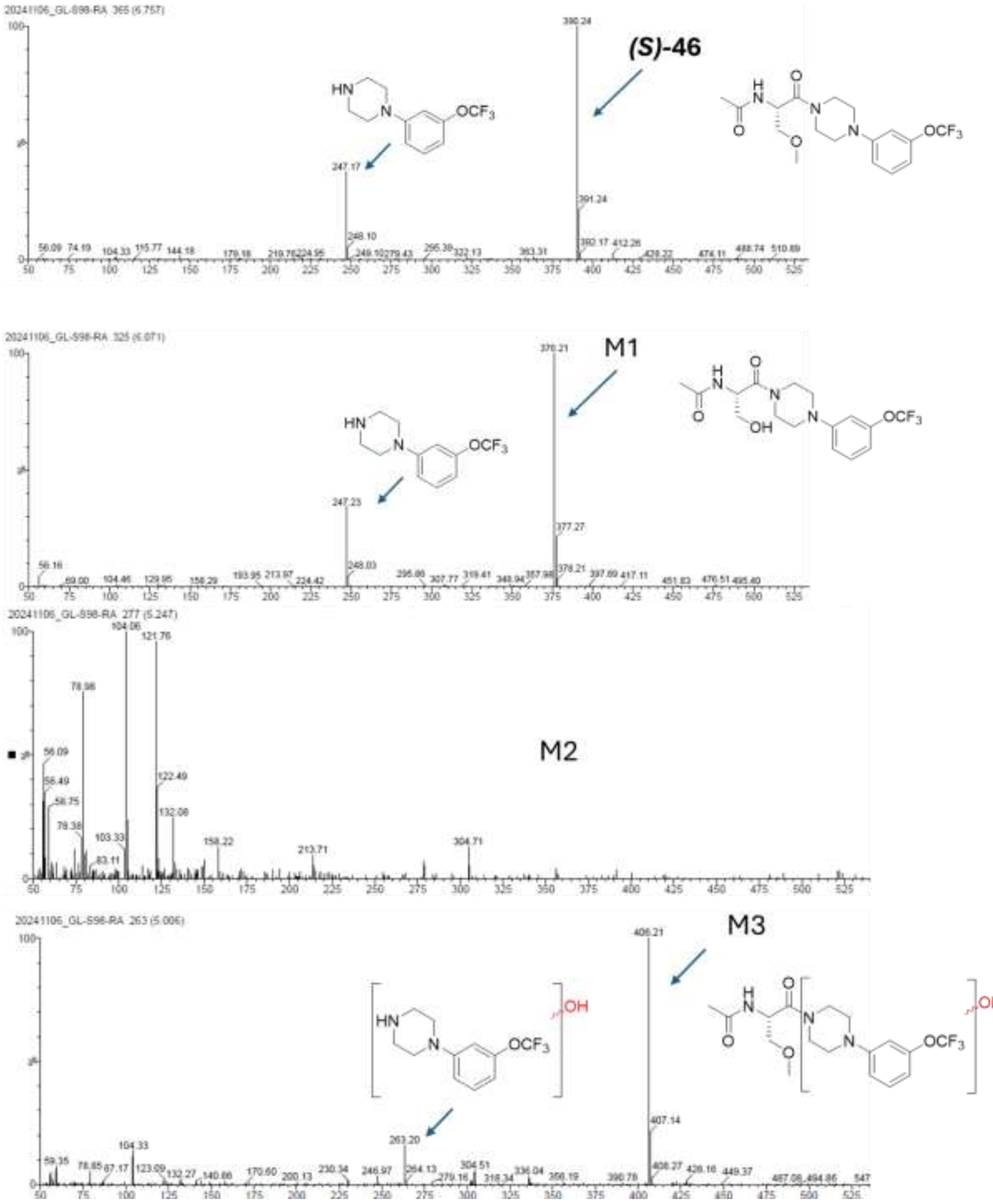

Fig. S4. MS of **(*S*)-46** and its metabolites after 120 min incubation with MLMs. The most probable structures of metabolites were also proposed.

CYP inhibition

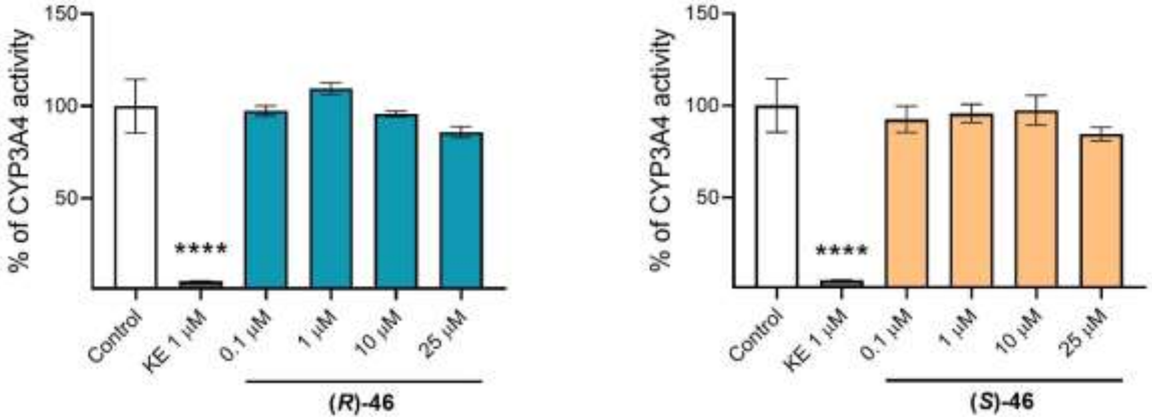

Fig. S5. The influence on CYP3A4 activity. Statistical significance (∗∗∗∗p<0.0001) was analyzed by Graph Pad Prism 8.0.1 software using One-way ANOVA and Bonferroni's Multiple Comparison Post Test. KE = reference inhibitor ketoconazole

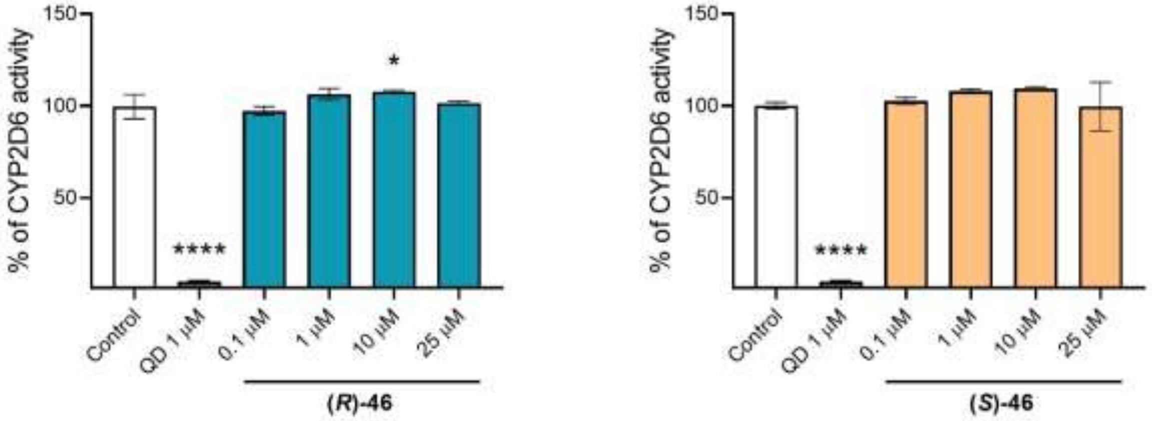

Fig. S6. The influence on CYP2D6 activity. Statistical significance (∗p<0.05, ∗∗∗∗p<0.0001) was analyzed by Graph Pad Prism 8.0.1 software using One-way ANOVA and Bonferroni's Multiple Comparison Post Test. QD = reference inhibitor quinidine.

Metabolic stability *in vitro*. Determination of metabolic pathways (MLMs)

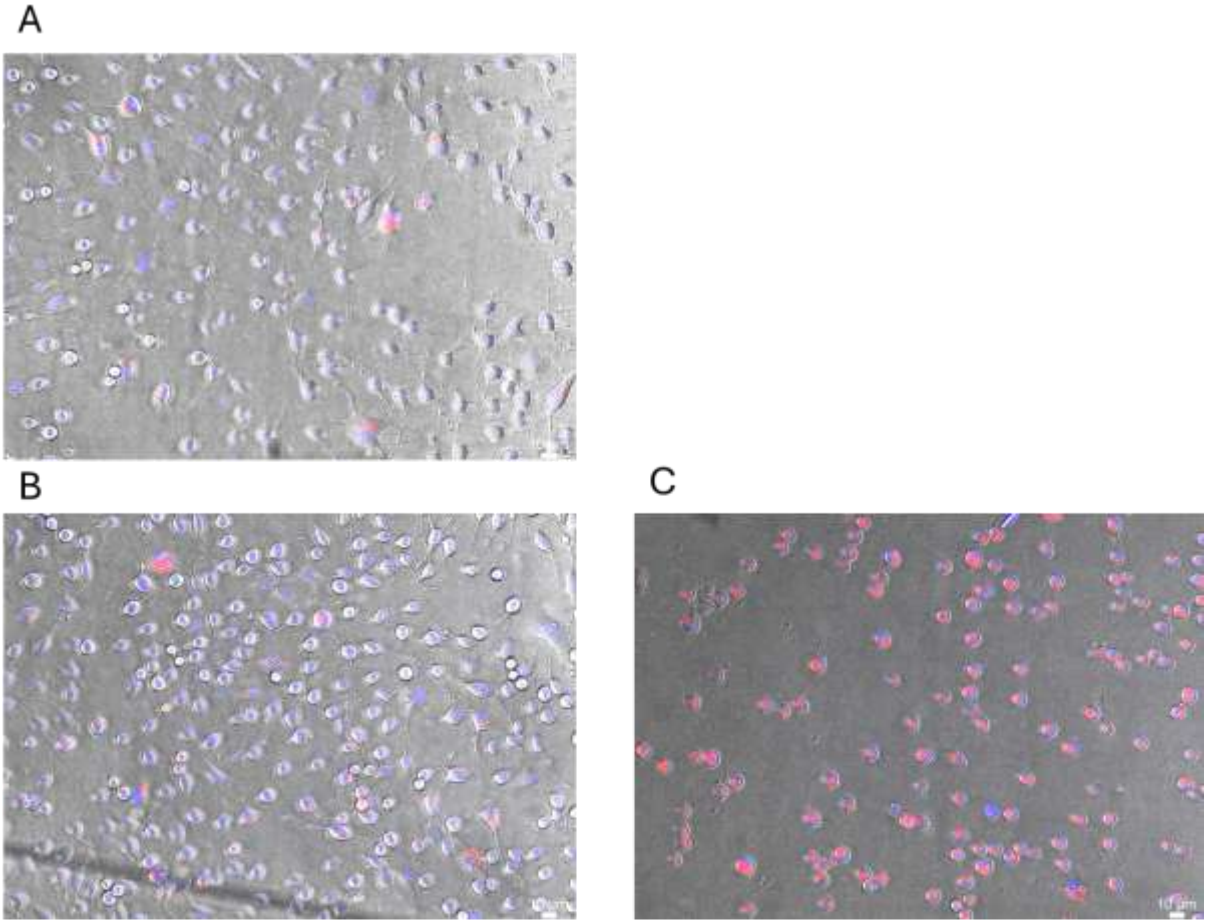

Fig. S7. Microscopy images of not treated HepG2 cells (A) and exposed for 24 h on 10 µM (B) and 50 µM (C) of verapamil (phospholipidosis positive control). The cells were stained by LYSO-ID® Red cytotoxicity kit containing Dual Color Detection Reagent. The red fluorescent lysosomal signal and the blue nuclear signal were registered by microscope Leica DMi8.

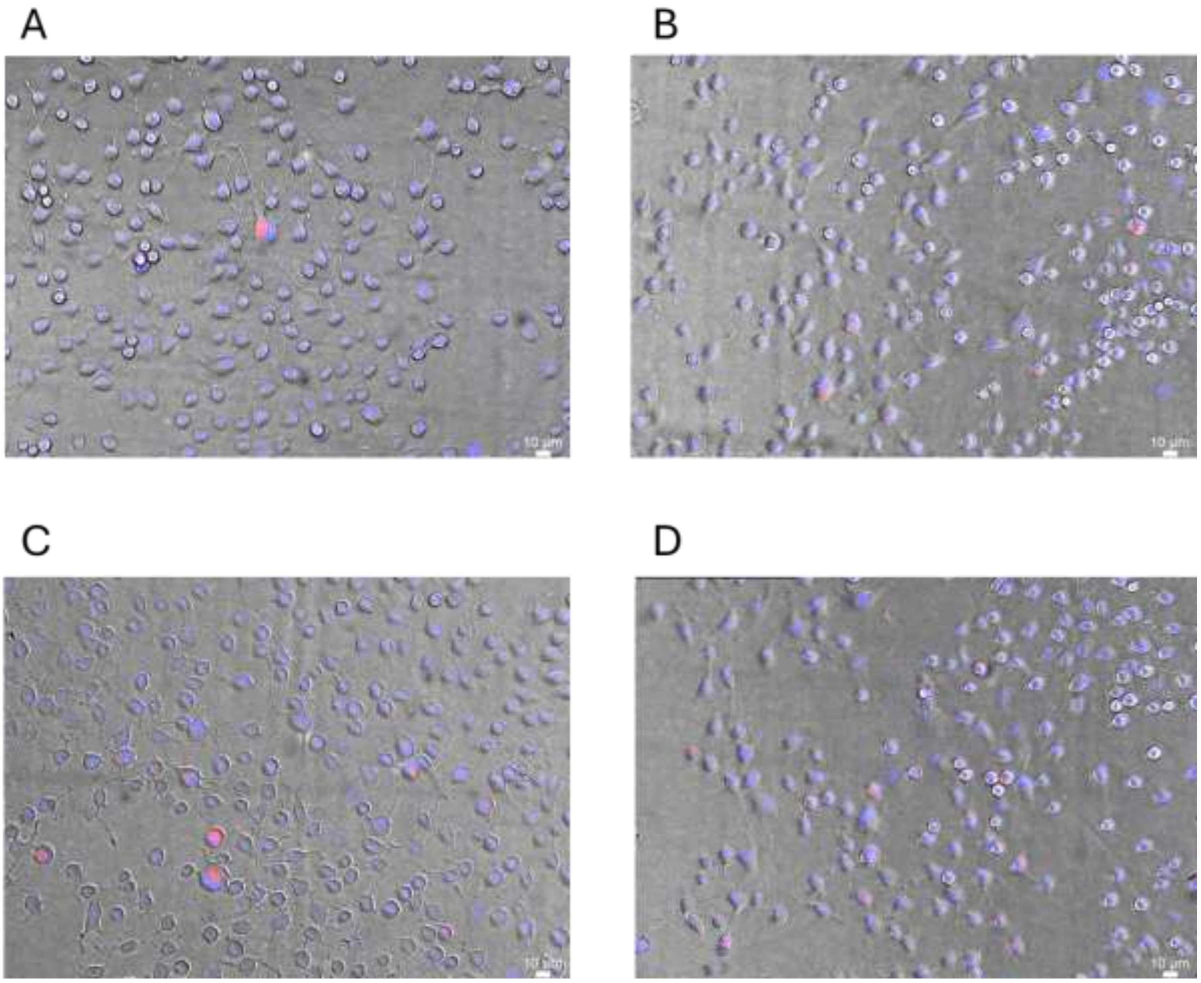

Fig. S8. Microscopy images of HepG2 cells exposed for 24 h on 50 µM (A), 100 µM (B)of **(*R*)-46** and 50 µM (C), 100 µM (D) of **(*S*)-46**. The cells were stained by LYSO-ID® Red cytotoxicity kit containing Dual Color Detection Reagent. The red fluorescent lysosomal signal and the blue nuclear signal were registered by microscope Leica DMi8.

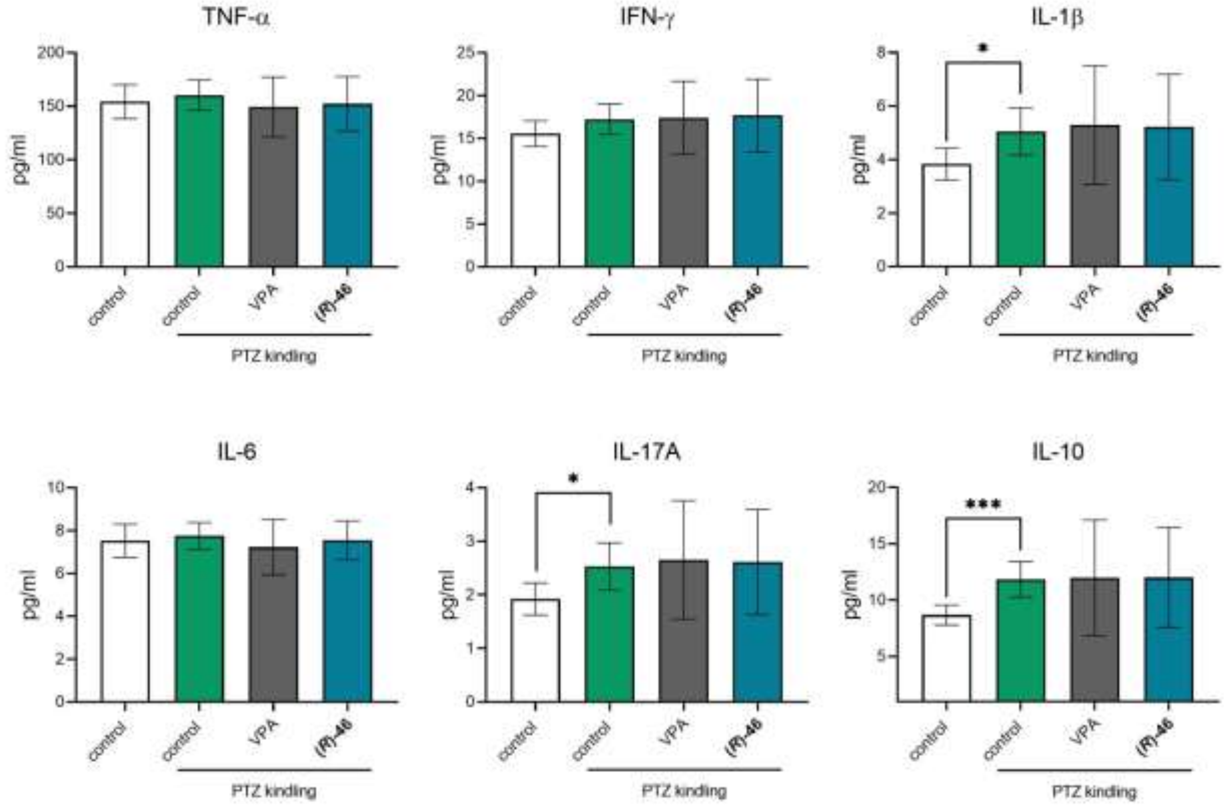

Fig. S9. Effects of **(*R*)-46** at a dose of 50 mg/kg on the concentrations of inflammatory markers in the hippocampus of kindled mice. **(*R*)-46**, valproate (VPA, 150 mg/kg) or vehicle were administered *i.p.* every 24 h. Kindling was induced by administering PTZ at a subconvulsive dose of 40 mg/kg (*i.p.*) three times a week, 30 min after the administration of compound **(*R*)-46**, VPA, or vehicle. Differences between the non-kindled control group and the PTZ-kindled control group were evaluated using Student's t-test: *p<0.05, ***p<0.001. Differences between all studied groups were evaluated using one way ANOVA (GraphPad Prism 8).

Pharmacokinetic studies

Concentration versus time profiles of **(*R*)-46** in serum and brain tissue following *i.p.* administration of this compound at two doses, i.e. 25 and 50 mg/kg to mice are presented in **Fig. S10.**

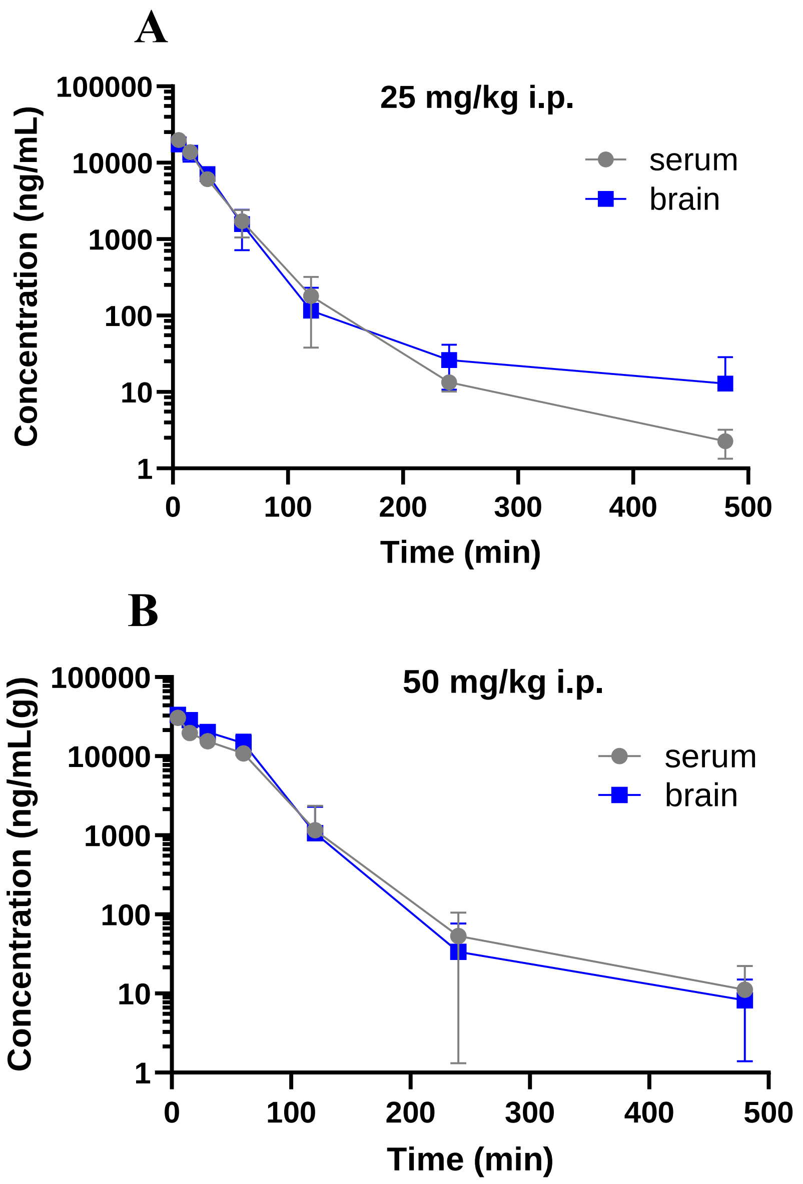

Fig. S10. Mean (±SD) serum and brain concentrations of **(*R*)-46** after *i.p.* administration of this compound at two doses 25 mg/kg (A) and 50 mg/kg **(**B) to mice (n=3-4).

Fig. S11 shows the pharmacokinetic profile of **(*R*)-46** in serum and brain after *p.o.* and *i.v.* administration of a dose of 25 and 10 mg/kg, respectively.

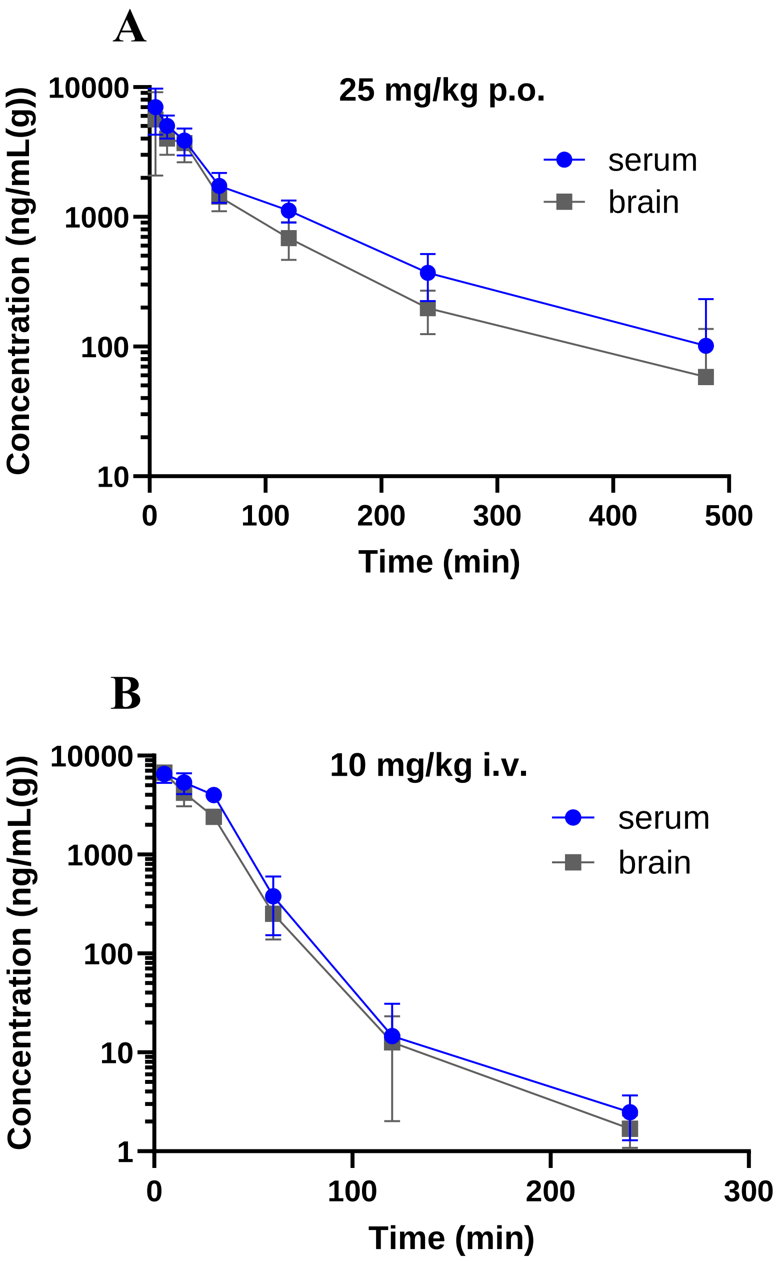

Fig. S11. Mean (±SD) serum and brain concentrations of **(*R*)-46** after *p.o.* (A) and *i.v.* (B) administration of this compound to mice (n=3-4).

Application: Bath solution (black), 0.34% DMSO (red)

First pulse

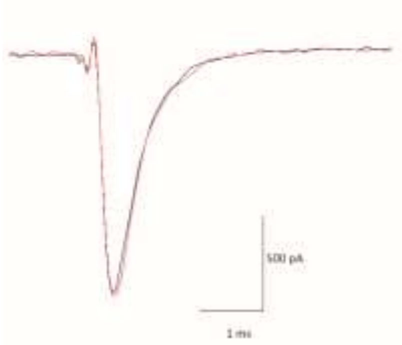

Last pulse

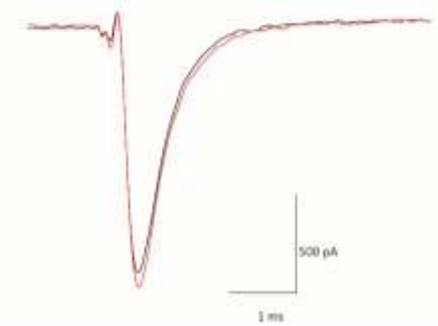

Application: Bath solution (black), 250 µM Carbamazepine (red)

First pulse

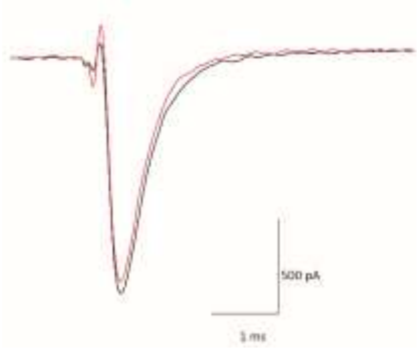

Last pulse

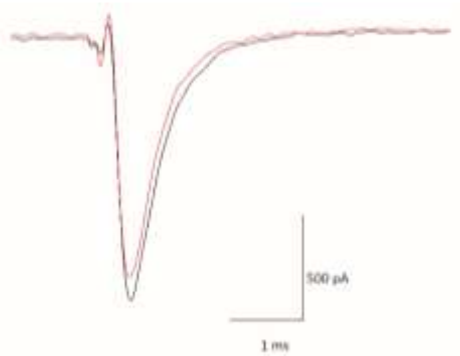

Application: Bath solution (black), 250 µM **(*R*)-46** (red)

First pulse

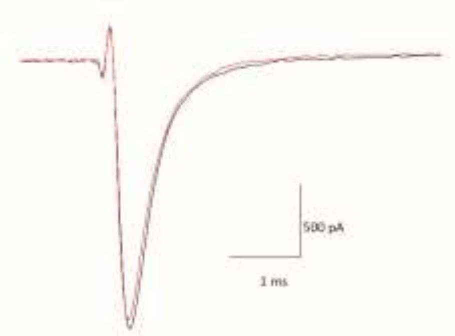

Last pulse

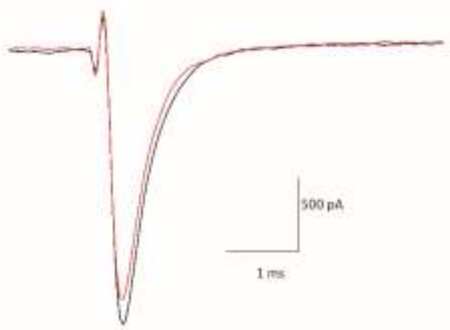

Fig. S12. Rate dependence effect of control, Carbamazepine and **(*R*)-46** on voltage gated sodium channels expressed in N1E-115 cells (including Nav1.1, Nav1.2, Nav1.3, Nav1.6, and Nav1.7).

Application: Bath solution (black), 0.34% DMSO (red), 250 µM LCS (green)

Resting state **Fast inactivated state** **Slow inactivated state**

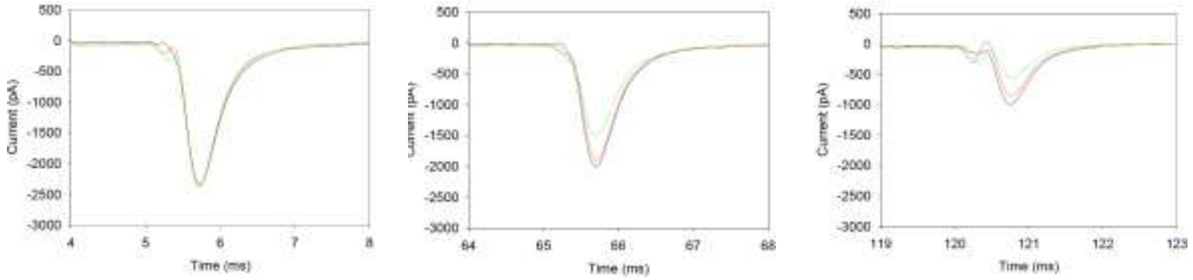

Application: Bath solution (black), 250 µM **(*R*)-46** (red)

Resting state **Fast inactivated state** **Slow inactivated state**

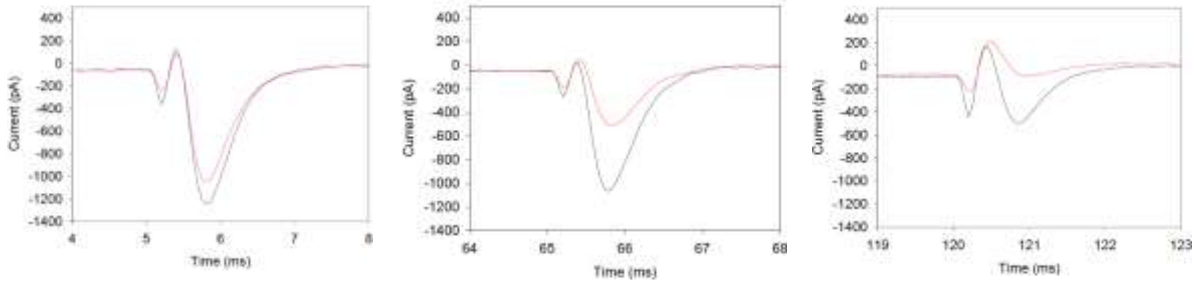

Fig. S13. State dependence effect of control, LCS and **(*R*)-46** on voltage gated sodium channels expressed in N1E-115 cells (including Nav1.1, Nav1.2, Nav1.3, Nav1.6, and Nav1.7).

Application: Bath solution (black) and 0.1% DMSO (red)

Resting state **Fast inactivated state** **Slow inactivated state**

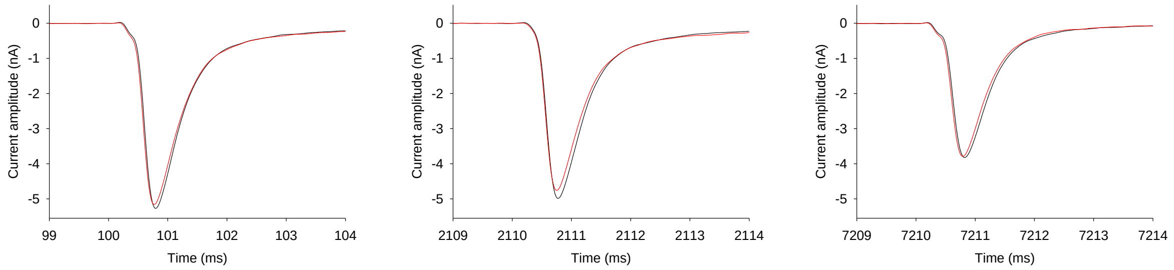

Application: Bath solution (black) and 250 µM **(*R*)-46** (red)

Resting state **Fast inactivated state** **Slow inactivated state**

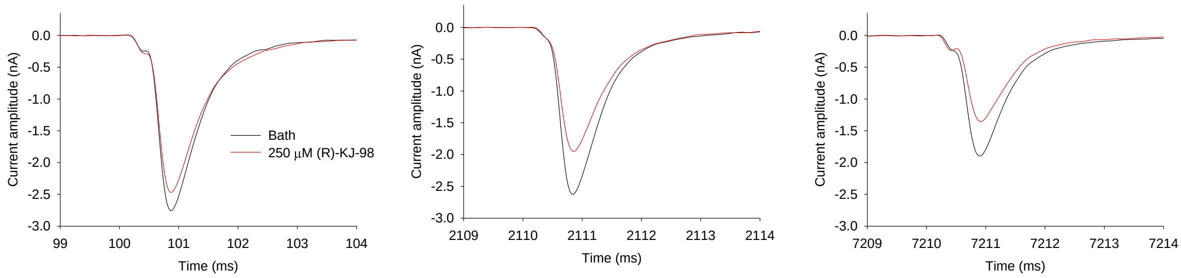

Application: Bath solution (black) and 250 µM LCS (red)

Resting state **Fast inactivated state** **Slow inactivated state**

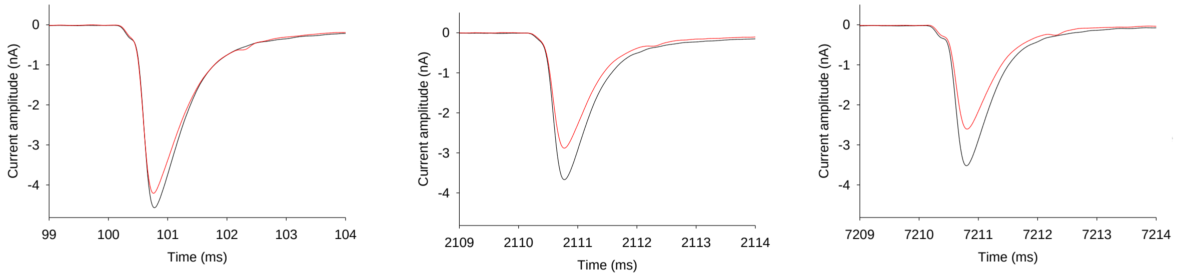

Application: Bath solution (black) and 10 µM Propafenone (red)

Resting state **Fast inactivated state** **Slow inactivated state**

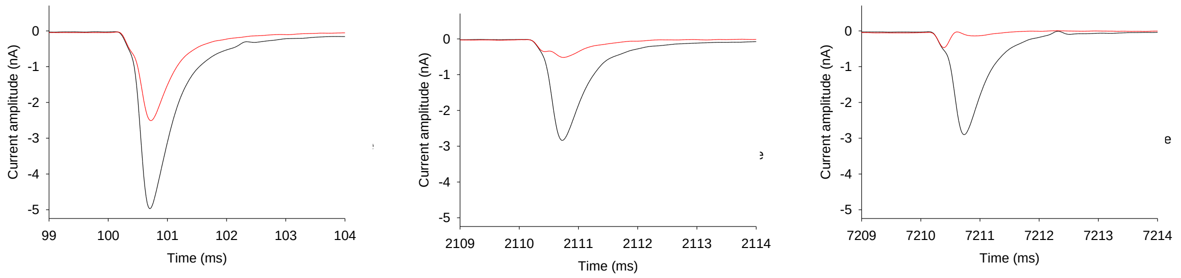

Fig. S14. Effect of control, LCS and (*R*)-46 and propafenone on voltage gated sodium channel subunit Nav1.5 expressed in CHO cells.

References

1. Brown, G.B. 3H-Batrachotoxinin-A Benzoate Binding to Voltage-Sensitive Sodium Channels: Inhibition by the Channel Blockers Tetrodotoxin and Saxitoxin. *J. Neurosci.* **1986**, *6*, 2064–2070, doi:10.1523/JNEUROSCI.06-07-02064.1986.
2. Gould, R.J.; Murphy, K.M.; Snyder, S.H. [3H]Nitrendipine-Labeled Calcium Channels Discriminate Inorganic Calcium Agonists and Antagonists. *Proc. Natl. Acad. Sci. U.S.A.* **1982**, *79*, 3656–3660, doi:10.1073/pnas.79.11.3656.
3. Huang, X.-P.; Mangano, T.; Hufeisen, S.; Setola, V.; Roth, B.L. Identification of Human Ether-à-Go-Go Related Gene Modulators by Three Screening Platforms in an Academic Drug-Discovery Setting. *ASSAY Drug Dev. Tech.* **2010**, *8*, 727–742, doi:10.1089/adt.2010.0331.
4. Https://Www.Eurofinsdiscovery.Com/Catalogmanagement/viewItem/TRPA1-Human-Transient-Potential-Ion-Channel-Cell-Based-Antagonist-Calcium-Flux-Assay-Cerep/5372.
5. Behrendt, H.-J.; Germann, T.; Gillen, C.; Hatt, H.; Jostock, R. Characterization of the Mouse Cold-Menthol Receptor TRPM8 and Vanilloid Receptor Type-1 VR1 Using a Fluorometric Imaging Plate Reader (FLIPR) Assay. *Br. J. Pharmacol.* **2004**, *141*, 737–745, doi:10.1038/sj.bjp.0705652.
6. Phelps, P.T.; Anthes, J.C.; Correll, C.C. Cloning and Functional Characterization of Dog Transient Receptor Potential Vanilloid Receptor-1 (TRPV1). *Eur. J. Pharmacol.* **2005**, *513*, 57–66, doi:10.1016/j.ejphar.2005.02.045.
7. Porter, R.H.P.; Benwell, K.R.; Lamb, H.; Malcolm, C.S.; Allen, N.H.; Revell, D.F.; Adams, D.R.; Sheardown, M.J. Functional Characterization of Agonists at Recombinant Human 5-HT2A, 5-HT2B and 5-HT2C Receptors in CHO-K1 Cells. *British Journal of Pharmacology* **1999**, *128*, 13–20, doi:10.1038/sj.bjp.0702751.

Chiral SFC traces

(*R,S*)-*N*-(1-(4-([1,1'-biphenyl]-3-yl)piperazin-1-yl)-3-methoxy-1-oxopropan-2-yl)acetamide (*R,S*)-44

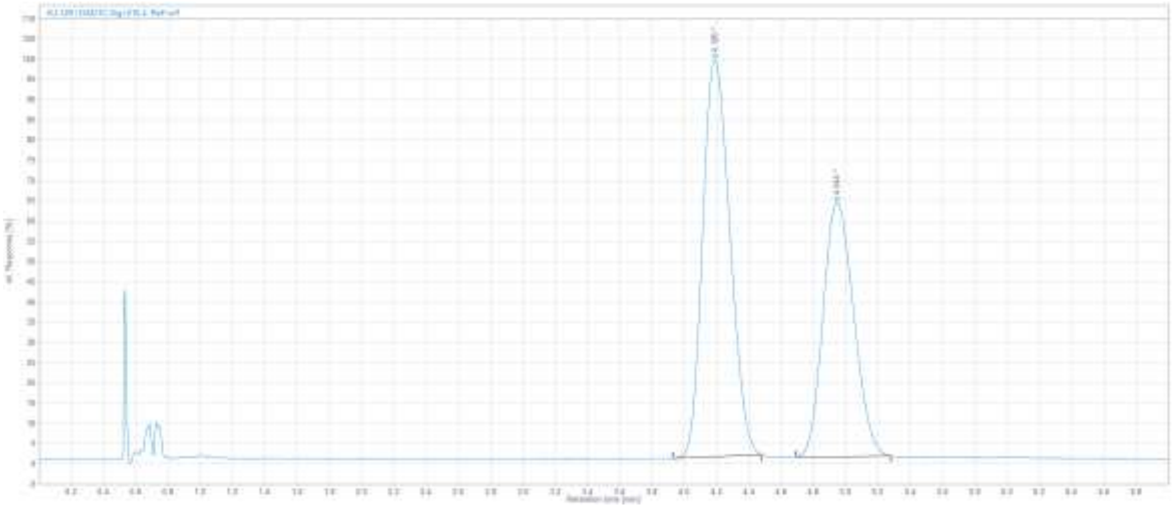

(*R*)-*N*-(1-(4-([1,1'-biphenyl]-3-yl)piperazin-1-yl)-3-methoxy-1-oxopropan-2-yl)acetamide (*R*)-44

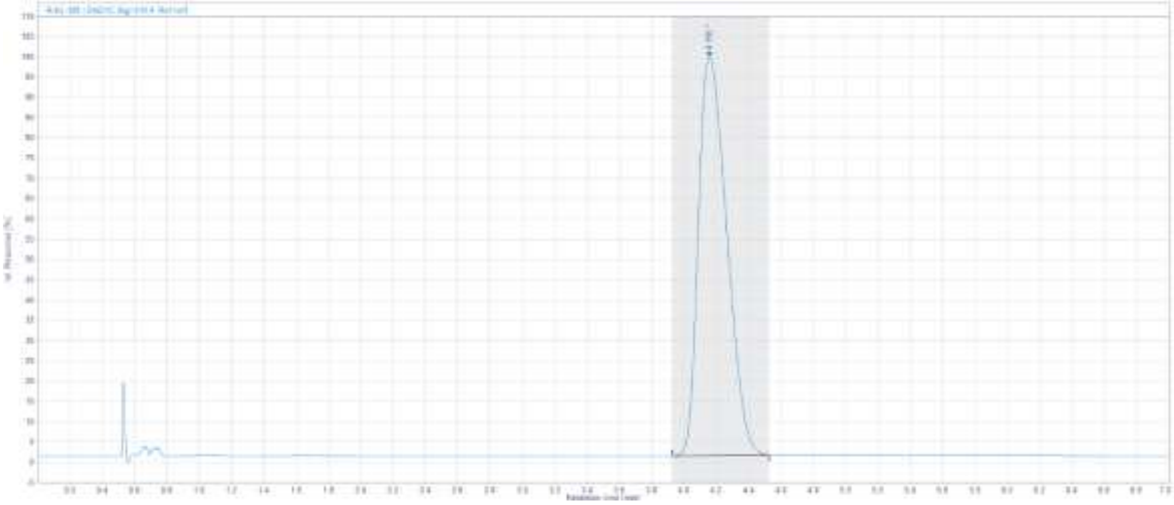

(*S*)-*N*-(1-(4-([1,1'-biphenyl]-3-yl)piperazin-1-yl)-3-methoxy-1-oxopropan-2-yl)acetamide (*S*)-44

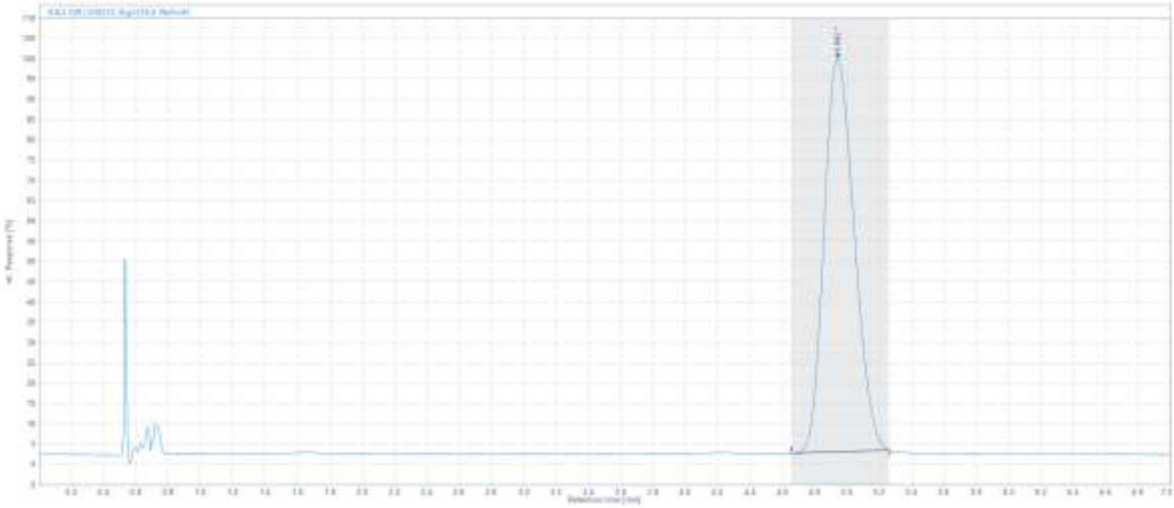

(*R,S*)-*N*-(3-methoxy-1-oxo-1-(4-(3-(trifluoromethoxy)phenyl)piperazin-1-yl)propan-2-yl)acetamide (*R,S*)-46

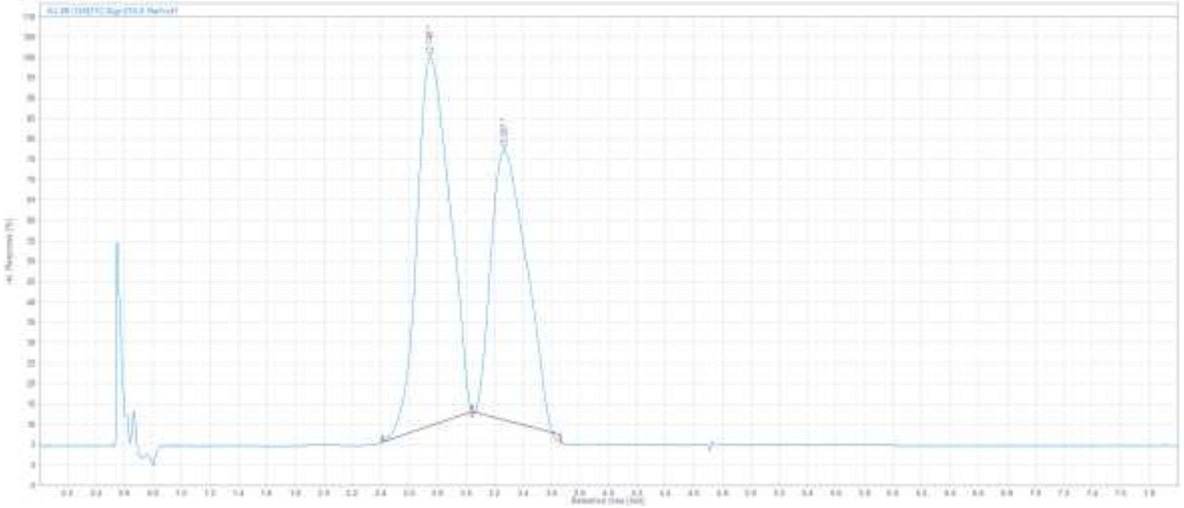

(*R*)-*N*-(3-methoxy-1-oxo-1-(4-(3-(trifluoromethoxy)phenyl)piperazin-1-yl)propan-2-yl)acetamide (*R*)-46

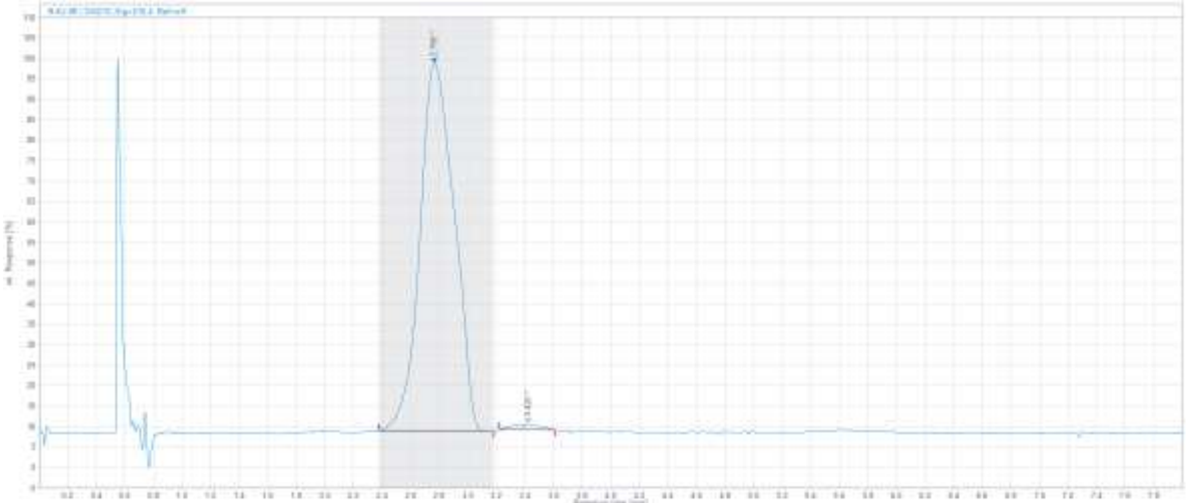

(*S*)-*N*-(3-methoxy-1-oxo-1-(4-(3-(trifluoromethoxy)phenyl)piperazin-1-yl)propan-2-yl)acetamide (*S*)-46

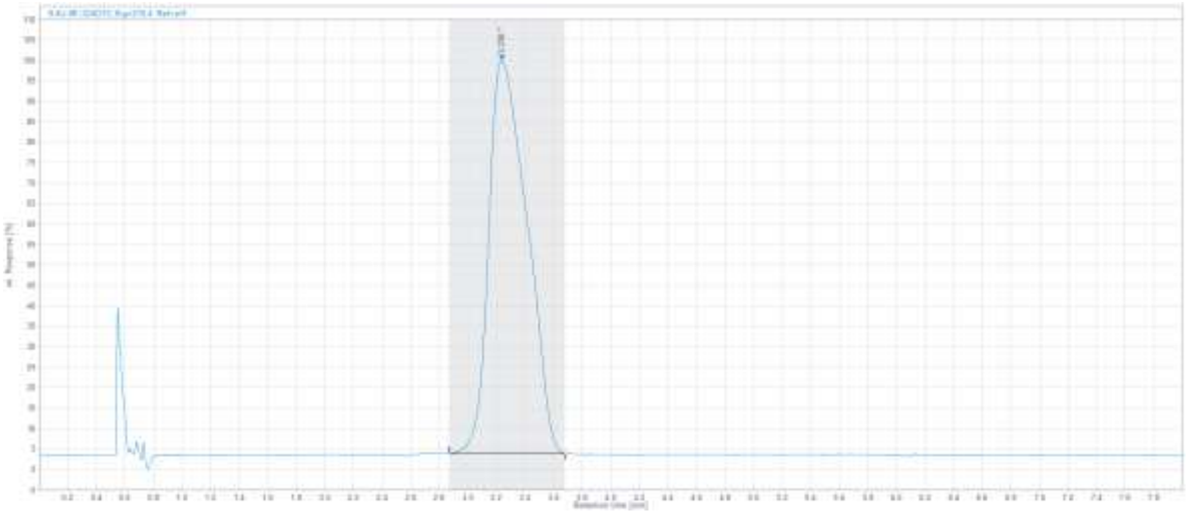

(*R,S*)-*N*-(3-methoxy-1-oxo-1-(4-(3-((trifluoromethyl)thio)phenyl)piperazin-1-yl)propan-2-yl)acetamide (*R,S*)-50

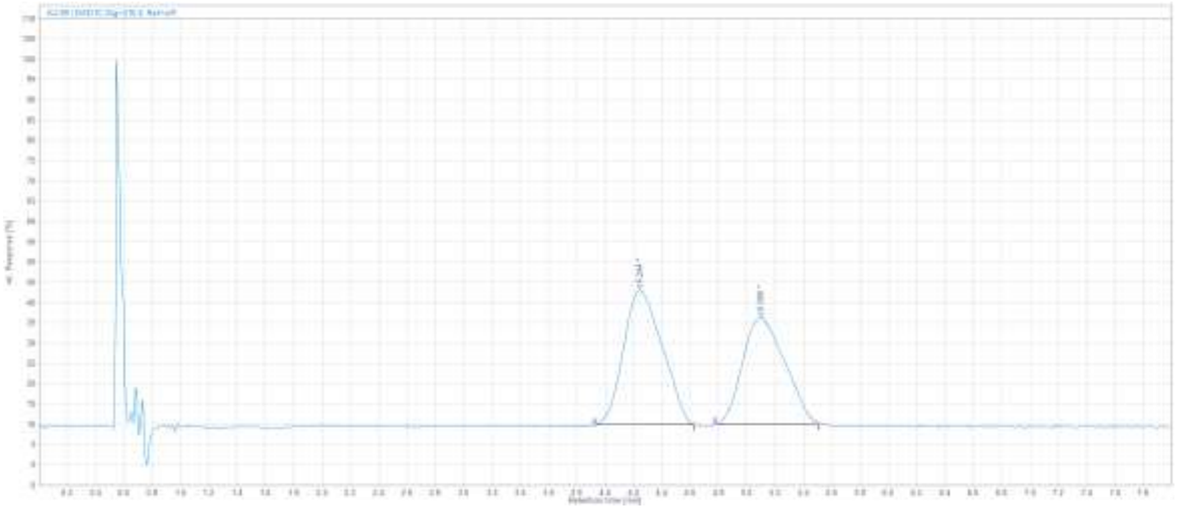

(*R*)-*N*-(3-methoxy-1-oxo-1-(4-(3-((trifluoromethyl)thio)phenyl)piperazin-1-yl)propan-2-yl)acetamide (*R*)-50

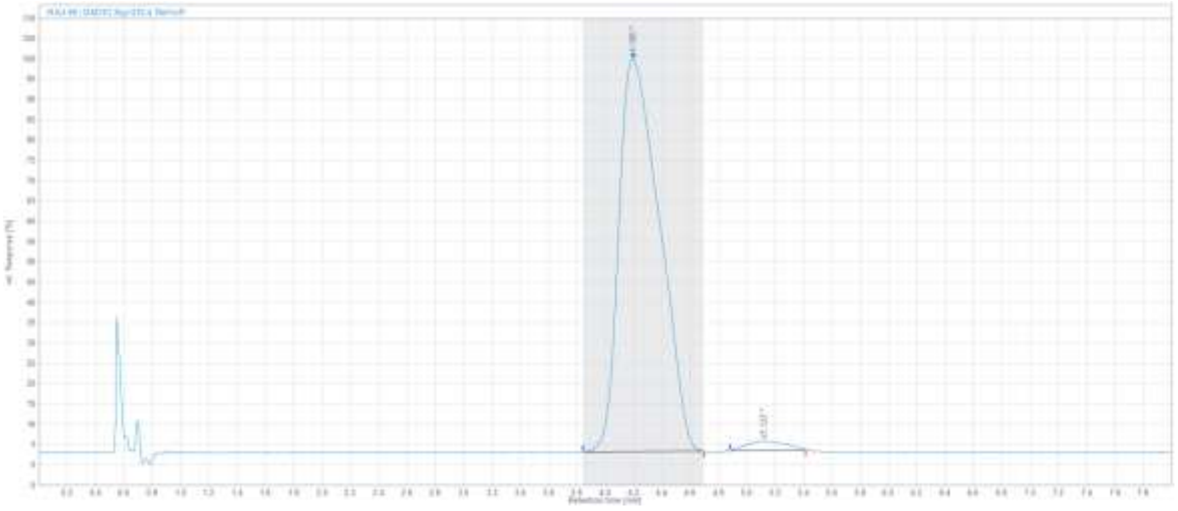

(*S*)-*N*-(3-methoxy-1-oxo-1-(4-(3-((trifluoromethyl)thio)phenyl)piperazin-1-yl)propan-2-yl)acetamide (*S*)-50

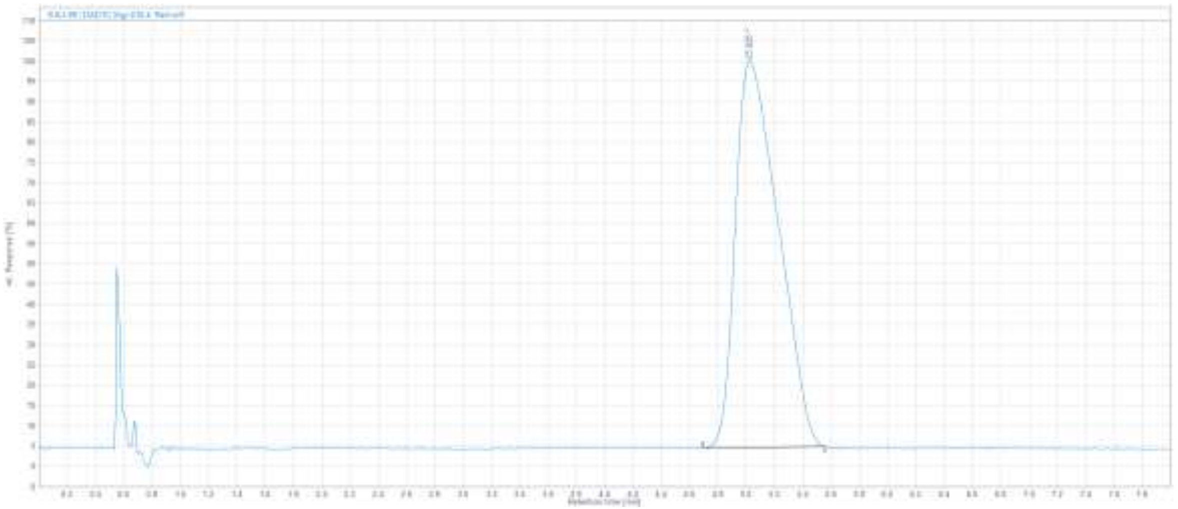

HRMS traces

(*R*)-*N*-(1-(4-([1,1'-biphenyl]-3-yl)piperazin-1-yl)-3-methoxy-1-oxopropan-2-yl)acetamide (*R*)-44

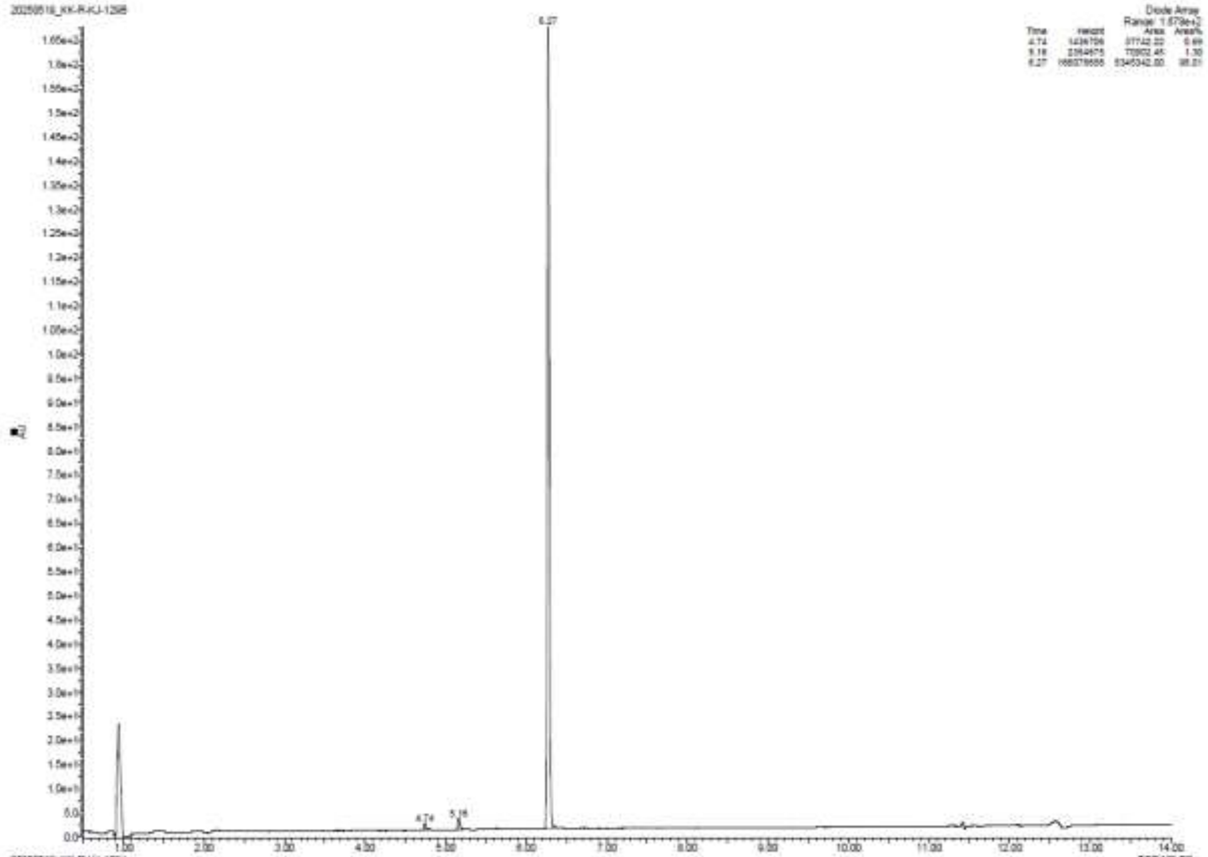

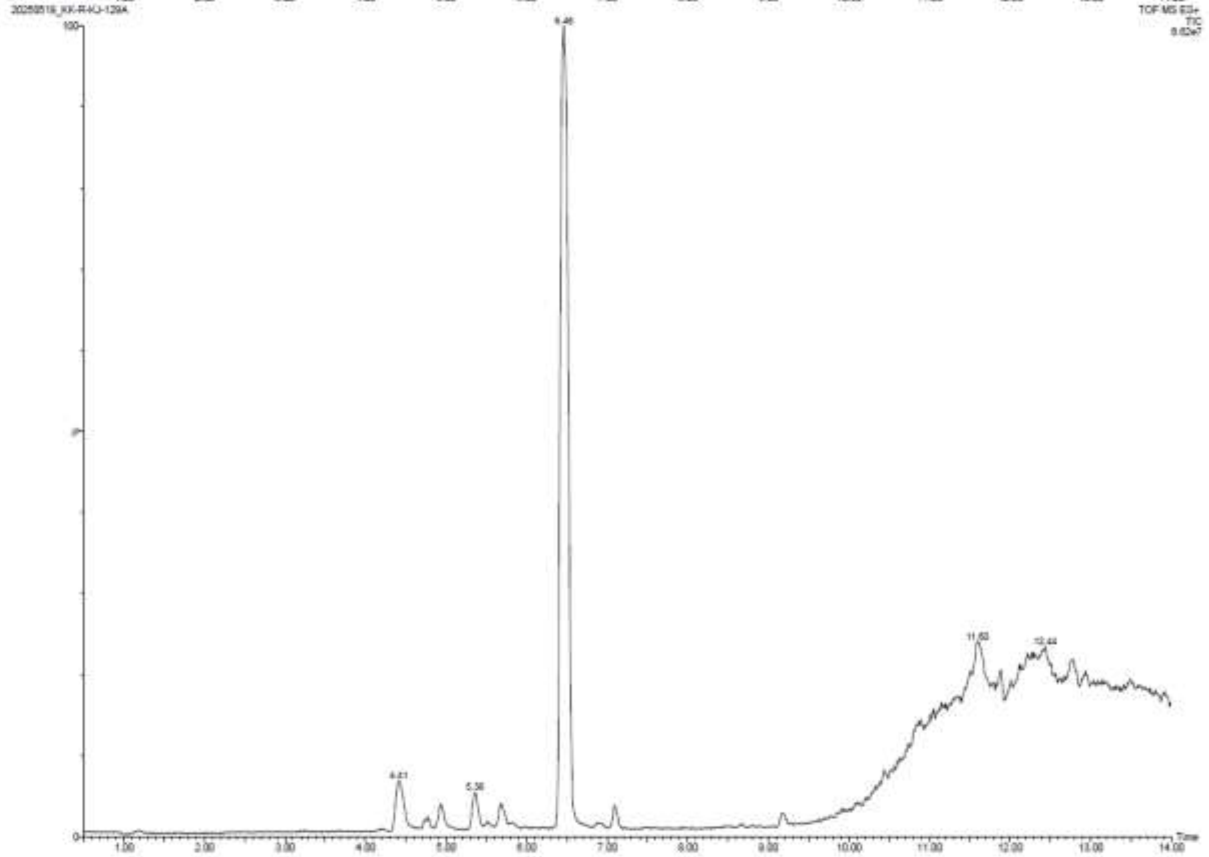

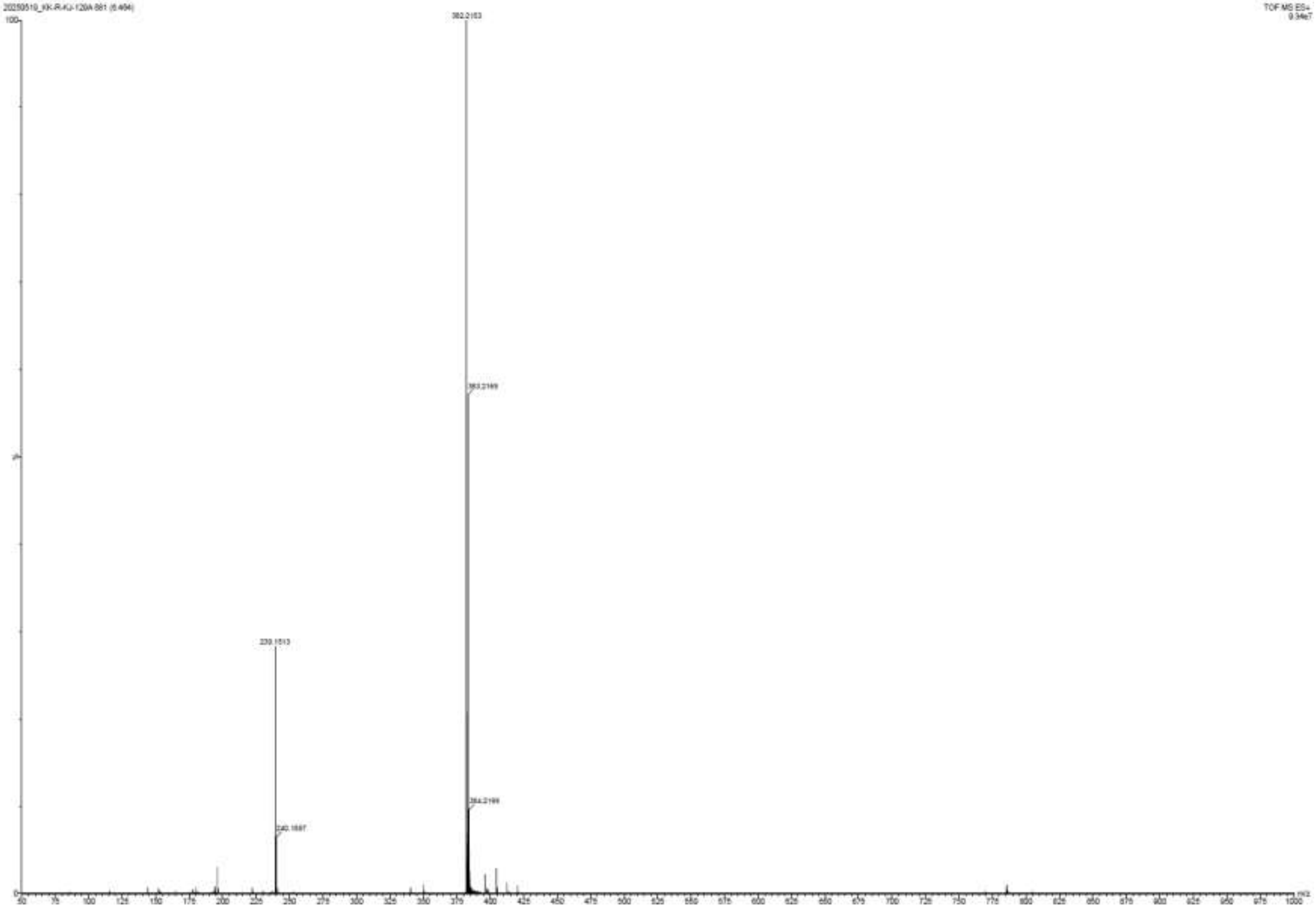
TOF MS ES+
9.34e7
382.2153
383.2189
239.1513
384.2199
240.1587

(*S*)-*N*-(1-(4-([1,1'-biphenyl]-3-yl)piperazin-1-yl)-3-methoxy-1-oxopropan-2-yl)acetamide (*S*)-44

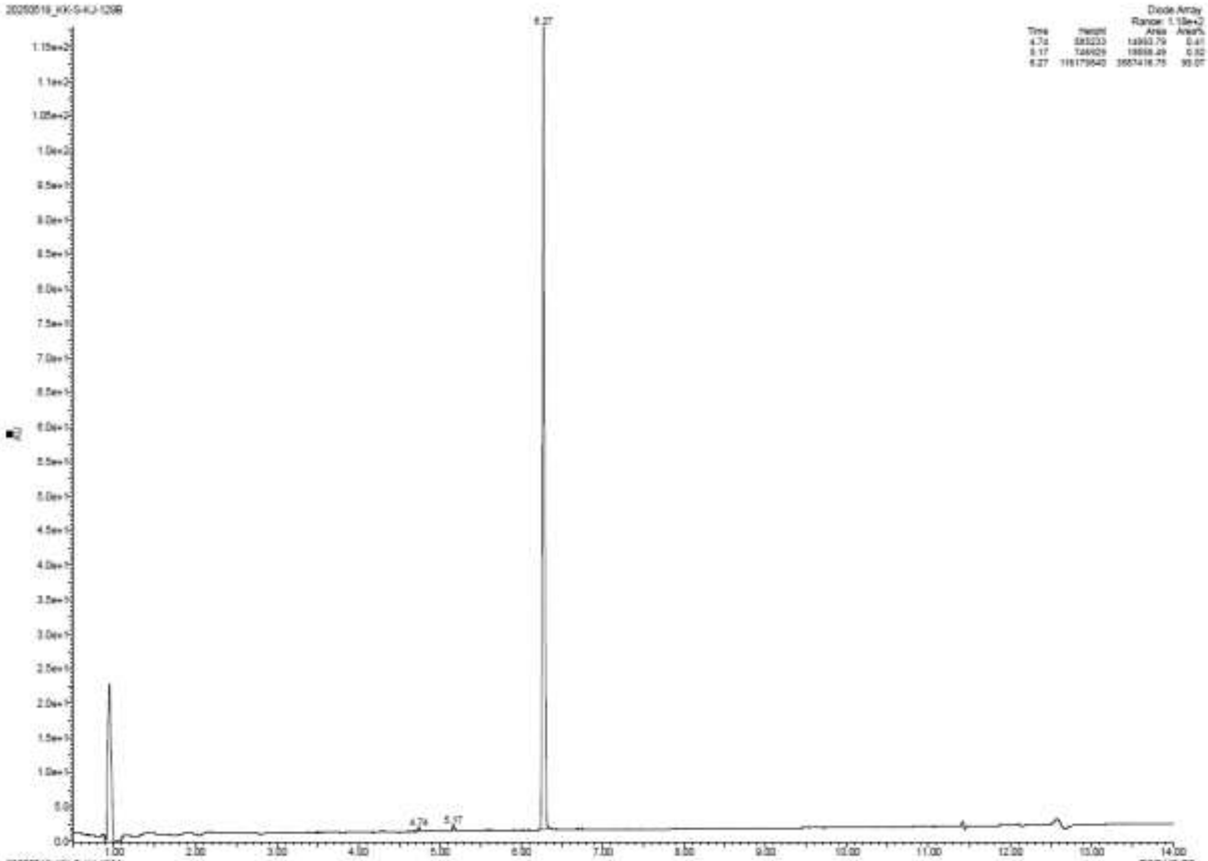

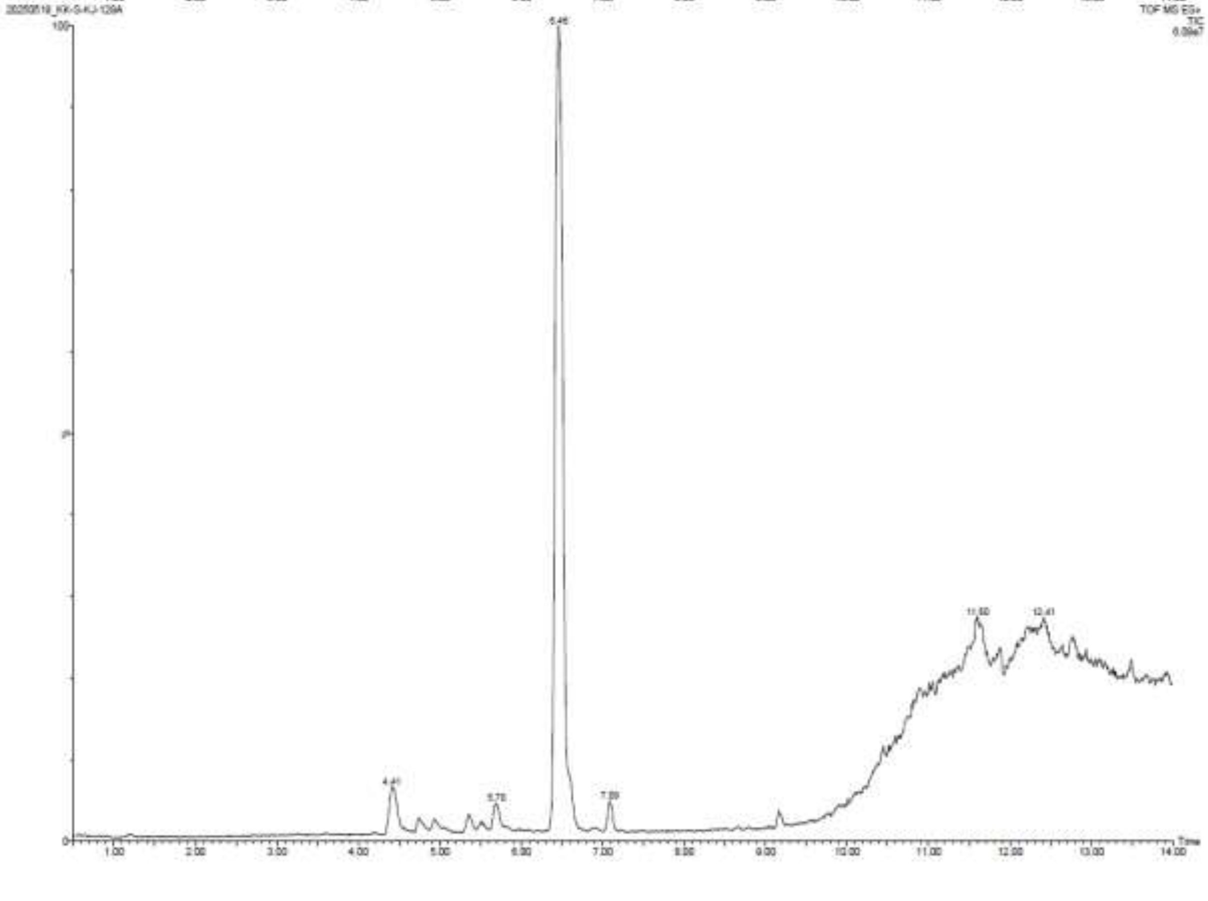

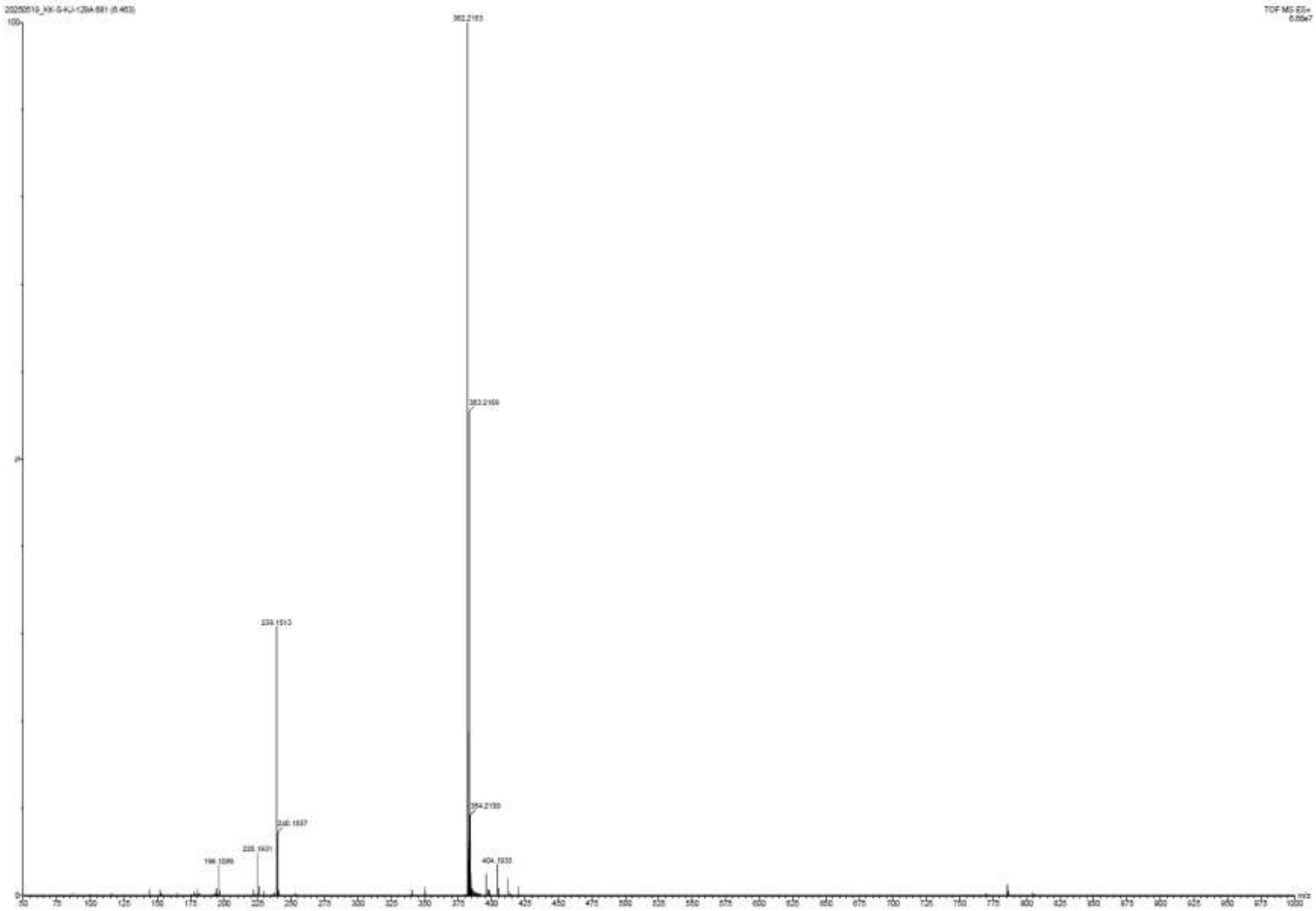
TOF MS ES+
383.2169
239.1513
404.1933
m/z

(*R*)-*N*-(3-methoxy-1-oxo-1-(4-(3-(trifluoromethoxy)phenyl)piperazin-1-yl)propan-2-yl)acetamide (*R*)-46

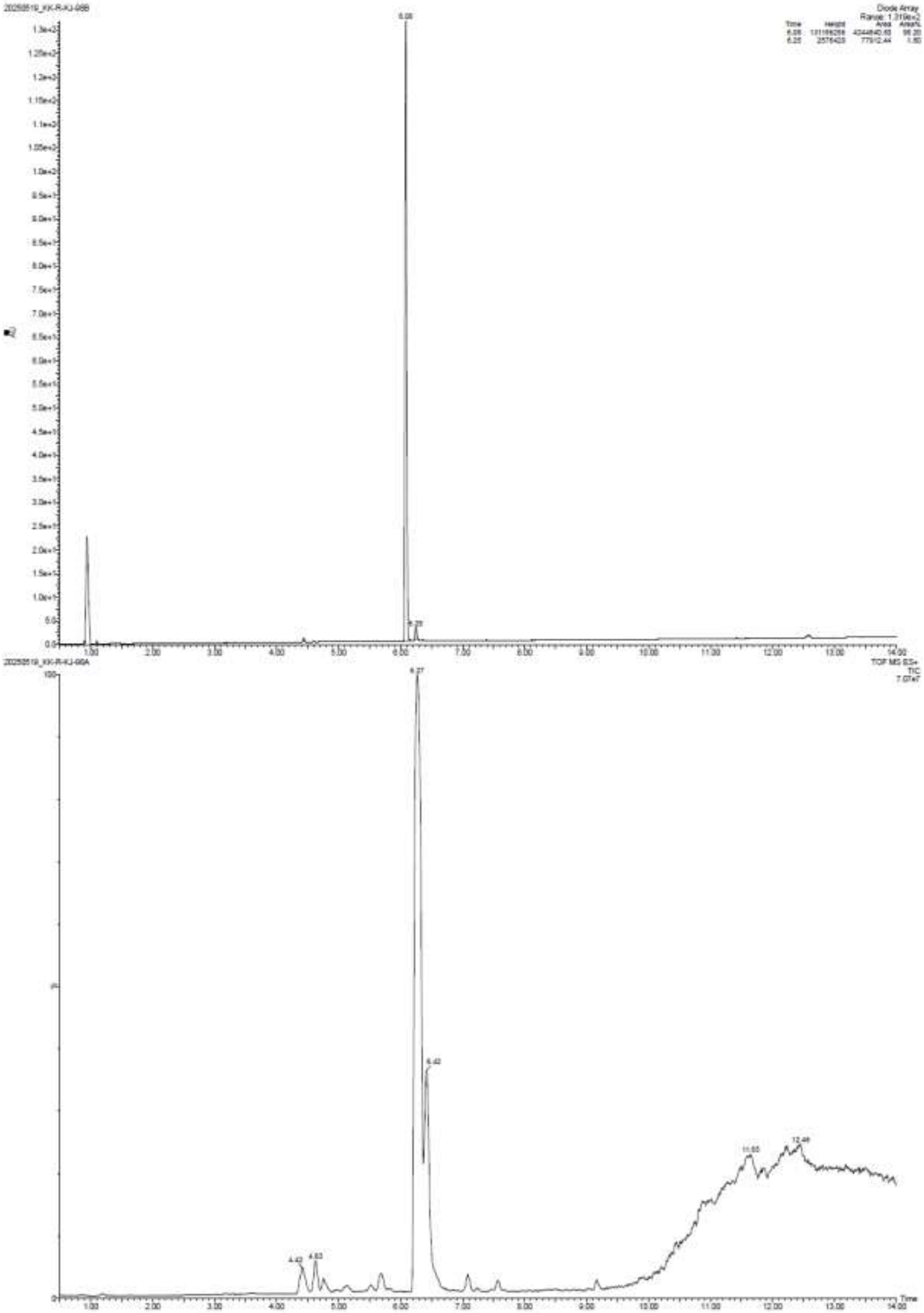

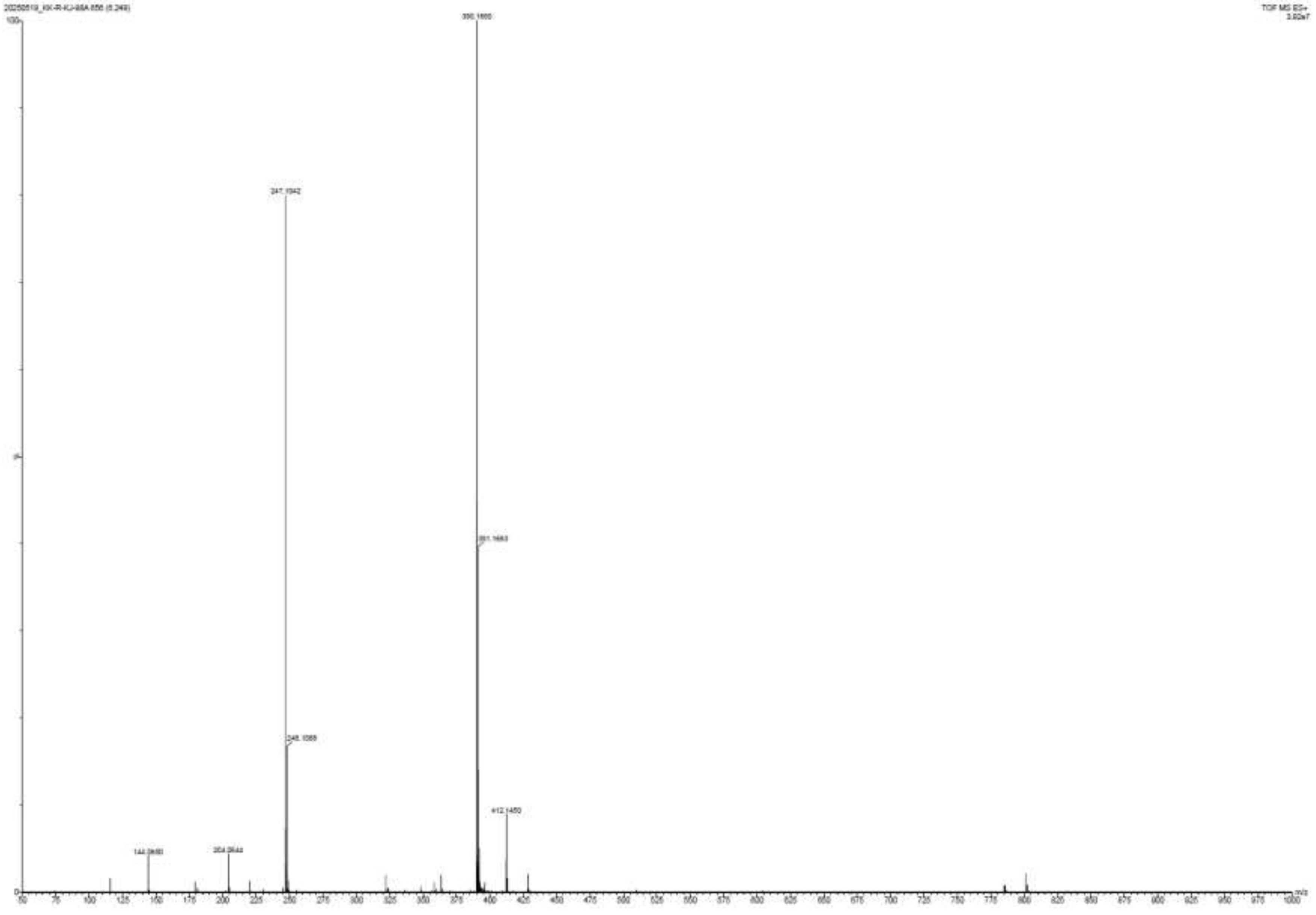
TOF MS ES+
247.1942
412.1450

(*S*)-*N*-(3-methoxy-1-oxo-1-(4-(3-(trifluoromethoxy)phenyl)piperazin-1-yl)propan-2-yl)acetamide (*S*)-46

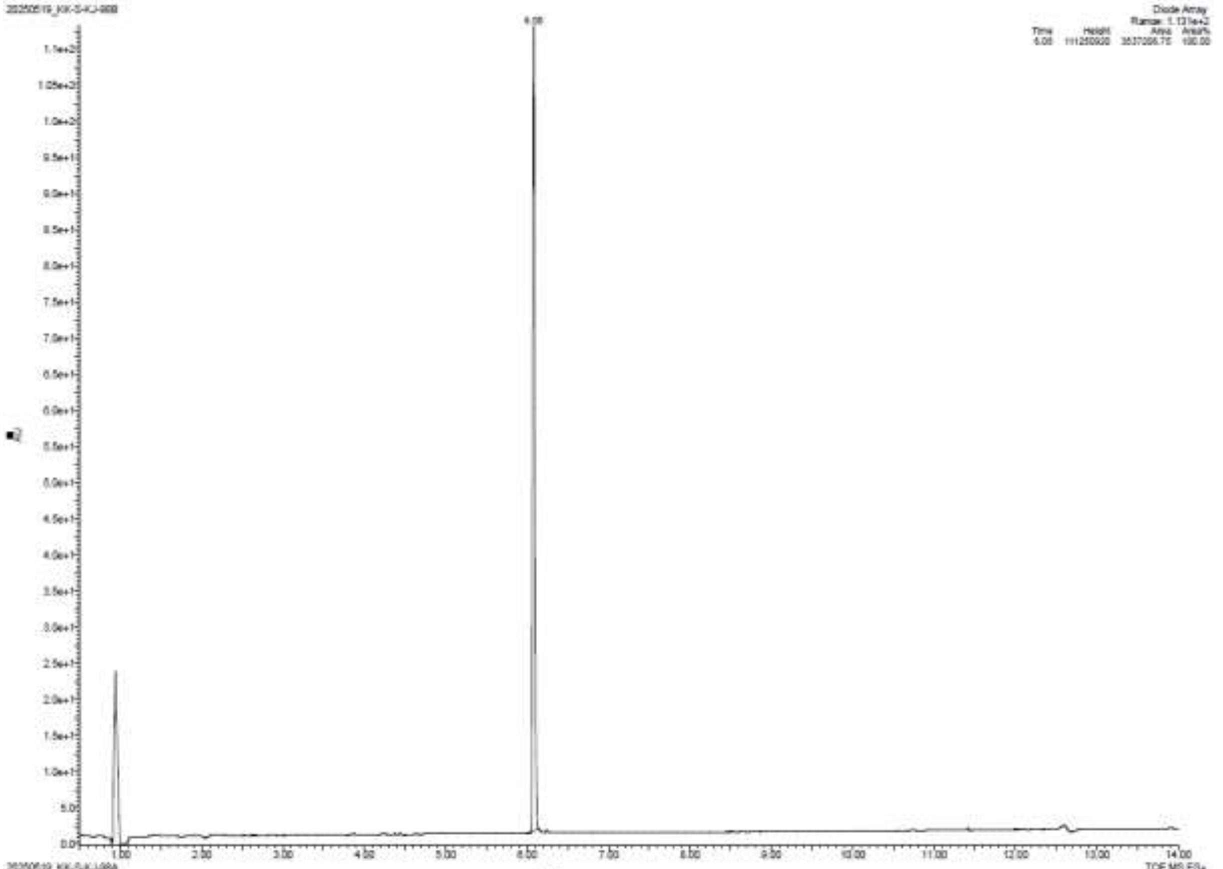

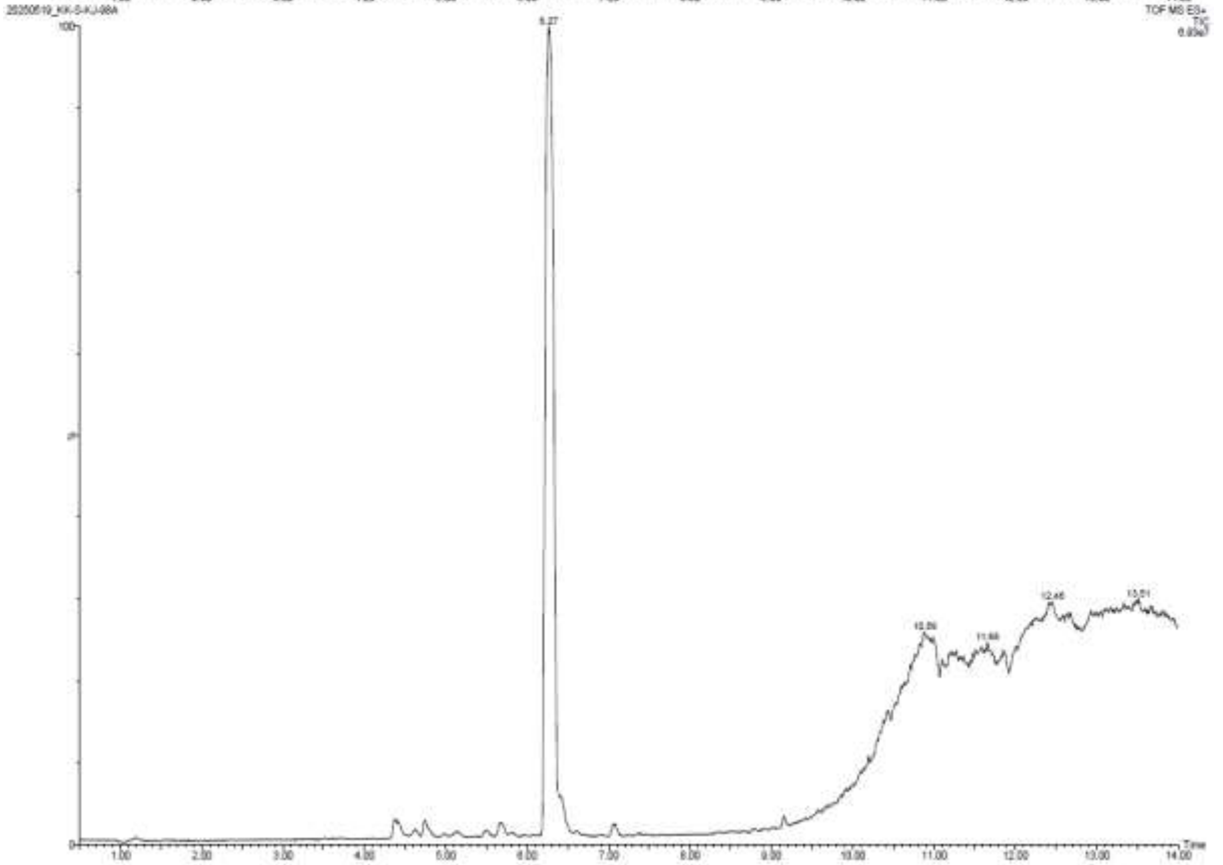

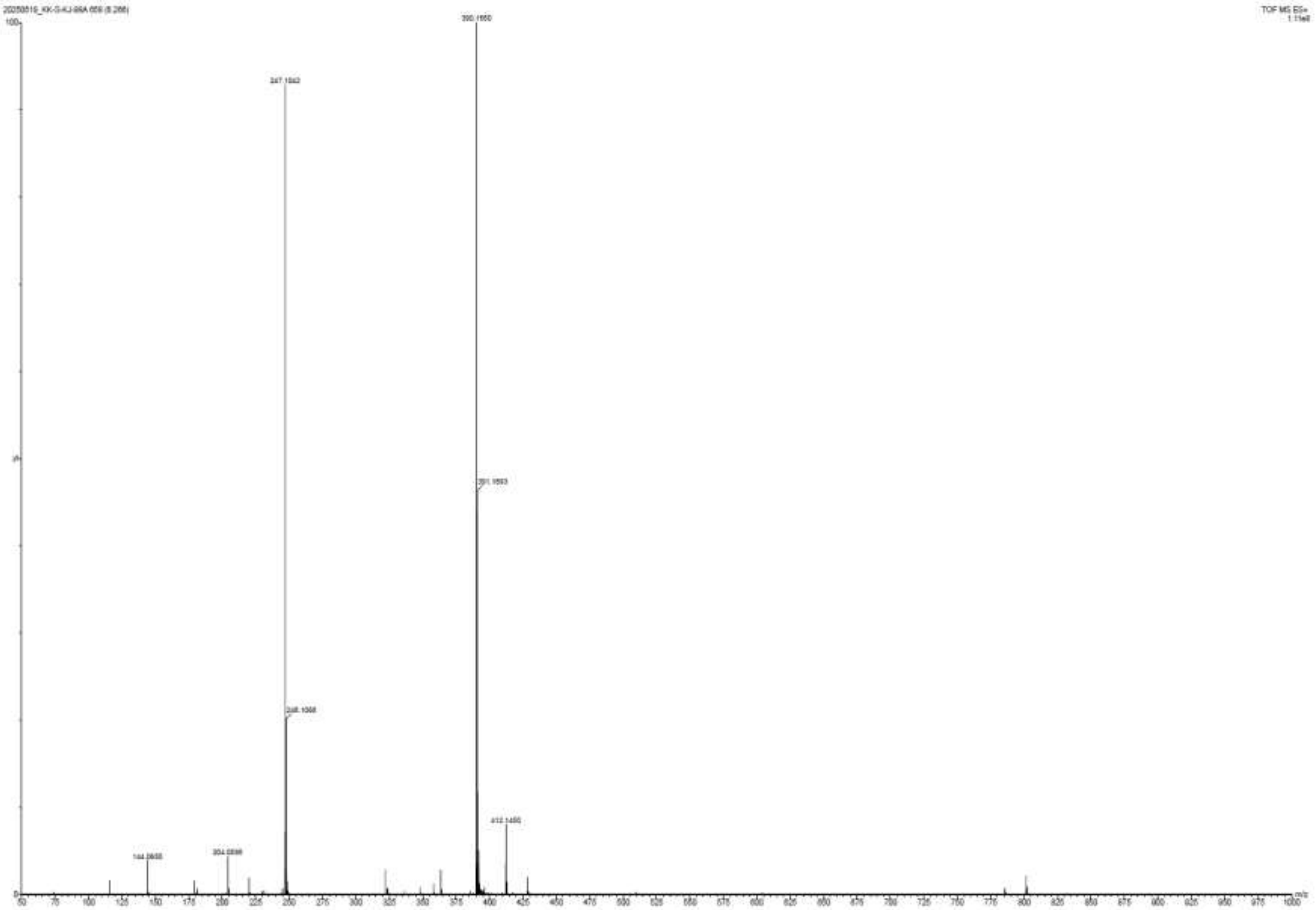
TOF MS ES+
m/z

(*R*)-*N*-(3-methoxy-1-oxo-1-(4-(3-((trifluoromethyl)thio)phenyl)piperazin-1-yl)propan-2-yl)acetamide (*R*)-50

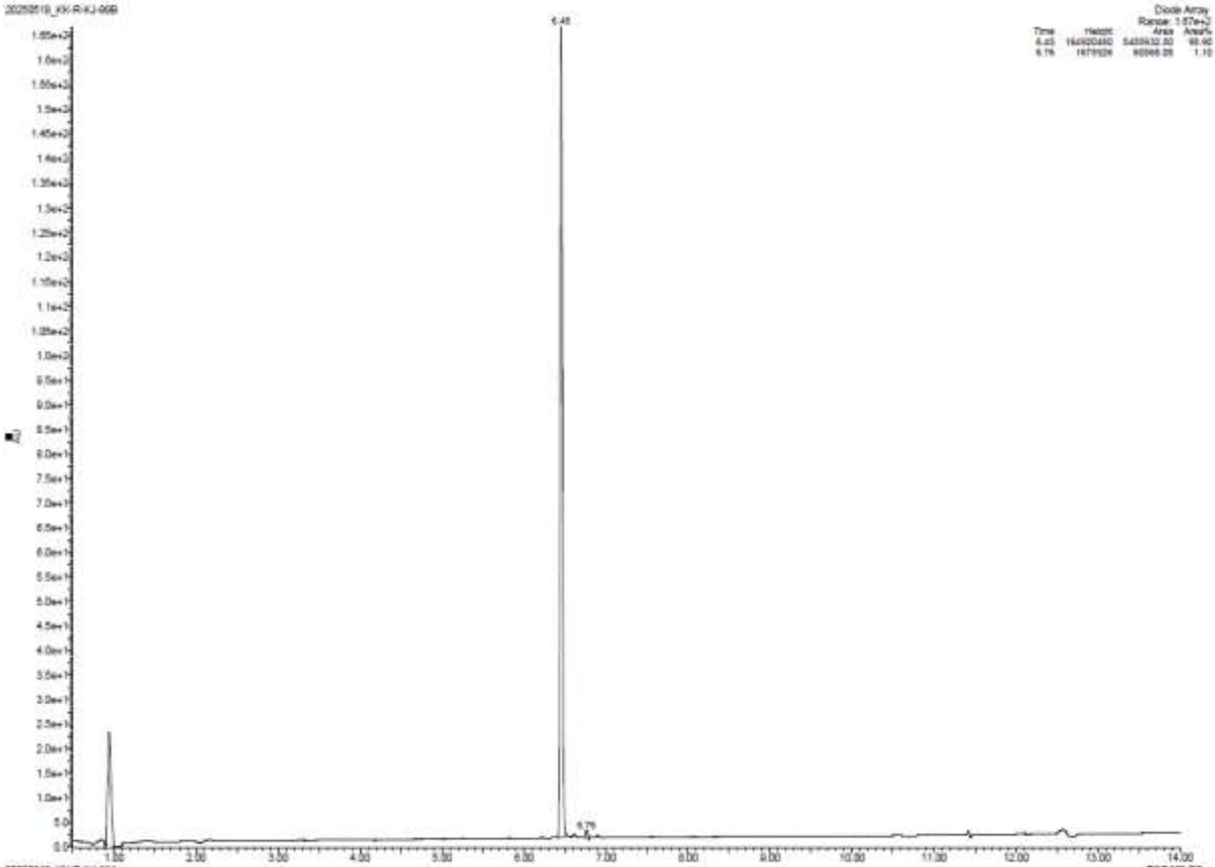

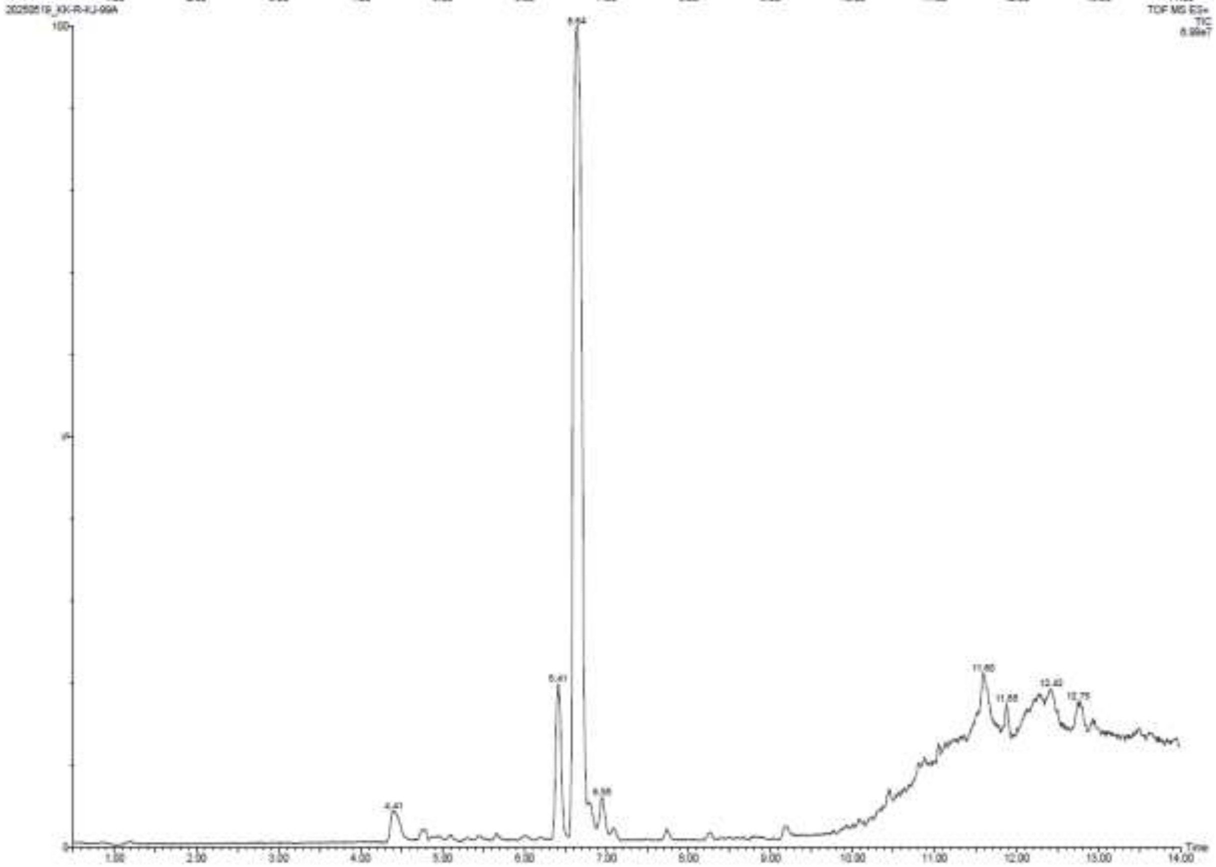

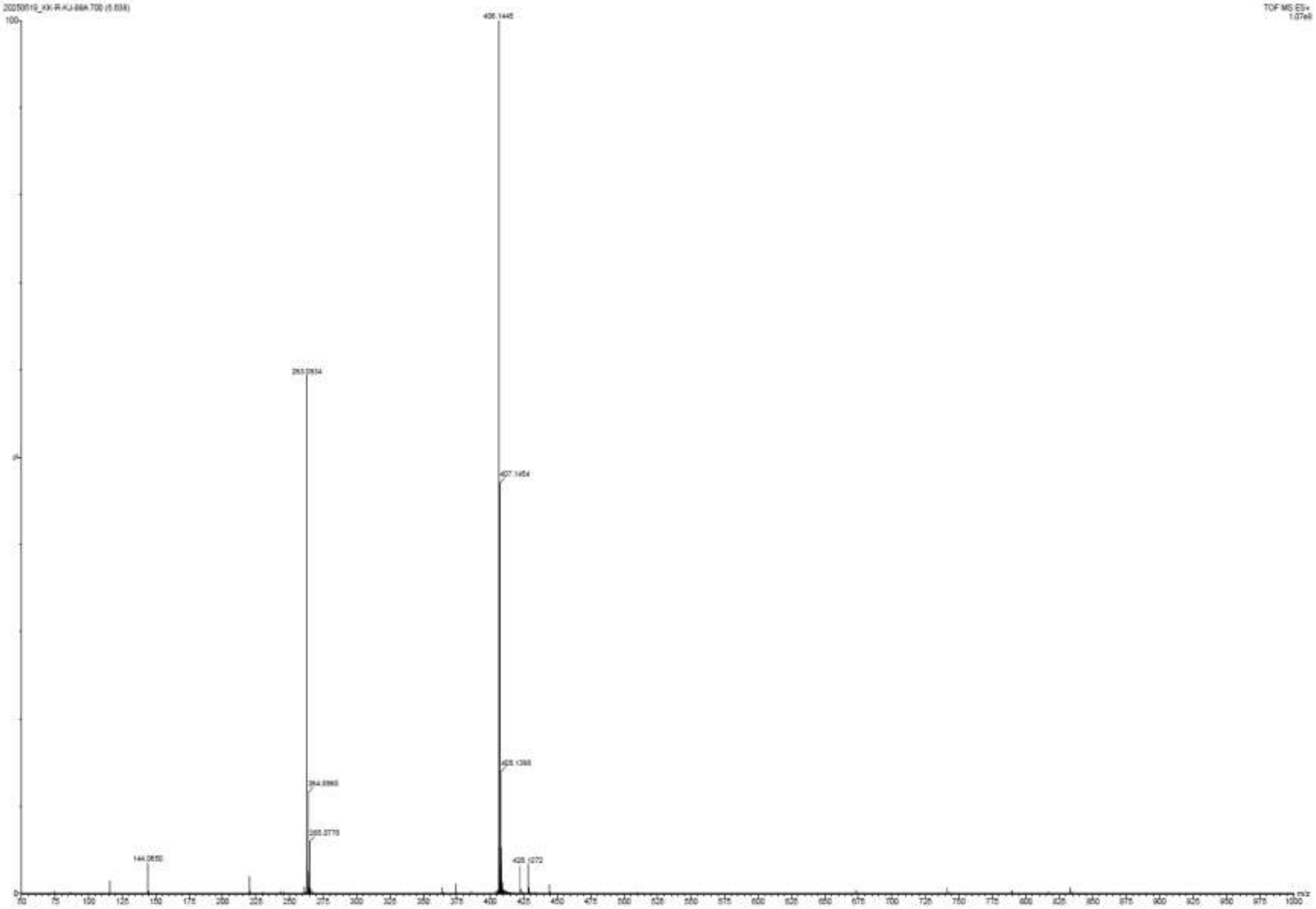

20250519_KK-R-KJ-88A 700 (6.838)
TOF MS ES+
1.07e8
406.1446
263.0834
407.1454
428.1268
264.0960
265.0770
144.0650
m/z

(*S*)-*N*-(3-methoxy-1-oxo-1-(4-(3-((trifluoromethyl)thio)phenyl)piperazin-1-yl)propan-2-yl)acetamide (*S*)-50

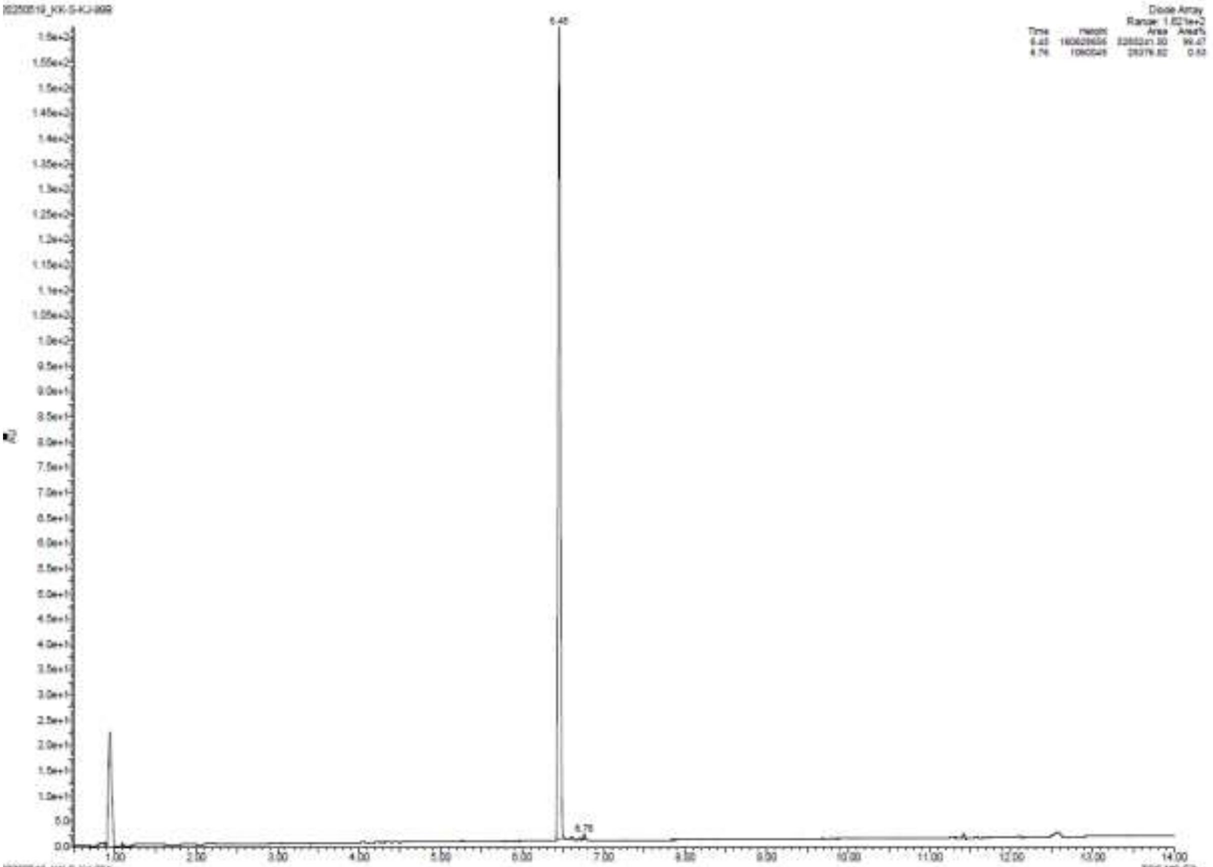

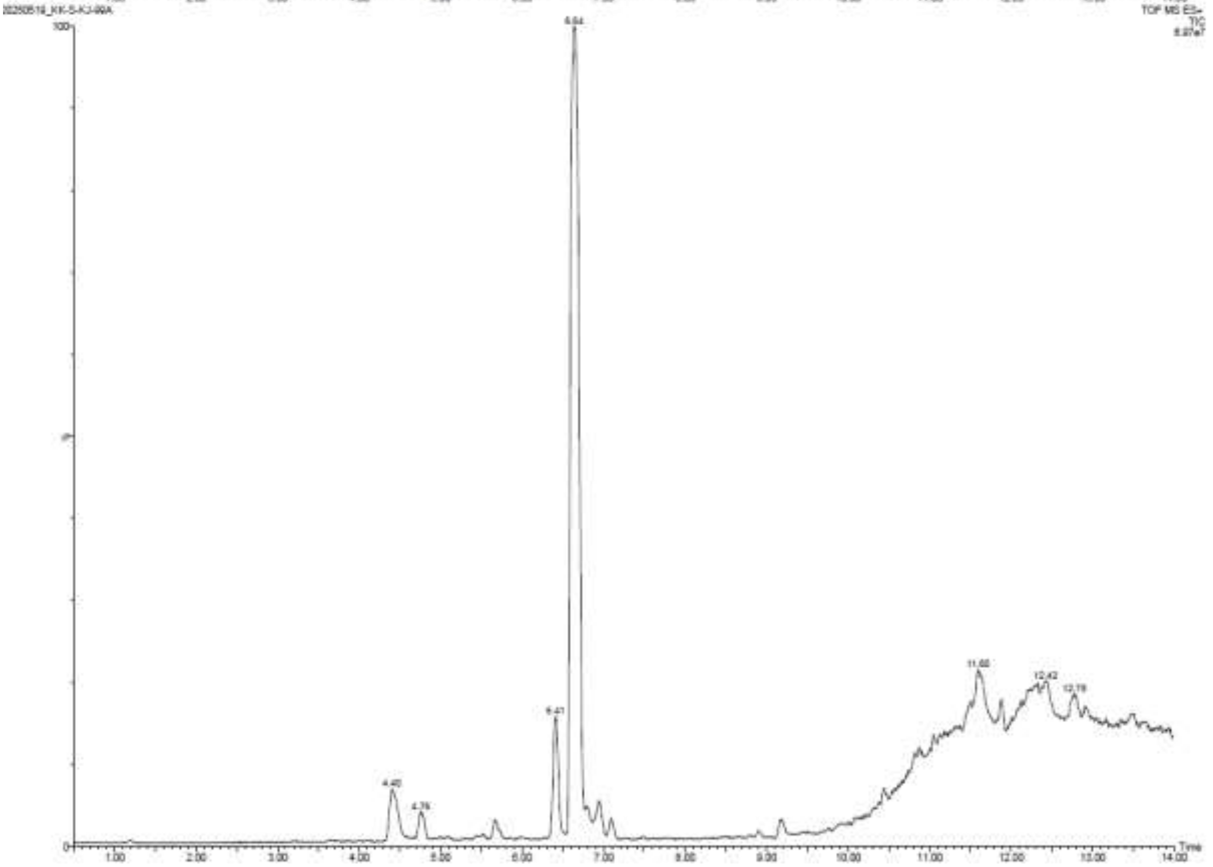

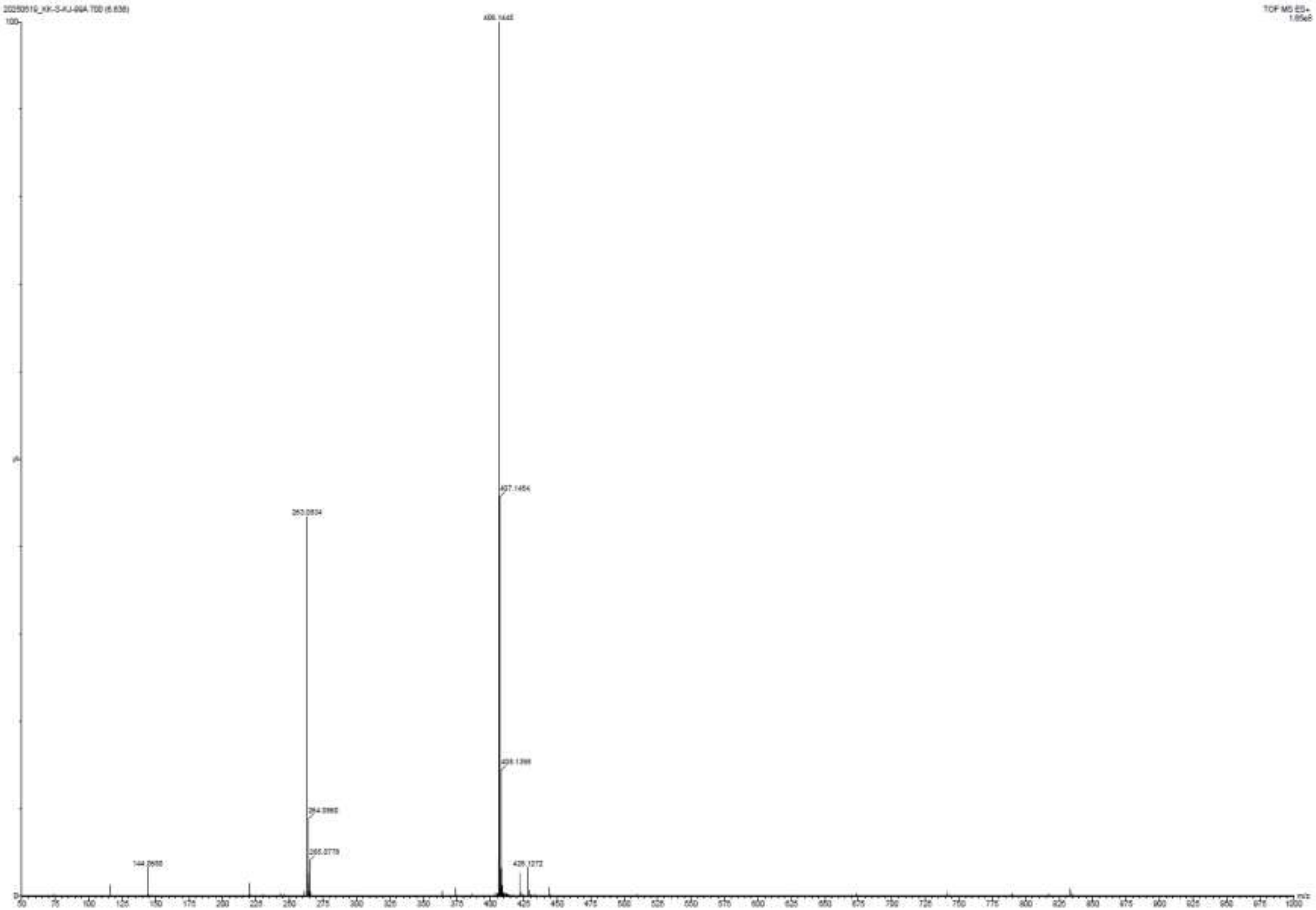

TOF MS ES+
407.1454
428.1272
m/z

^{1}H NMR and ^{13}C NMR spectra for the final compounds

(*R*,*S*)-*N*-(3-methoxy-1-oxo-1-(4-phenylpiperazin-1-yl)propan-2-yl)acetamide (*R*,*S*)-35 – ^{1}H NMR

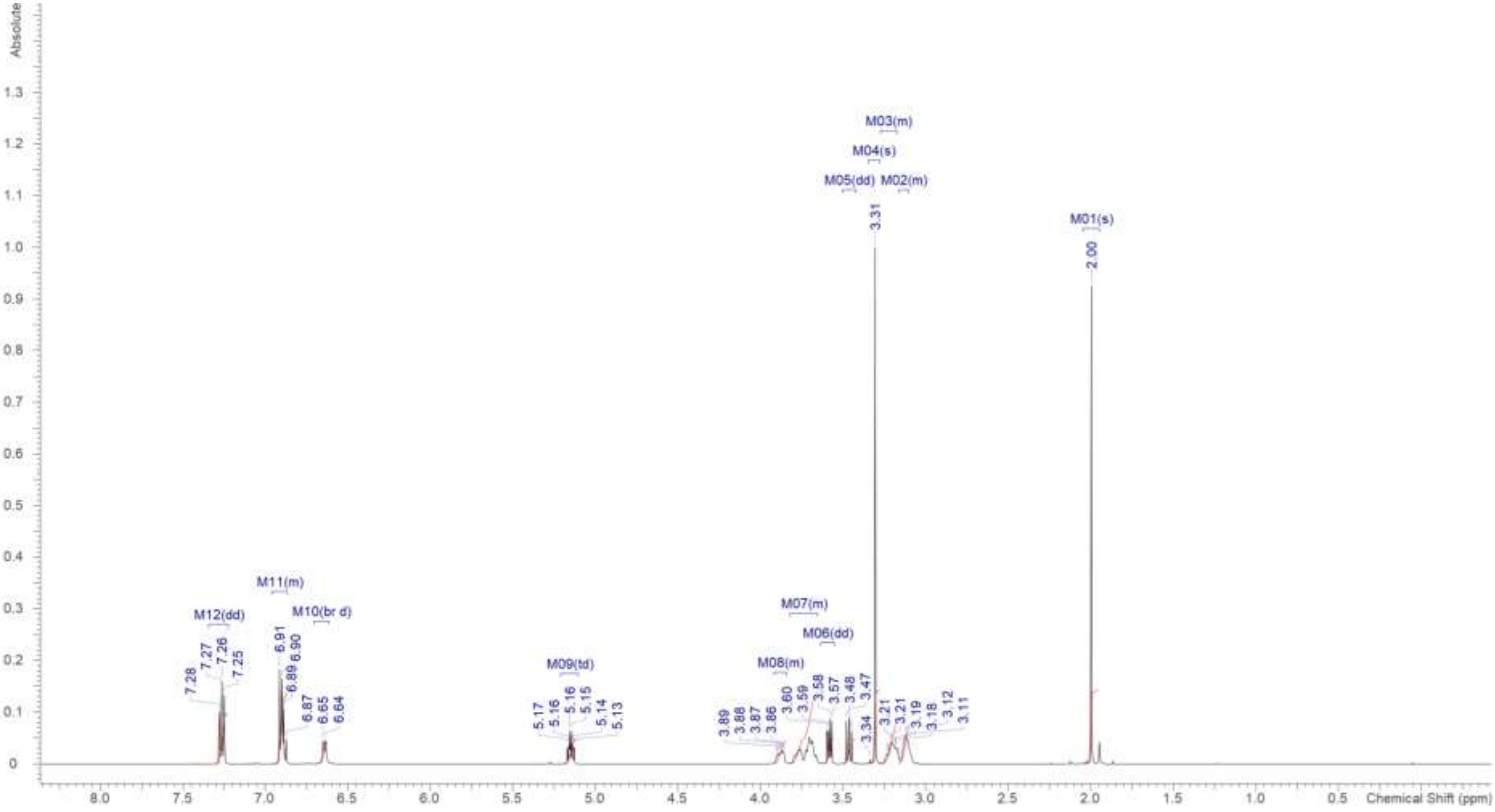

(*R*,*S*)-*N*-(3-methoxy-1-oxo-1-(4-phenylpiperazin-1-yl)propan-2-yl)acetamide (*R*,*S*)-35 – ^{13}C NMR

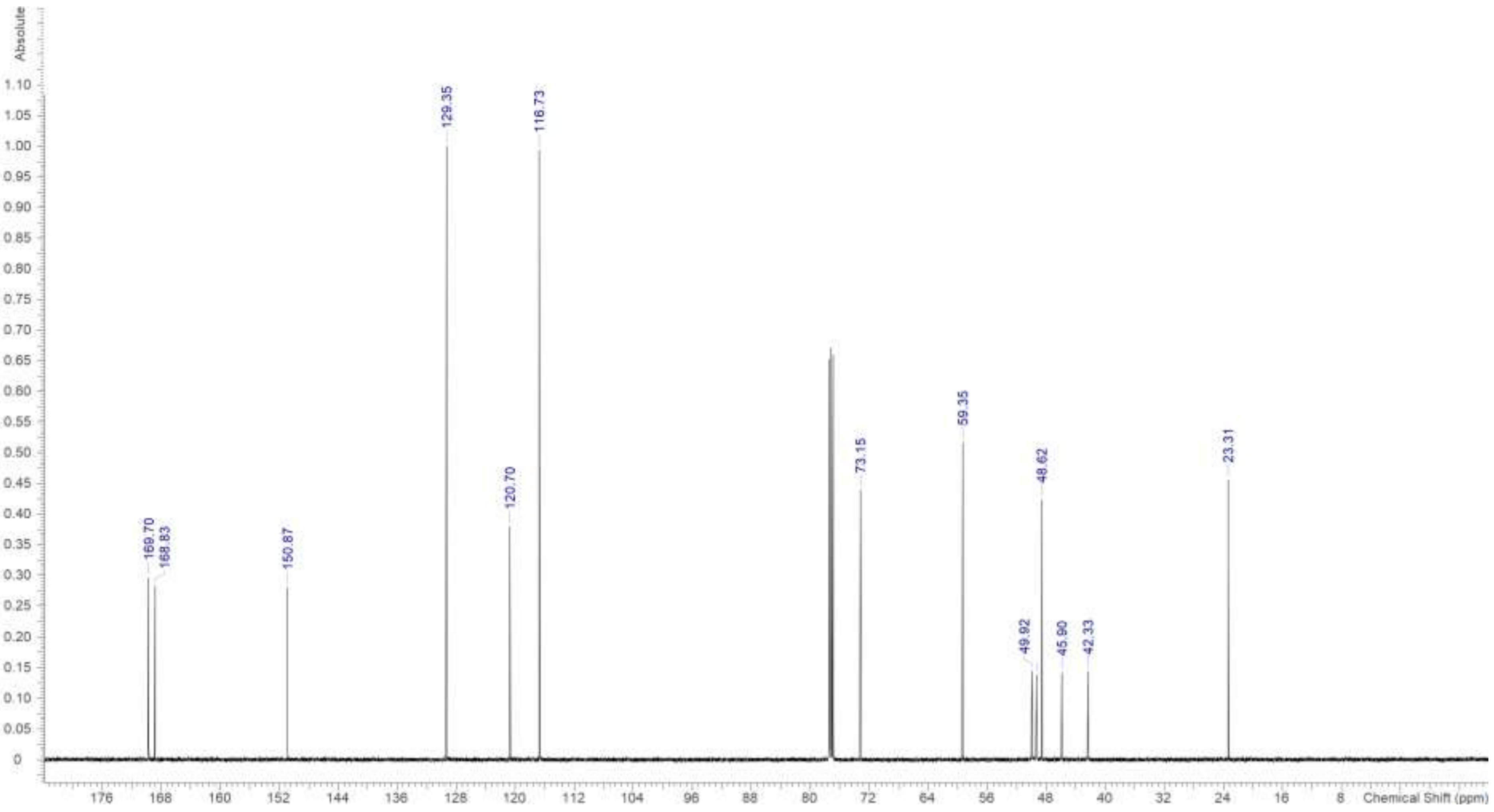

(*R*,*S*)-*N*-(1-(4-(3-fluorophenyl)piperazin-1-yl)-3-methoxy-1-oxopropan-2-yl)acetamide (*R*,*S*)-36 – ^{1}H NMR

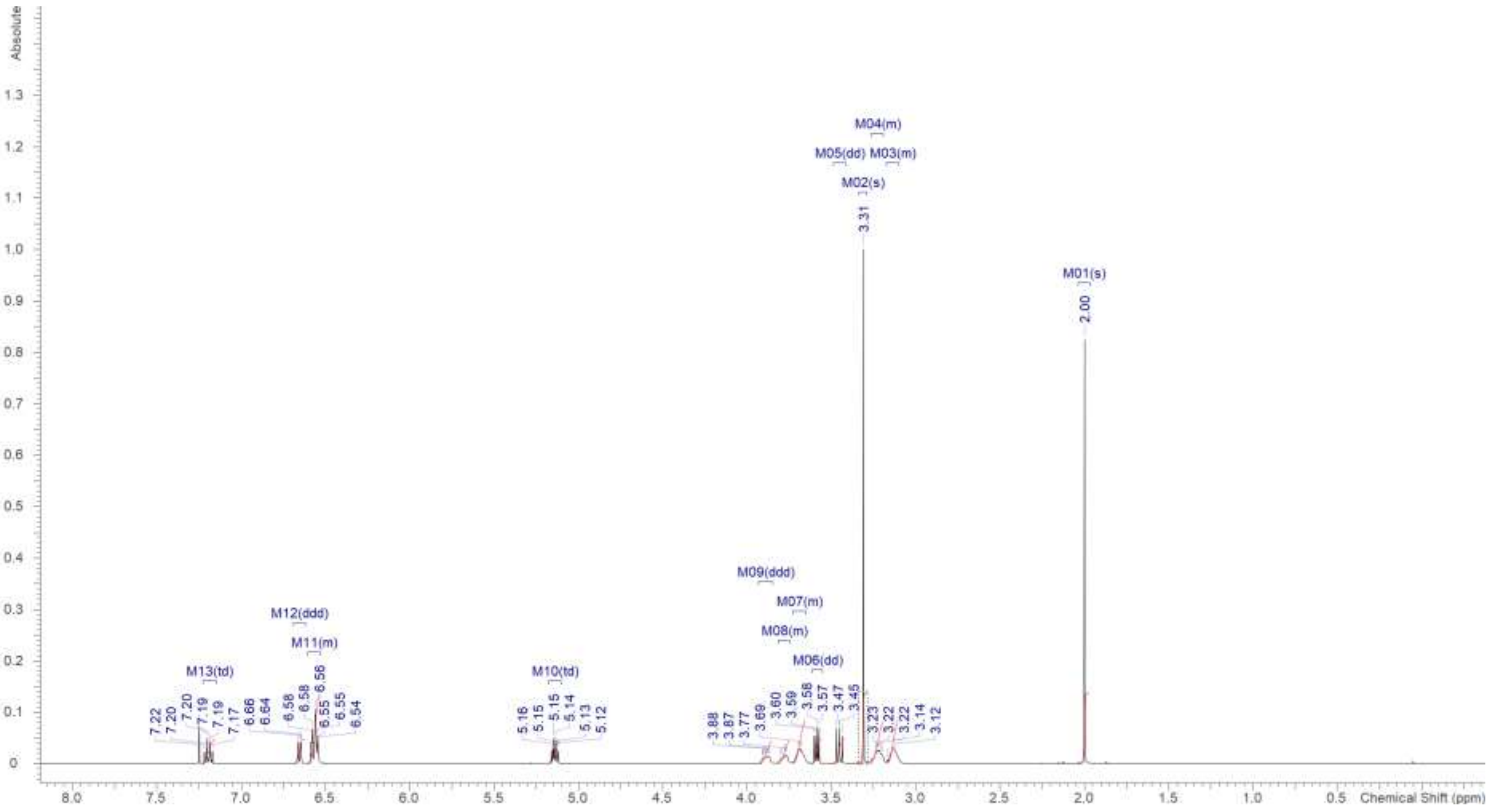

(*R*,*S*)-*N*-(1-(4-(3-fluorophenyl)piperazin-1-yl)-3-methoxy-1-oxopropan-2-yl)acetamide (*R*,*S*)-36 – ^{13}C NMR

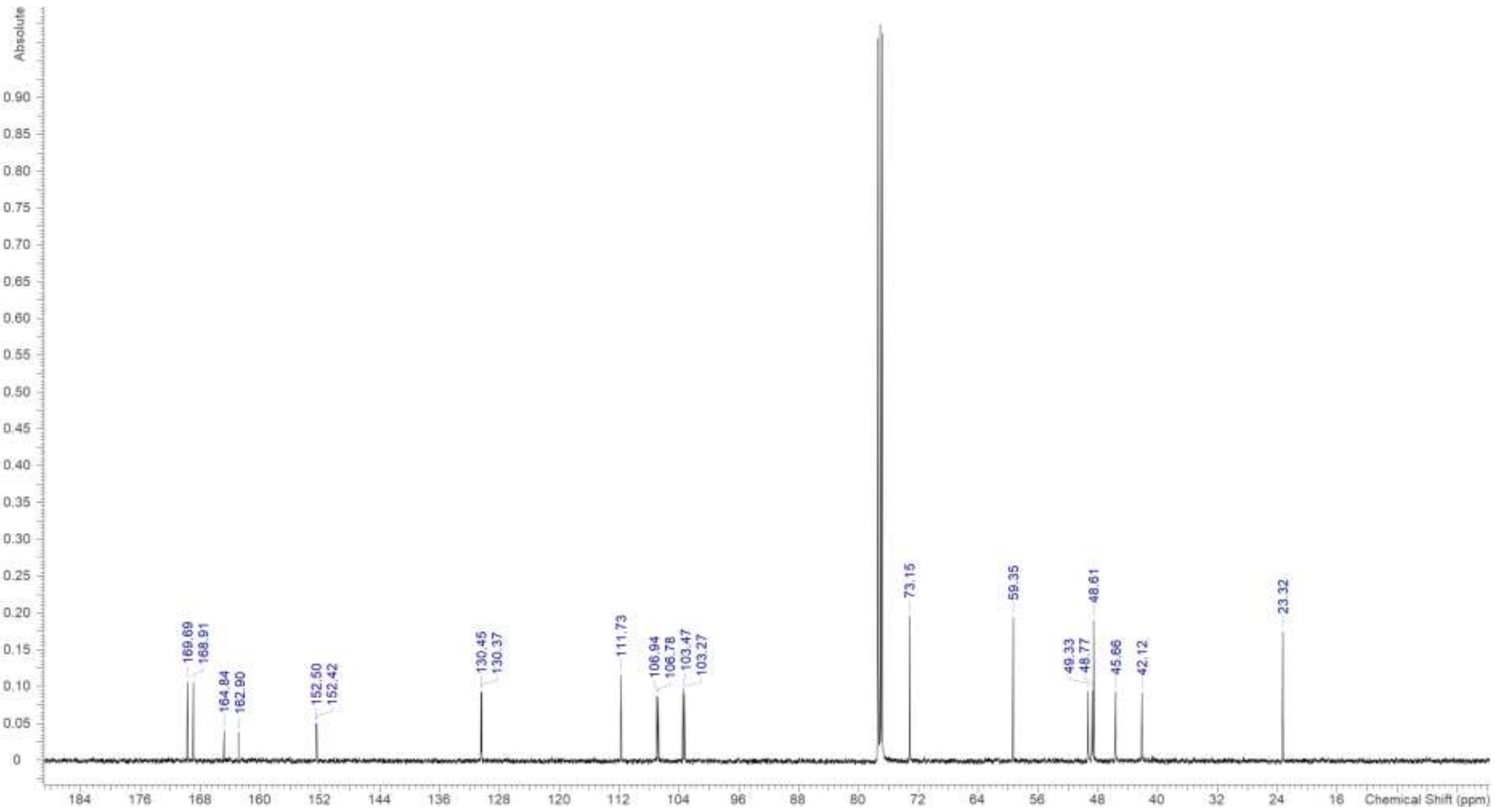

(*R*,*S*)-*N*-(1-(4-(4-fluorophenyl)piperazin-1-yl)-3-methoxy-1-oxopropan-2-yl)acetamide (*R*,*S*)-37 – ^{1}H NMR

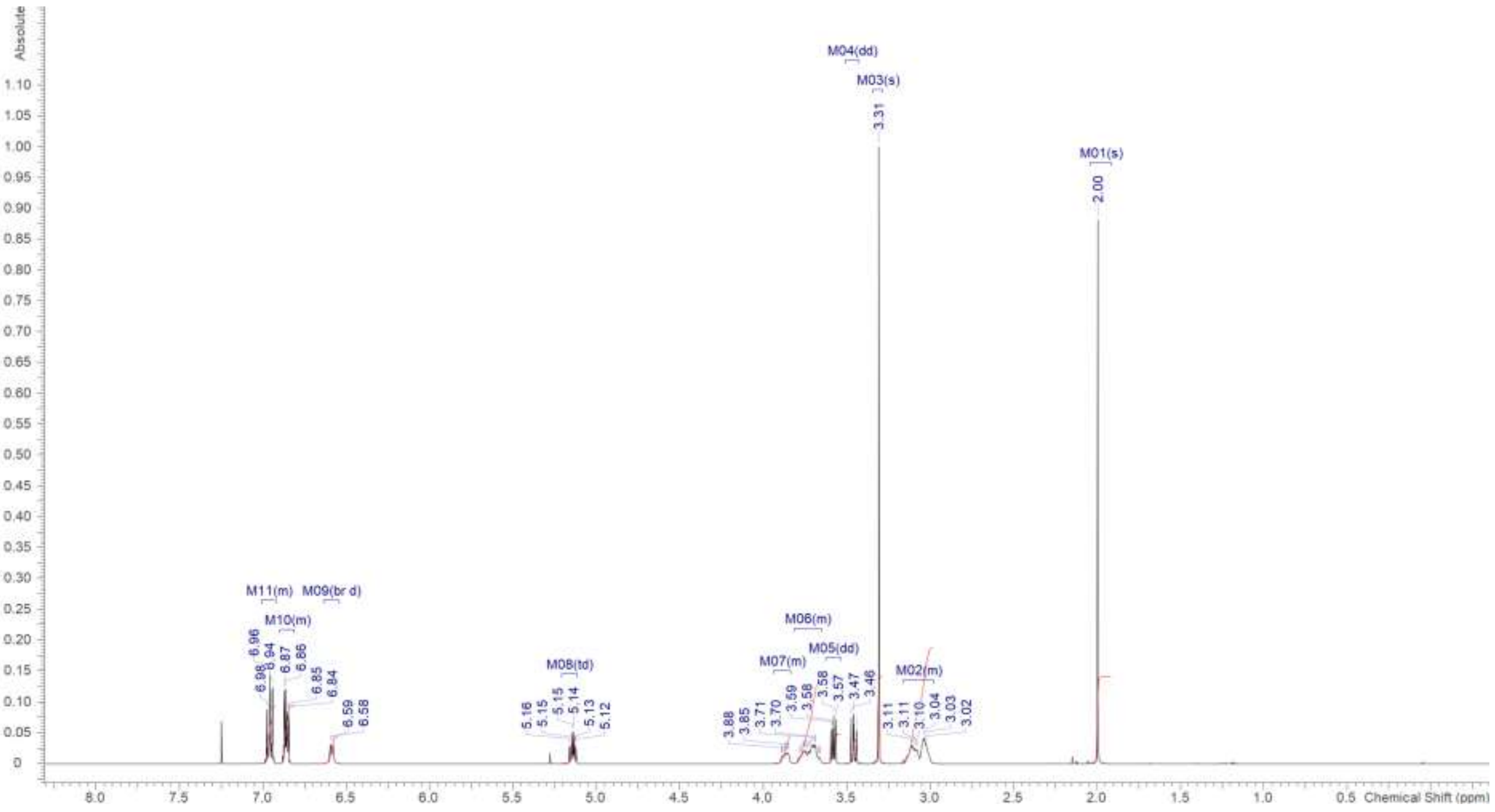

(*R,S*)-*N*-(1-(4-(4-fluorophenyl)piperazin-1-yl)-3-methoxy-1-oxopropan-2-yl)acetamide (*R,S*)-37 – ^{13}C NMR

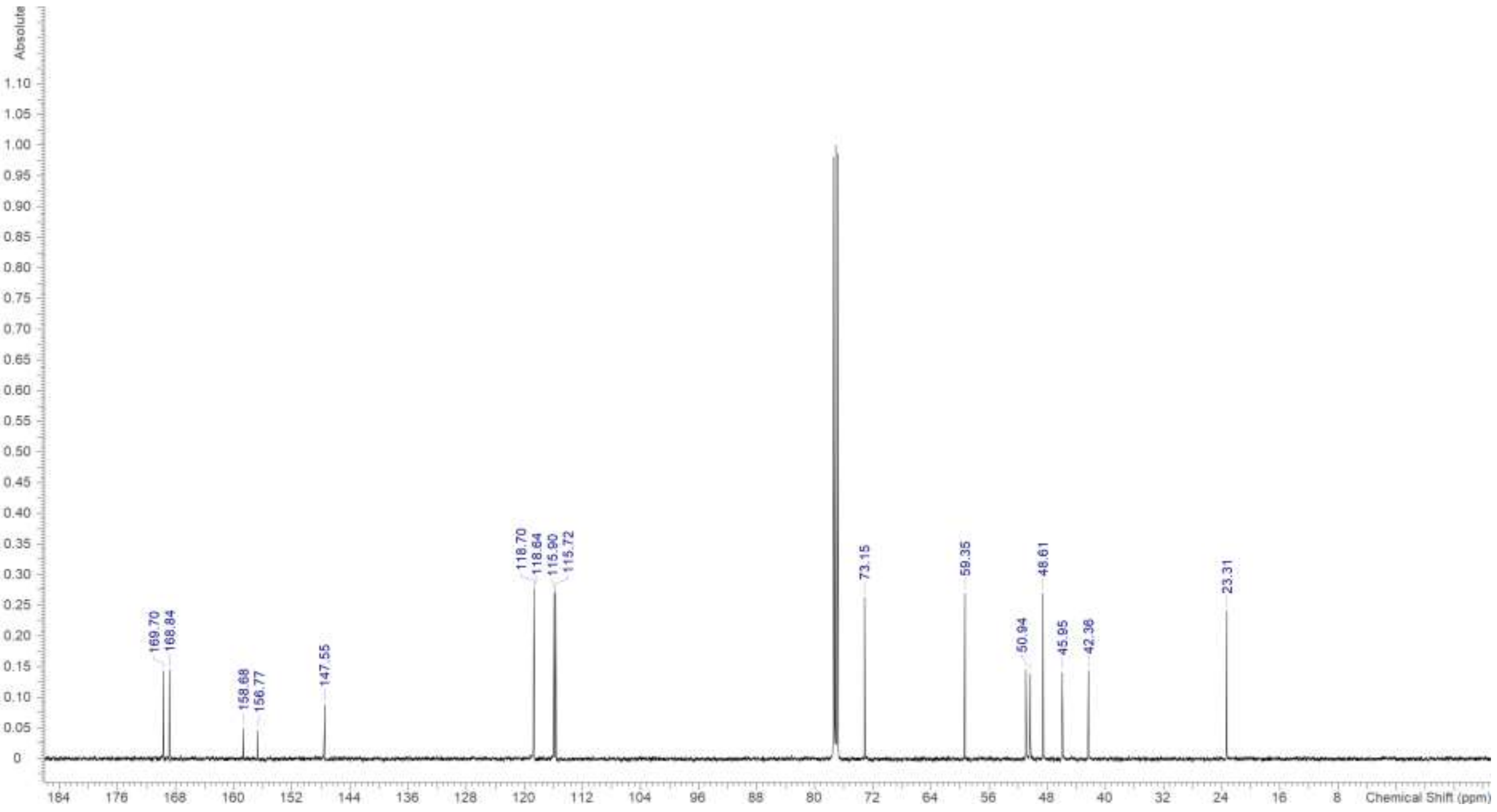

(*R*,*S*)-*N*-(1-(4-(3-chlorophenyl)piperazin-1-yl)-3-methoxy-1-oxopropan-2-yl)acetamide (*R*,*S*)-38 – ^{1}H NMR

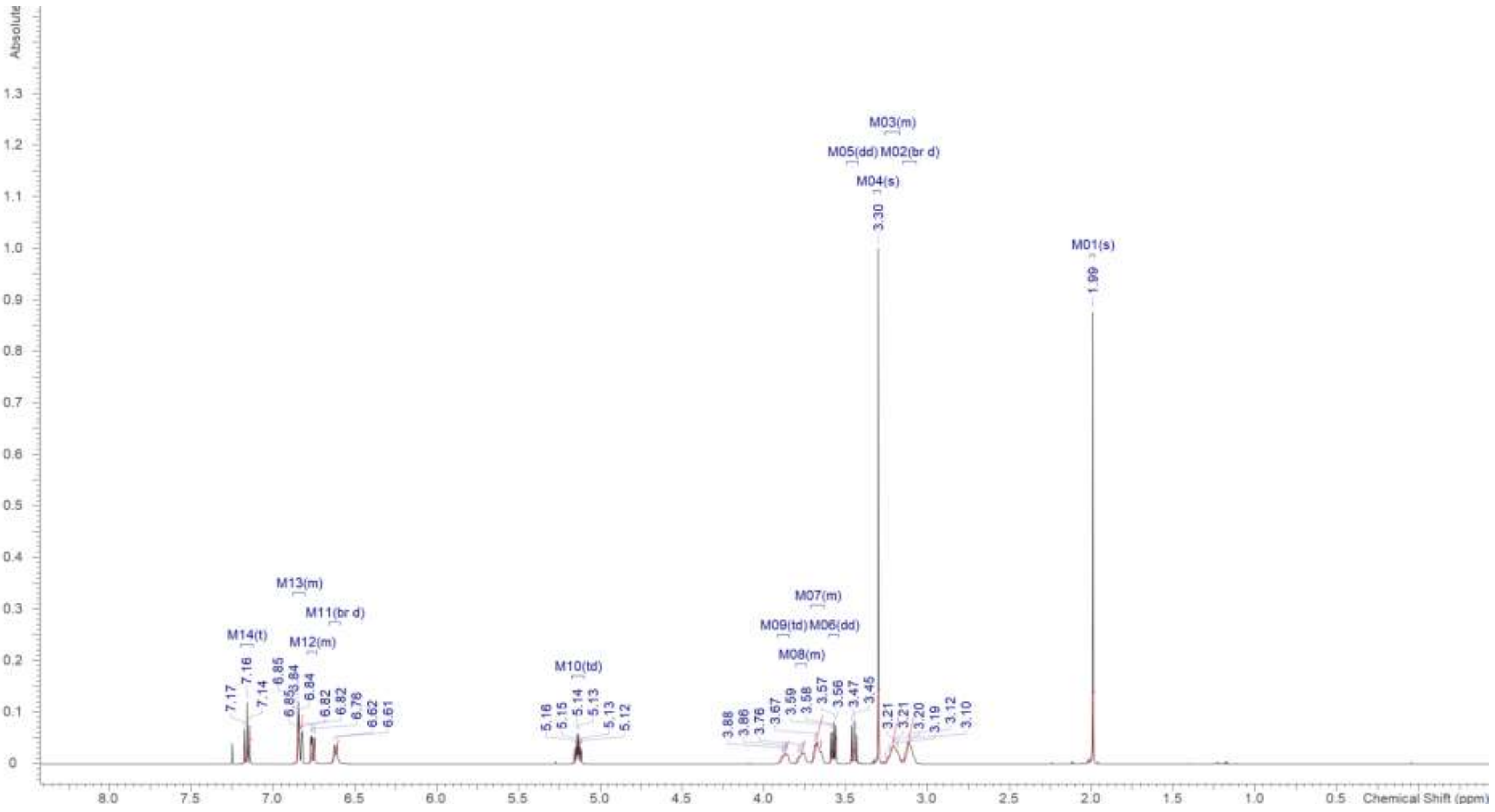

(*R,S*)-*N*-(1-(4-(3-chlorophenyl)piperazin-1-yl)-3-methoxy-1-oxopropan-2-yl)acetamide (*R,S*)-38 – ^{13}C NMR

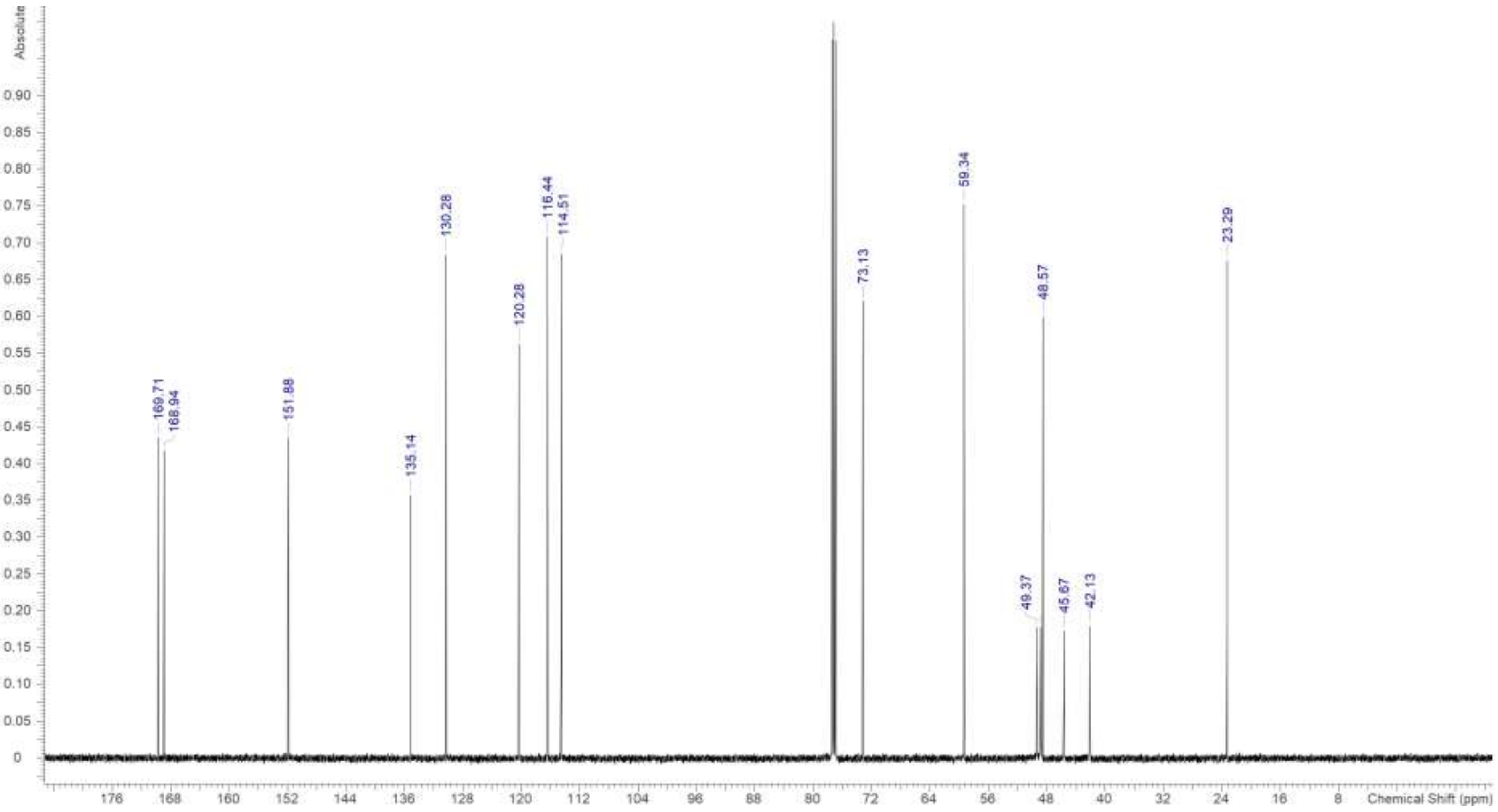

(*R*,*S*)-*N*-(1-(4-(4-chlorophenyl)piperazin-1-yl)-3-methoxy-1-oxopropan-2-yl)acetamide (*R*,*S*)-39 – ^{1}H NMR

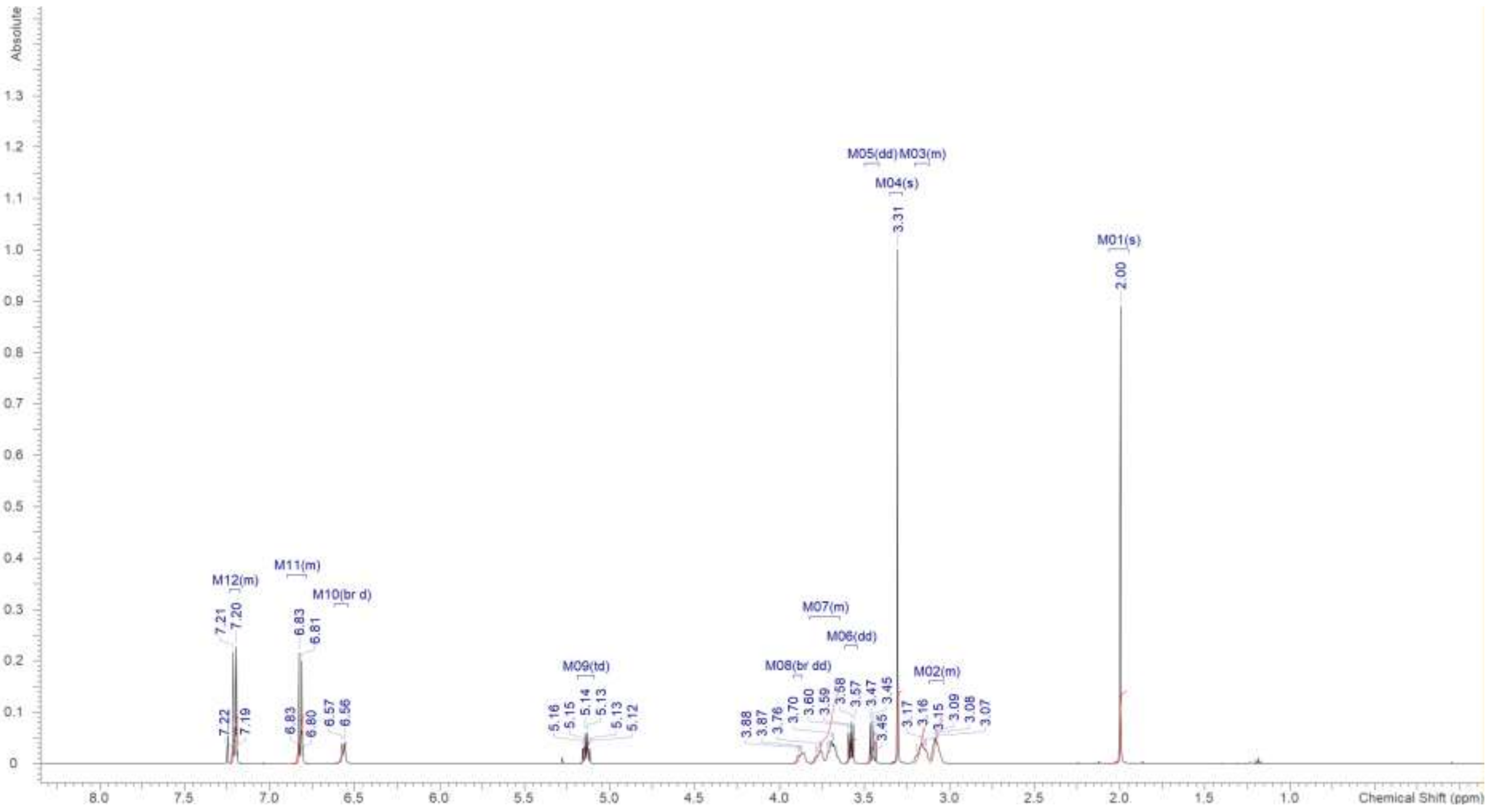

(*R,S*)-*N*-(1-(4-(4-chlorophenyl)piperazin-1-yl)-3-methoxy-1-oxopropan-2-yl)acetamide (*R,S*)-39 – ^{13}C NMR

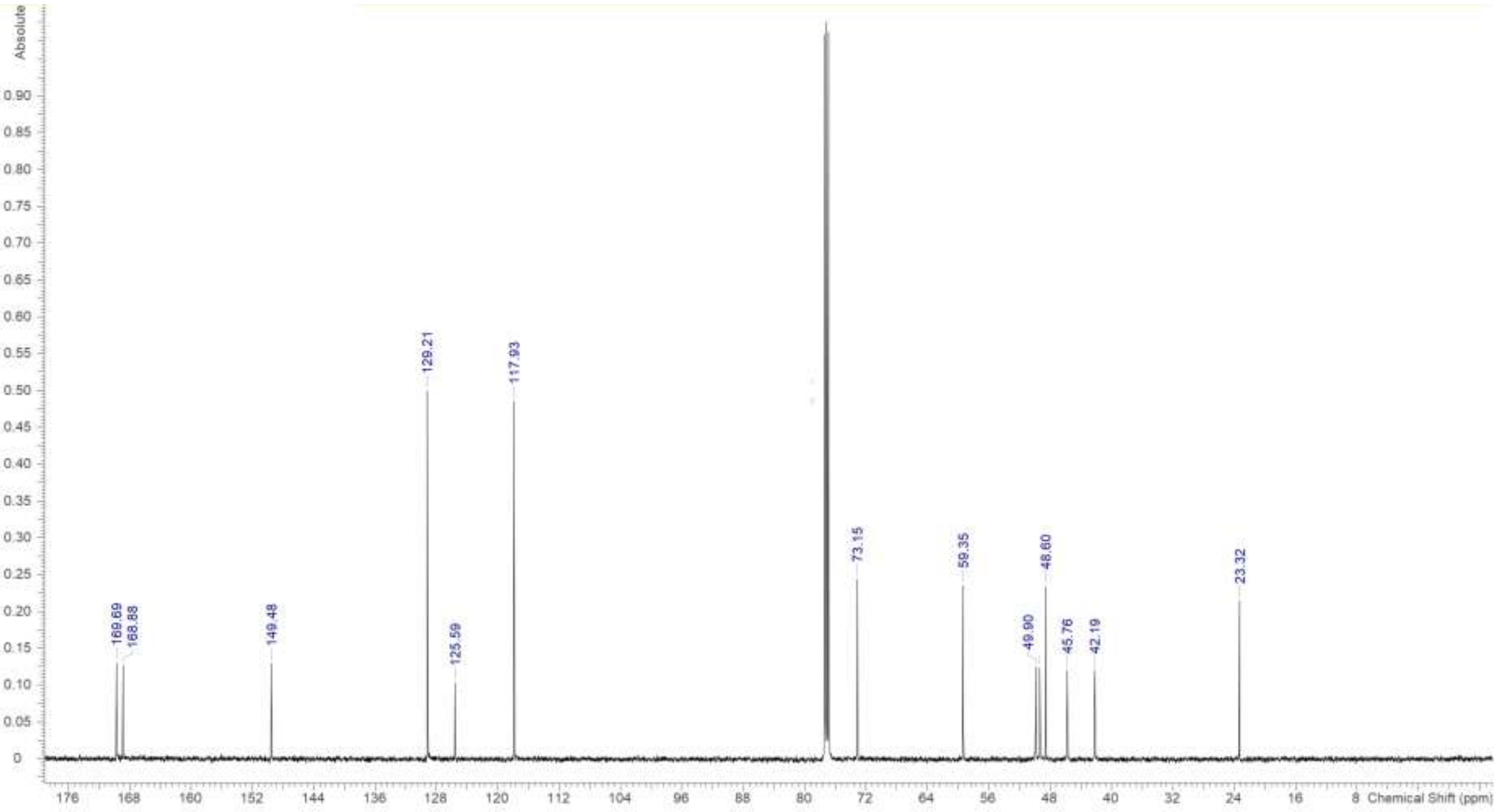

(*R*,*S*)-*N*-(1-(4-(3,4-dichlorophenyl)piperazin-1-yl)-3-methoxy-1-oxopropan-2-yl)acetamide (*R*,*S*)-40 – ^{1}H NMR

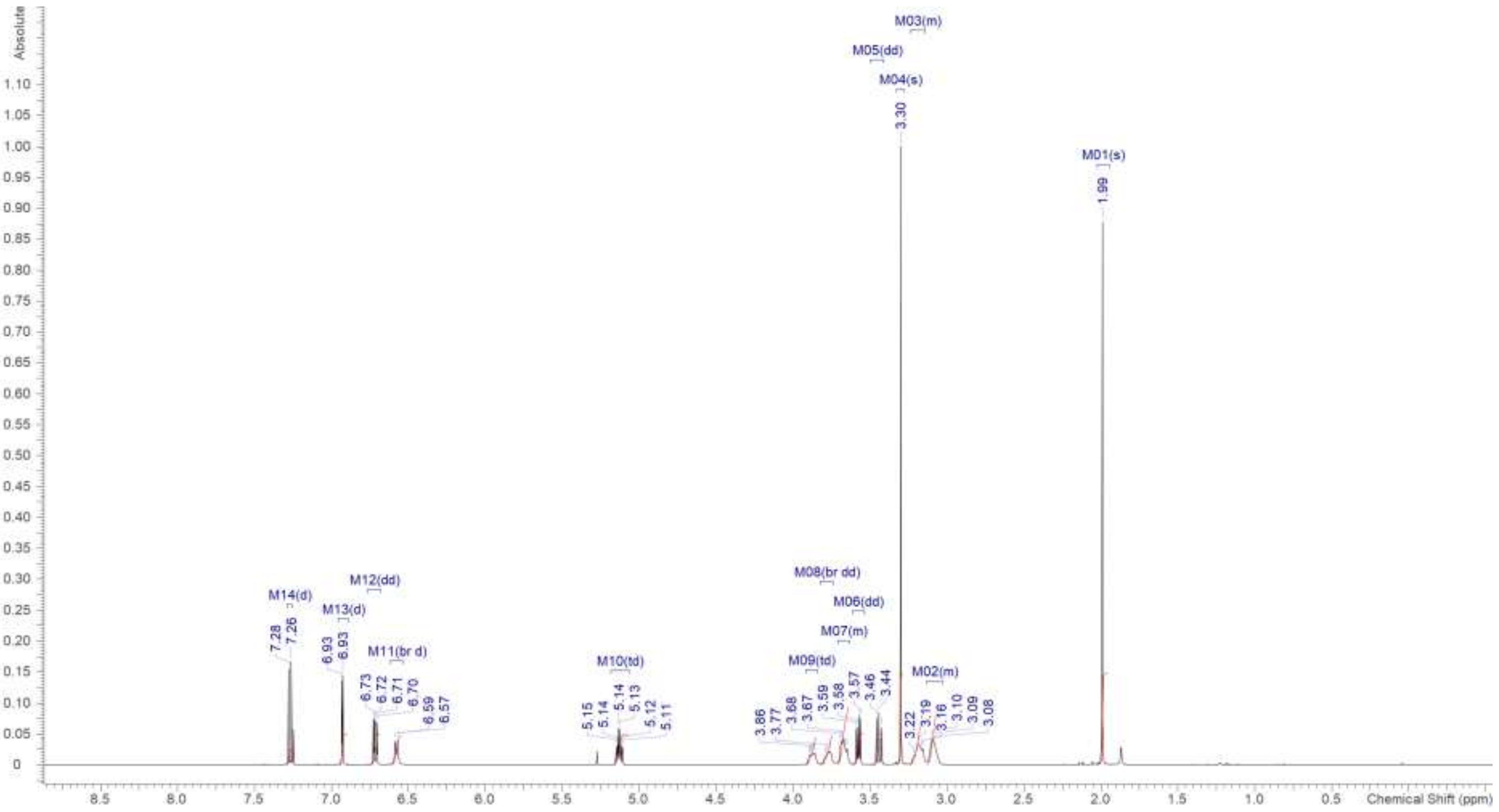

(*R*,*S*)-*N*-(1-(4-(3,4-dichlorophenyl)piperazin-1-yl)-3-methoxy-1-oxopropan-2-yl)acetamide (*R*,*S*)-40 – ^{13}C NMR

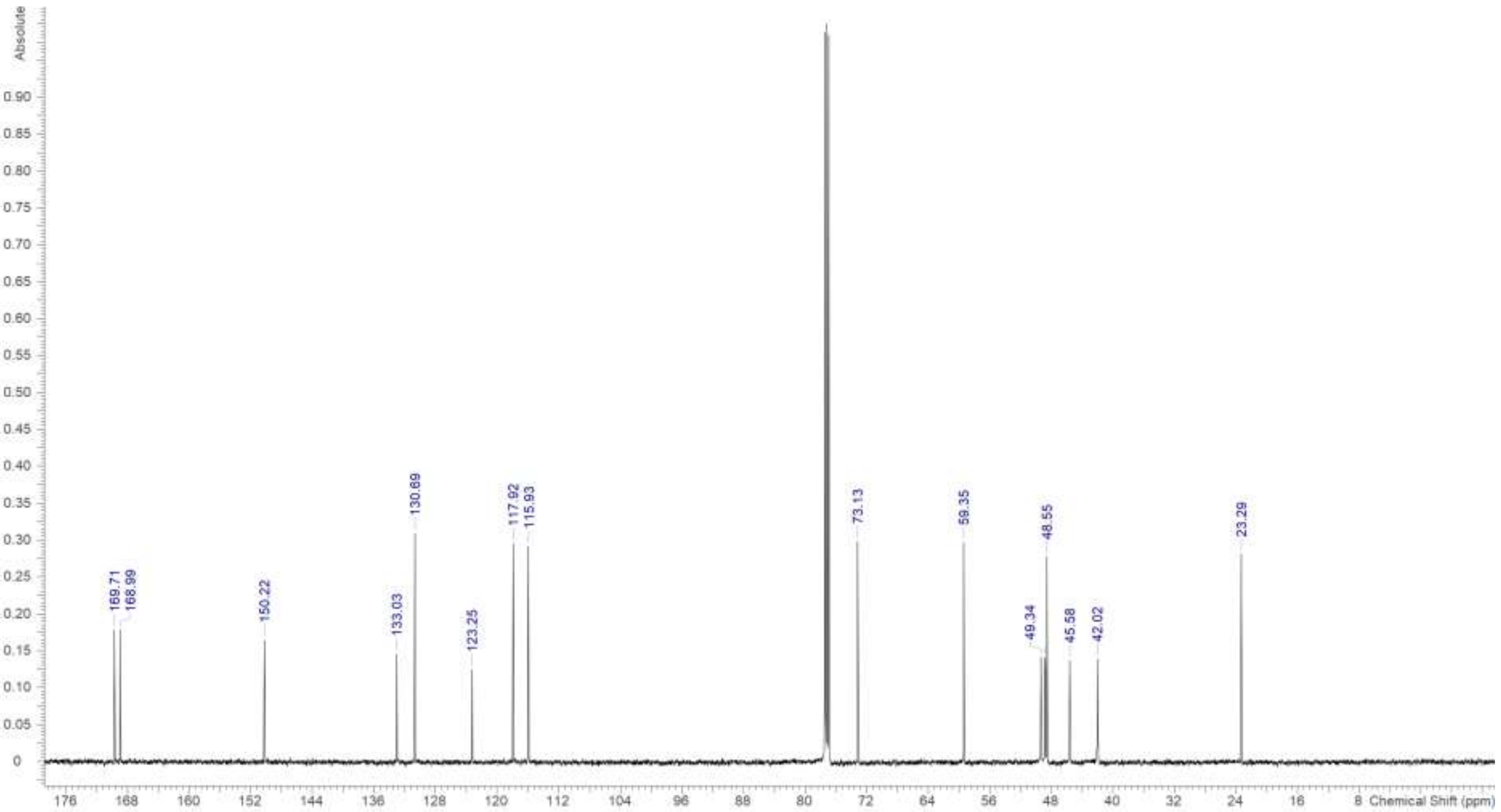

(*R*,*S*)-*N*-(1-(4-(3,5-dichlorophenyl)piperazin-1-yl)-3-methoxy-1-oxopropan-2-yl)acetamide (*R*,*S*)-41 – ^{1}H NMR

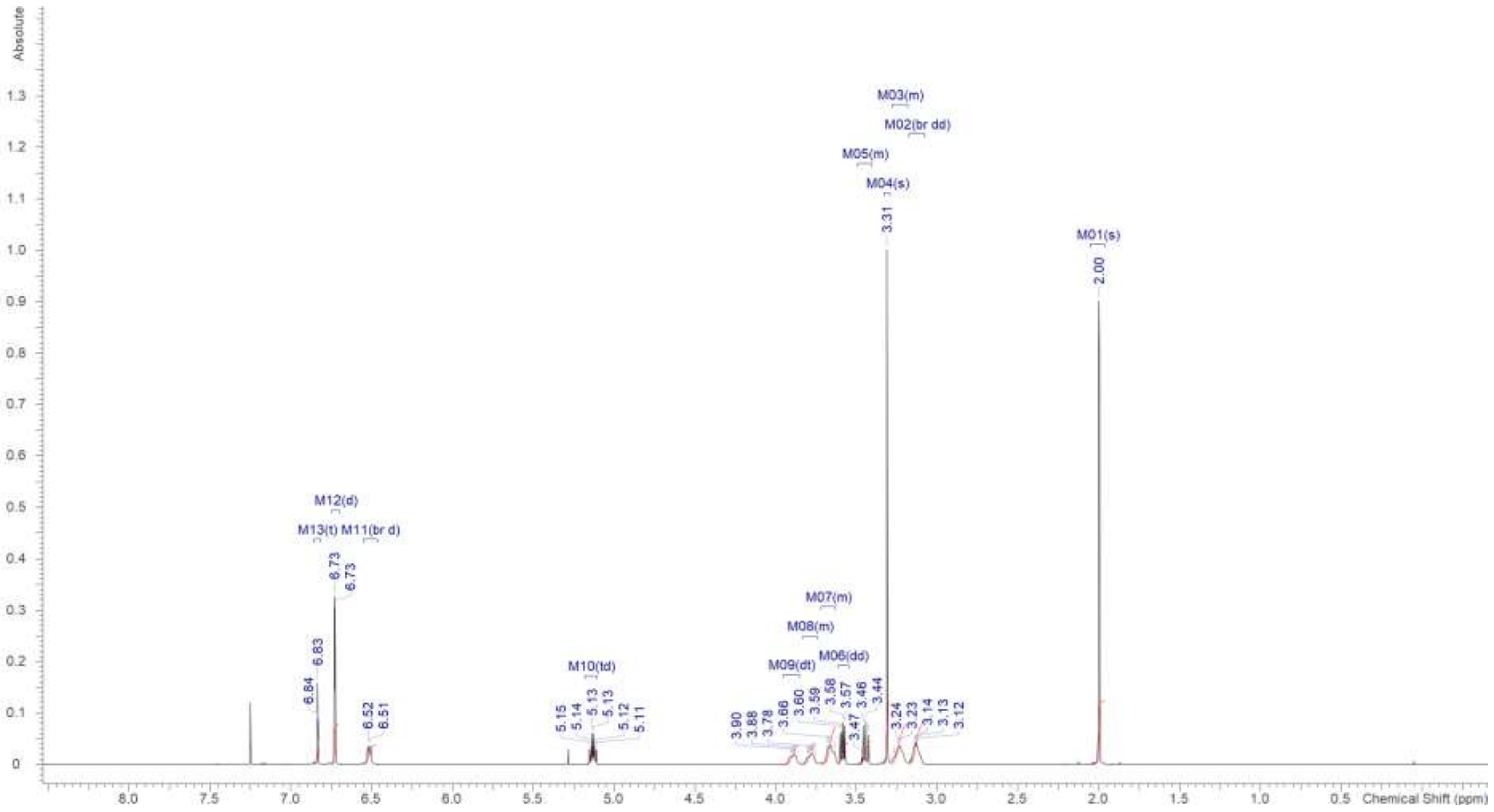

(*R*,*S*)-*N*-(1-(4-(3,5-dichlorophenyl)piperazin-1-yl)-3-methoxy-1-oxopropan-2-yl)acetamide (*R*,*S*)-41 – ^{13}C NMR

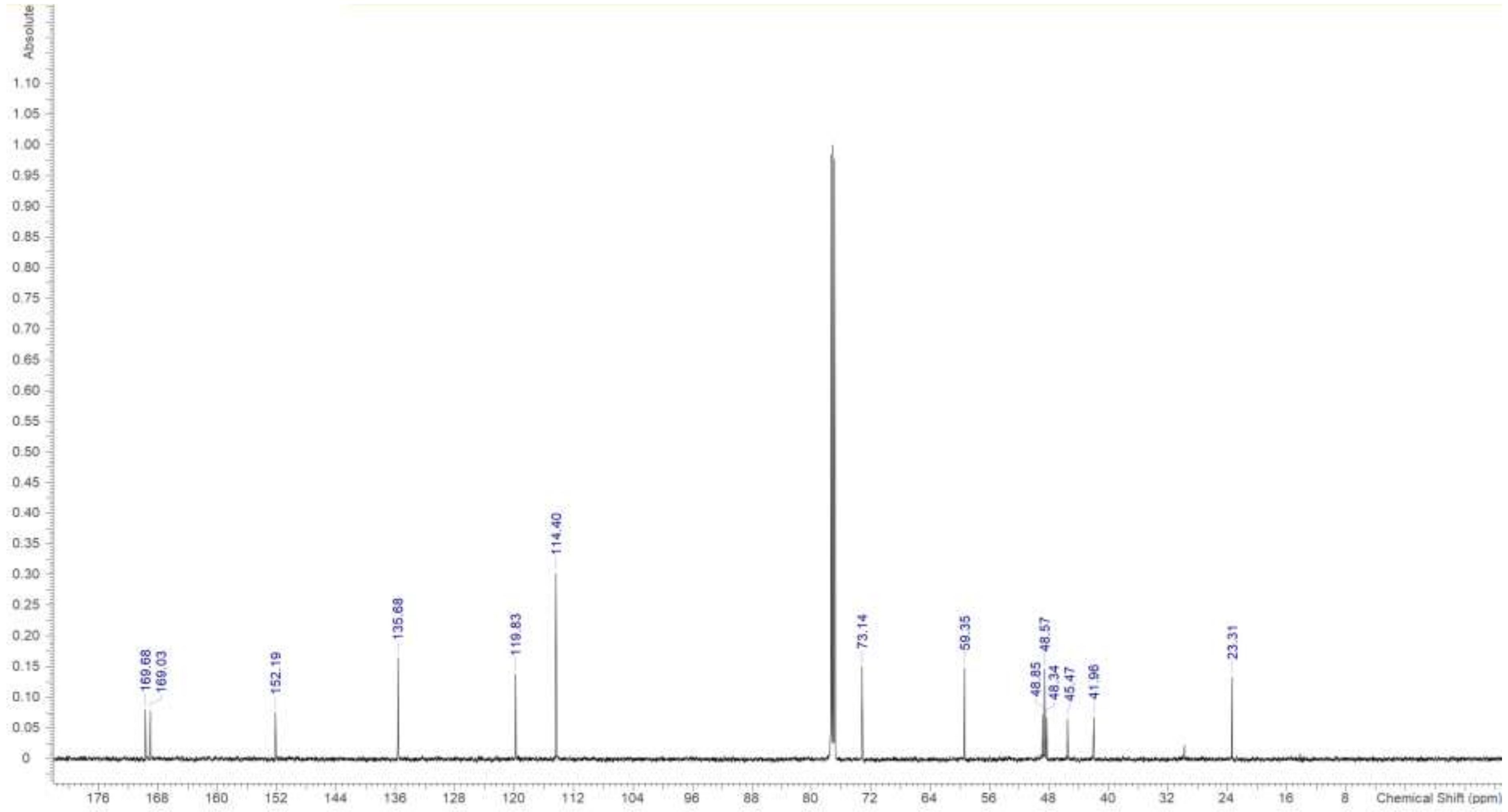

(*R,S*)-*N*-(3-methoxy-1-oxo-1-(4-(3-(trifluoromethyl)phenyl)piperazin-1-yl)propan-2-yl)acetamide (*R,S*)-42 – ^{1}H NMR

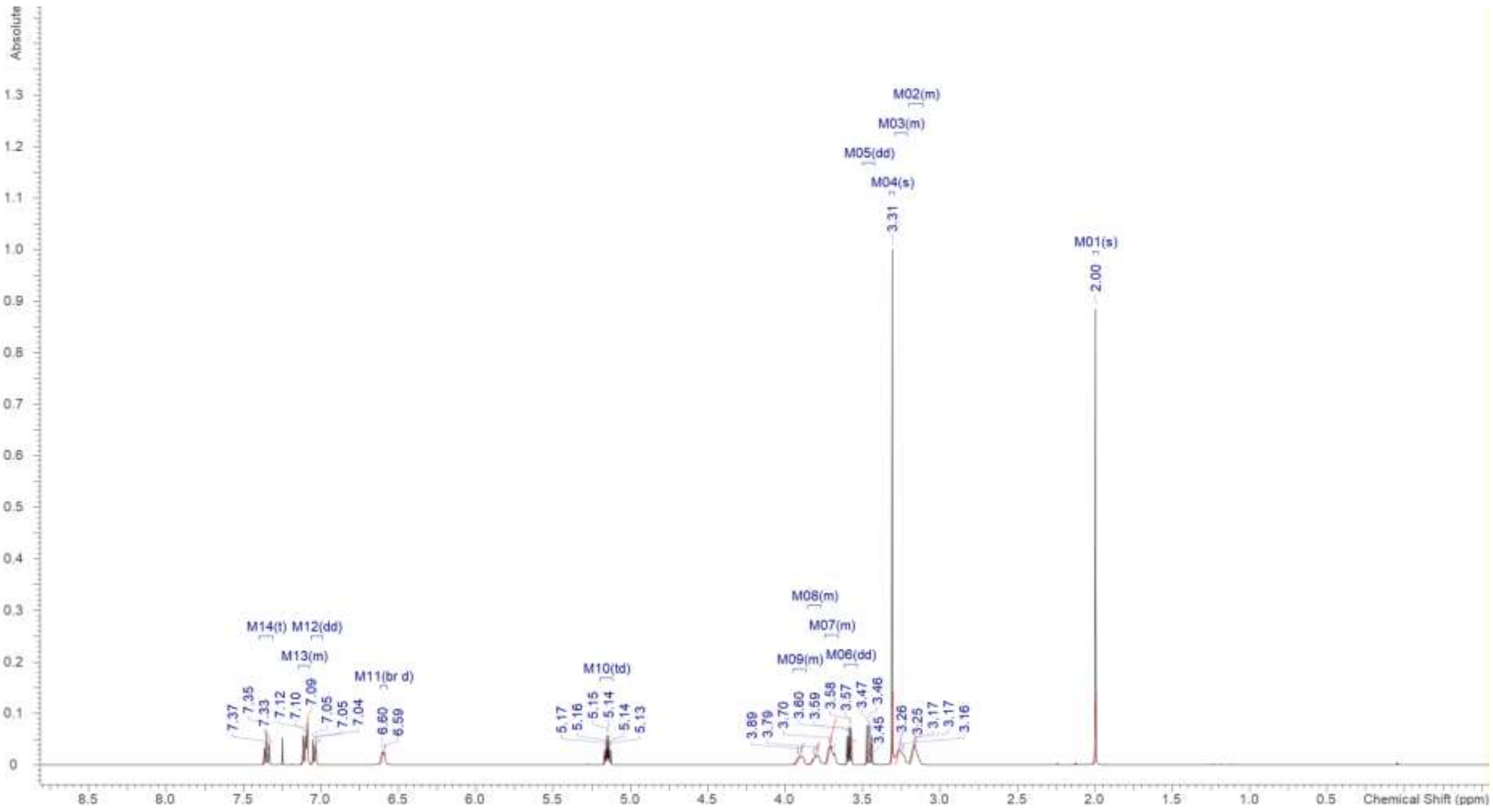

(*R,S*)-*N*-(3-methoxy-1-oxo-1-(4-(3-(trifluoromethyl)phenyl)piperazin-1-yl)propan-2-yl)acetamide (*R,S*)-42 – ^{13}C NMR

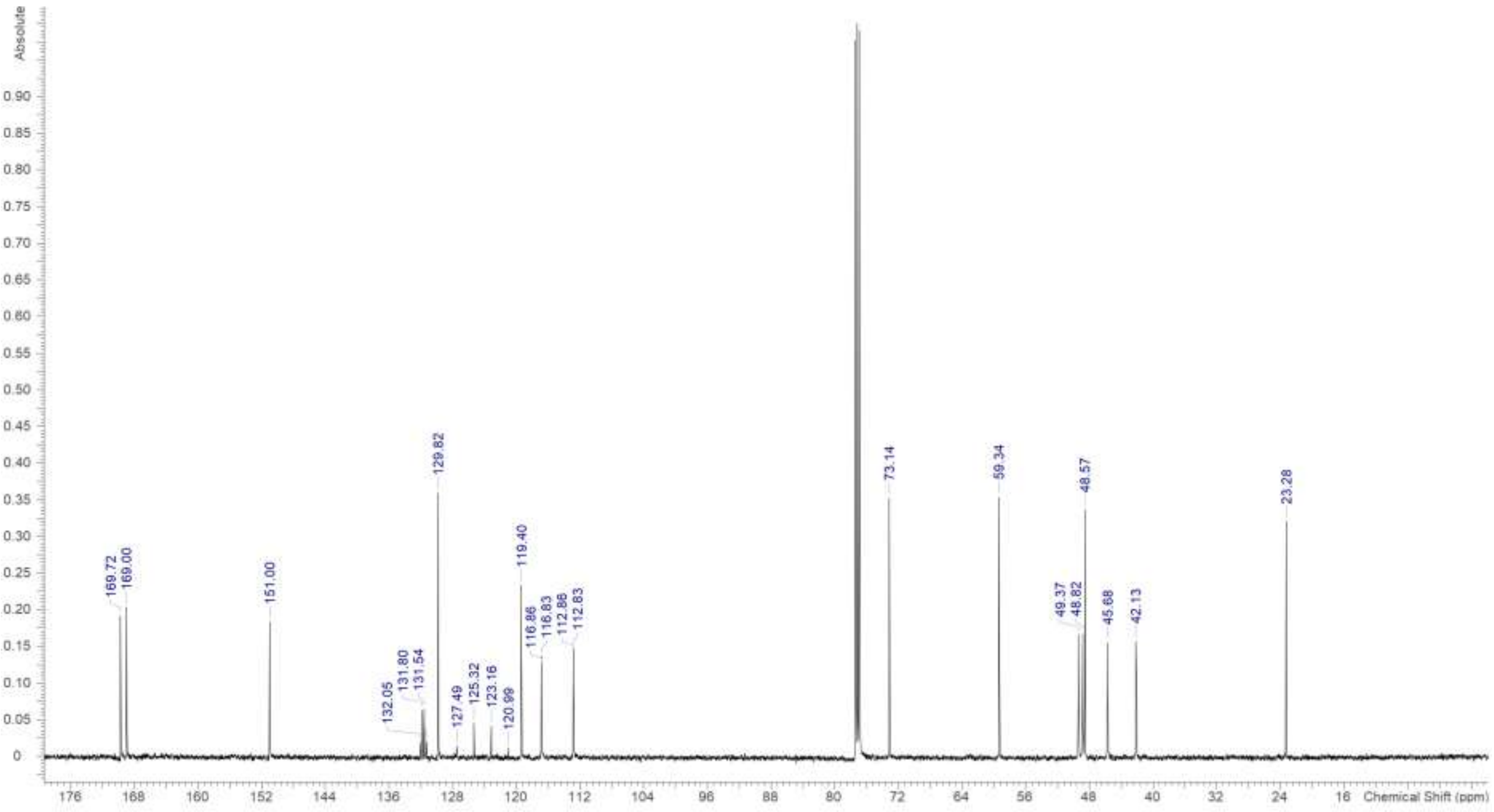

(*R,S*)-*N*-(3-methoxy-1-oxo-1-(4-(4-(trifluoromethyl)phenyl)piperazin-1-yl)propan-2-yl)acetamide (*R,S*)-43 – ^{1}H NMR

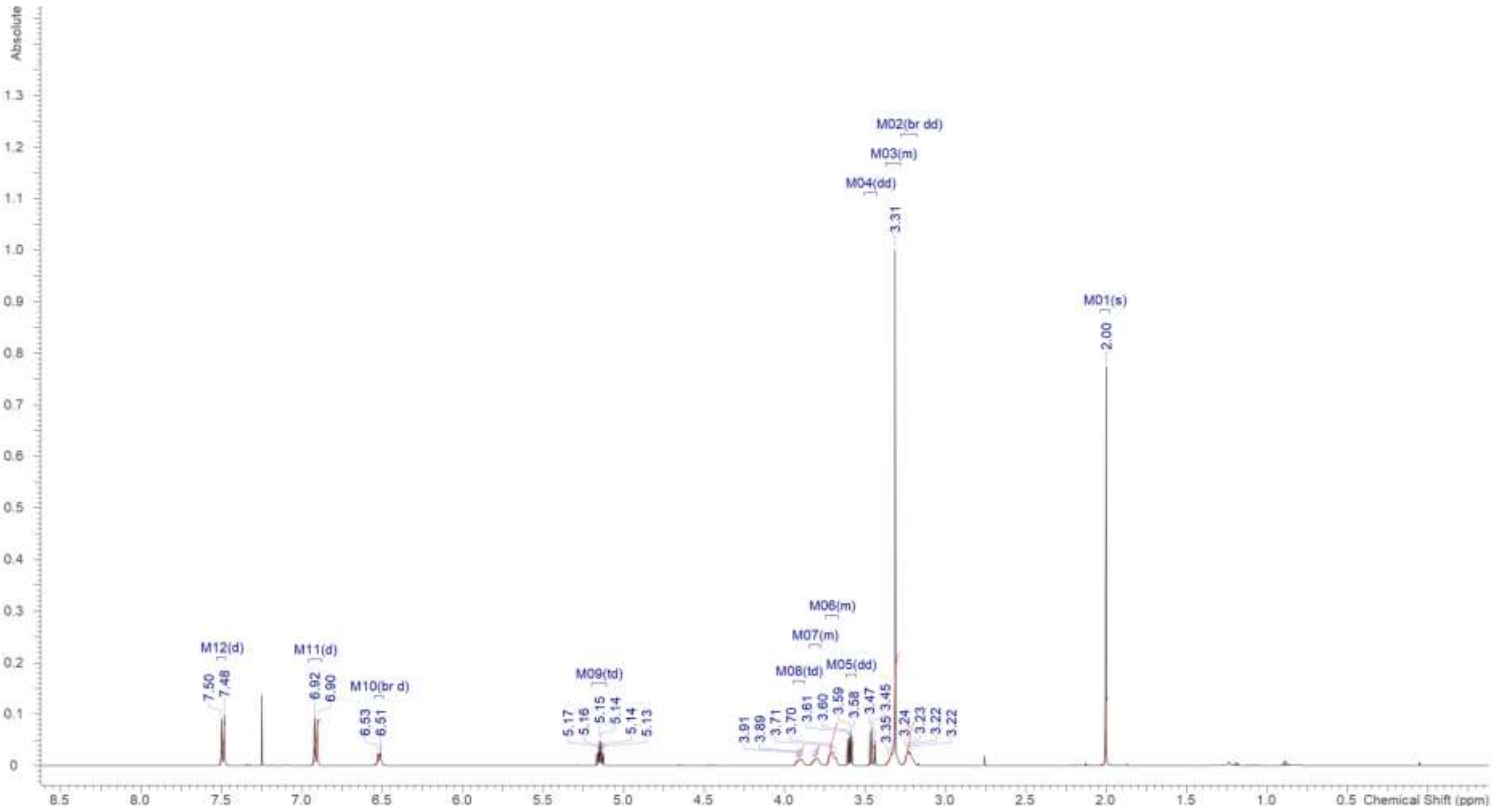

(*R,S*)-*N*-(3-methoxy-1-oxo-1-(4-(4-(trifluoromethyl)phenyl)piperazin-1-yl)propan-2-yl)acetamide (*R,S*)-43 – ^{13}C NMR

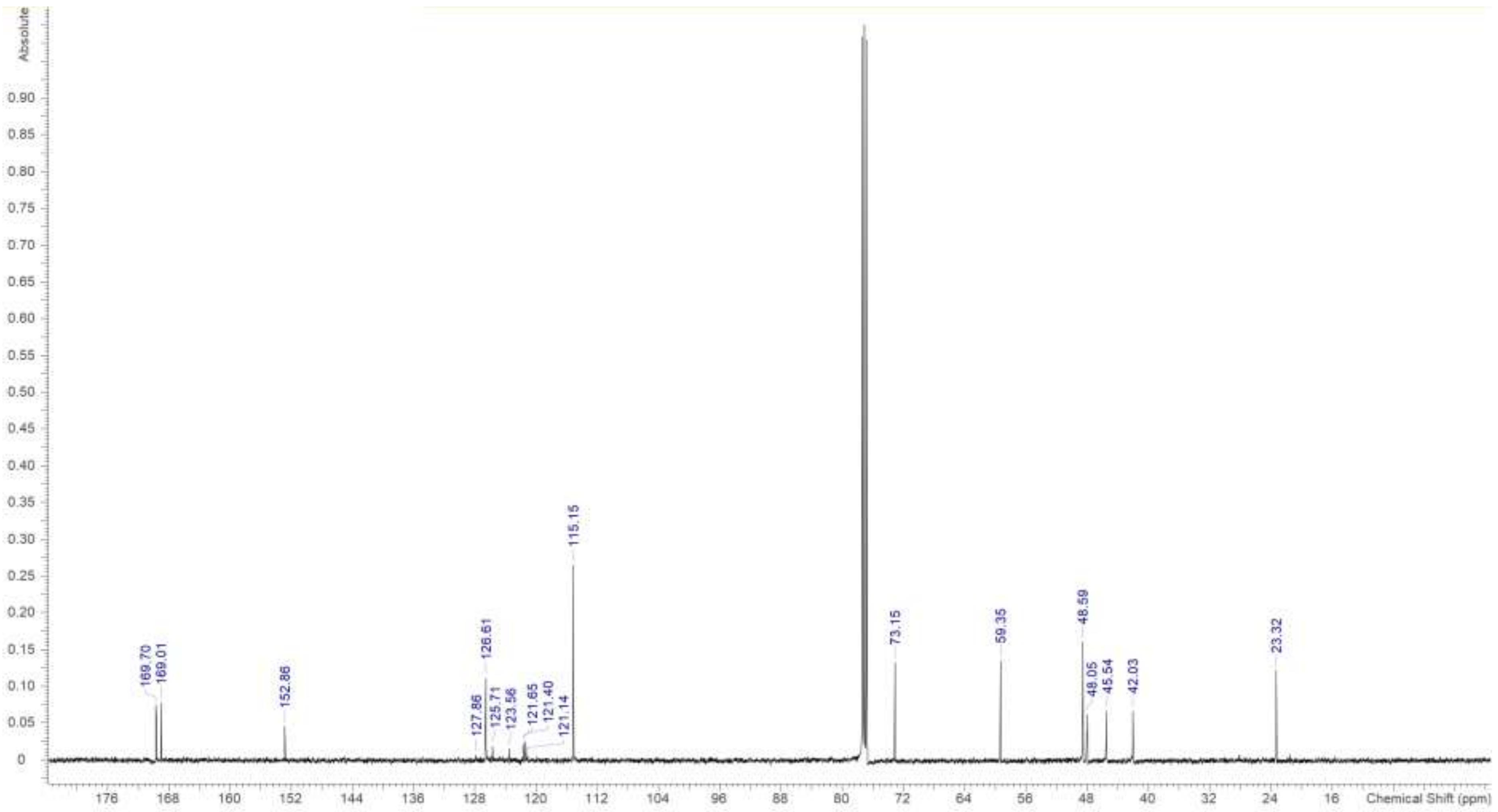

(*R*,*S*)-*N*-(1-(4-([1,1'-biphenyl]-3-yl)piperazin-1-yl)-3-methoxy-1-oxopropan-2-yl)acetamide (*R*,*S*)-44 – ^{1}H NMR

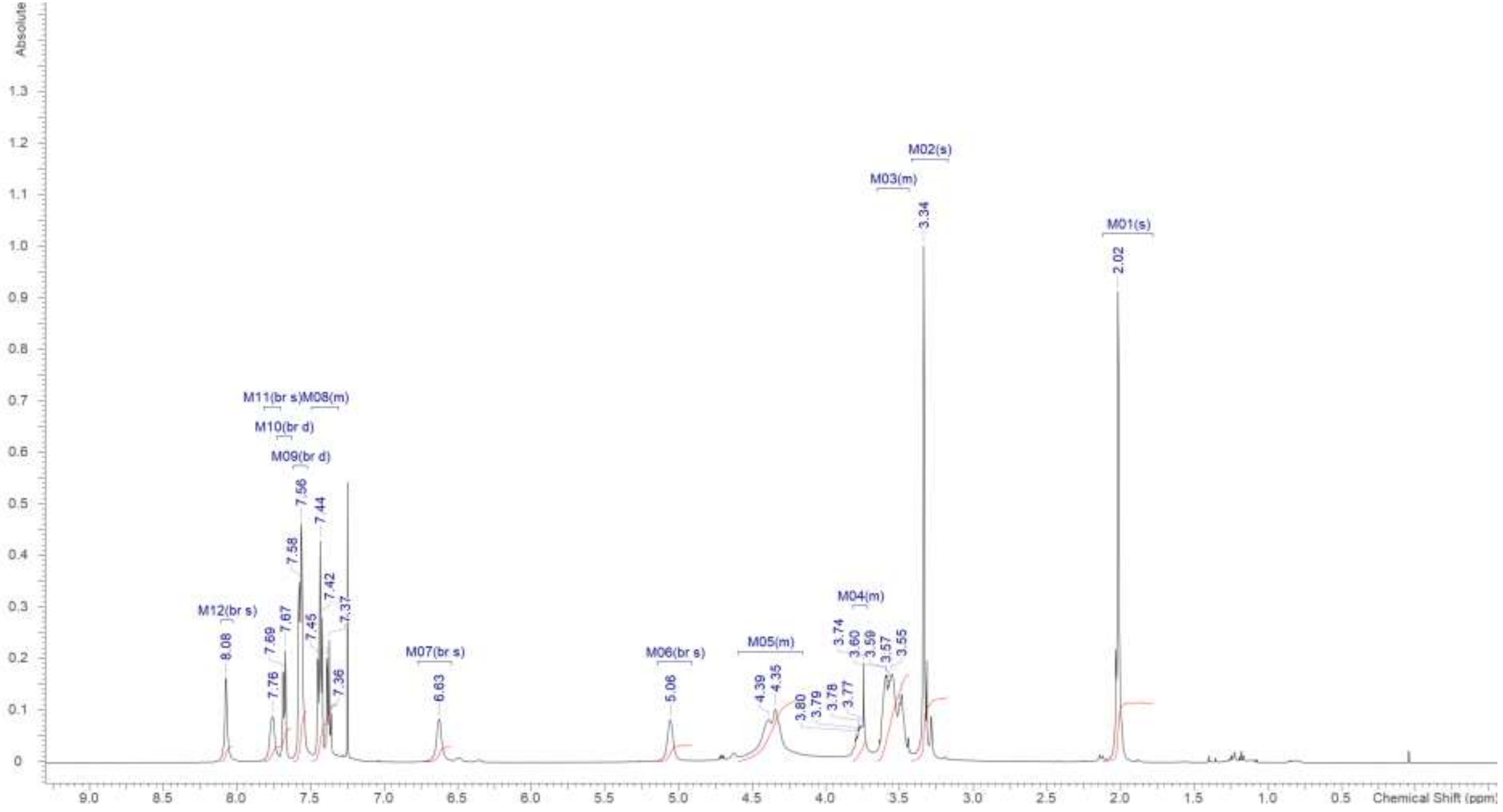

(*R*,*S*)-*N*-(1-(4-([1,1'-biphenyl]-3-yl)piperazin-1-yl)-3-methoxy-1-oxopropan-2-yl)acetamide (*R*,*S*)-44 – ^{13}C NMR

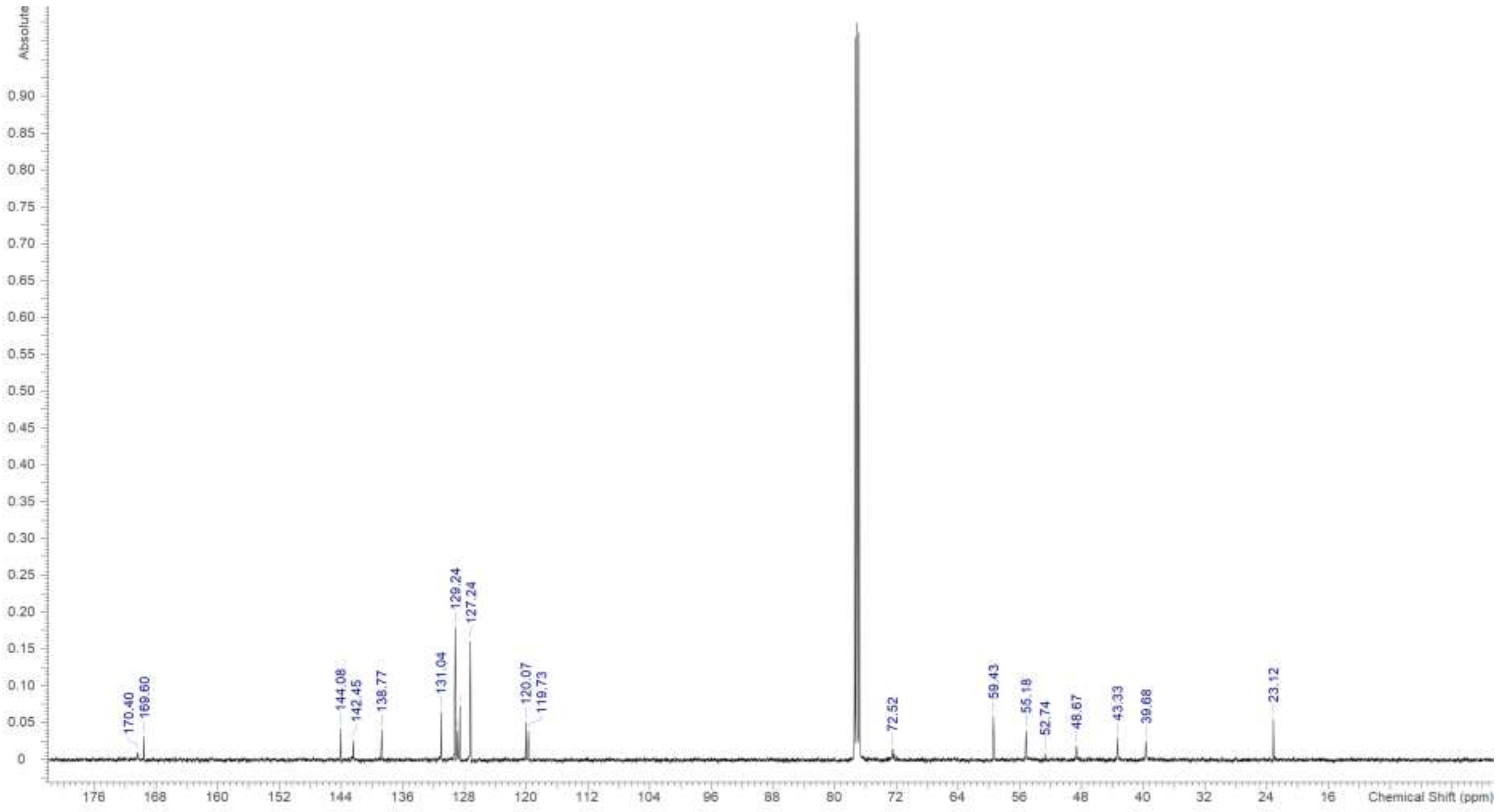

(*R*)-*N*-(1-(4-([1,1'-biphenyl]-3-yl)piperazin-1-yl)-3-methoxy-1-oxopropan-2-yl)acetamide (*R*)-44 – ^{1}H NMR

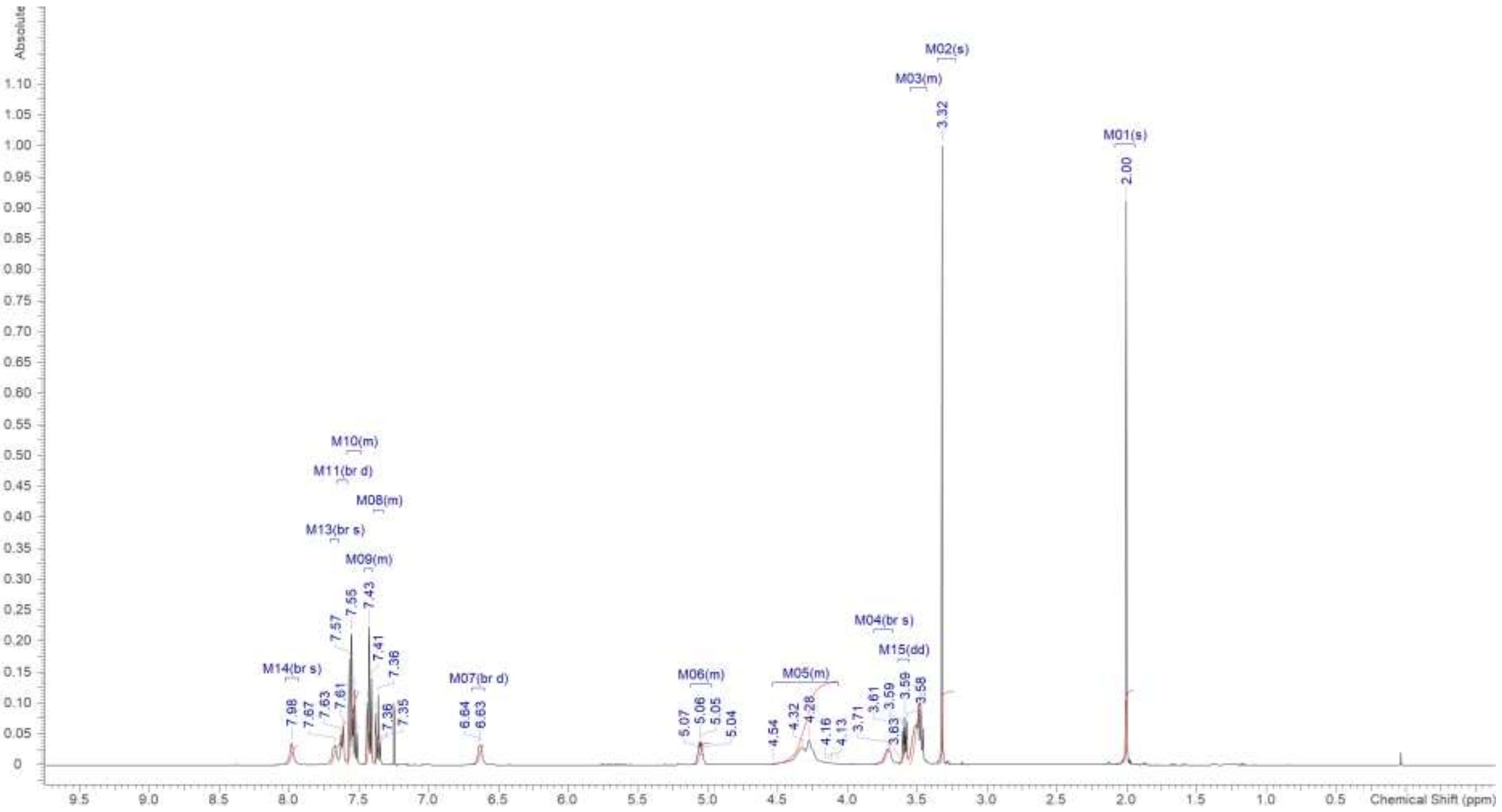

(*R*)-*N*-(1-(4-([1,1'-biphenyl]-3-yl)piperazin-1-yl)-3-methoxy-1-oxopropan-2-yl)acetamide (*R*)-44 – ^{13}C NMR

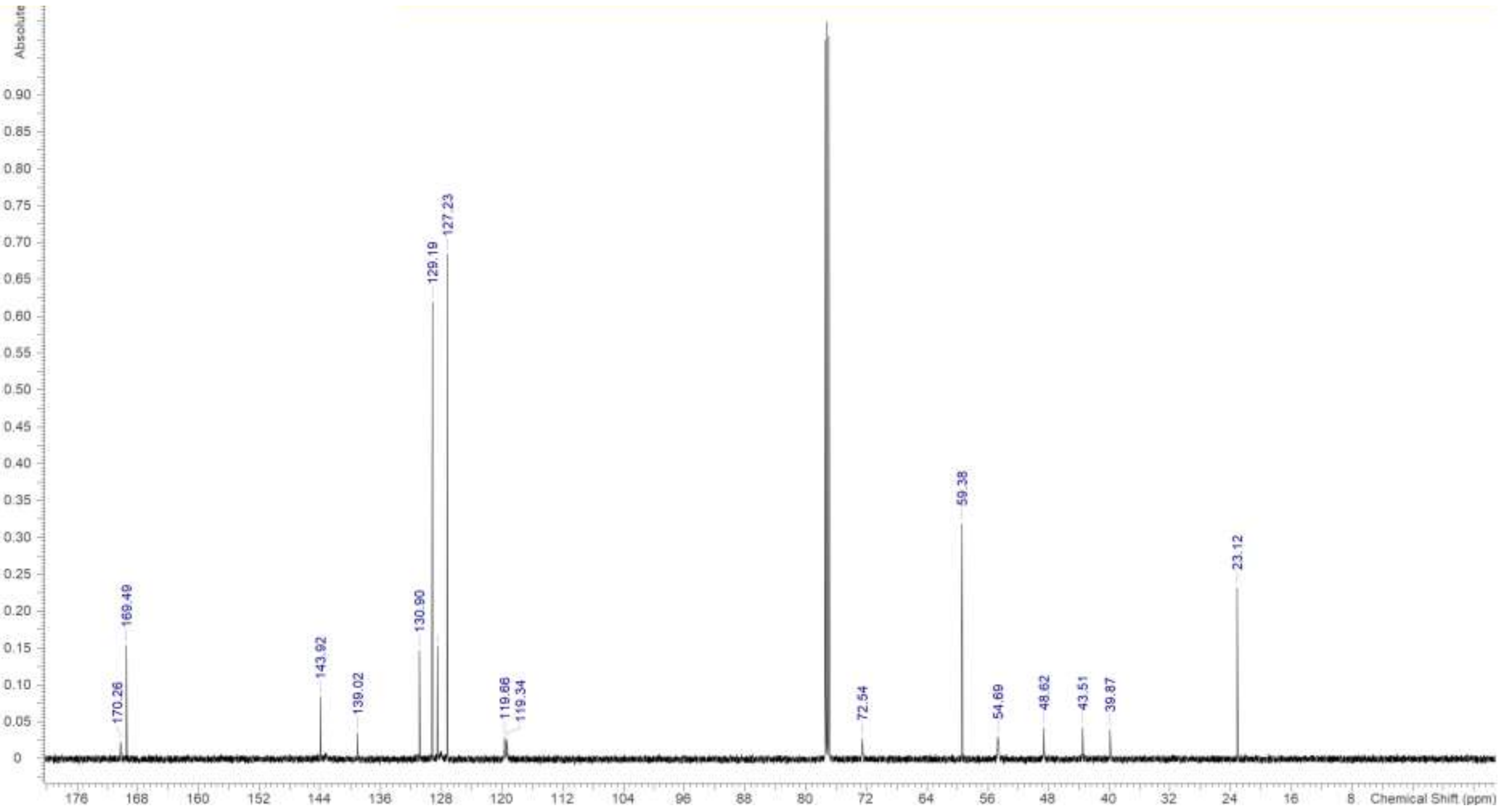

(*S*)-*N*-(1-(4-([1,1'-biphenyl]-3-yl)piperazin-1-yl)-3-methoxy-1-oxopropan-2-yl)acetamide (*S*)-44 – ^{1}H NMR

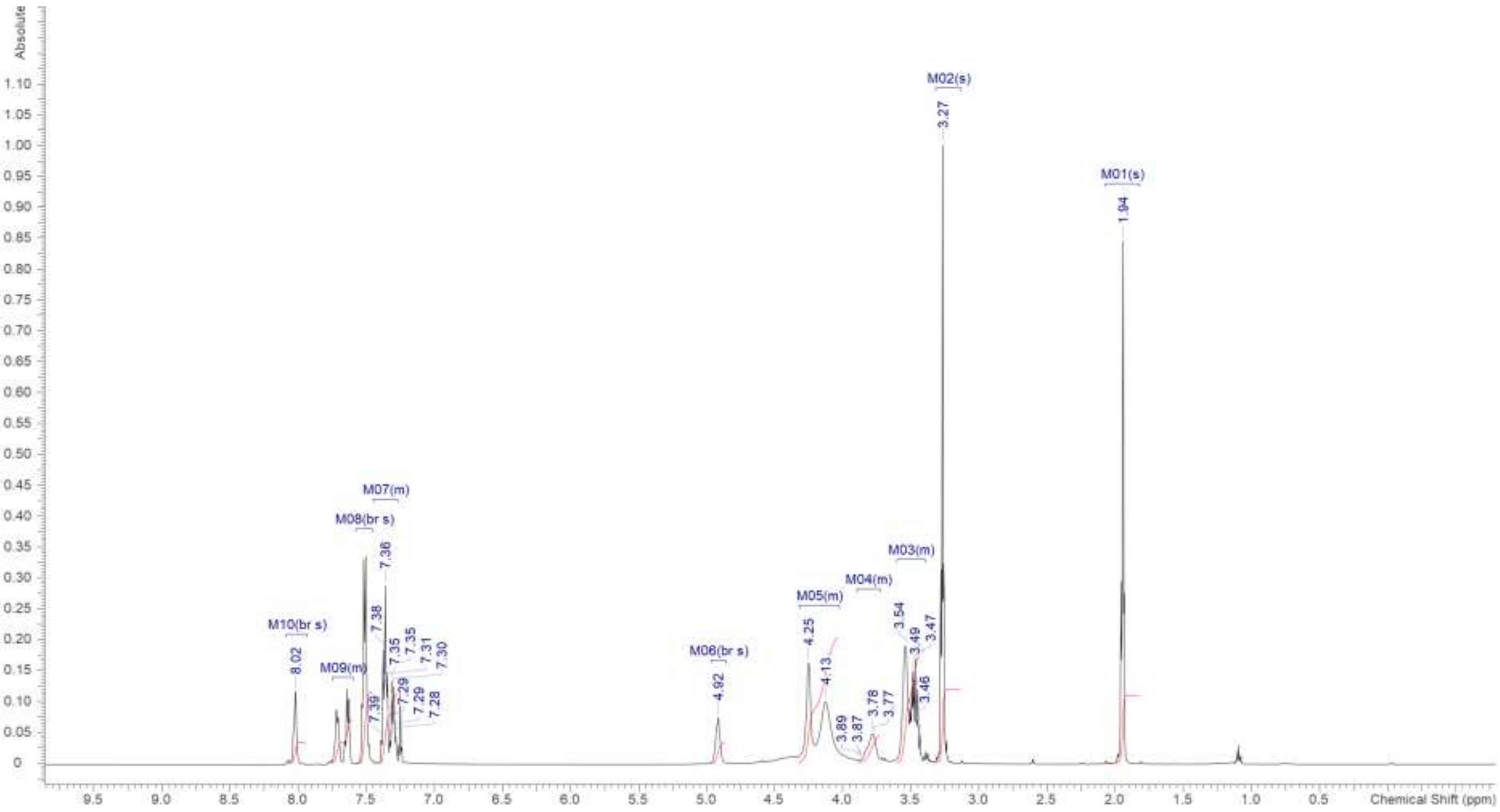

(*S*)-*N*-(1-(4-([1,1'-biphenyl]-3-yl)piperazin-1-yl)-3-methoxy-1-oxopropan-2-yl)acetamide (*S*)-44 – ^{13}C NMR

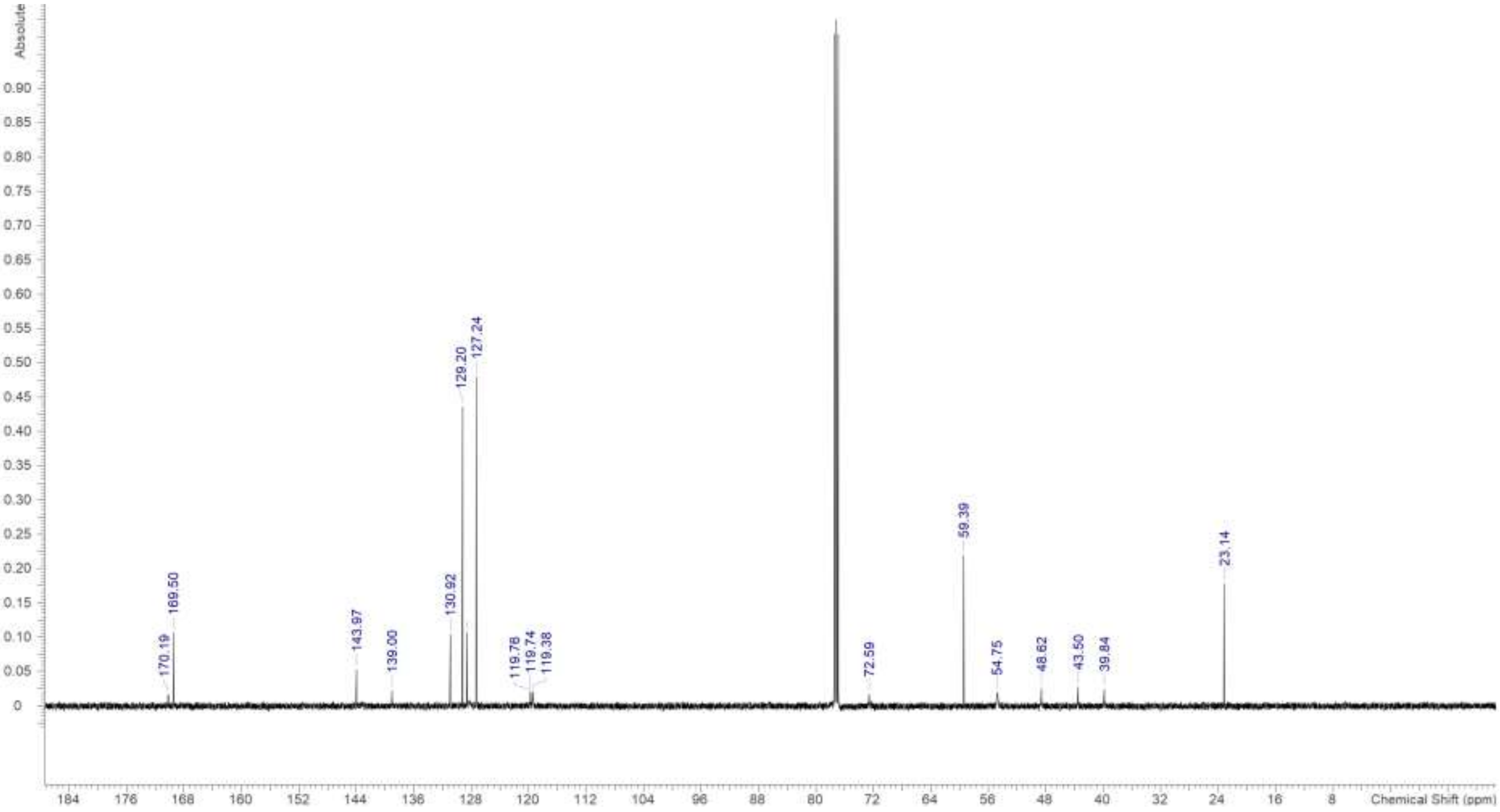

(*R*,*S*)-*N*-(1-(4-([1,1'-biphenyl]-4-yl)piperazin-1-yl)-3-methoxy-1-oxopropan-2-yl)acetamide (*R*,*S*)-45 – ^{1}H NMR

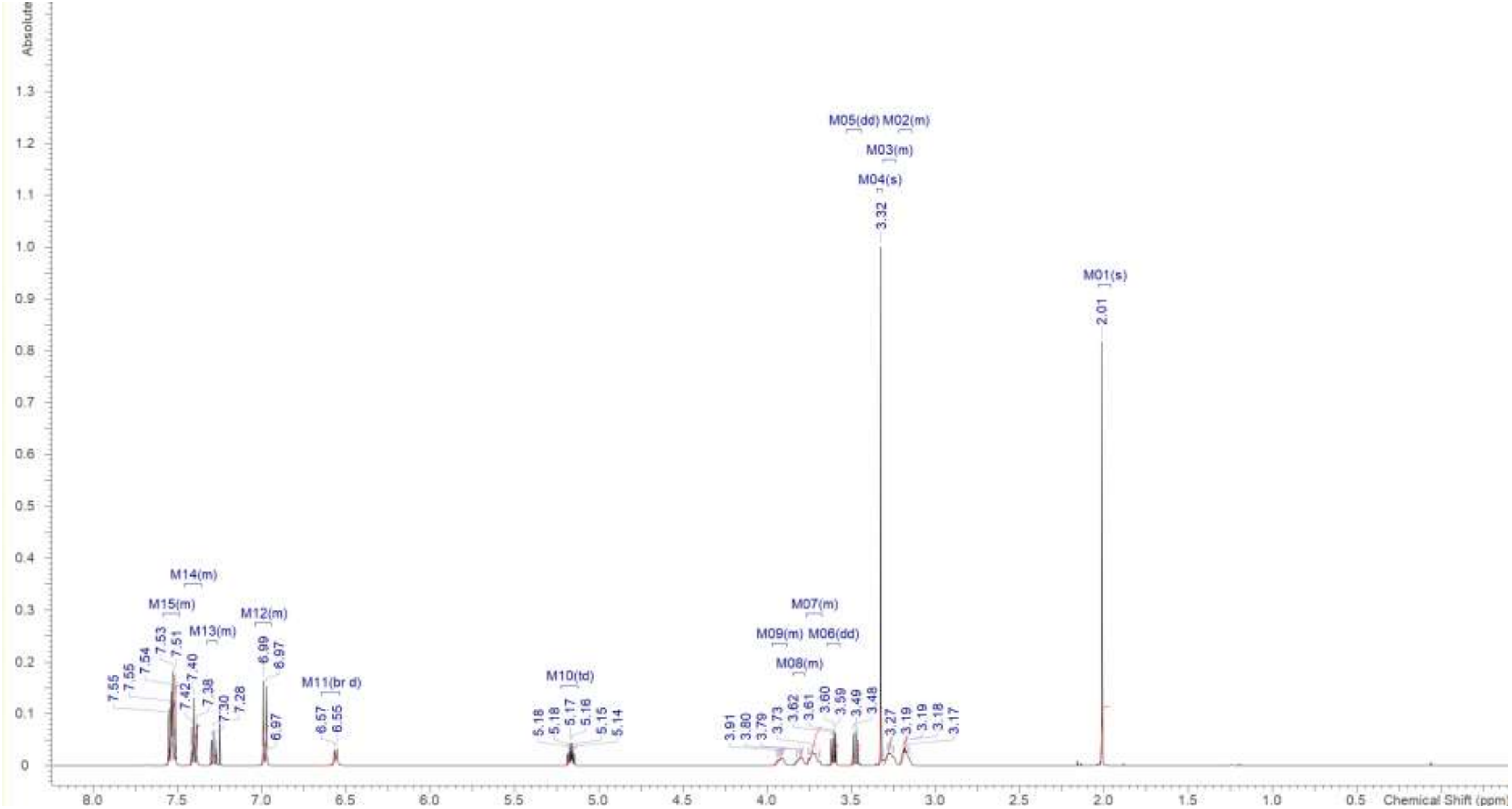

(*R*,*S*)-*N*-(1-(4-([1,1'-biphenyl]-4-yl)piperazin-1-yl)-3-methoxy-1-oxopropan-2-yl)acetamide (*R*,*S*)-45 – ^{13}C NMR

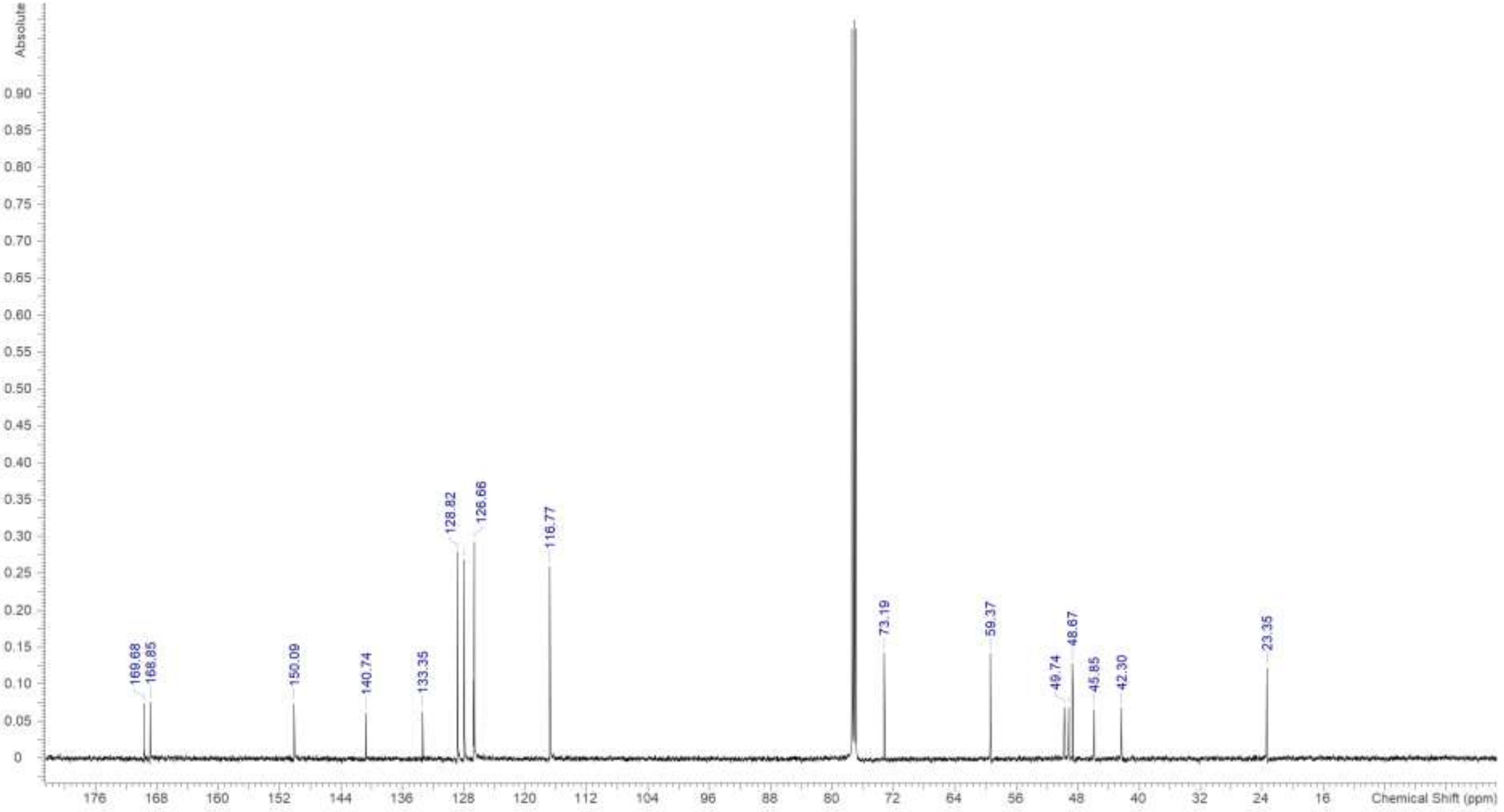

(*R*,*S*)-*N*-(3-methoxy-1-oxo-1-(4-(3-(trifluoromethoxy)phenyl)piperazin-1-yl)propan-2-yl)acetamide (*R*,*S*)-46 – ^{1}H NMR

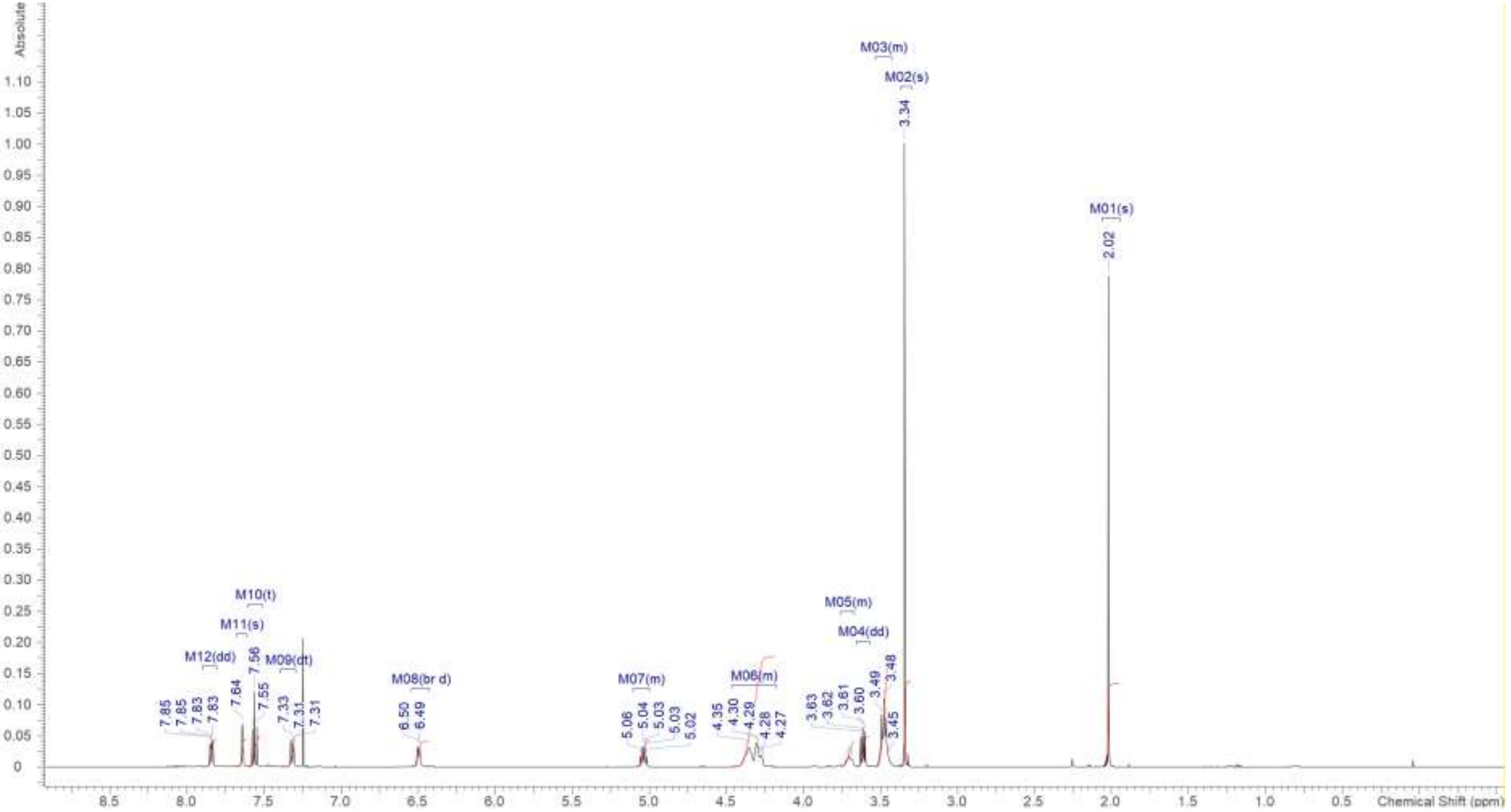

(*R*,*S*)-*N*-(3-methoxy-1-oxo-1-(4-(3-(trifluoromethoxy)phenyl)piperazin-1-yl)propan-2-yl)acetamide (*R*,*S*)-46 – ^{13}C NMR

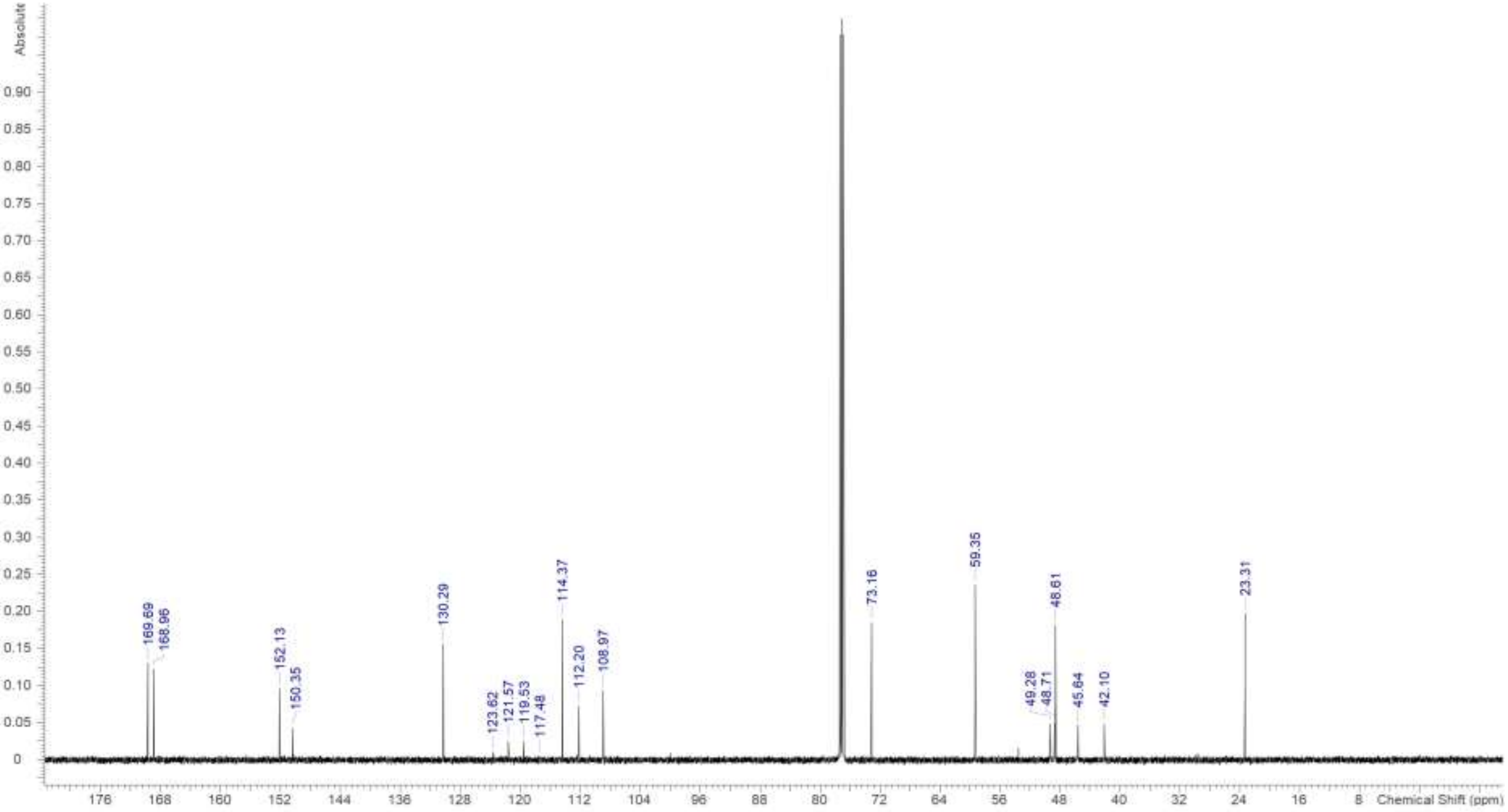

(*R*)-*N*-(3-methoxy-1-oxo-1-(4-(3-(trifluoromethoxy)phenyl)piperazin-1-yl)propan-2-yl)acetamide (*R*)-46 – ^{1}H NMR

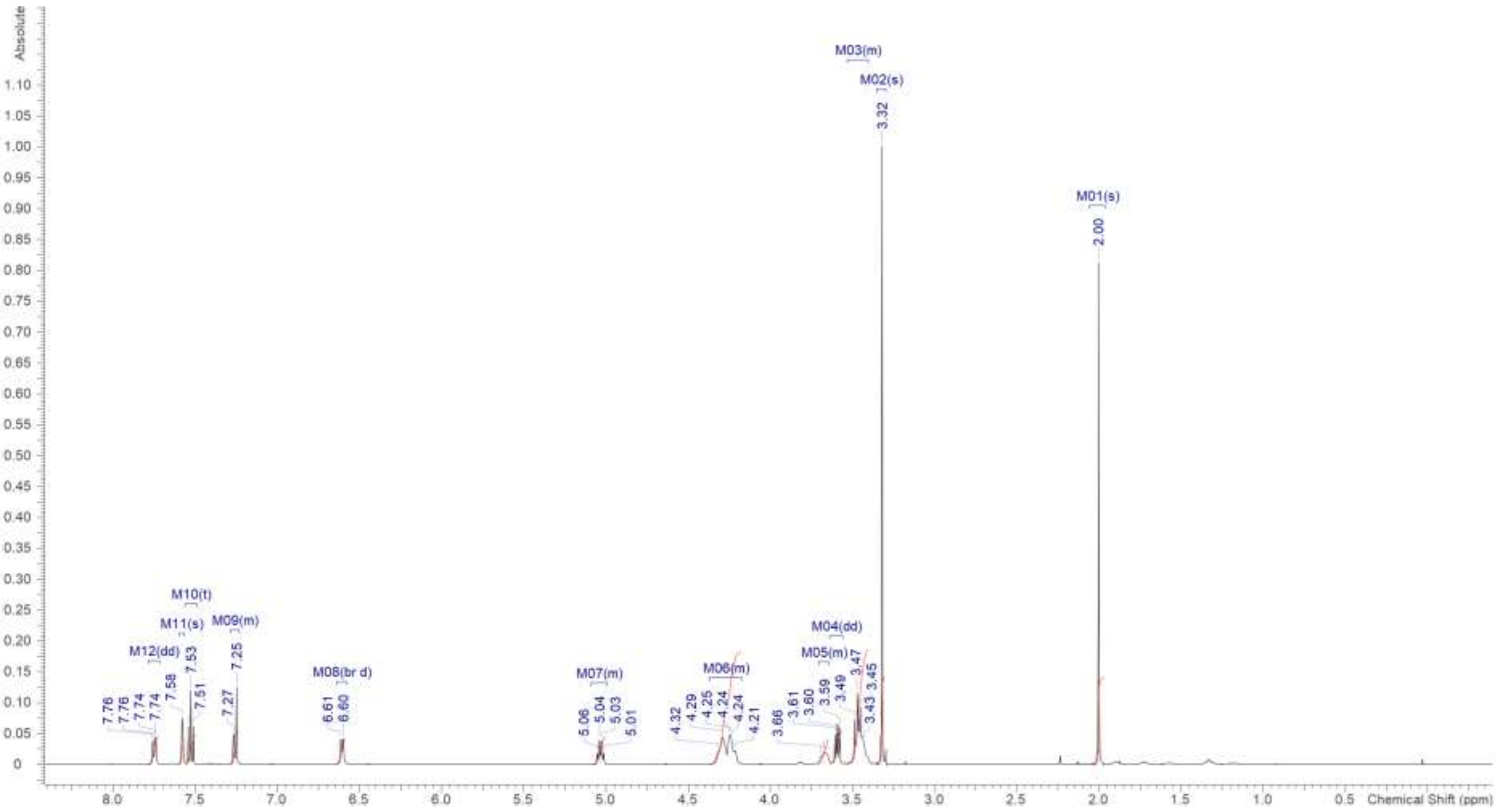

(*R*)-*N*-(3-methoxy-1-oxo-1-(4-(3-(trifluoromethoxy)phenyl)piperazin-1-yl)propan-2-yl)acetamide (*R*)-46 – ^{13}C NMR

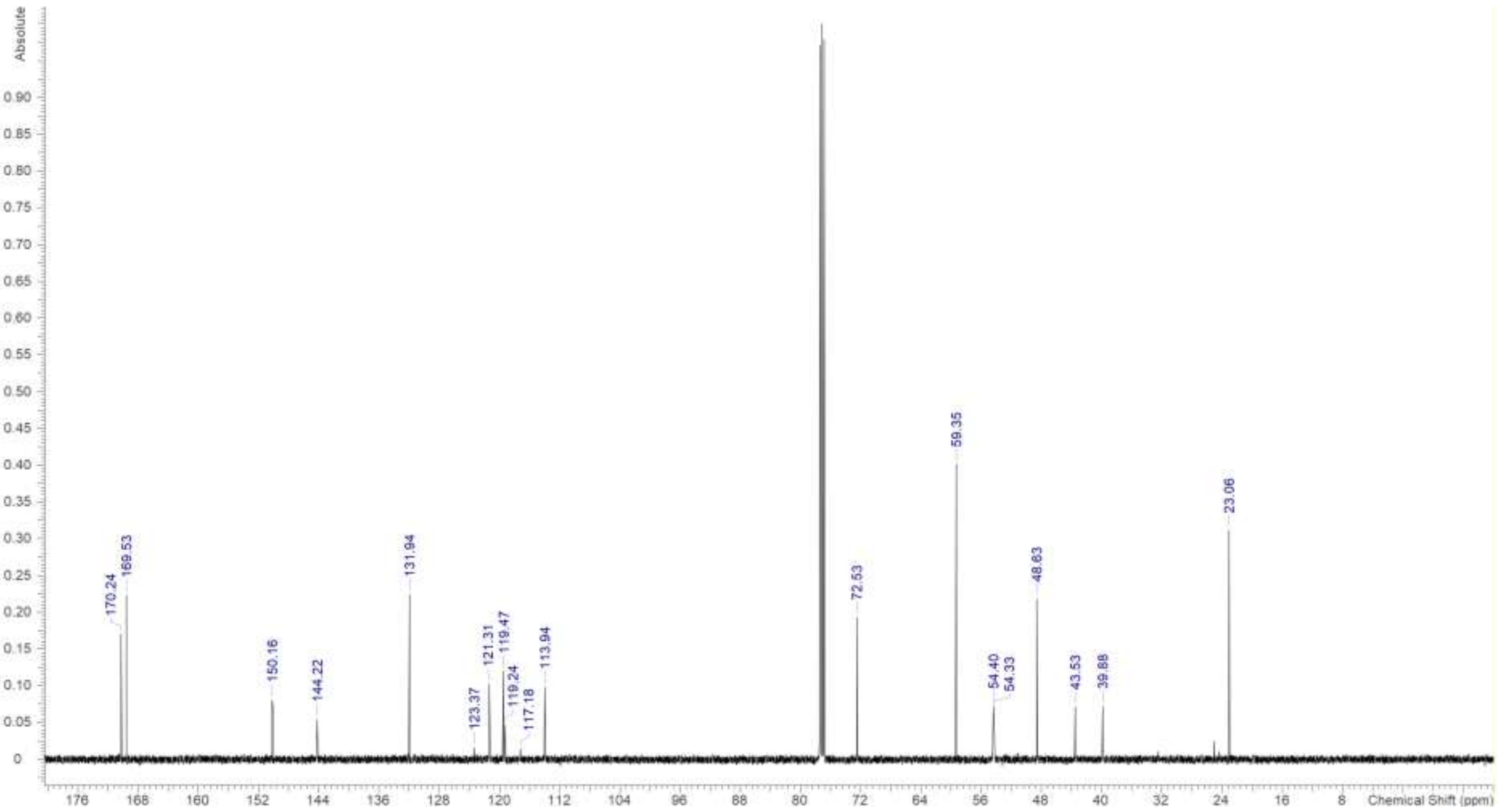

(*S*)-*N*-(3-methoxy-1-oxo-1-(4-(3-(trifluoromethoxy)phenyl)piperazin-1-yl)propan-2-yl)acetamide (*S*)-46 – ^{1}H NMR

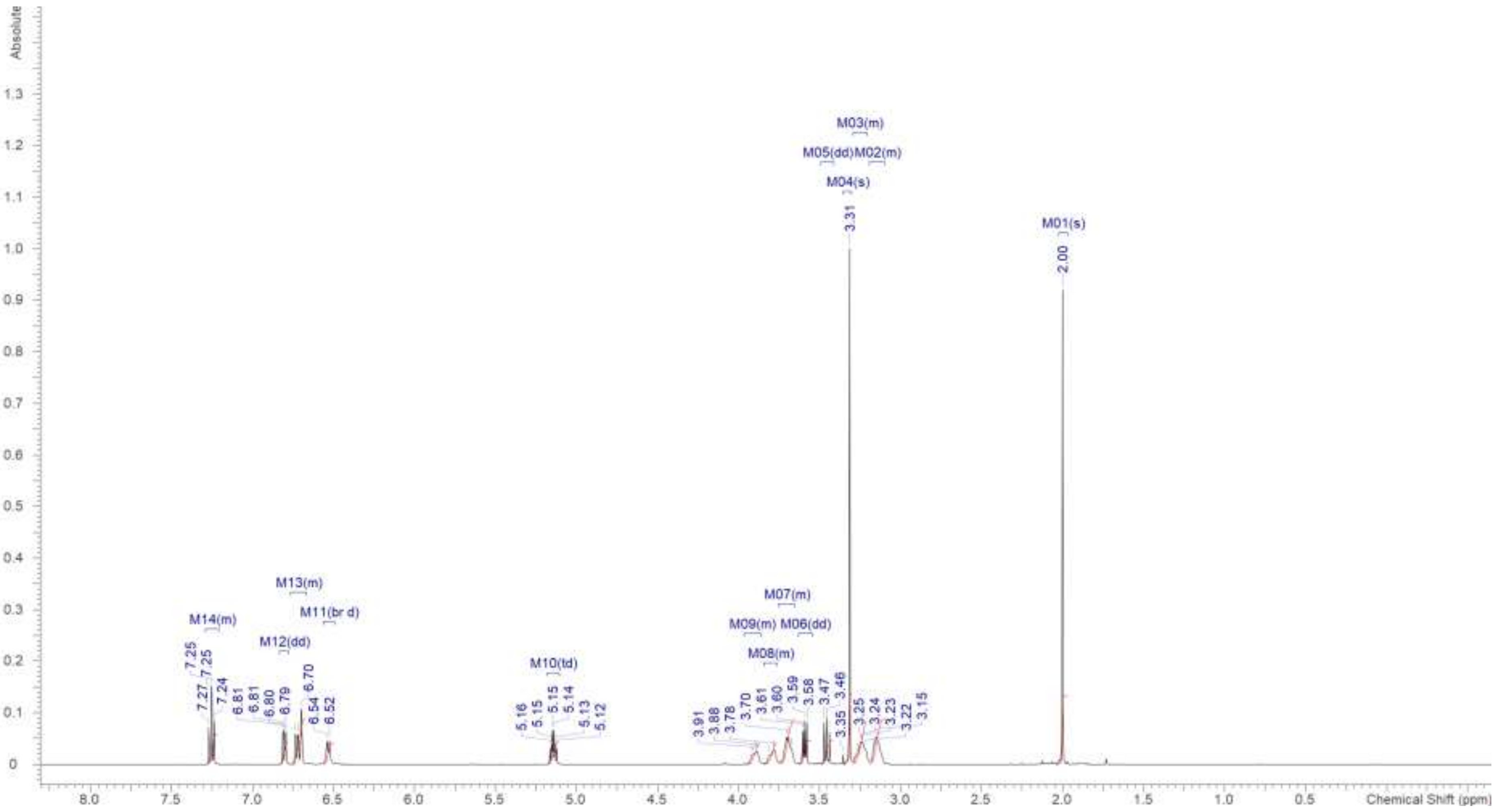

(*S*)-*N*-(3-methoxy-1-oxo-1-(4-(3-(trifluoromethoxy)phenyl)piperazin-1-yl)propan-2-yl)acetamide (*S*)-46 – ^{13}C NMR

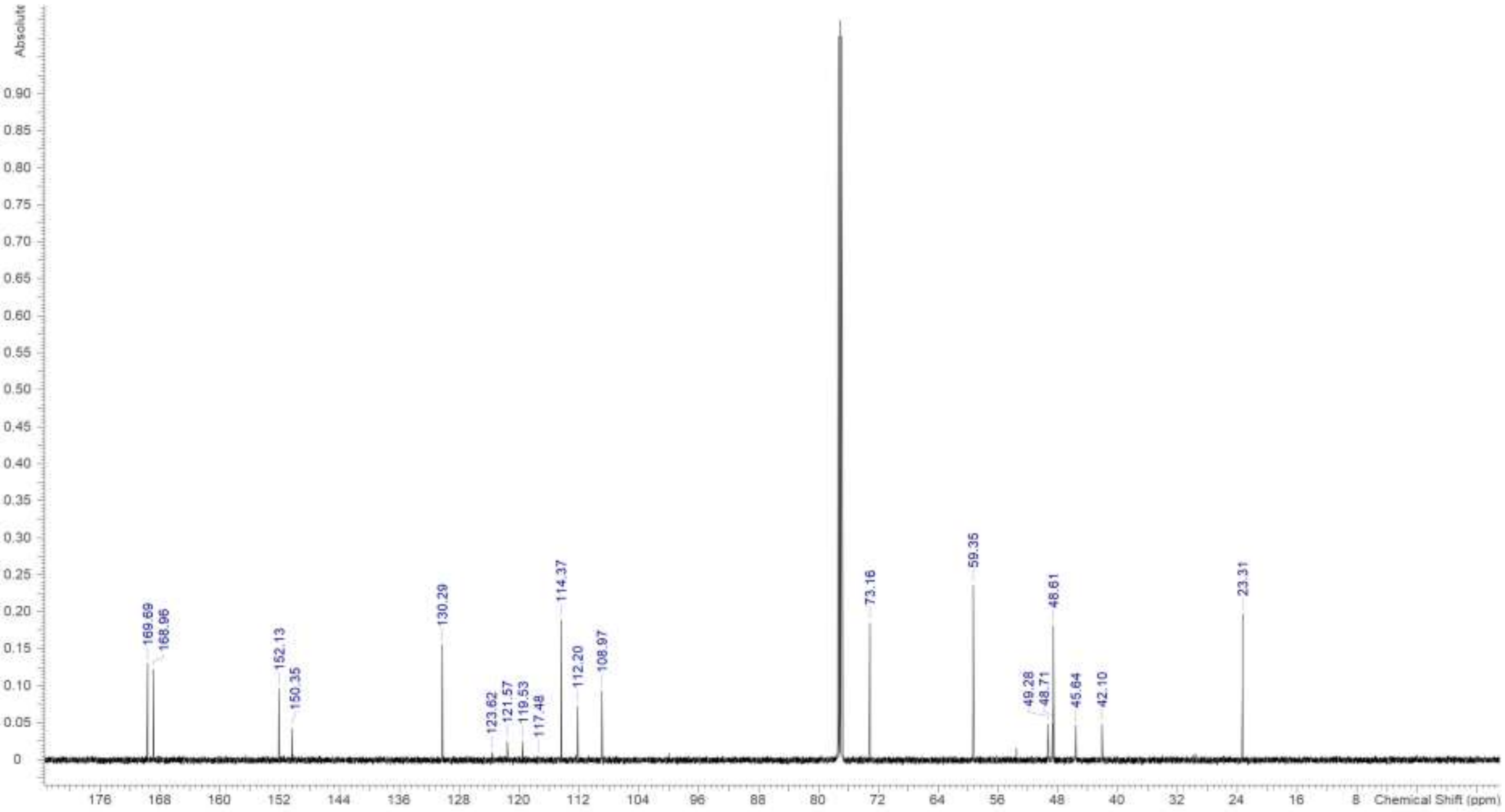

(*R*,*S*)-*N*-(3-methoxy-1-oxo-1-(4-(4-(trifluoromethoxy)phenyl)piperazin-1-yl)propan-2-yl)acetamide (*R*,*S*)-47 – ^{1}H NMR

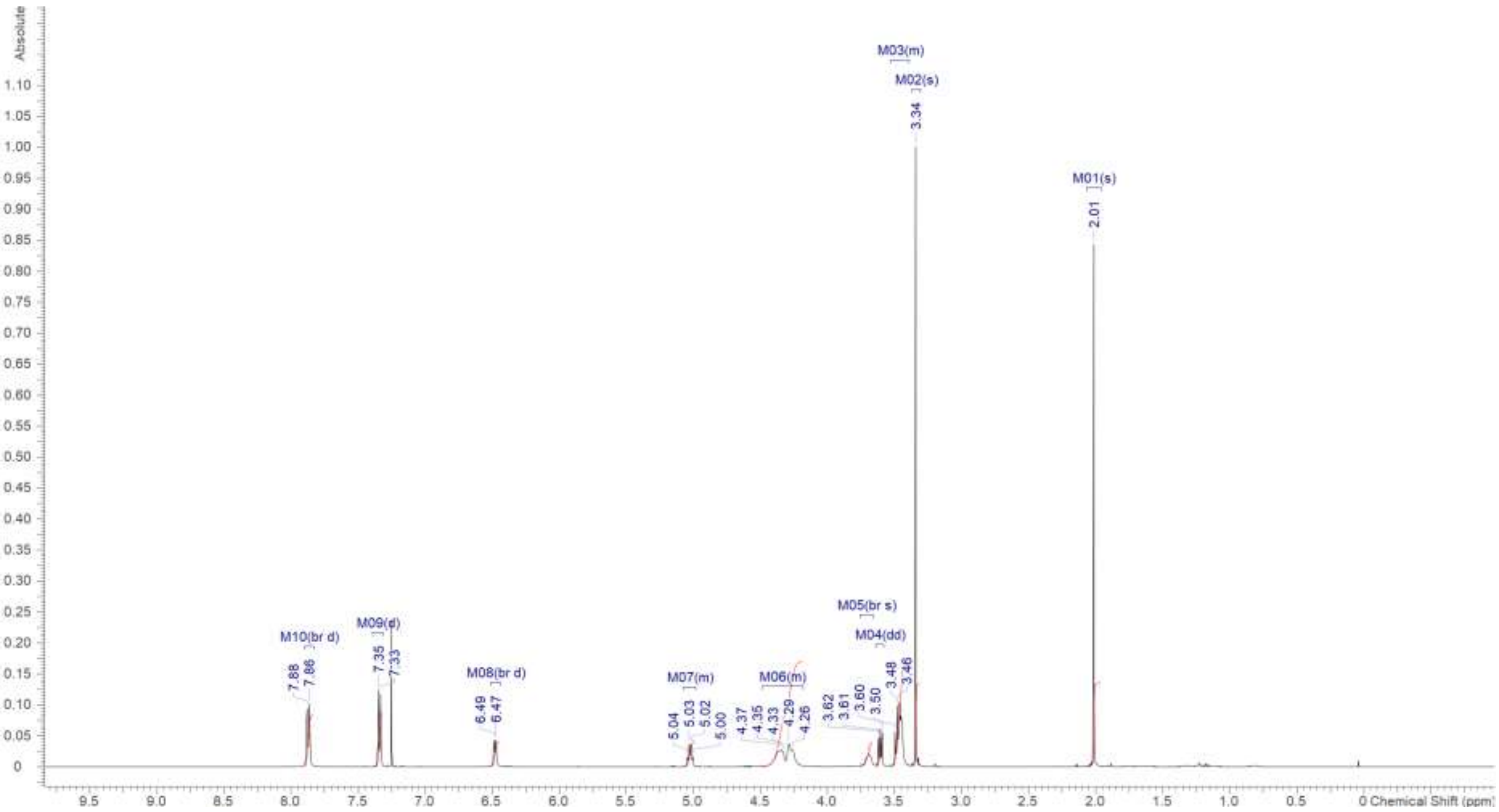

(*R*,*S*)-*N*-(3-methoxy-1-oxo-1-(4-(4-(trifluoromethoxy)phenyl)piperazin-1-yl)propan-2-yl)acetamide (*R*,*S*)-47 – ^{13}C NMR

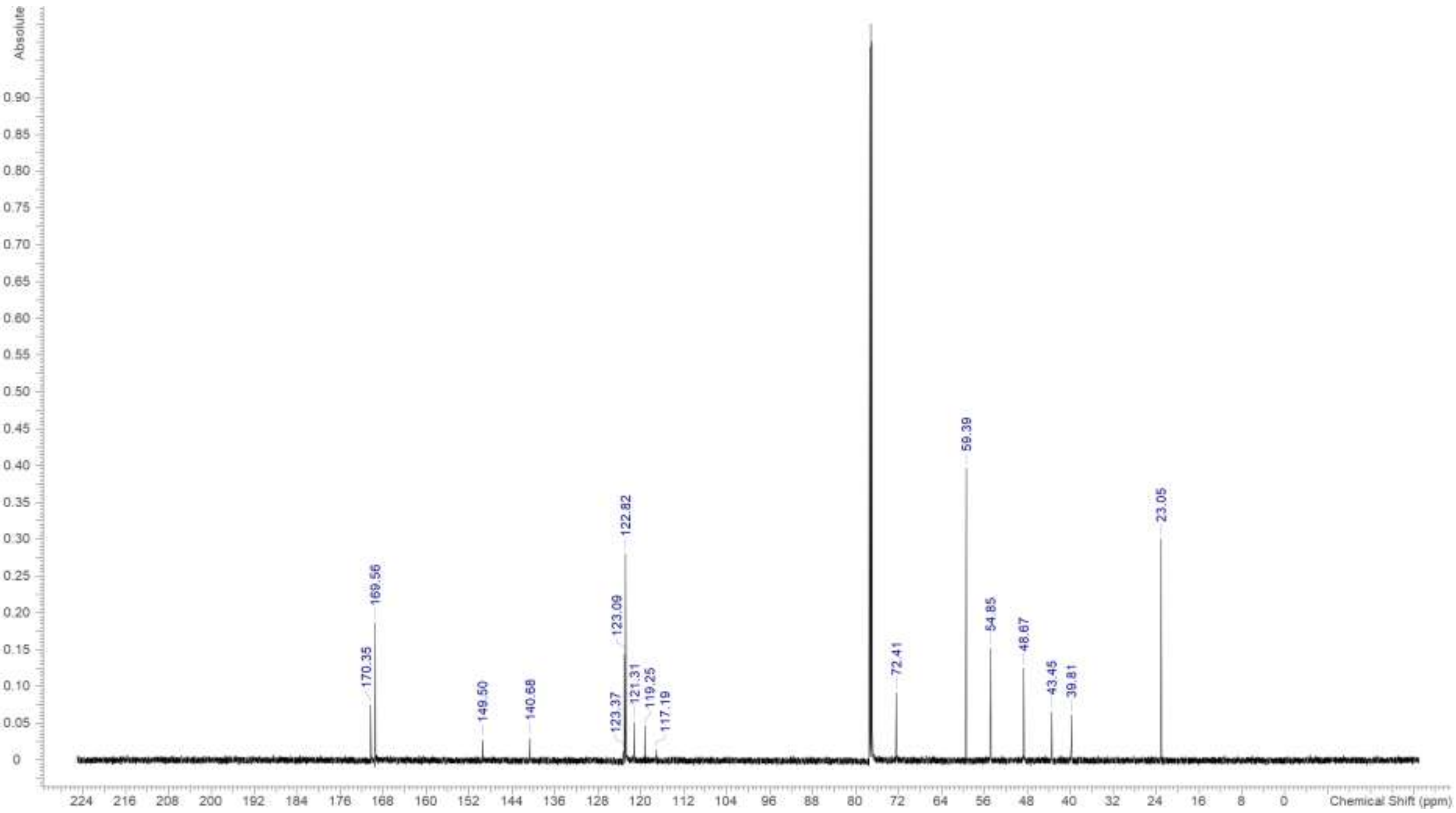

(*R*,*S*)-*N*-(3-methoxy-1-oxo-1-(4-(3-phenoxyphenyl)piperazin-1-yl)propan-2-yl)acetamide (*R*,*S*)-48 – ^{1}H NMR

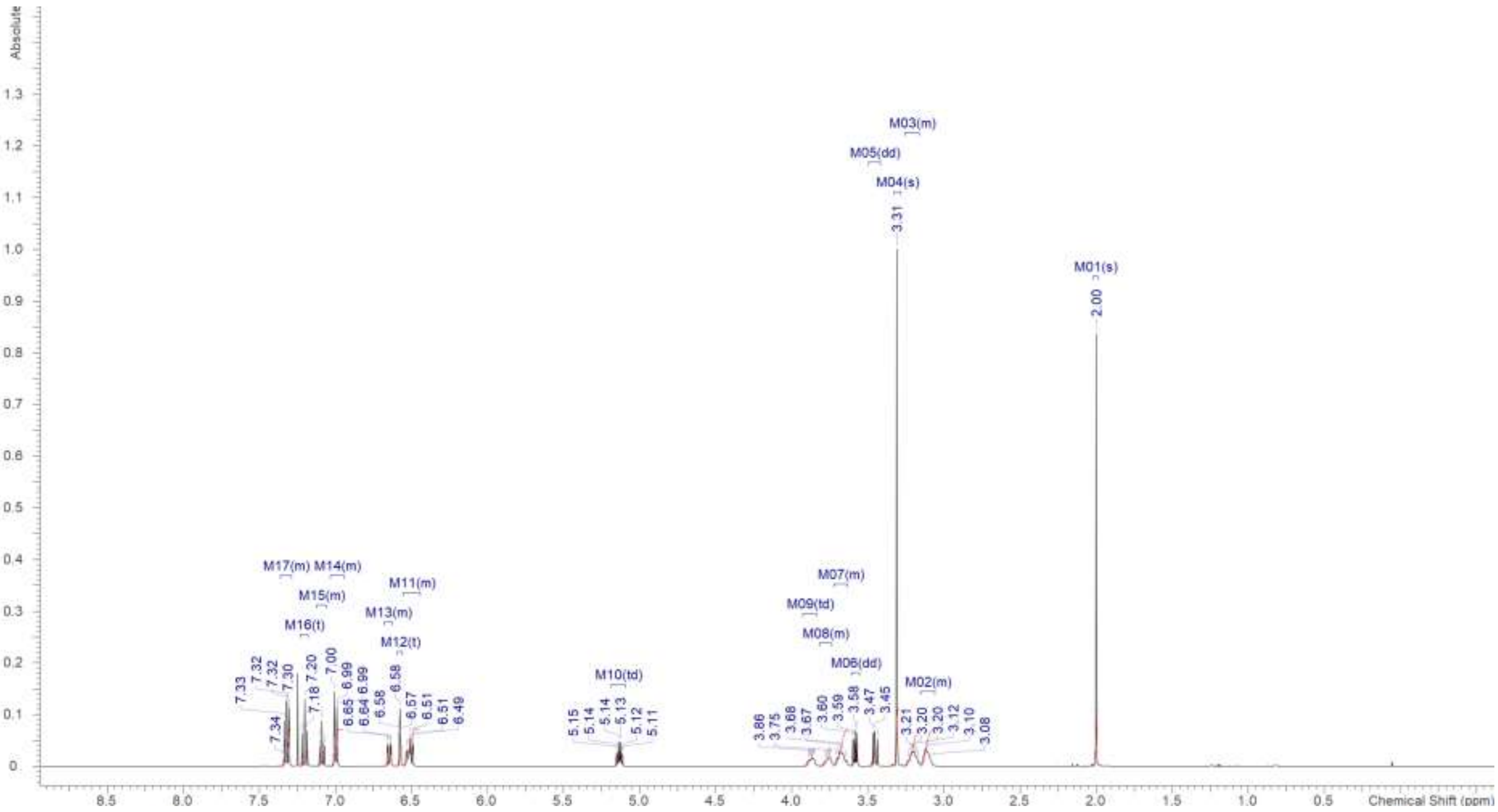

(*R*,*S*)-*N*-(3-methoxy-1-oxo-1-(4-(3-phenoxyphenyl)piperazin-1-yl)propan-2-yl)acetamide (*R*,*S*)-48 – ^{13}C NMR

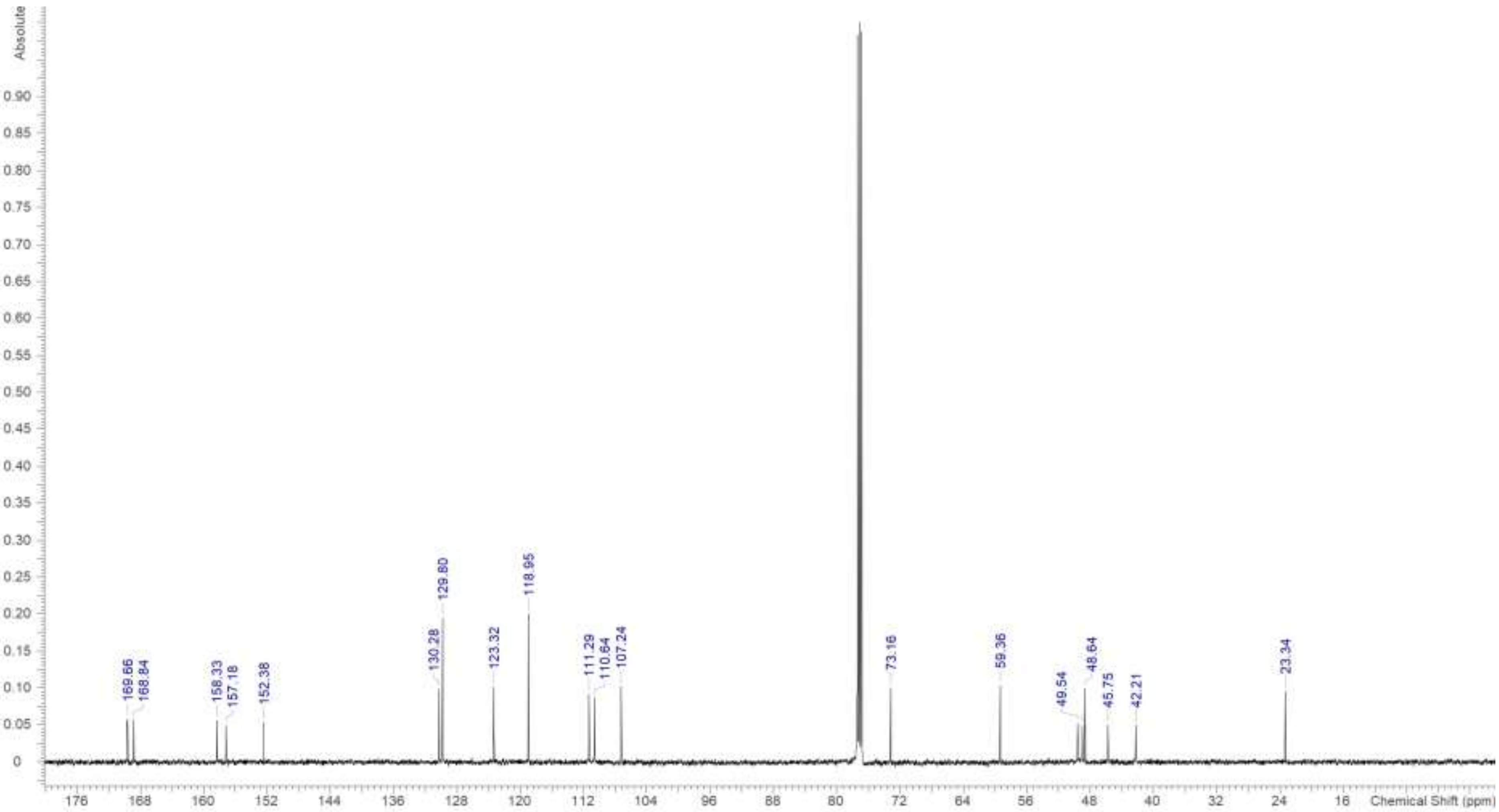

(*R*,*S*)-*N*-(3-methoxy-1-oxo-1-(4-(4-phenoxyphenyl)piperazin-1-yl)propan-2-yl)acetamide (*R*,*S*)-49 – ^{1}H NMR

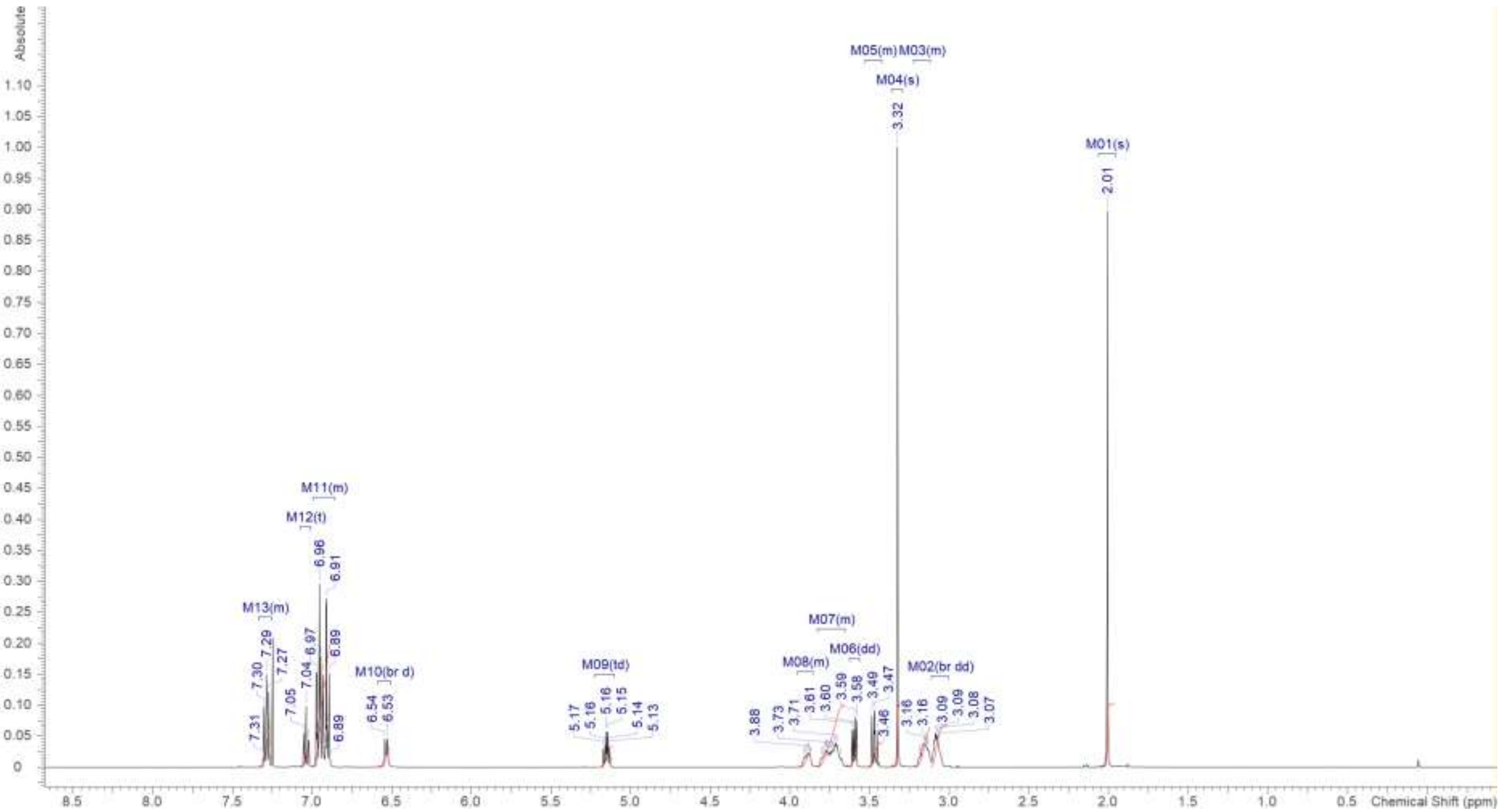

(*R*,*S*)-*N*-(3-methoxy-1-oxo-1-(4-(4-phenoxyphenyl)piperazin-1-yl)propan-2-yl)acetamide (*R*,*S*)-49 – ^{13}C NMR

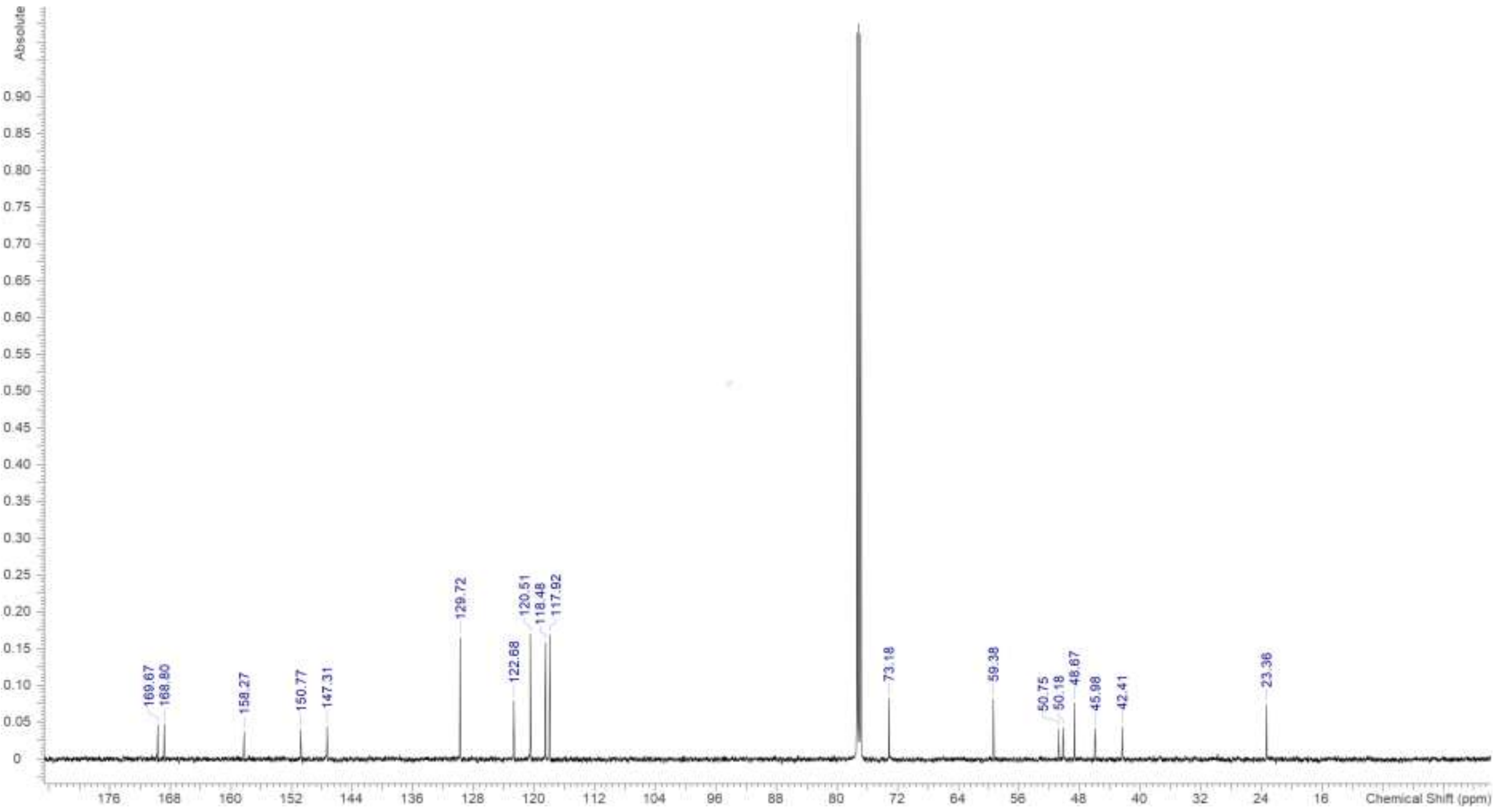

(*R,S*)-*N*-(3-methoxy-1-oxo-1-(4-(3-((trifluoromethyl)thio)phenyl)piperazin-1-yl)propan-2-yl)acetamide (*R,S*)-50 – ^{1}H NMR

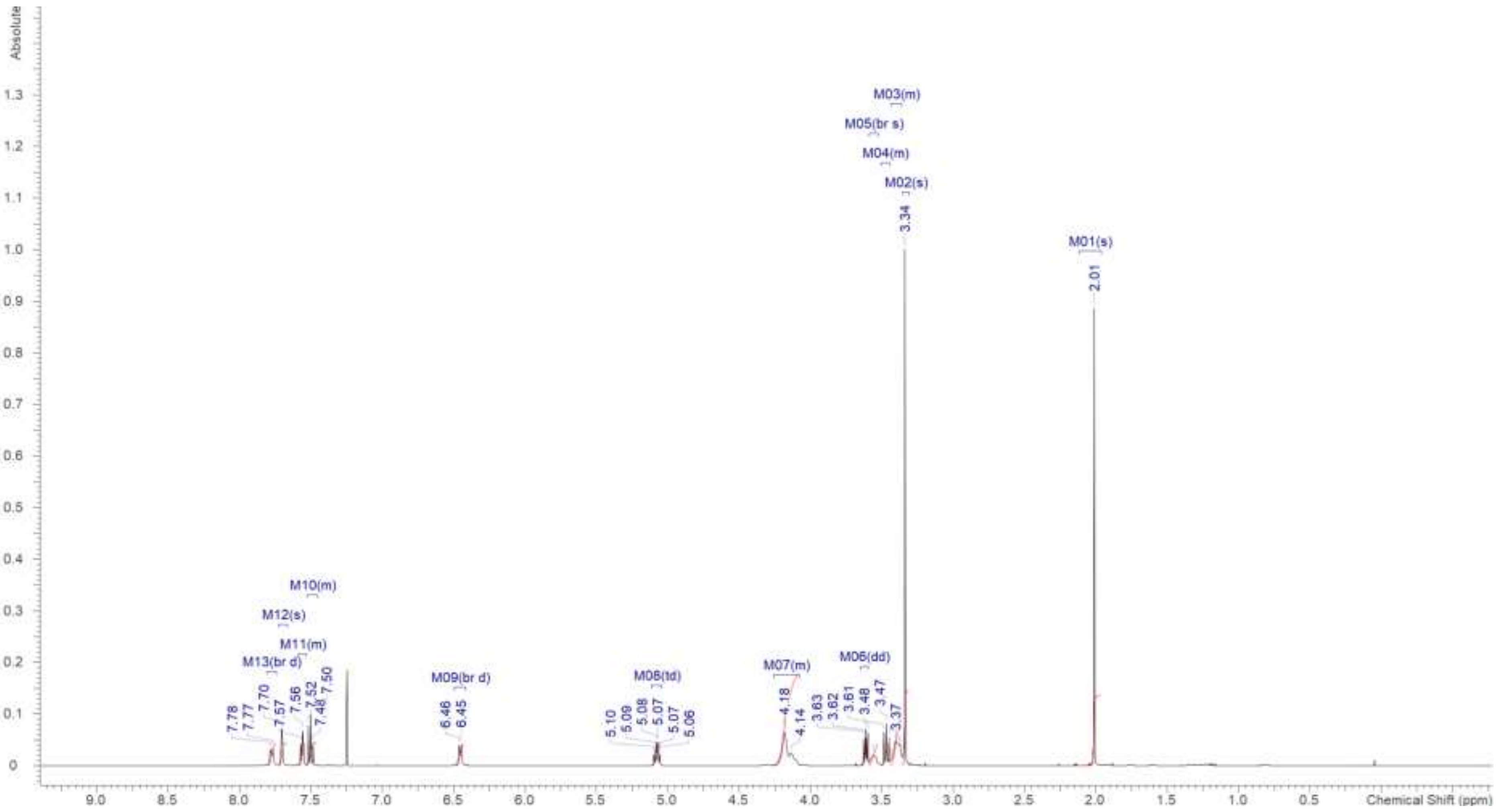

(*R*,*S*)-*N*-(3-methoxy-1-oxo-1-(4-(3-((trifluoromethyl)thio)phenyl)piperazin-1-yl)propan-2-yl)acetamide (*R*,*S*)-50 – ^{13}C NMR

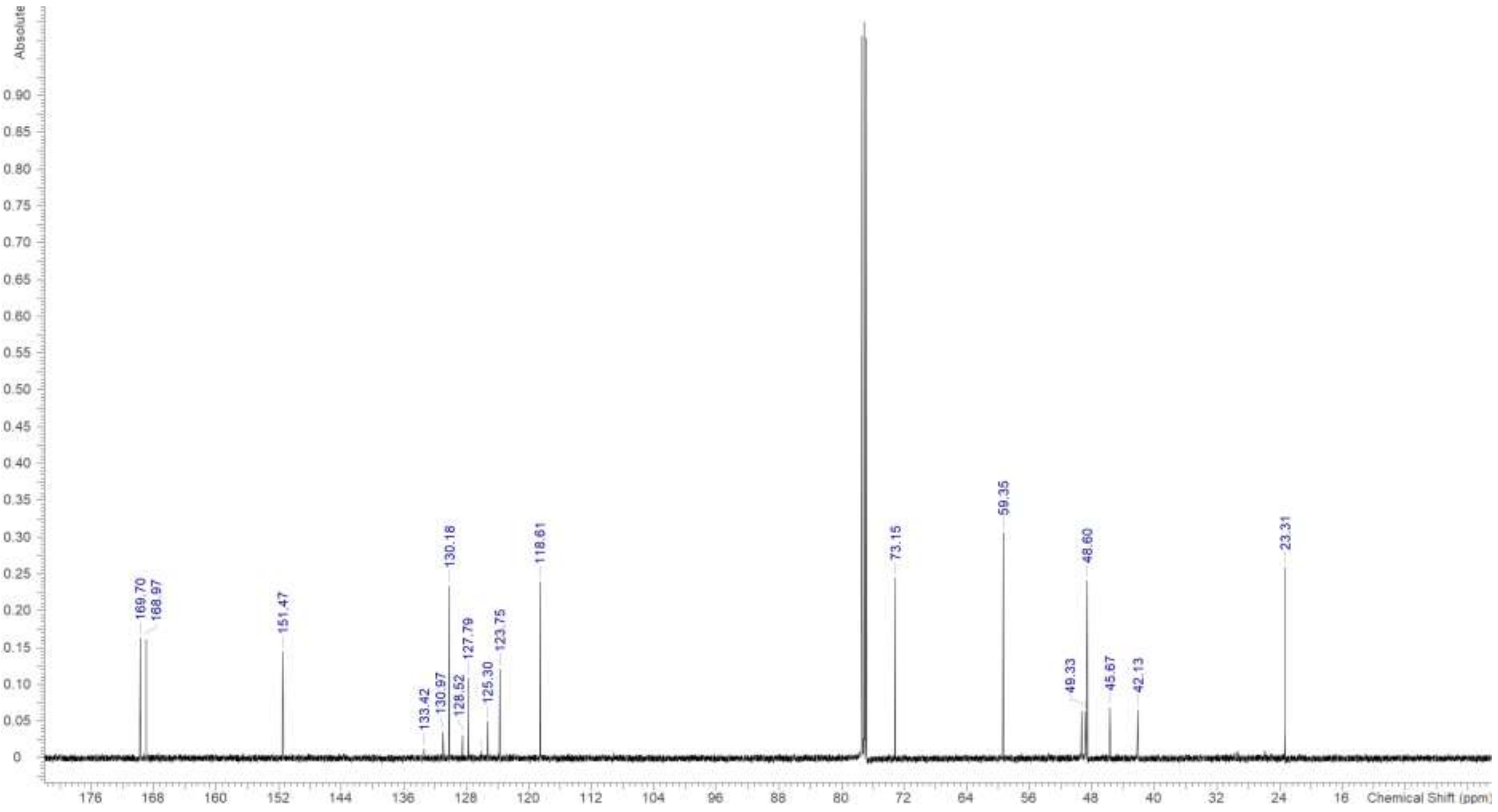

(*R*)-*N*-(3-methoxy-1-oxo-1-(4-(3-((trifluoromethyl)thio)phenyl)piperazin-1-yl)propan-2-yl)acetamide (*R*)-50 – ^{1}H NMR

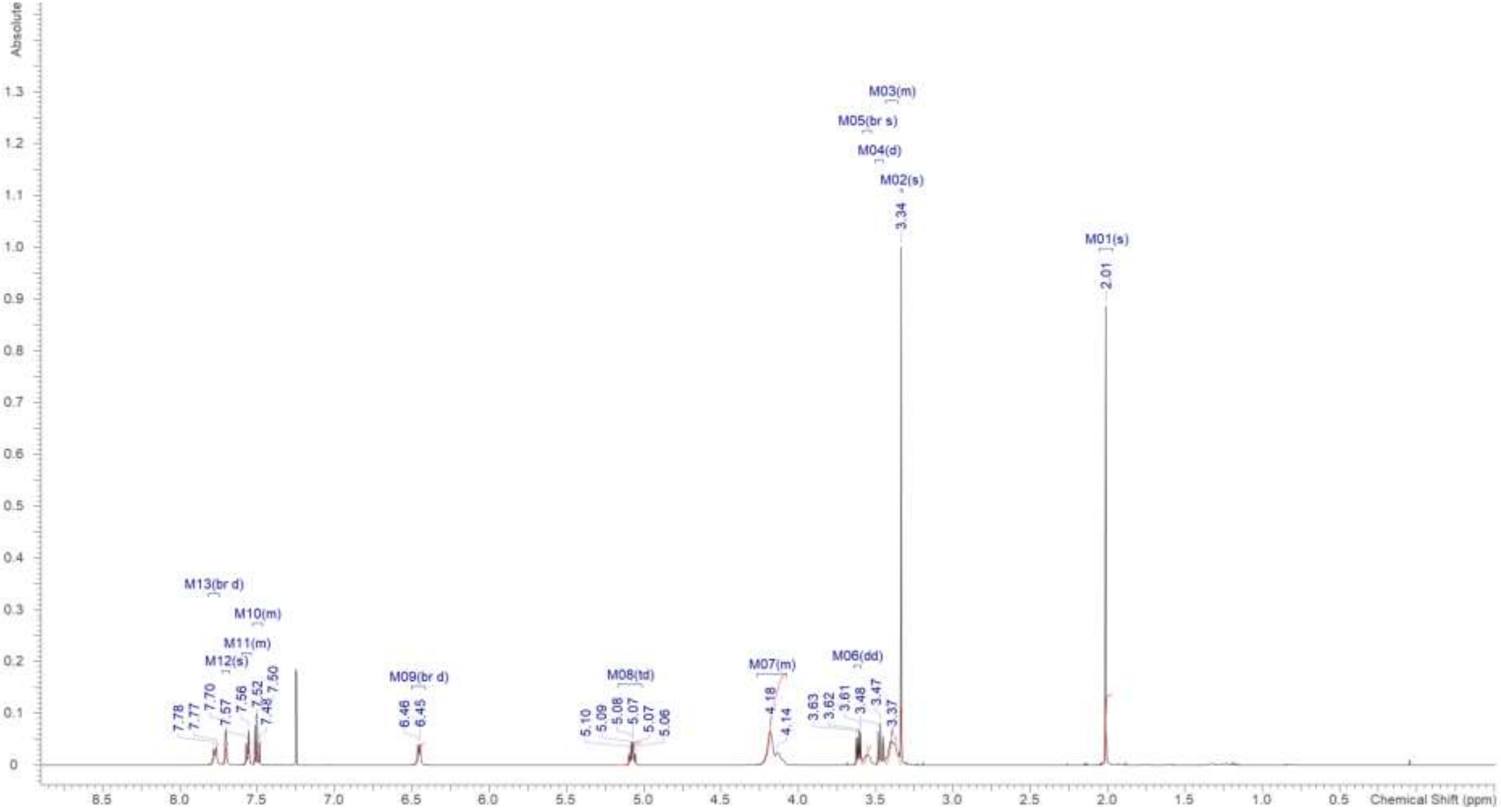

(*R*)-*N*-(3-methoxy-1-oxo-1-(4-(3-((trifluoromethyl)thio)phenyl)piperazin-1-yl)propan-2-yl)acetamide (*R*)-50 – ^{13}C NMR

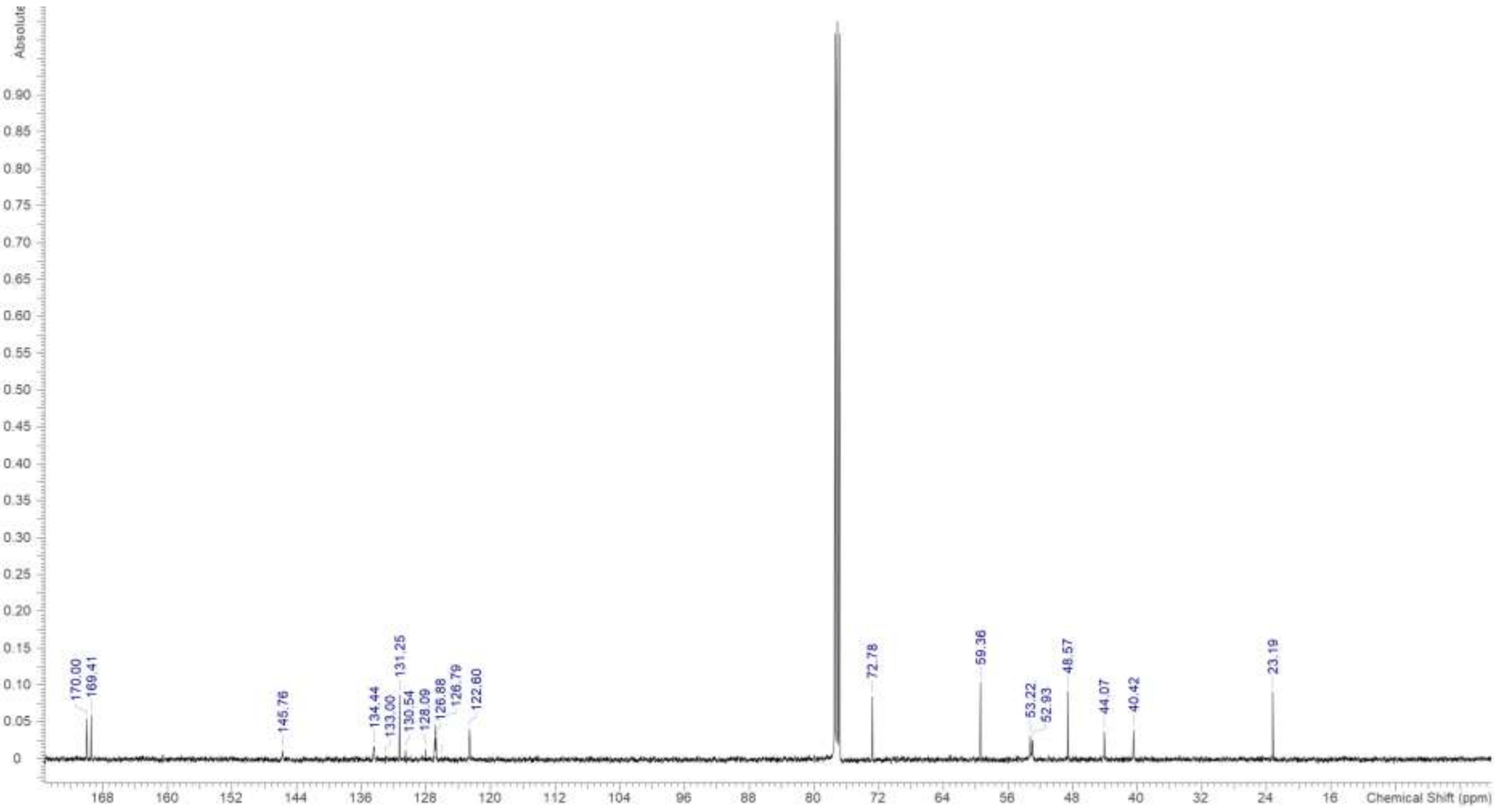

(*S*)-*N*-(3-methoxy-1-oxo-1-(4-(3-((trifluoromethyl)thio)phenyl)piperazin-1-yl)propan-2-yl)acetamide (*S*)-50 – ^{1}H NMR

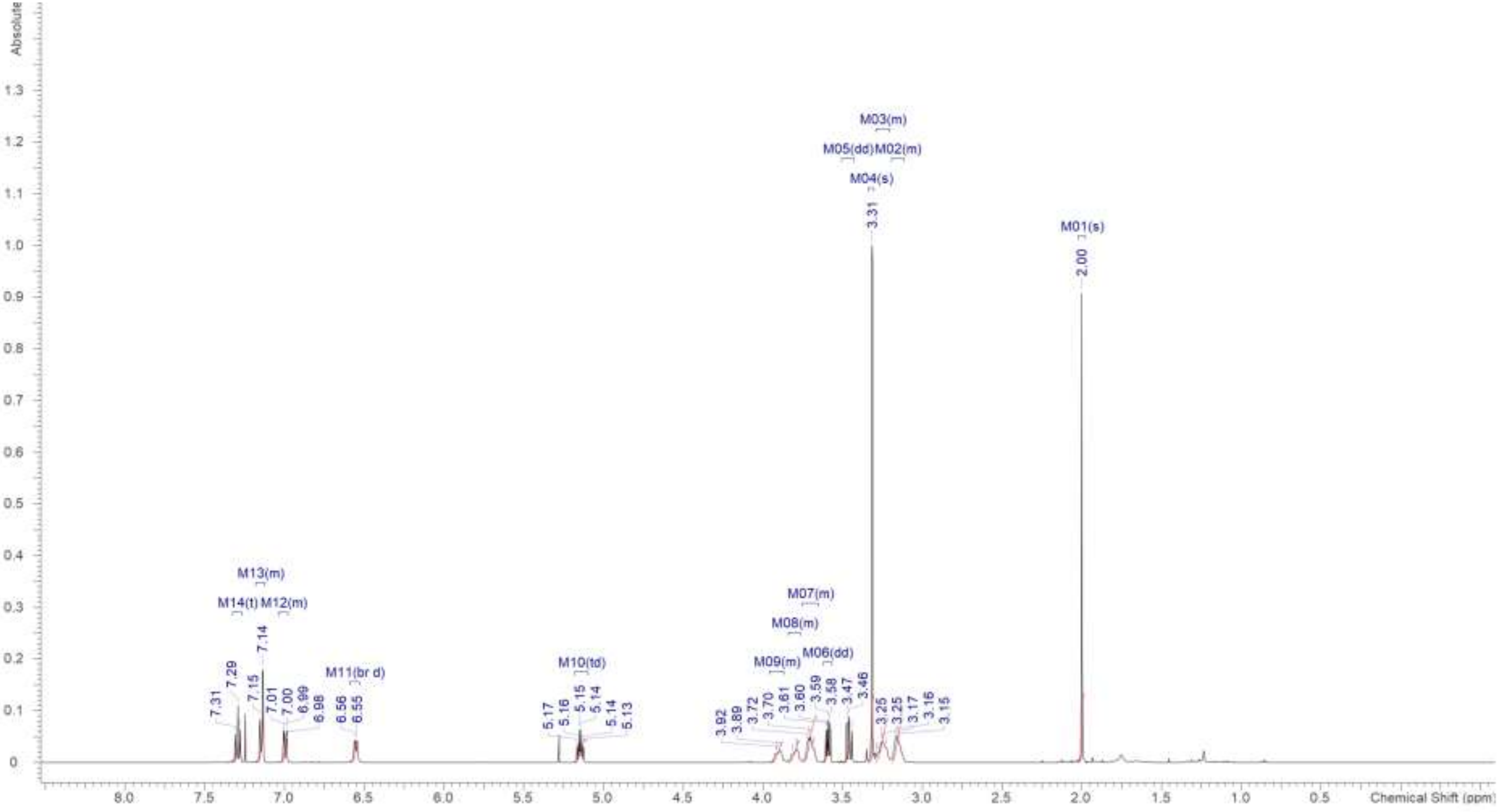

(*S*)-*N*-(3-methoxy-1-oxo-1-(4-(3-((trifluoromethyl)thio)phenyl)piperazin-1-yl)propan-2-yl)acetamide (*S*)-50 – ^{13}C NMR

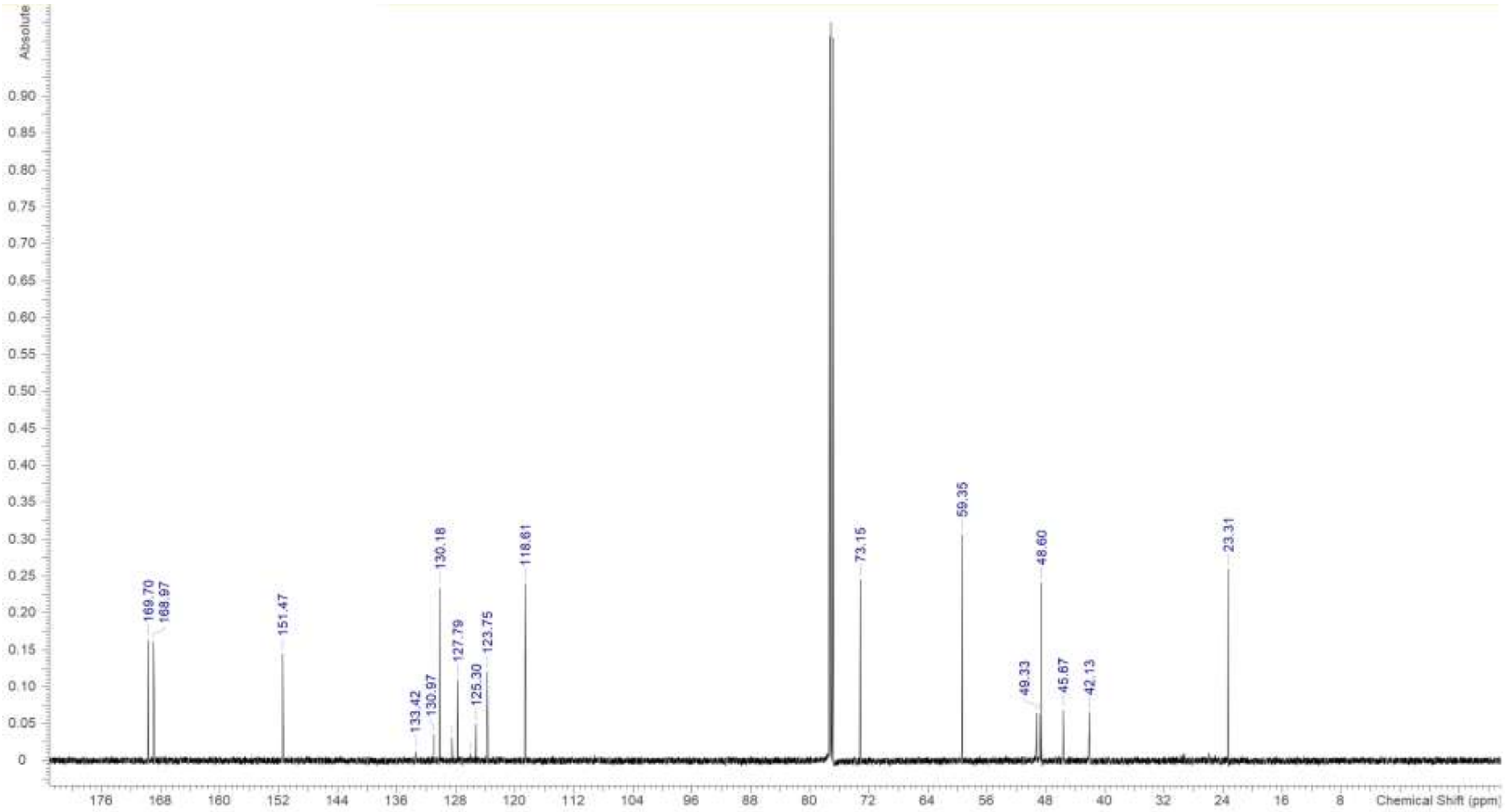

(*R*,*S*)-*N*-(3-methoxy-1-oxo-1-(4-(4-((trifluoromethyl)thio)phenyl)piperazin-1-yl)propan-2-yl)acetamide (*R*,*S*)-51– ^{1}H NMR

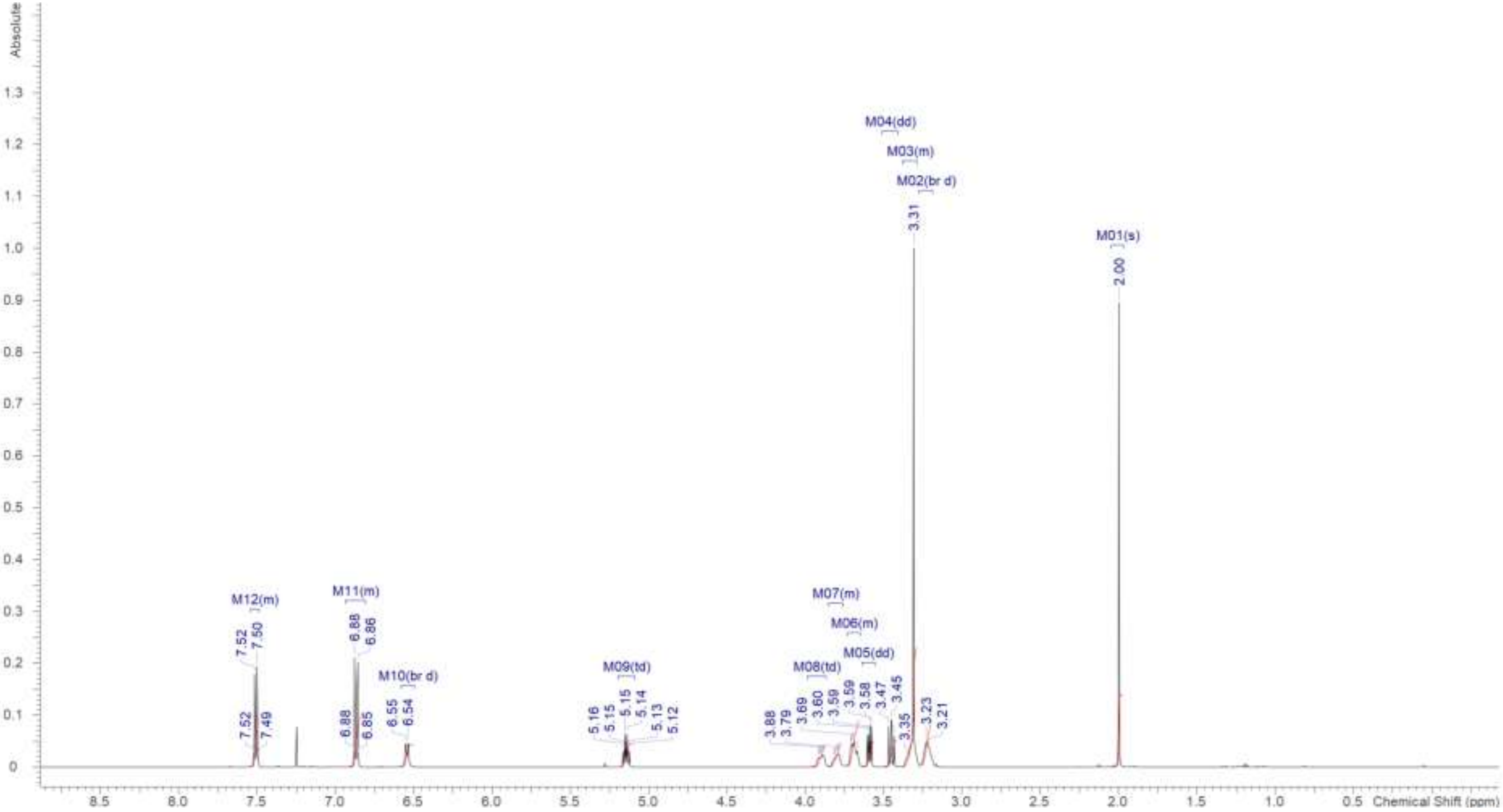

(*R,S*)-*N*-(3-methoxy-1-oxo-1-(4-(4-((trifluoromethyl)thio)phenyl)piperazin-1-yl)propan-2-yl)acetamide (*R,S*)-51 – ^{13}C NMR

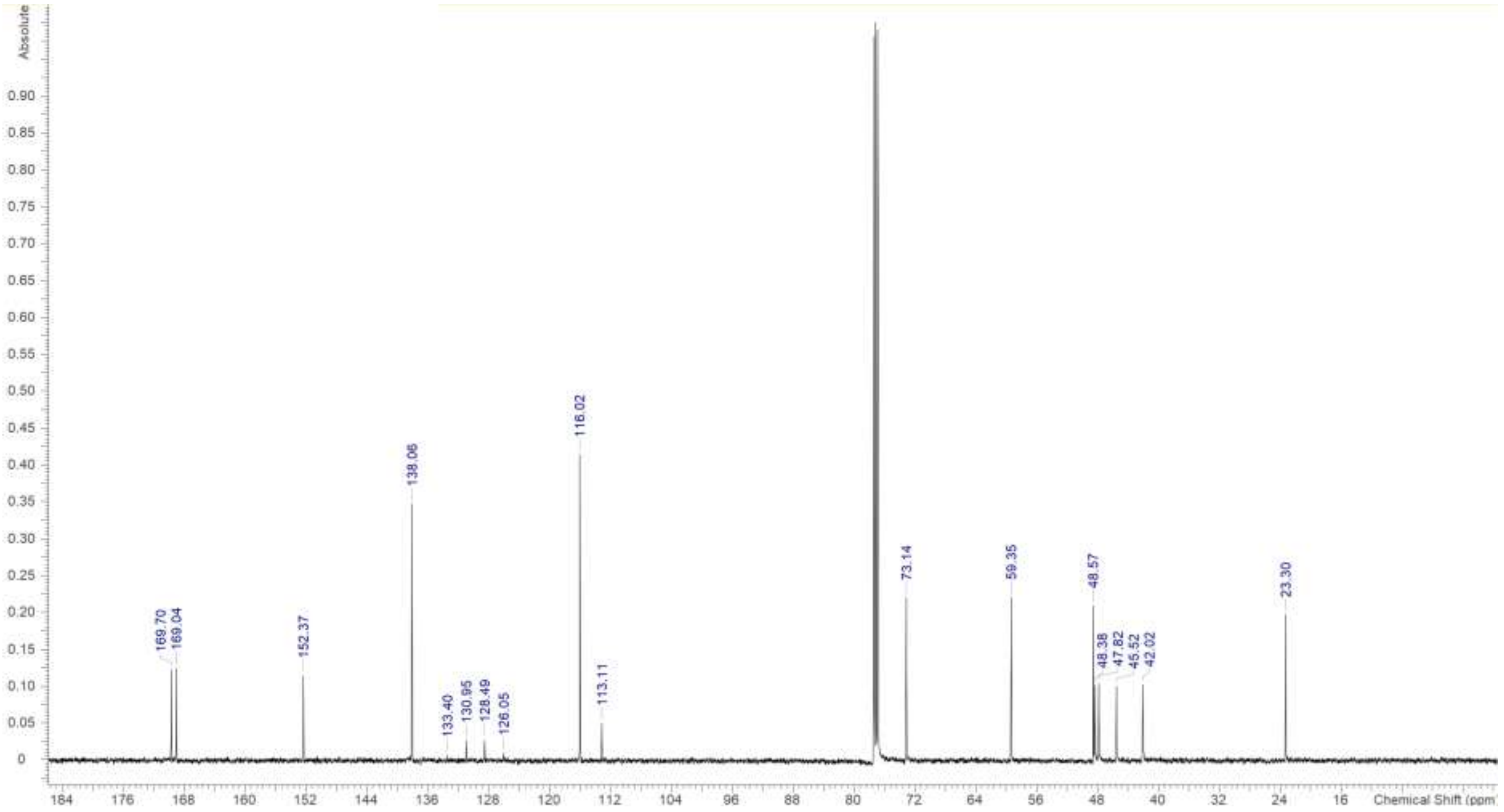

Supplement: Supplementary file 1 [file jm5c02093_si_001.pdf]
